# Nitrogenous compounds characterized in the deterrent skin extract of migratory adult sea lamprey from the Great Lakes region

**DOI:** 10.1371/journal.pone.0217417

**Published:** 2019-05-23

**Authors:** Amila A. Dissanayake, C. Michael Wagner, Muraleedharan G. Nair

**Affiliations:** 1 Department of Horticulture, Michigan State University, East Lansing, Michigan, United States of America; 2 Department of Fisheries and Wildlife, Michigan State University, East Lansing, Michigan, United States of America; Duke University Marine Laboratory, UNITED STATES

## Abstract

The sea lamprey (*Petromzons marinus*) is a devastating invasive species that represents a significant impediment to restoration of the Laurentian Great Lakes. There is substantial interest in developing environmentally benign control strategies for sea lamprey, and many other aquatic invasive species, that employ the manipulation of semiochemical information (pheromones and chemical cues) to guide the movements of invaders into control opportunities (e.g. traps, locations for safe pesticide application, etc.). A necessary precursor to the use of semiochemicals in conservation activities is the identification of the chemical constituents that compose the odors. Here, we characterize the major nitrogenous substances from the water-soluble fraction of a skin extract that contains the sea lamprey alarm cue, a powerful repellent that has proven effective in guiding the movements of migrating sea lamprey in rivers. Nitrogenous compounds are suspected components of fish alarm cues as the olfactory sensory neurons that mediate alarm responses transduce amino acids and related compounds. A laboratory assay confirmed the behavioral activity contained in the alarm cue resides in the water-soluble fraction of the skin extract. This water-soluble fraction consisted primarily of creatine (70%), heterocyclic nitrogenous compounds (4.3%) and free amino acids (18.4%), respectively. Among the free amino acids characterized in our study, essential amino acids constituted 13% of the water-soluble fraction. Free amino acids isolated from the water-soluble fraction composed of arginine, phenylalanine, threonine, and asparagine 3.9, 2.7, 2.6 and 2.4% of the water-soluble fraction, respectively. We discuss the implications of these findings for understanding the nature and use of the sea lamprey alarm cue in conservation activities.

## Introduction

Parasitic feeding by the invasive sea lamprey (*Petromyzon marinus*) remains the most significant source of non-fishing mortality for many fishes of the Laurentian Great Lakes and represents one of the greatest impediments to restoration of the world’s largest freshwater ecosystem. The United States and Canada currently expend more than $20 million (US) each year to suppress the invasive population by killing riverine larvae with lampricides before they metamorphose into parasites, and by maintaining dams that prevent migrating adults from entering hundreds of rivers with high-quality spawning habitat [1]. Though effective, there is considerable societal interest in reducing pesticide applications to the environment and reconnecting the Great Lakes to its tributaries via dam removal and fish passage [2]. Thus, the development and testing of new environmentally benign tactics for reducing reproductive success of the sea lamprey is a significant conservation goal [3].

Behavioral manipulation refers to pest management techniques that rely on exploiting an animal’s innate responses to environmental information [4]. By manipulating the presentation of sensory information, an animal is deceived into making a fitness reducing decision, such as entering an unproductive habitat. Semiochemicals represent one such class of manipulative information—molecules released by animals into the environment that are used to communicate with another individual to complete important tasks (e.g. reproductive pheromones) or that inadvertently broadcast public information about the environment (cues). The sea lamprey relies extensively on semiochemicals during its reproductive migration into rivers. Overlapping generations of stream-resident larvae release a cue that labels areas of past reproductive success and guides migrants from offshore into streams likely to support future generations [5–7]. Following stream selection, nesting sexually mature males release a pheromone to attract ovulating females and complete the act of spawning [8,9]. Both odors are attractive and reveal the presence of opportunities to maximize fitness by facilitating mate search [10] and restricting reproduction to habitats suitable to support newly hatched offspring [5–7].

Sea lamprey also produce a natural repellent, putatively an alarm cue emitted from damaged skin and other tissues [11–13]. By labeling the surrounding waters, alarm cues released from recently attacked or killed individuals operate as public information and notify conspecifics, and other taxa attuned to the cue, of the presence of predation risk [14–16]. Prey that detect an alarm cue typically exhibit antipredator behaviors including flight, avoidance, reduced activity, or shelter seeking [17–18]. During the transition from offshore waters to streams, migrating sea lampreys pass through an ecotone partly defined by a radically altered predator community. In rivers, these nocturnal migrants often move in close proximity to river shorelines to ensure entry into tributaries emitting larval odor [7,19]. Such movement tendencies may increase the likelihood of contact with mammalian shoreline predators, important nighttime consumers of diadromous fishes [20,21]. Migrating sea lamprey demonstrate a strong and consistent avoidance response to an alarm cue extracted from conspecifics [11,13,22] and will avoid areas of a natural stream activated with this substance [12,23,24]. The alarm cue has also proven effective in guiding sea lampreys towards trapping devices placed on a shoreline [25].

These findings have sparked considerable interest in the isolation and identification of the chemical compounds that compose the sea lamprey alarm cue for use as a repellent in conservation activities. The chemical nature of alarm substances contained in fish tissue is unresolved and has received sparse research activity [26,27], yet remains a high priority [28]. In the relatively well-studied Ostariophysan fishes, evidence has accrued to suggest these alarm substances include a variety of water soluble nitrogenous compounds including amino acids and oligopeptides [29,30], hypoxanthine-3-N-oxide and similar compounds containing the nitrogen-oxide functional group of purine-N-oxides [31–33], histamine [34], protein, possibly as a carrier molecule [35], and glycosaminoglycan chondroitin [36]. Further, mixtures of odorants are expected to compose each alarm cue, as full behavioral reactivity appears species specific, with partial overlap observed among related species [37,38] including lampreys [39,40].

Examination of the olfactory sensory neurons (OSNs) in fishes also suggests the potential for nitrogenous compounds to serve as constituents in fish alarm cue mixtures. The olfactory organ of fishes is innervated by three primary OSN morphotypes that occupy distinct layers in the olfactory epithelium: ciliated cells, microvillous cells, and crypt cells [26]. Crypt cells are sparsely distributed in superficial layer of the olfactory organ, and express V1R-type receptors that rely on cAMP-activated signaling during odorant detection, indicating transduction of amino acids and related nitrogenous compounds [41–44]. The crypt cell is the OSN that reacts to fish alarm cue extracts [36,45] and is unique to fishes, including elasmobranchs [46]. The sea lamprey olfactory system also contains three OSN morphotypes that are homologous to those observed in bony fishes and elasmobranchs [47–49]. In particular, the ‘short’ lamprey OSN is sparsely distributed, displays the characteristic egg-shape, and occupies the most superficial layer of the olfactory epithelium, each consistent with the description of crypt cells in more derived fishes [46,50]. These findings led Laframboise et al. [49] to suggest evolutionary conservation of the crypt cell from the Agnatha to the Gnathostomata. Further, (Green et al. [51]) report a chemotactic map of neural activity in response to odorants in three bulbar regions of the sea lamprey olfactory bulb that receive axons from the sea lamprey main olfactory epithelium and the accessory olfactory organ, the latter a feature unique to lampreys [52]. Each region was reactive to amino acids.

Based on this evidence we hypothesize the sea lamprey alarm cue contains water soluble nitrogenous compounds that are emitted from damaged skin that induce anti-predator behavior in migrating sea lamprey. In this study, we characterize the nitrogenous compounds contained in a previously reported aqueous ethanolic Soxhlet extract from lamprey skin that contains the repellent molecules [12,13,53]. First, we separated the extract into chloroform-soluble and chloroform-insoluble fractions; the chloroform-soluble fraction exhibits no behavioral reactivity and contains cholesterol esters, tri- and di-glycerides, cholesterol, free fatty acids and minor amounts of environmental pollutants [53]. We report evidence that the alarm cue is contained in the water-soluble (chloroform-insoluble) fraction and proceeded to fractionate, purify, and chemically characterize the major nitrogenous compounds that compose this mixture.

## Materials and methods

### Collection and preparation of sub-adult migratory sea lamprey for extraction and behavioral assays

Migrating sea lamprey were obtained from the annual spring trapping operations of the US Fish and Wildlife Service and the Canadian Department of Fisheries and Oceans in tributaries to Lake Huron (Cheboygan and Ocqueoc Rivers, Michigan, USA). After capture, government staff transported the lampreys to the U.S. Geological Survey’s Hammond Bay Biological Station (Millersburg, Michigan, USA; 45.4976906°N, 84.0363127°W) and placed them into 800 L tanks receiving continuous water from Lake Huron water (5–18°C depending on date). Lampreys were held until use in the behavioral assays, or for the collection of skin. Prior to removal of the skin, each animal was euthanized via anesthetic overdose (Ethyl 3-aminobenzoate methanesulfonate *aka* tricaine methanesulfonate *aka* MS-222, CAS No. 886-86-2) by immersion in a bath at a concentration of 10 mg L^-1^ until respiration ceased for five minutes, followed by decapitation. After death, the carcass was rinsed in deionized water and the skin removed with a scalpel. Skins were stored at -20°C until use in the extraction procedures. All procedures for lamprey maintenance, euthanasia, and processing were approved by the Michigan State University Institutional Animal Care and Use Committee (permit # AUF 01/14-007-00).

### Extraction of the skin odorants

Frozen sea lamprey skins 2.14 Kg (220 skins) were extracted in a Soxhlet apparatus with 80% ethanol and 20% RO water as solvent (~100 g of skins in each extraction with 600 mL of 80% ethanol for 6 hours). We did not use special precautions for Soxhlet extraction of sea lamprey skin based on our earlier observation that heating did not impact the odorants. The combined extract, evaporated under vacuum to remove ethanol, was lyophilized to yield aqueous ethanolic extract as a powder (41.9 g). Solvent partitioning of the aqueous ethanolic extract (41.7 g) resulted in chloroform-soluble (21.4 g) and insoluble (water-soluble) (20.3 g) fractions. Skin extract and fractions were stored at -80°C until further use.

### Confirmation of behavioral reactivity in the chloroform-insoluble fraction

To confirm the hypothesis that behavioral reactivity was confined to the chemical constituents captured in the chloroform-insoluble fraction, we examined the response of migratory-phase male sea lamprey to the extraction fractions using a standard laboratory space-use assay [12,13]. Specifically, we examined whether sea lampreys were repelled by the full skin extract (positive control) and the chloroform-insoluble fraction of the full skin extract, but were not repelled by the extraction solvent (negative control) or the chloroform-soluble fraction, as predicted. To accomplish this we observed the space-use of ten replicate groups of ten male sea lampreys (N = 10 for each stimulus odor) after exposure to the extraction fractions and the solvent control in a 5.0 m X 1.84 m section of a linear raceway at the Hammond Bay Biological Station (Millersburg, Michigan, USA; 45.4976906°N, 84.0363127°W). On a given night, five groups of ten male sea lampreys were stocked into the holding section of each raceway at 15:00 to acclimate the animals to the water. Males were chosen because (a) male and female sexually immature sea lamprey do not differ in their response to the extracted predator cue, and (b) the response does not attenuate in males at the onset of maturation, but does in females [13]. The first trial each of night began at ~22:00. A single trial lasted for 30 min and consisted of a 10 min pre-stimulus period (no odor) and a 20 min stimulus period when the odor was introduced into one half of the raceway. Prior to introducing the odor into the raceway, we mixed a stimulus odor into 400 ml of lake water collected from the raceway in a 500 mL Erlenmeyer flask that was continuously stirred with a 2 cm magnetic stir bar during release. We introduced the test odor/lake water mixture into one side of each raceway at the rate of necessary to achieve a 1:10^6^ dilution (by volume when mixed into one half of the discharge) with a laboratory-grade peristaltic pump (MasterFlex model 7533–20). To ensure no cross-contamination of odors we used separate sets of pump tubing for each stimulus odor. We observed lamprey movements in an adjacent room on video monitors and recorded their activity onto digital media. To analyze lamprey distributions, we recorded the position of each subject every 30 sec after the start of a trial by replaying the video and assigning each lamprey to the stimulus or non-stimulus side of the experimental arena based on the position of its head. The data from the final 10 min of the stimulus period was used to obtain the proportion of animals on the stimulus side of the raceway for each trial. The predictions relative to the solvent control were analyzed with two-tailed t-tests (α = 0.05, assuming equal variance) where the proportion of animals on the stimulus side of the raceway was the dependent variable. Normality was confirmed with Shapiro-Wilks tests (all P > 0.12).

### General procedures for chromatographic purification and spectroscopic analyses

Solvents used for isolation and purification steps were ACS reagent grade—Sigma-Aldrich Chemical Company (St. Louis, MO, USA). A CombiFlash MPLC purification system (Teledyne ISCO, Lincoln, NE, USA), equipped with C18 RediSep (86 g, C18 reverse phase) column, was used for the fractionation chloroform-insoluble fraction. Preparative HPLC (LC-20, Japan analytical industry Co., Ltd, Tokyo, Japan) equipped with XTerra Prep MS C-8 column (10 μm, 19 x 250 mm, Waters Corporation, Milford, MA, USA) was used for the purification of CombiFlash MPLC fractions. NMR spectra were recorded on 500 MHz (Varian Unity ±500, ^1^H NMR) and 125 MHz (Varian Unity ±500, ^13^C NMR) VRX instruments. Unless specified, D_2_O was used as the solvent for NMR experiments. HR-ESITOFMS spectra of pure isolates were recorded on a Waters Xevo G2-S QTOF LC mass spectrometer (Waters Corporation, Milford, MA, USA).

### Optical rotation measurements

Optical rotations was determined on a PerkinElmer model 341 polarimeter at 20°C and 589 nm according to published procedure [54]. The specific rotations were calculated according to the equation [α]^20^_D_ = (100α)/(l × *c*) where l is the path length in decimeters and *c* is the concentration in g/100 mL.

### Chromatographic purification and isolation of pure compounds in sea lamprey skin ethanolic extract

**Fig 1:**
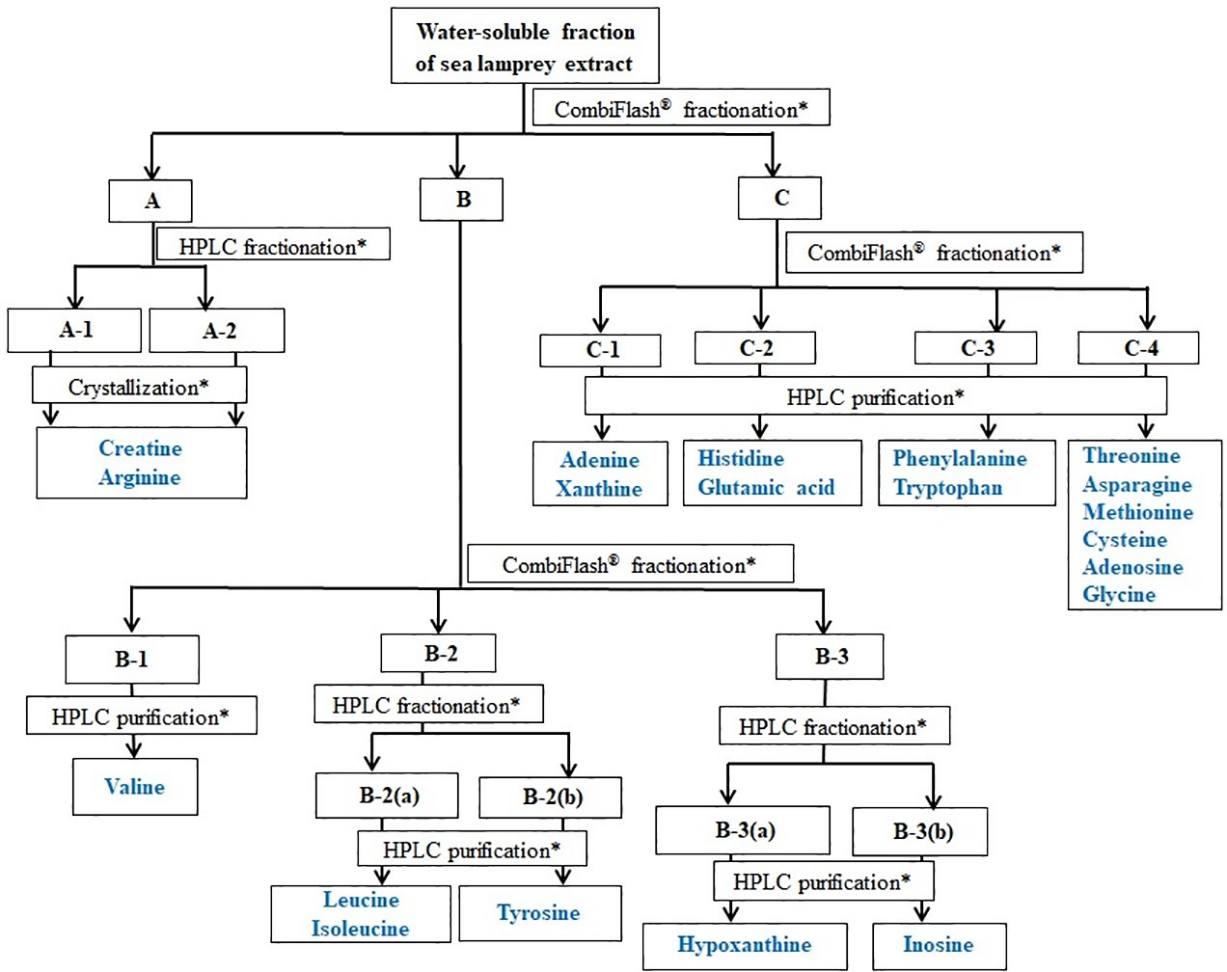
Fractionation and purification of components from the deterrent water-soluble fraction of sea lamprey skin extract. *Details are in the experimental section.

**Fig 2:**
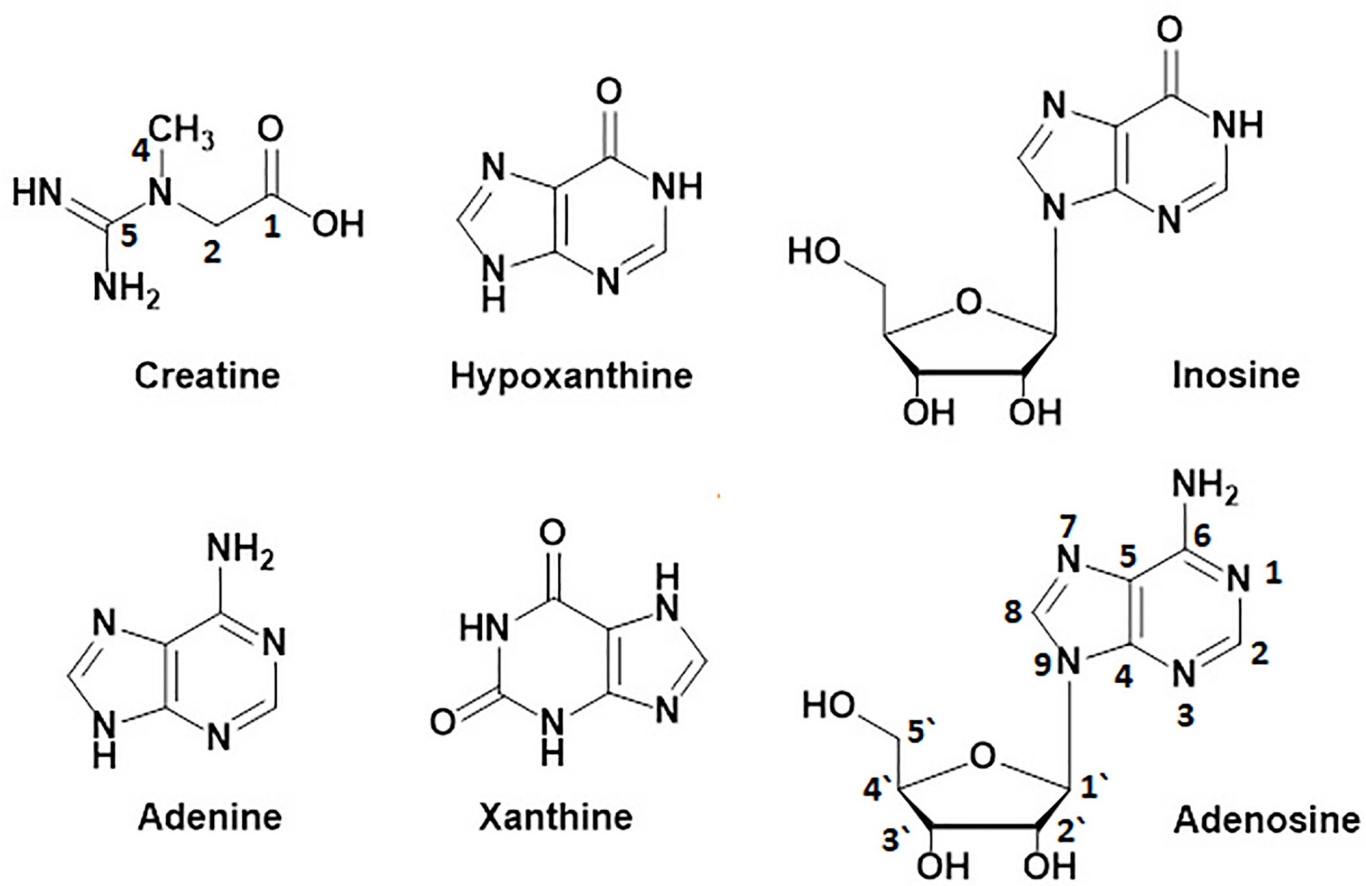
Chemical structures of other nitrogenous compounds isolated from the deterrent water-soluble fraction of the sea lamprey skin extract: creatine, hypoxanthine, inosine, adenine, xanthine, and adenosine.

**Fig 3:**
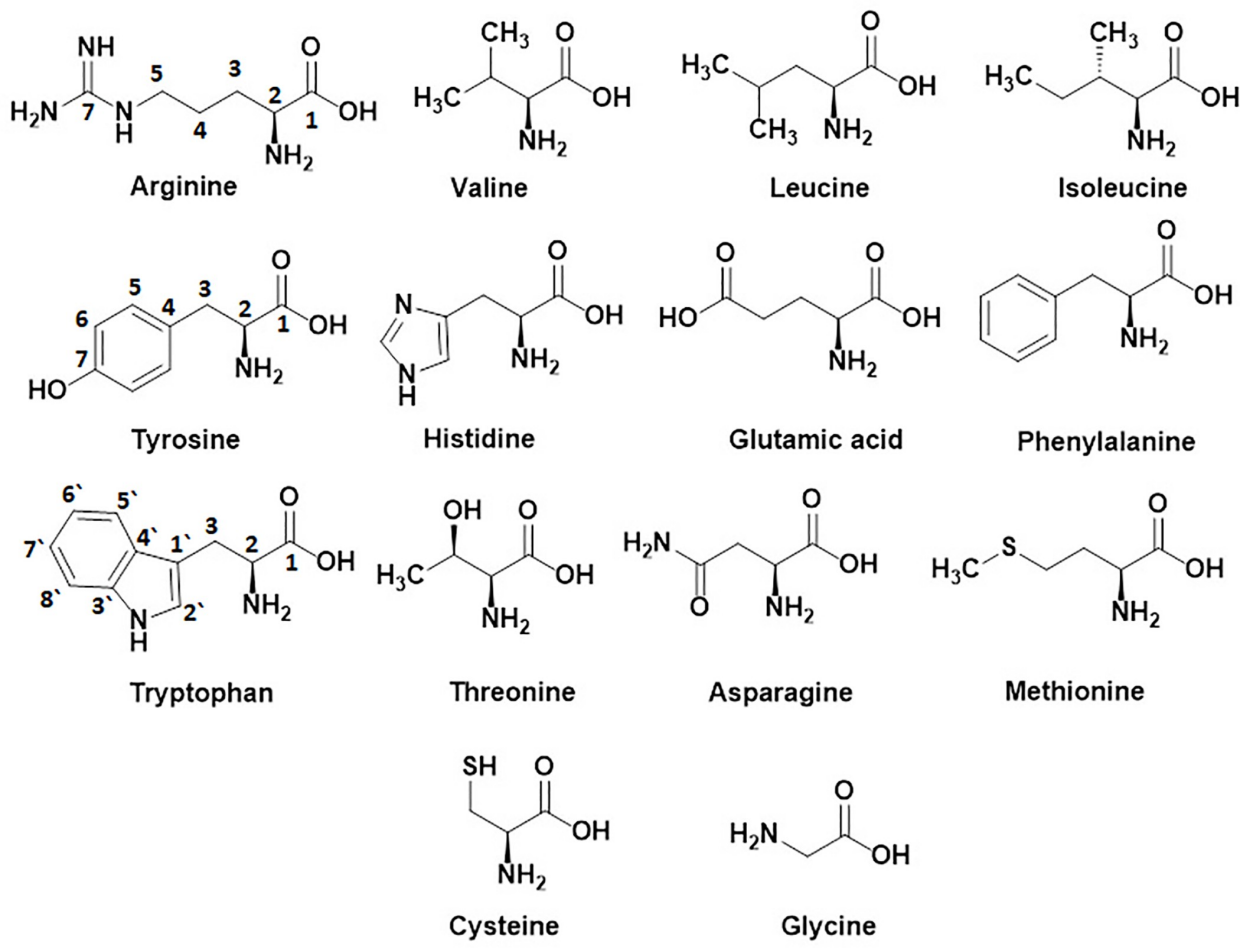
Chemical structures of free amino acids isolated from the deterrent water-soluble fraction of the sea lamprey skin extract: arginine, valine, leucine, isoleucine, tyrosine, histidine, glutamic acid, phenylalanine, tryptophan, threonine, asparagine, methionine, cysteine, and glycine.

An aliquot (20.1 g) of the chloroform-insoluble fraction was further fractionated by CombiFlash MPLC purification system by eluting with water:methanol step gradients (8:2, 1:1, 3:7 *v/v*) and finally with methanol (100%) gave fractions that were then combined based on similar UV profile. The resulting fractions were **A** (14.4 g), **B** (1.88 g) and **C** (3.74 g), respectively (**Fig 1**, Figure A in S1 File). An aliquot of the fraction **A** (1.4 g), further fractionated by HPLC and eluting with water:methanol 95:5 *v/v*, yielded three sub-fractions, **A-1** (1.06 g), **A-2** (172 mg) and **A-3** (168 mg), respectively (**Fig 1**, Figure D in S1 File). Fraction **A-1** (102 mg), crystallized from water:methanol twice, afforded creatine (94 mg, **Fig 2**, Figures A-D in S2 File) [55]. Similarly, an aliquot of fraction **A-2** (104 mg), purified by crystallization from water:acetone, yielded another batch of creatine (56 mg) and arginine (47 mg, **Fig 3**, Figures E-H in S2 File) [56–58]. Fractions **A-3** was a complex mixture as indicated by HPLC profile and hence kept aside.

An aliquot of fraction **B** (1.75 g), fractionated on CombiFlash MPLC purification system by eluting with water:methanol gradient (9:1, 8:2, 7:3, 6:4 and 1:1, *v/v*), yielded sub-fractions **B-1** (520 mg), **B-2** (420 mg) and **B-3** (680 mg), respectively (**Fig 1**, Figure B in S1 File). The HPLC purification of fraction **B-1** (400 mg) by elution with water:methanol (95:5 *v/v*) under isocratic conditions yielded sub-fractions **B-1(a)** (32 mg), **B-1(b)** (90 mg), **B-1(c)** (148 mg), **B-1(d)** (33 mg), **B-1(e)** (78 mg) and **B-1(f)** (25 mg), respectively (**Fig 1**, Figure E in S1 File). Fraction **B-1(c)** (65 mg), purified by crystallization from water:methanol, afforded another batch of creatine (48 mg) [55]. Purification of fraction **B-1(e)** (75 mg) by HPLC and elution with water:methanol (95:5 *v/v*) (4 mL/min) under isocratic conditions yielded valine (71 mg, **Fig 3**, Figures I-L in S2 File) [56–58]. Fractions **B-1(a)**, **B-1(b)**, **B-1(d)** and **B-1(f)**, complex mixtures as indicated by HPLC profile, were in minute quantities and hence kept aside. Similarly, fraction **B-2** (370 mg) was further fractionated by HPLC by eluting with water:methanol 95:5 *v/v* (4 mL/min) under isocratic condition afforded fractions **B-2(a`)** (40 mg), **B-2(a)** (215 mg) and **B-2(b)** (115 mg), respectively (**Fig 1**, Figure F in S1 File). Fraction **B-2(a`)**, a complex mixture as indicated by HPLC profile, was in small quantity and hence kept aside. An aliquot of fraction **B-2(a)** (190 mg), purified by HPLC by eluting with water:methanol 98:2 *v/v*, 2 mL/min under isocratic conditions yielded leucine (120 mg, 32 min, **Fig 3**, Figures M-P in S2 File) [56–58] and isoleucine (54 mg, 38 min, **Fig 3**, Figures Q-T in S2 File) [56–58]. Similarly, aliquot of fraction **B-2(b)** (100 mg), purified by HPLC and eluting with water:methanol 98:2 *v/v* (2 mL/min) under isocratic condition yielded isoleucine (35 mg) and **6** (tyrosine, 26 mg, 42 min, **Fig 3**, Figures U-X in S2 File) [56–58]. An aliquot of fraction **B-3** (400 mg), fractionated by HPLC by eluting with water:methanol 95:5 *v/v* (3 mL/min) under isocratic conditions yielded three fractions **B-3(a`)** (59 mg), **B-3(a)** (255 mg) and **B-3(b)** (80 mg), respectively (**Fig 1,** Figure G in S1 File). Fraction **B-3(a`)** was a complex mixture, as indicated by HPLC profile, and in very small quantity. Due to inadequate quantity, we did not analyze it further. Fraction **B-3(a)** (140 mg), purified by HPLC by eluting with water:methanol 98:2 *v/v* (3 mL/min) under isocratic conditions, yielded hypoxanthine (122 mg, 65 min, **Fig 2**, Figures A-D in S3 File) [59]. Purification of fraction **B-3(c)** (35 mg) under same conditions yielded inosine (22 mg, 82 min, **Fig 2**, Figures E-H in S3 File) [59].

Fraction **C** (2.1 g), fractionated on CombiFlash MPLC purification system by eluting with water:methanol (7:3, 6:4, 1:1 *v/v* and 100% methanol) yielded four fractions **C-1** (130 mg), **C-2** (235 mg), **C-3** (630 mg) and **C-4** (1.07 g), respectively (**Fig 1**, Figures C and H-N in S1 File). An aliquot of fraction **C-1** (120 mg), purified by HPLC and eluting with water:methanol 85:15 *v/v* (3 mL/min) under isocratic conditions yielded adenine (38 mg, 55 min, **Fig 2**, Figures I-L in S3 File) [60] and xanthine (78 mg, 66 min, **Fig 2**, Figures M-P in S3 File) [60]. Fraction **C-2** (160 mg), purified by HPLC (water:methanol 95:5 *v/v*, 4 mL/min) under isocratic conditions gave histidine (30 mg, 55 min, **Fig 3**, Figures Q-T in S3 File) [56–58] and glutamic acid (15 mg, 74 min, **Fig 3**, Figures U-X in S3 File) [56–58]. Purification of an aliquot of fraction **C-3** (100 mg) by HPLC (water:methanol 95:5 *v/v*, 2.2 mL/min) under isocratic conditions afforded phenylalanine (48 mg, 51 min, **Fig 3**, Figures A-D in S4 File) [56–58] and tryptophan (20 mg, 84 min, **Fig 3**, Figures E-H in S4 File) [56–58]. Fraction **C-4** (300 mg), fractionated by HPLC by eluting with water:methanol 80:20 *v/v* (3 mL/min) gave three fractions **C-4(a)** (159 mg), **C-4(b)** (87 mg) and **C-4(c)** (48 mg), respectively (**Fig 1**, Figures L-N in S1 File). Purification of **C-4(a)** (155 mg) by HPLC (water:methanol 90:10 *v/v*, 4 mL/min, isocratic) gave threonine (82 mg, 41 min, **Fig 3**, Figures I-L in S4 File) [56–58] and asparagine (77 mg, 47 min, **Fig 3**, Figures M-P in S4 File) [56–58]. Similarly, purification of **C-4(b)** (85 mg) under identical conditions yielded methionine (27 mg, 35 min, **Fig 3**, Figures Q-T in S4 File) [56–58] and cysteine (55 mg, 42 min, **Fig 3**, Figures U-X in S4 File) [56–58]. Similarly, fraction **C-4(c)** (45 mg), purified by HPLC (water:methanol 90:10 *v/v*, 3 mL/min, isocratic) yielded adenosine (32 mg, 46 min, **Fig 2**, Figures A-D in S5 File) [56–58] and glycine (16 mg, 53 min, **Fig 3**, Figures E-H in S5 File) [56–58].

## Results

**Fig 4:**
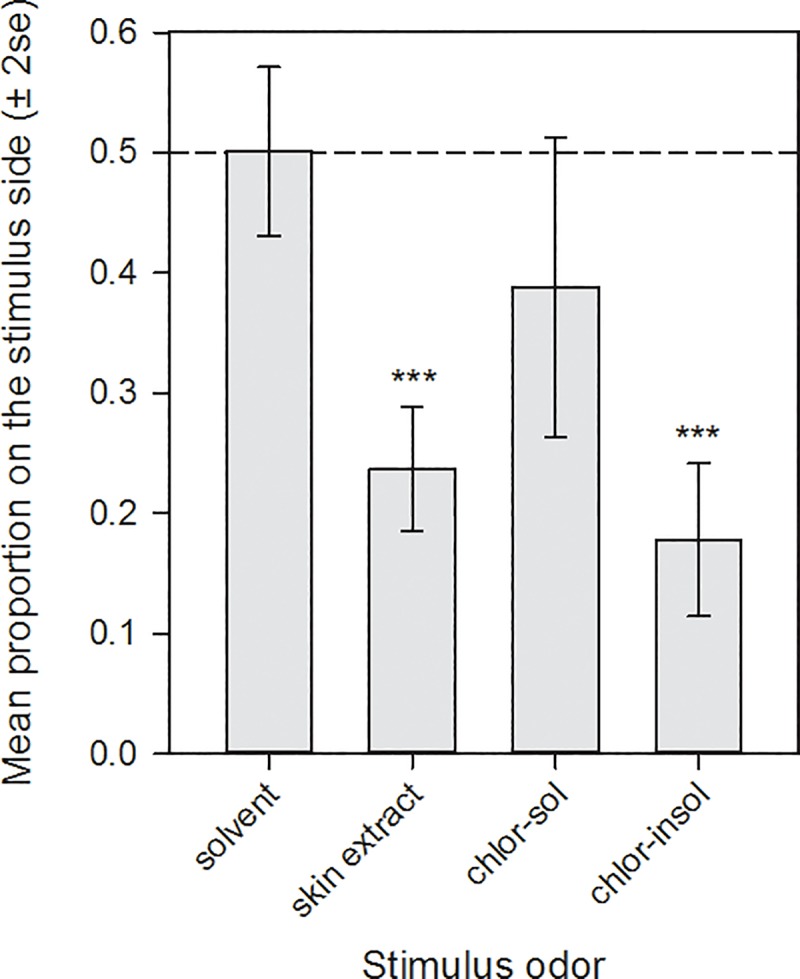
**The mean proportion (± 2 se) of migratory-phase sea lampreys on the side of a laboratory raceway receiving one of four stimulus odors:** (a) a solvent control, (b) the full aqueous ethanolic extract from sea lamprey skin, (c) the chloroform-soluble fraction of the aqueous ethanolic skin extract, and (d) the chloroform-insoluble (water-soluble) fraction of the aqueous ethanolic skin extract (*** indicates P < 0.0001 in t-tests of the mean reaction vs. the solvent control).

The lampreys exhibited no preference response to the solvent control (proportion on the stimulus side, mean ± 2 SE, 0.501 ± 0.07; **Fig 4**). As predicted, migratory-phase male sea lamprey were repelled by the full skin extract (vs. solvent control, t_1,18_ = 6.06, P < 0.0001), and the chloroform-insoluble fraction (vs. solvent control, t_1,18_ = 6.83, P < 0.0001). The lampreys were not significantly repelled by the chloroform-soluble fraction (vs. solvent control, t_1,18_ = 1.58, P = 0.13).

Therefore, fractionation and purification of adult sea lamprey deterrent water-soluble fraction of skin extract, prepared by Soxhlet extraction of the skin with 80% aqueous ethanol for 6 hours [6], were carried out with medium pressure liquid chromatography (MPLC, CombiFlash) and preparative HPLC. The chemical identity of all pure isolates were determined by ^1^H- and ^13^C-NMR and HRESIMS experiments. The ^1^H- (500 MHz) and ^13^C- NMR (125 MHz) chemical shift values presented below for each pure isolate are expressed in ppm, based on the residual chemical shift values for D_2_O at 3.79 ppm, and for DMSO-d_6_ at 2.50 and 39.9 ppm, respectively. NMR (^1^H and ^13^C) spectral data of the isolated compounds are summarized in Tables 1 and 2 and HRESIMS and optical rotation data in Table 3. The spectral and optical rotation data of the isolates were identical to the spectral and optical rotation data for authentic samples of L-amino acids (Fig 3) and nitrogenous compounds (Fig 2) [55–61].

**Table 1:** ^1^H and ^13^C NMR spectral data of amino acids ^a^^,^^b^.

no.	Arginine^a^		Valine^a^		Leucine^a^	
	*δ*_H_ (multi, *J* in Hz)	δ_C_	*δ*_H_ (multi, *J* in Hz)	*δ*_C_	*δ*_H_ (multi, *J* in Hz)	*δ*_C_
1	-	174.3	-	174.2	-	175.6
2	3.16 (t, 6.3)	54.2	3.57 (t, 4.9)	60.2	3.69 (t, 4.9)	53.2
3	1.74–1.76 (m)	27.4	2.21–2.26 (m)	28.9	1.66–1.69 (m) ^c^	39.7
4	1.51–1.53 (m)	23.8	1.0 (t, 6.8)	17.8		20.7
5	3.08 (t, 6.9)	40.4	0.95 (t, 6.8)	16.5	0.91 (d, 5.9) ^c^	24.0
6	-	156.6				21.9
	**Tyrosine**^a^		**Histidine**^a^		**Glutamic acid**^a^	
	*δ*_H_ (multi, *J* in Hz)	*δ*_C_	*δ*_H_ (multi, *J* in Hz)	*δ*_C_	*δ*_H_ (multi, *J* in Hz)	*δ*_C_
1	-	174.2	-	173.9	-	173.4
2	3.89 (m)	56.0	3.91 (dd, 7.8, 4.4)	54.7	3.76 (dd, 6.4 and 6.4)	53.7
3	3.17 (m)/3.02 (m)	35.5	3.03 (m)/3.14 (m)	28.1	2.48–2.51 (m) ^c^	25.4
4	-	126.7	-	132.1	2.06–2.12 (m) ^c^	29.9
5	6.86 (d, 8.6)	115.7	6.97 (d, 1)	116.6	-	176.9
6	7.12 (d, 8.6)	130.7	-	-		
7	-	154.9	7.66 (d, 1)	136.2		
	**Threonine**^a^		**Asparagine**^a^		**Methionine**^a^	
	*δ*_H_ (multi, *J* in Hz)	*δ*_C_	*δ*_H_ (multi, *J* in Hz)	*δ*_C_	*δ*_H_ (multi, *J* in Hz)	*δ*_C_
1	-	172.7	-	174.3	-	174.1
2	4.18–4.23(m)	60.3	3.98 (m)	51.1	3.81 (dd, 6.8 and 6.8)	53.7
3	3.54 (d, 4.9)	65.8	2.93 (m)/2.81 (m)	43.3	2.05–2.16 (m) ^c^	29.5
4	1.28 (d, 6.4)	19.3	-	173.2	2.56–2.59 (m) ^c^	28.7
5					-	-
6					2.09 (s)	13.8
	**Isoleucine**^a^		**Phenylalanine**^a^		**Cysteine**^b^	
	*δ*_H_ (multi, *J* in Hz)	*δ*_C_	*δ*_H_ (multi, *J* in Hz)	*δ*_C_	*δ*_H_ (multi, *J* in Hz)	*δ*_C_
1	-	174.1	-	173.9	-	170.1
2	3.61 (d, 3.9)	59.4	3.95 (dd, 7.8 and 5.3)	55.9	4.29 (dd, 5.8 and 4.4)	54.3
3	1.91–1.94 (m)	35.7	3.08 (m)/3.26 (m)	36.2	3.12 (m)/ 3.07(m)	23.8
4	1.40 (m)/1.21 (m)	25.4	-	134.9		
5	0.88 (dd, 3.0 and 3.9)	10.9	7.28–7.33 (dd, 7.9 and 2.0)	129.2		
6	0.95 (d, 7.3)	14.5	7.39–7.41 (dd, 8.3 and 1.0)	128.9		
7			7.35–7.37 (dd, 7.7 and 1.5)	127.6		
	**Tryptophan**^a^		**Glycine**^a^			
	*δ*_H_ (multi, *J* in Hz)	*δ*_C_	*δ*_H_ (multi, *J* in Hz)	*δ*_C_		
1	-	174.4	-	172.3		
2	3.99 (dd, 8.3 and 4.9)	54.9	3.51 (s)	41.4		
3	3.44 (m)/ 3.26 (m)	26.3				
1`	7.26 (s)	124.9				
2`	-	107.4				
3`	-	126.5				
4`	-	136.2				
5`	7.49 (d, 8.3)	111.8				
6`	7.68 (d, 8.4)	188.3				
7`	7.15 (dd, 6.9 and 6.9)	199.3				
8`	7.24 (dd, 8.3 and 8.3)	122.0				

^a^ Data were measured in D_2_O.

^b^ Data were measured in DMSO.

^c^ Overlapped signals.

**Table 2:** ^1^H and ^13^C NMR spectral data of nitrogenous compounds ^a^^,^^b^.

no.	Adenosine^b^		Inosine^b^		Xanthine^b^	
	*δ*_H_ (multi, *J* in Hz)	*δ*_C_	*δ*_H_ (multi, *J* in Hz)	*δ*_C_	*δ*_H_ (multi, *J* in Hz)	*δ*_C_
1	-	-	-	-	-	-
2	8.12 (s)	152.4	8.08 (s)	145.9	-	164.2
3	-	-	-	-	-	-
4	-	149.1	-	148.2	-	160.2
5	-	-	-	124.4	-	116.8
6	-	156.2	-	156.6	-	161.9
7	-	-	-	-	-	-
8	8.34 (s)	139.9	8.34 (s)	138.7	7.92 (s)	151.4
9	-	-	-	-	-	-
1`	4.86 (d, 6.4)	87.9	5.83 (d, 5.8)	87.4		
2`	4.58 (dd, 11.3 and 5.9)	73.4	4.44 (dd, 5.3 and 5.3)	74.1		
3`	4.13 (dd, 7.8 and 4.9)	70.7	4.10 (dd, 4.9 and 3.5)	70.3		
4`	3.94 (dd, 6.9 and 3.4)	85.9	3.92 (dd, 7.6 and 3.8)	85.6		
5`	3.55 (m)/3.66 (m)	61.7	3.62 (m)/3.53 (m)	61.3		
	**Adenine**^b^		**Creatine**^a^		**Hypoxanthine**^b^	
	*δ*_H_ (multi, *J* in Hz)	*δ*_C_	*δ*_H_ (multi, *J* in Hz)	*δ*_C_	*δ*_H_ (multi, *J* in Hz)	*δ*_C_
1	-	-	-	174.5	-	-
2	8.14 (s)	153.7	3.89 (s)	36.8	7.96 (s)	144.6^c^
3	-	-	-	-	-	-
4	-	155.2	-	157.0	-	144.6^c^
5	-	120.1	2.99 (s)	53.7	-	140.3^c^
6	-	160.4			-	155.4
7	-	-			-	-
8	8.12(s)	150.6			8.11 (s)	140.3^c^
9	-	-			-	-

^a^ Data were measured in D_2_O.

^b^ Data were measured in DMSO.

**Table 3:** HRESIMS and optical rotation data of amino acids and nitrogenous compounds.

	HRESIMS		Optical rotation ^c^
	Observed (*m/z*) ^a^	Calculated (*m/z)* ^a^	
Arginine	175.1260	175.1195	+12.3°
Valine	118.0878	118.0868	+5.1°
Leucine	132.1035	132.1024	-12.1°
Isoleucine	132.1035	132.1024	+13.1°
Tyrosine	182.0828	182.0817	-9.8° ^d^
Histidine	156.0784	156.0773	-37.9°
Phenylalanine	166.0875	166.0868	-34.1°
Tryptophan	205.0984	205.0977	-33.3°
Threonine	120.0671	120.0660	-28.1°
Asparagine	133.0625	133.0613	-5.3°
Methionine	150.0598	150.0588	-10.4°
Cysteine	122.0285	122.0275	+116.8°
Glycine	98.5137 ^b^	98.5122 ^b^	-
Glutamic acid	148.0619	148.0609	+11.6°
Creatine	132.0780	132.0773	-
Hypoxanthine	137.0473	137.0463	-
Inosine	269.0888	269.0885	-50.1°
Adenine	136.0636	136.0623	-
Xanthine	153.0426	153.0412	-
Adenosine	268.1053	268.1045	-56.4°

^a^ HRESIMS observed and calculated for [M + H]^+^.

^b^ HRESIMS observed and calculated for [M + Na]^+^.

^c^ Specific rotations were calculated according to the equation [α]^20^_D_ = (100α)/(l × *c*) where l is the path length in decimeters, *c* is the concentration in g/100 mL and data were measured in H_2_O, *c* = 1.

^d^ Data were measured in 1N HCl, *c* = 5.

## Discussion

Behavioral reactivity was confined to the chloroform-insoluble fraction of a Soxhlet skin extract, suggesting constituents of the sea lamprey alarm cue are water-soluble nitrogenous compounds, as confirmed by detailed NMR and MS experiments, consistent with the predictions arising from the sensory physiology of fishes, including lampreys, and prior reported work with individual compounds (e.g. hypoxanthine-3-N-oxide). The most abundant component of the water-soluble fraction was creatine (70%). Other major compounds in the water-soluble fraction were heterocyclic nitrogen compounds and free amino acids, 4.3 and 18.4% of the water-soluble fraction, respectively (Table 4). We have not yet identified compounds from minor fractions yielded from the purification of major compounds from the active water-soluble fraction of the sea lamprey skin extract. This was only because of the very small quantity of these fractions yielded from a large-scale extraction and purification steps. We anticipate to complete the characterization of minor compounds in these fractions since it requires large-scale extractions and availability of animals.

**Table 4:** Concentration of the nitrogenous compounds isolated from the adult migratory sea lamprey skin.

	Concentration	
	μg/g of water-soluble fraction (or % of total water-soluble fraction)	μmol/g of wet skin
Arginine	373 (3.92)	2.14
Valine	48 (0.51)	0.41
Leucine	100 (1.05)	0.76
Isoleucine	34 (0.36)	0.26
Tyrosine	16 (0.17)	0.09
Histidine	36 (0.38)	0.24
Phenylalanine	251 (2.64)	1.52
Tryptophan	104 (1.09)	0.51
Threonine	243 (2.56)	2.01
Asparagine	228 (2.40)	1.73
Methionine	79 (0.83)	0.61
Cysteine	163 (1.71)	1.34
Glycine	47 (0.49)	0.63
Glutamic acid	18 (0.19)	0.12
Creatine	6645 (70.0)	50.7
Hypoxanthine	166 (1.74)	1.22
Inosine	42 (0.44)	0.16
Adenine	34 (0.36)	0.25
Xanthine	70 (0.74)	0.46
Adenosine	94 (0.99)	0.36

Fourteen amino acids were identified from the behaviorally-reactive fraction, each of which have been previously identified from lamprey skin or cartilage excepting tryptophan which is reported in lamprey plasma and fibrinogen [62–66]. Based on our results, adult migratory sea lamprey skin contained about 30% more free amino acids (12.4 μmol/g of wet weight) when compared to reported free amino acids in muscles (anterolateral trunk) (9.39 μmol/g of wet weight) extracted at 4°C in buffer. This is a four-fold increase in essential amino acids (9.82 μmol/g of wet weight) in the skin with respect to the muscles (2.362 μmol/g of wet weight) (Table 4) [64]. Among the essential amino acids, we observed a two-fold increase in arginine, fourteen-fold increase in phenylalanine and seven-fold increase in threonine levels in adult sea lamprey skin. Similarly, we also observed a thirteen- and three-fold decrease in non-essential amino acids such as glutamate and glycine levels in adult sea lamprey skin, respectively. Total distribution of free amino acids in plasma, liver and muscles during various migratory phases of the sea lampreys (ammocoete, parasitic and upstream adult migrant) has been previously reported [64,67]. Total free plasma amino acid and the essential amino acid concentrations were not significantly different among ammocoete, parasitic and upstream migrant sea lampreys [64].

Whether amino acids constitute odorants for lampreys is unresolved. In a single unpublished study, Li [68,69], reports electro-olfactogram (EOG) recordings of adult sea lamprey in response to 42 amino acids, concluding only L- and D-arginine elicit strong olfactory responses. However, it is important to note that the EOG recordings were taken from the main olfactory epithelium, which likely did not record responses from the accessory olfactory organ, where the suspected alarm cue OSNs (the ‘short’ OSN homolog to the teleost crypt cell) are principally distributed [51,52]. It is also notable that the L- and D- forms of aspartic acid, histidine, lysine, tyrosine, tryptophan, as well as L-OH-proline and L-tyrosine, elicited significant olfactory activity (compared to a water control), although at lower intensity than arginine. This finding contrasts significantly with teleost fishes, where the D- form of amino acids typically elicit no olfactory response [70]. More recently, Libants et al. [71] report genetic evidence for 28 trace amine-associated odorant receptor (TAAR) and four V1R odorant receptor genes from sea lamprey, the latter implicated in olfactory detection of amino acids [41–44]. Finally, EOG is a useful screening tool. However, given the exquisite sensitivity of the sea lamprey olfactory apparatus, strong behavioral responses occur at concentrations below the ability of an EOG to measure in the olfactory epithelium. For example, the sea lamprey male sex pheromone component 3-keto-petromyzonol sulfate (3kPZS) elicits significant EOG activity at concentrations above 10^−12^ M [72], whereas behavioral responses in the field may occur at two orders of magnitude lower concentration [73].

To our knowledge, this is the first reporting of the nitrogenous heterocycles hypoxanthine, inosine and xanthine from sea lamprey skin. The presence of hypoxanthine in appreciable quantities in the skin is notable. The nitrogen oxide form of hypoxanthine has been implicated as an alarm cue component in a number of fishes from the superorder Ostariophysi [74], whereas similar molecules lacking the nitrogen oxide functional group, including hypoxanthine, often fail to elicit a behavioral response (e.g., white catfish *Ictalurus catus*, [30]). More recently, Brown and colleagues [32,33] conclude the nitrogen oxide functional group is important to elicit alarm responses in the Ostariophysi, and report examples from more ancestral lineages (*convict cichlids*, *Acrchocentrus nigrofasciatus*, Cichlidae, Acanthopterygii and rainbow trout, *Oncorhynchus mykiss*, Salmonidae, Protacanthopterygii) known to possess alarm cues that do not respond to hypoxanthine-3-N-oxide. Given the Agnatha is ancestral to all gnathostomes, and the lack of conservation of alarm cue chemistry at larger phylogenetic distances as evidenced by a lack of response to heterospecific cues from distant vs. close relatives [33,75,76]. The sea lamprey also exhibits declining behavioral response to alarm cues collected from confamilial species of Petromyzontide, but fail to respond to cues from more ancestral (Atlantic hagfish, Myxine glutinosa, Myxinidae, Agnatha) and derived (white sucker, *Catastomus commersonii*, Catastomidae, Actinopterygii) taxa [40]. Further, the process of Soxhlet extraction at high temperature (70–80°C) for multiple hours does not degrade the reactivity of the extract vs. that derived from grinding and freezing skin [13, Wagner et al., in review]. Thus, it is reasonable to anticipate the findings for fishes of the Teleostei may not be instructive as to the chemical composition of lamprey alarm cues. As with other alarm cues, it is very likely the cue is a mixture that encodes two informational aspects, risk and the identity of the wounded species.

The abundance of creatine in the skin was also notable. Because sea lamprey cease parasitic feeding prior to the onset of migration, energy is mobilized from the tissues to support the expenditures associated with long-distance swimming and reproduction [77] principally in the form of lipid metabolism [78]. However, creatine is a major component in arginine metabolism. The mechanism by which the conversion of adenosine triphosphate (ATP) to adenosine diphosphate (ADP) results in the transfer of one of the phosphate groups to creatine to form creatine phosphate [67]. Hence, accumulation of large amount of creatine, adenine and adenosine in the skin may support the metabolism of internal energy stores during the high-energy demand associated with the reproductive migration.

The sea lamprey alarm cue induces context-specific expression of predator avoidance behaviors in nature when applied to natural streams that may be utilized to achieve novel control measures. For example, when the odor is confined to a portion of the channel, migrants will swim on the opposite side [24]. However, if the river’s discharge is fully activated with the odor, the response becomes responsive to circumstance. Luhring and co-authors [23] demonstrated that individuals entering the river from a lake simply switch anti-predator tactics from spatial avoidance to exposure minimization by increasing swimming speed, whereas those already in the river delayed upstream movement (i.e. remained hidden before moving upstream). Together, these findings suggest development of control tactics based on the manipulation of movement paths, but not blockage, will prove viable. Approaches include trapping at dams and in open river channels, guiding migrating sea lamprey into streams targeted for future lampricide treatments, and the creation of selective fish passage devices that utilize the repellent to block sea lamprey from entering a fishway [11,12,25,79]. However, utilization of the alarm cue repellent, or any semiochemical discovered in sea lamprey, is subject to regulation by the U.S. EPA and Health-Canada. Specifically, the use of an odorant or mixture of odorants to manipulate the behavior of a pest species is classified as a biopesticide under the Federal Insecticide, Fungicide, and Rodenticide Act (FIFRA, USA). Thus, to meet the legal requirements for use as a biopesticide, the chemical structure of the active ingredients must be known and its application practices (concentration, duration) specified. As a practical matter, discovery of the chemical structure(s) of the components of the alarm cue is necessary to produce it in sufficient quantities for use throughout the basin.

In conclusion, we have isolated and chemically characterized the major nitrogenous compounds in the water-soluble fraction of a skin extract that contains the behavioral activity of an alarm cue from sea lamprey. The constituents identified are molecules common in nature and animal tissue. Further behavioral testing is underway to ascertain which of these induce avoidance behavior in sea lamprey, and to ensure that minor constituents of the extract awaiting characterization are not the principal alarm cue components. Among the isolates, creatine was the most abundant component in the water-soluble fraction. Based on our findings migratory sea lamprey skin contained 30% more free amino acids when compared to free amino acids reported in its muscles. Report of nitrogenous heterocycles hypoxanthine, inosine and xanthine from the sea lamprey skin is also for the first time.

## Supporting information

S1 File CombiFlash and HPLC profiles of the extract and its fractions. (PDF)

S2 File Nuclear Magnetic Resonance (NMR) and High Resolution Mass Spectrometry (HRMS) data for creatine, arginine, valine, leucine, isoleucine, and tyrosine. (PDF)

S3 File Nuclear Magnetic Resonance (NMR) and High Resolution Mass Spectrometry (HRMS) data for hypoxanthine, inosine, adenine, xanthine histidine and glutamic acid. (PDF)

S4 File Nuclear Magnetic Resonance (NMR) and High Resolution Mass Spectrometry (HRMS) data for phenylalanine, tryptophan, threonine, asparagine, methionine and cysteine. (PDF)

S5 File Nuclear Magnetic Resonance (NMR) and High Resolution Mass Spectrometry (HRMS) data for adenosine, and glycine. (PDF)

## Abbreviations

DMSO: Dimethyl sulfoxide
GLFC: Great Lakes Fisheries Commission
EOG: Electro-olfactogram
HPLC: High pressure liquid chromatography
HR-ESITOFMS: High resolution electron spray ionization time of flight mass spectroscopy
MPLC: Medium pressure liquid chromatography
MSU: Michigan State University
NMR: Nuclear magnetic resonance
OSNs: Olfactory sensory neurons
RO water: Reverse osmosis water
TFM: 3-trifluoromethyl-4-nitrophenol
