# Supplementary material for: Nitrogenous compounds characterized in the deterrent skin extract of migratory adult sea lamprey from the Great Lakes region
Source: PLoS One. 2019 May 23;14(5):e0217417. doi: 10.1371/journal.pone.0217417 (PMC6532902; doi:10.1371/journal.pone.0217417)
Supplement: S1 File — (PDF) [file pone.0217417.s001.pdf]

Nitrogenous Compounds Characterized in the deterrent Skin extract of Migratory Adult Sea Lamprey from the Great Lakes Region

Amila A. Dissanayake,[1] C. Michael Wagner,[2] Muraleedharan G. Nair,[1*]

[1] Department of Horticulture, Michigan State University, East Lansing, Michigan, United States of America

[2] Department of Fisheries and Wildlife, Michigan State University, East Lansing, Michigan, United States of America

Supporting Information

Figure A. CombiFlash Rf chromatogram of the water-soluble fraction

Figure B. CombiFlash Rf chromatogram of the fraction **B**

Figure C. CombiFlash Rf chromatogram of the fraction **C**

Figure D. HPLC profiles of the fraction **A**

Figure E. HPLC profiles of the fraction **B-1**

Figure F. HPLC profiles of the fraction **B-2**

Figure G. HPLC profiles of the fraction **B-3**

Figure H. HPLC profiles of fraction **C-1**

Figure I. HPLC profiles of fraction **C-2**

Figure J. HPLC profiles of fraction **C-3**

Figure K. HPLC profiles of fraction **C-4**

Figure L. HPLC profiles of fraction **C-4(a)**

Figure M. HPLC profiles of fraction **C-4(b)**

Figure N. HPLC profiles of fraction **C-4(c)**

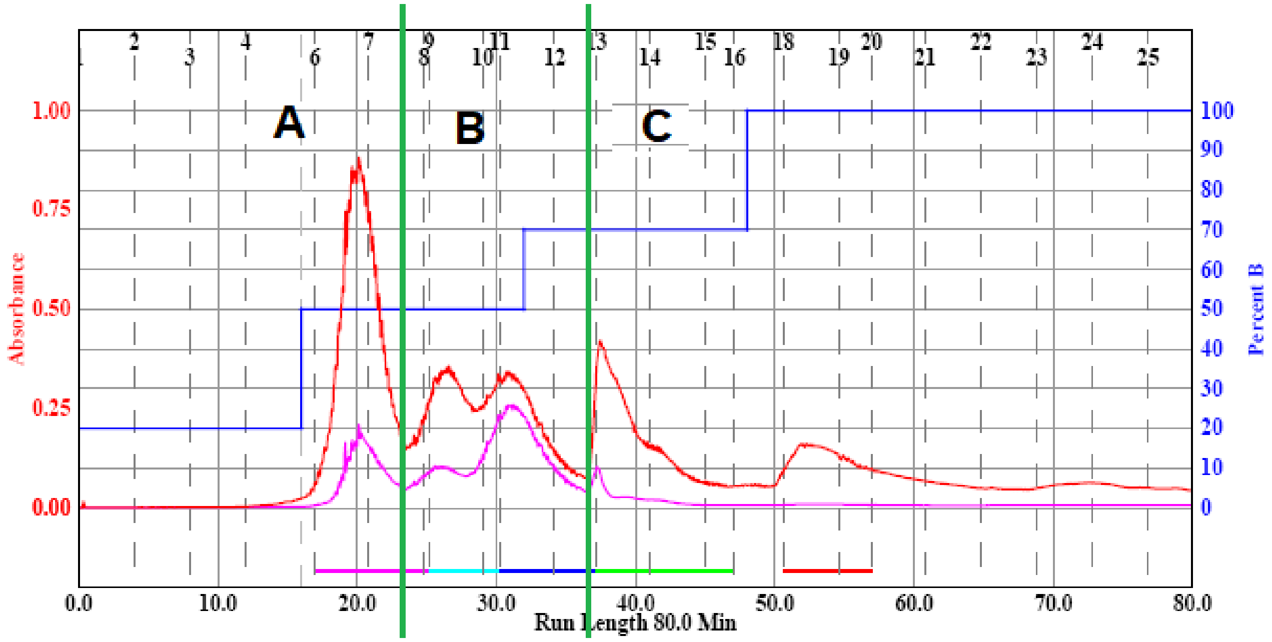

Figure A. CombiFlash chromatogram of the water-soluble fraction. Peaks monitored at 210 nm (red) and 254 nm (purple).

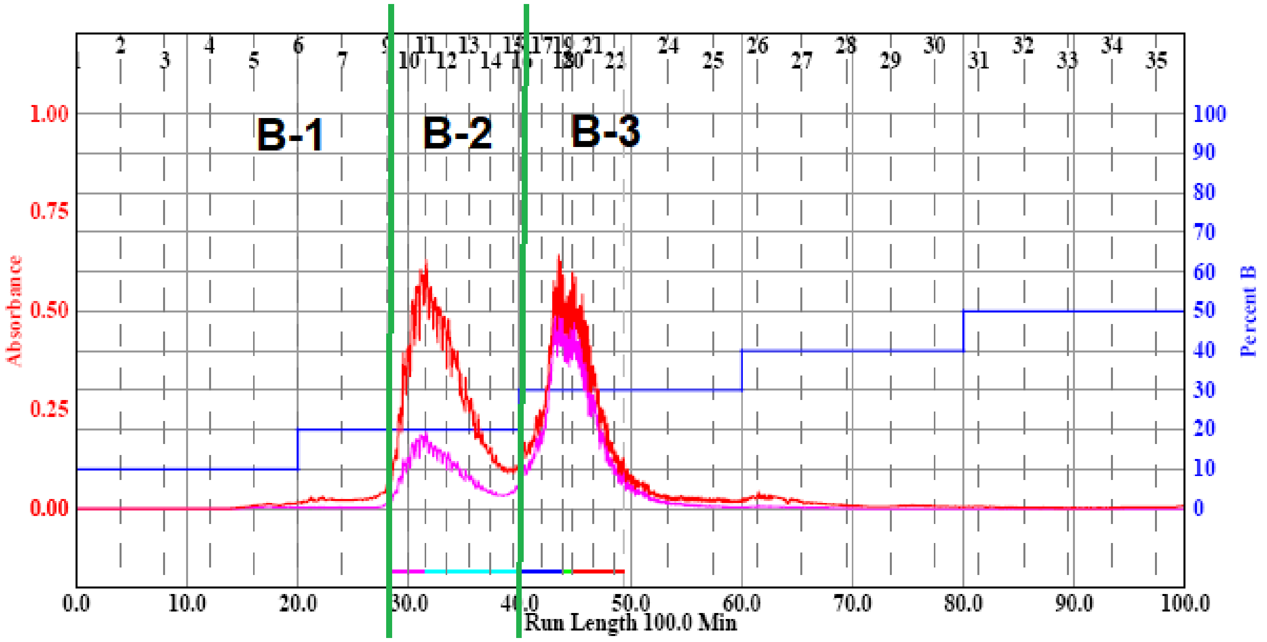

Figure B. CombiFlash chromatogram of the fraction **B**. Peaks monitored at 210 nm (red) and 254 nm (purple).

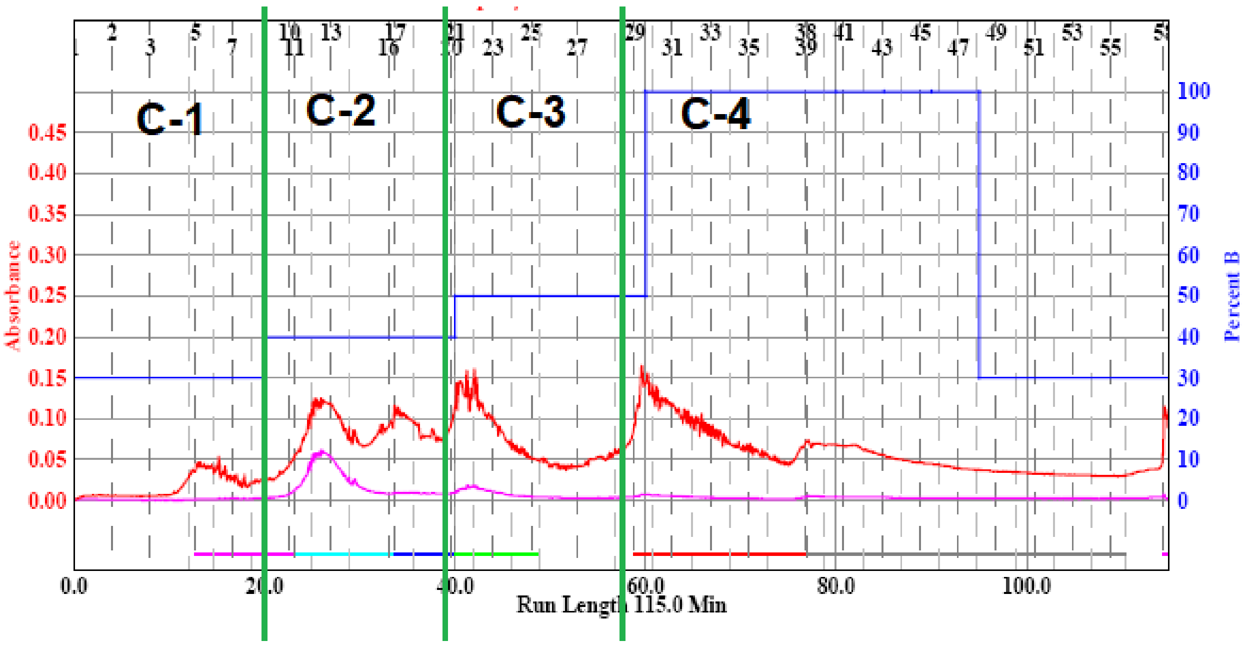

Figure C. CombiFlash chromatogram of the fraction **C**. Peaks monitored at 210 nm (red) and 254 nm (purple).

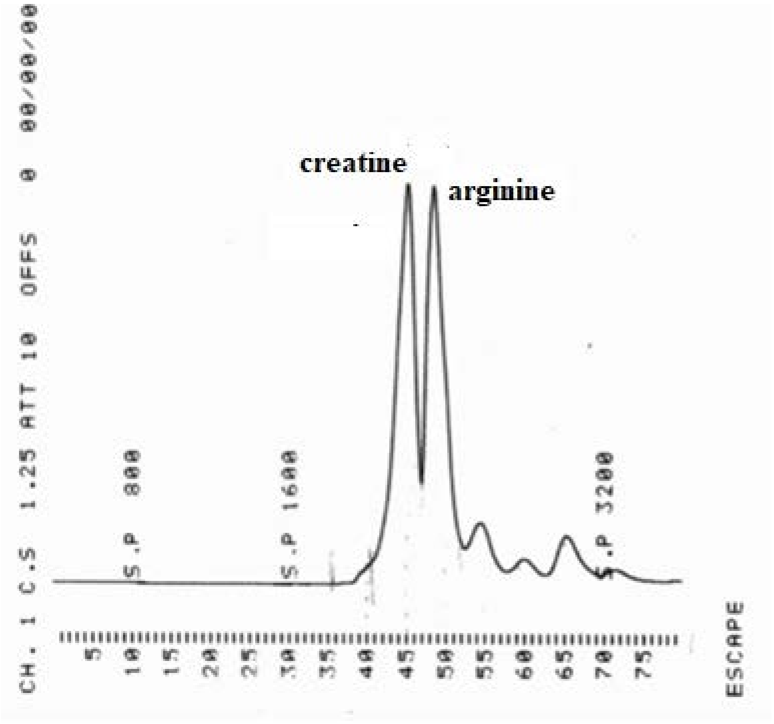

Figure D. HPLC profiles of the fraction **A**, Solvent system; water: methanol, 95:5 @ 26°C on a C-8 preparative HPLC column (Xtera, Waters Corp.). Flow rate 3 mL/min and peaks monitored at 210 nm.

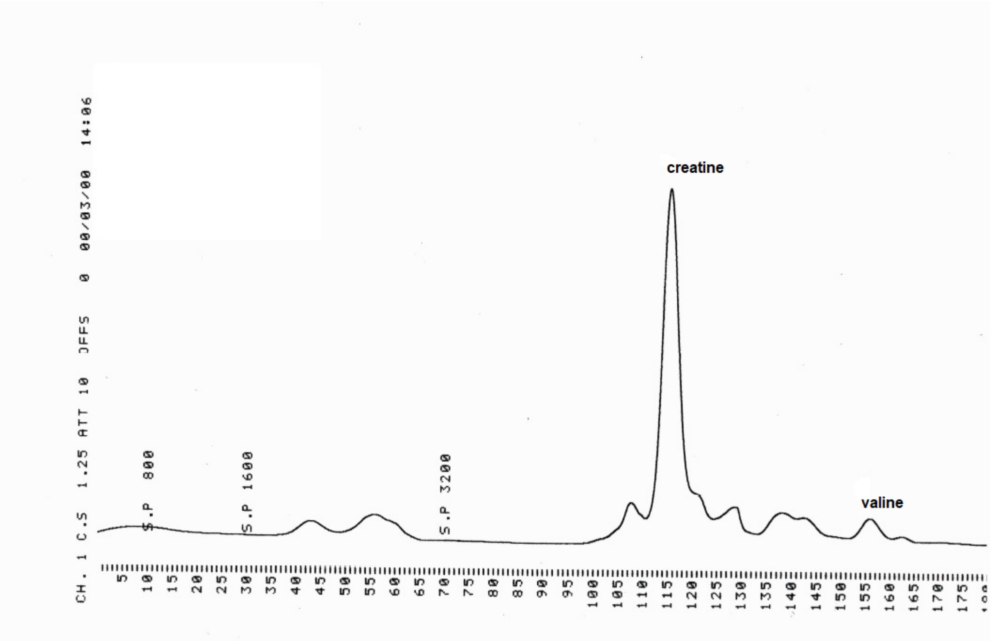

Figure E. HPLC profiles of the fraction **B-1**, Solvent system; water: methanol, 95:5 @ 26°C on a C-8 preparative HPLC column (Xtera, Waters Corp.). Flow rate 1.5 mL/min and peaks monitored at 210 nm.

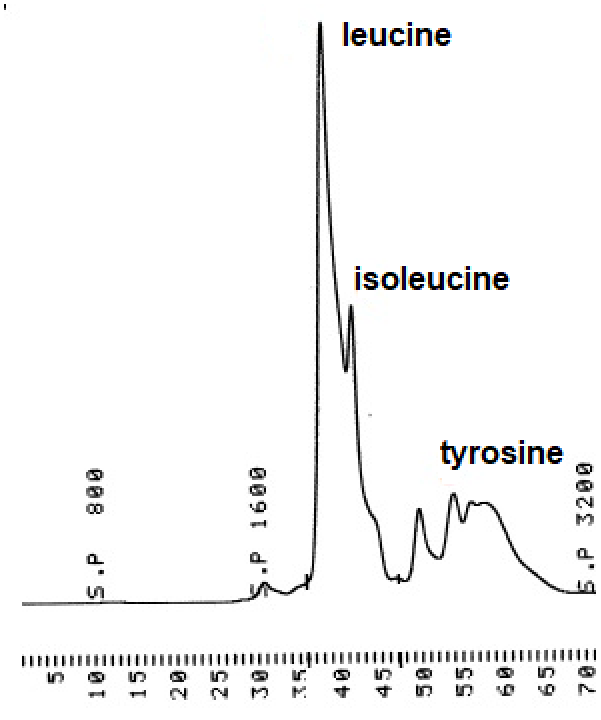

Figure F. HPLC profiles of fraction **B-2**, Solvent system; water: methanol, 95:5 @ 26°C on a C-8 preparative HPLC column (Xtera, Waters Corp.). Flow rate 4.0 mL/min and peaks monitored at 210 nm.

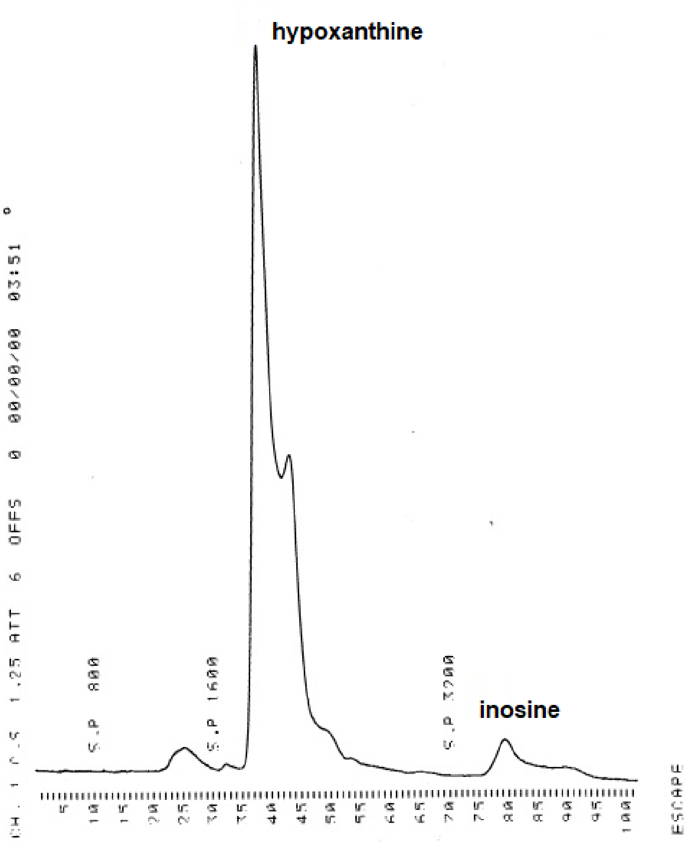

Figure G. HPLC profiles of fraction **B-3**, Solvent system; water: methanol, 98:3 @ 26°C on a C-8 preparative HPLC column (Xtera, Waters Corp.). Flow rate 3.0 mL/min and peaks monitored at 210 nm.

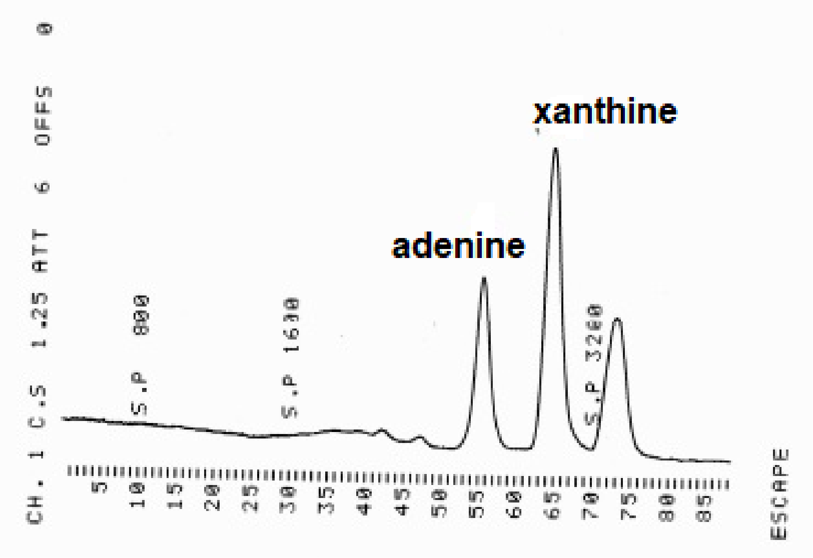

Figure H. HPLC profiles of fraction **C-1**, Solvent system; water: methanol, 95:5 @ 26°C on a C-8 preparative HPLC column (Xtera, Waters Corp.). Flow rate 4.0 mL/min and peaks monitored at 210 nm.

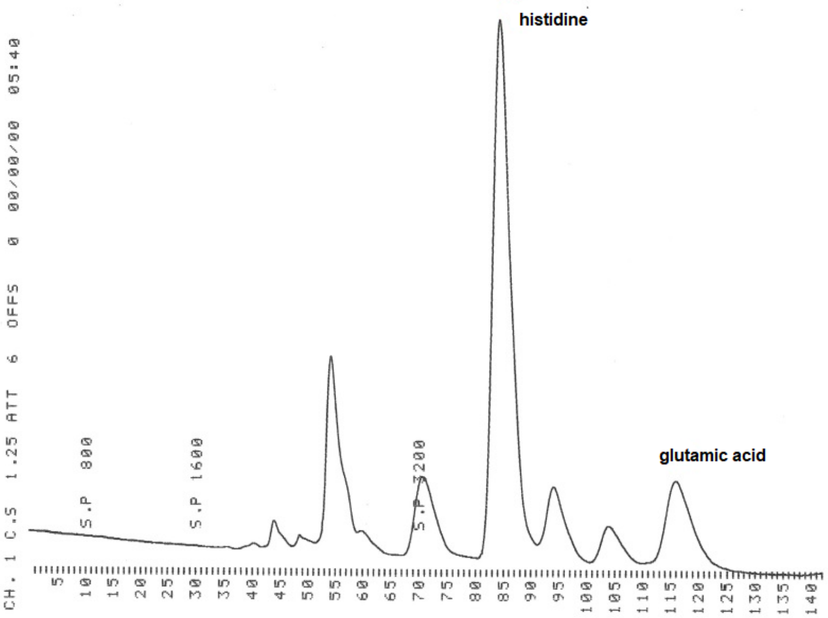

Figure I. HPLC profiles of fraction **C-2**, Solvent system; water: methanol, 95:5 @ 26°C on a C-8 preparative HPLC column (Xtera, Waters Corp.). Flow rate 4.0 mL/min and peaks monitored at 210 nm.

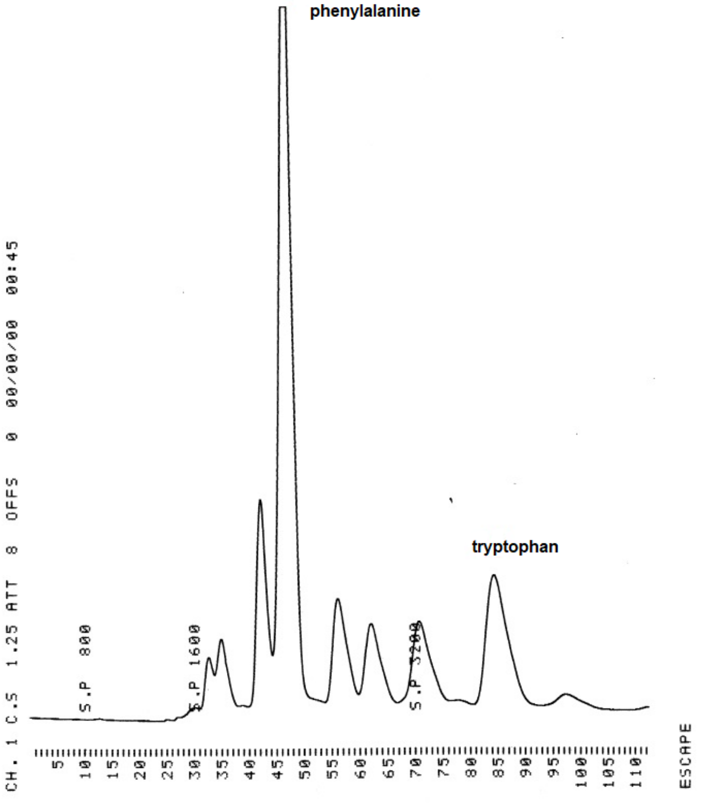

Figure J. HPLC profiles of fraction **C-3**, Solvent system; water: methanol, 95:5 @ 26°C on a C-8 preparative HPLC column (Xtera, Waters Corp.). Flow rate 4.0 mL/min and peaks monitored at 210 nm.

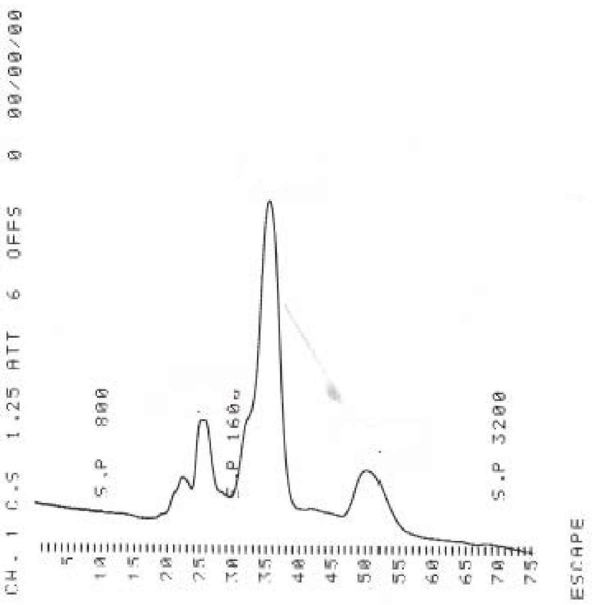

Figure K. HPLC profiles of fraction **C-4**, Solvent system; water: methanol, 80:20 @ 26°C on a C-8 preparative HPLC column (Xtera, Waters Corp.). Flow rate 3.0 mL/min and peaks monitored at 210 nm.

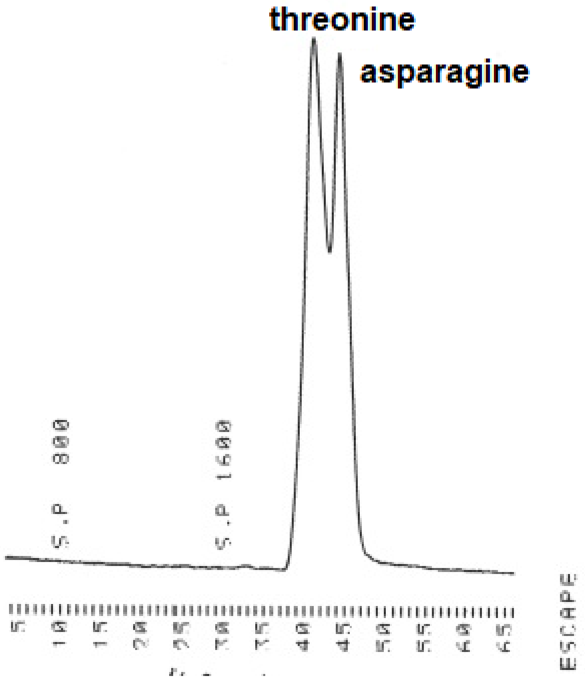

Figure L. HPLC profiles of fraction **C-4(a)**, Solvent system; water: methanol, 90:10 @ 26°C on a C-8 preparative HPLC column (Xtera, Waters Corp.). Flow rate 4.0 mL/min and peaks monitored at 210 nm.

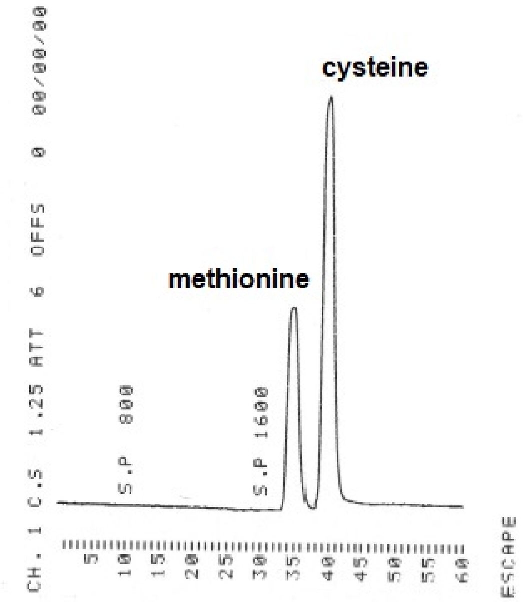

Figure M. HPLC profiles of fraction **C-4(b)**, Solvent system; water: methanol, 90:10 @ 26°C on a C-8 preparative HPLC column (Xtera, Waters Corp.). Flow rate 4.0 mL/min and peaks monitored at 210 nm.

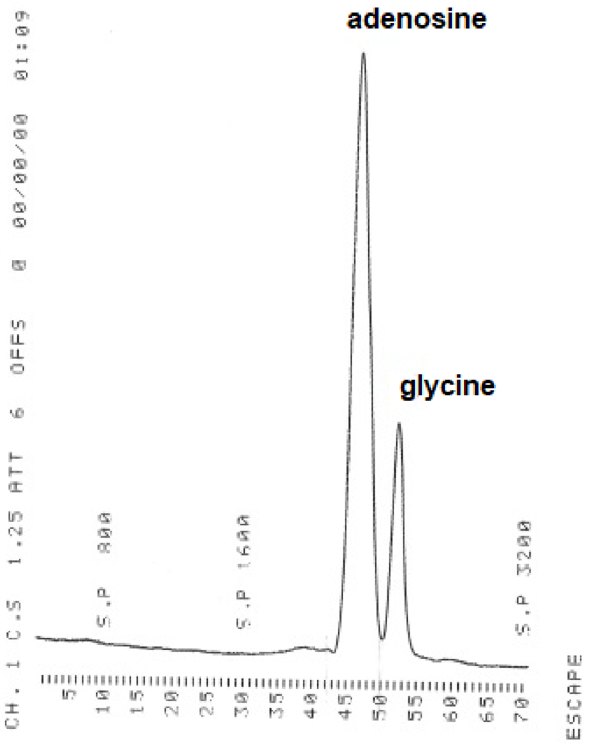

Figure N. HPLC profiles of fraction **C-4(c)**, Solvent system; water: methanol, 90:10 @ 26°C on a C-8 preparative HPLC column (Xtera, Waters Corp.). Flow rate 3.0 mL/min and peaks monitored at 210 nm.
