# Supplementary material for: Nitrogenous compounds characterized in the deterrent skin extract of migratory adult sea lamprey from the Great Lakes region
Source: PLoS One. 2019 May 23;14(5):e0217417. doi: 10.1371/journal.pone.0217417 (PMC6532902; doi:10.1371/journal.pone.0217417)
Supplement: S2 File — (PDF) [file pone.0217417.s002.pdf]

Nitrogenous Compounds Characterized in the deterrent Skin extract of Migratory Adult Sea Lamprey from the Great Lakes Region

Amila A. Dissanayake,[1] C. Michael Wagner,[2] Muraleedharan G. Nair,[1*]

[1] Department of Horticulture, Michigan State University, East Lansing, Michigan, United States of America

[2] Department of Fisheries and Wildlife, Michigan State University, East Lansing, Michigan, United States of America

Supporting Information

Figure A. ^{1}H NMR spectrum of creatine in D_2O
Figure B. ^{13}C NMR spectrum of creatine in D_2O
Figure C. DEPT spectrum of creatine in D_2O
Figure D. HRMS spectrum of creatine (positive ion mode)
Figure E. ^{1}H NMR spectrum of arginine in D_2O
Figure F. ^{13}C NMR spectrum of arginine in D_2O
Figure G. DEPT spectrum of arginine in D_2O
Figure H. HRMS spectrum of arginine (positive ion mode)
Figure I. ^{1}H NMR spectrum of valine in D_2O
Figure J. ^{13}C NMR spectrum of valine in D_2O
Figure K. DEPT spectrum of valine in D_2O
Figure L. HRMS spectrum of valine (positive ion mode)
Figure M. ^{1}H NMR spectrum of leucine in D_2O
Figure N. ^{13}C NMR spectrum of leucine in D_2O
Figure O. DEPT spectrum of leucine in D_2O
Figure P. HRMS spectrum of leucine (positive ion mode)
Figure Q. ^{1}H NMR spectrum of isoleucine in D_2O
Figure R. ^{13}C NMR spectrum of isoleucine in D_2O
Figure S. DEPT spectrum of isoleucine in D_2O
Figure T. HRMS spectrum of isoleucine (positive ion mode)
Figure U. ^{1}H NMR spectrum of tyrosine in D_2O
Figure V. ^{13}C NMR spectrum of tyrosine in D_2O
Figure W. DEPT spectrum of tyrosine in D_2O
Figure X. HRMS spectrum of tyrosine (positive ion mode)

Figure A

Sample Name:
AD_120_37A
Data Collected on:
ormuzd-vnmrs500
Archive directory:
/home/walkup/vnmrsys/data/amilad
Sample directory:
AD_120_37A_20170418_01
FidFile: AD_120_37A_CARBON_01

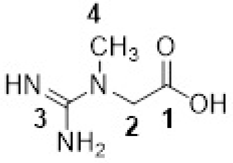

INDEX	FREQUENCY	PPM	HEIGHT
1	21929.5	174.530	22.3
2	19727.9	157.008	11.1
3	6751.5	[illegible]	95.2
4	[illegible]	[illegible]	[illegible]

Agilent Technologies

Pulse Sequence: CARBON (s2pul)
Solvent: d2o
Data collected on: Apr 18 2017

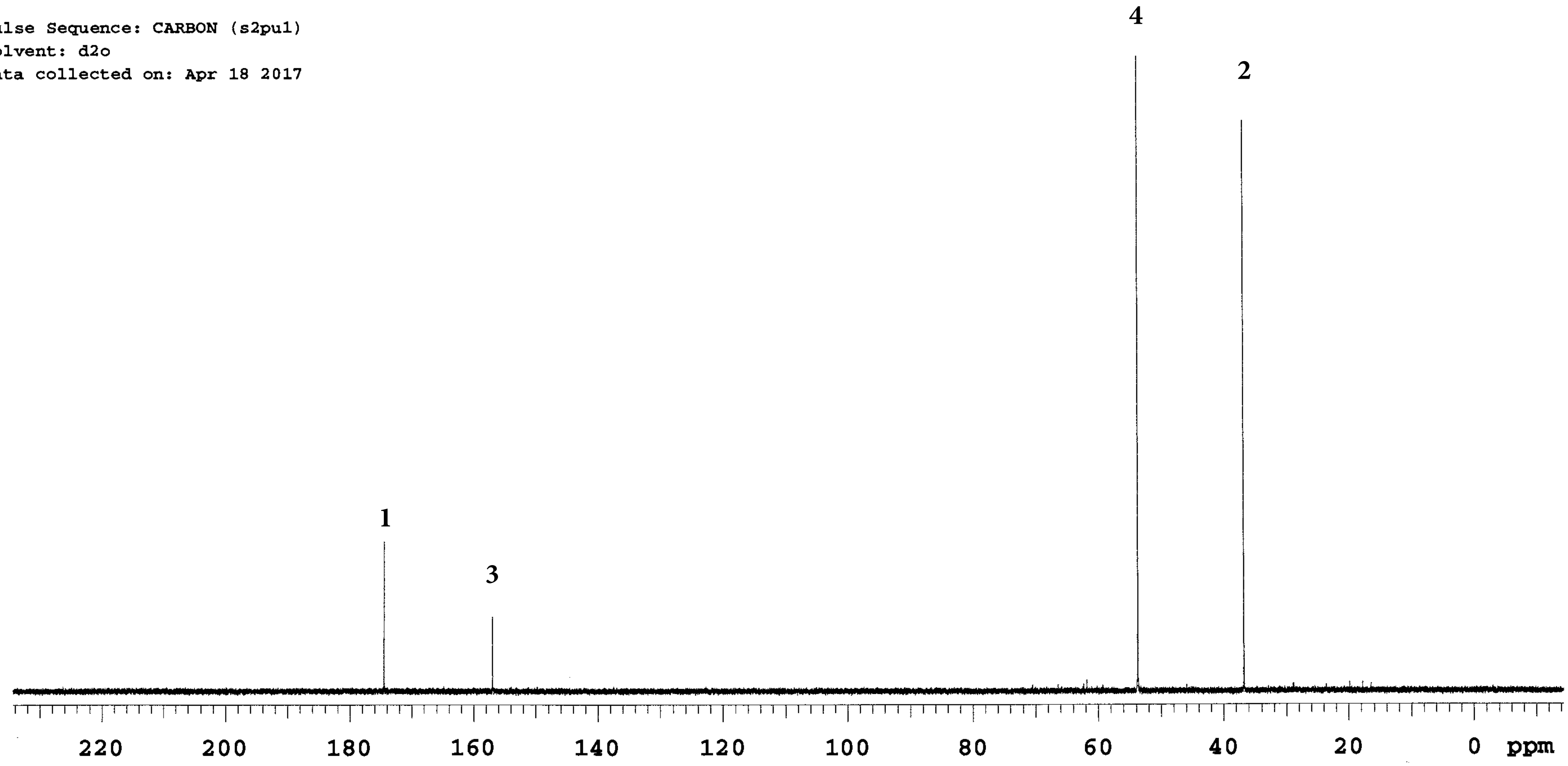

Figure B

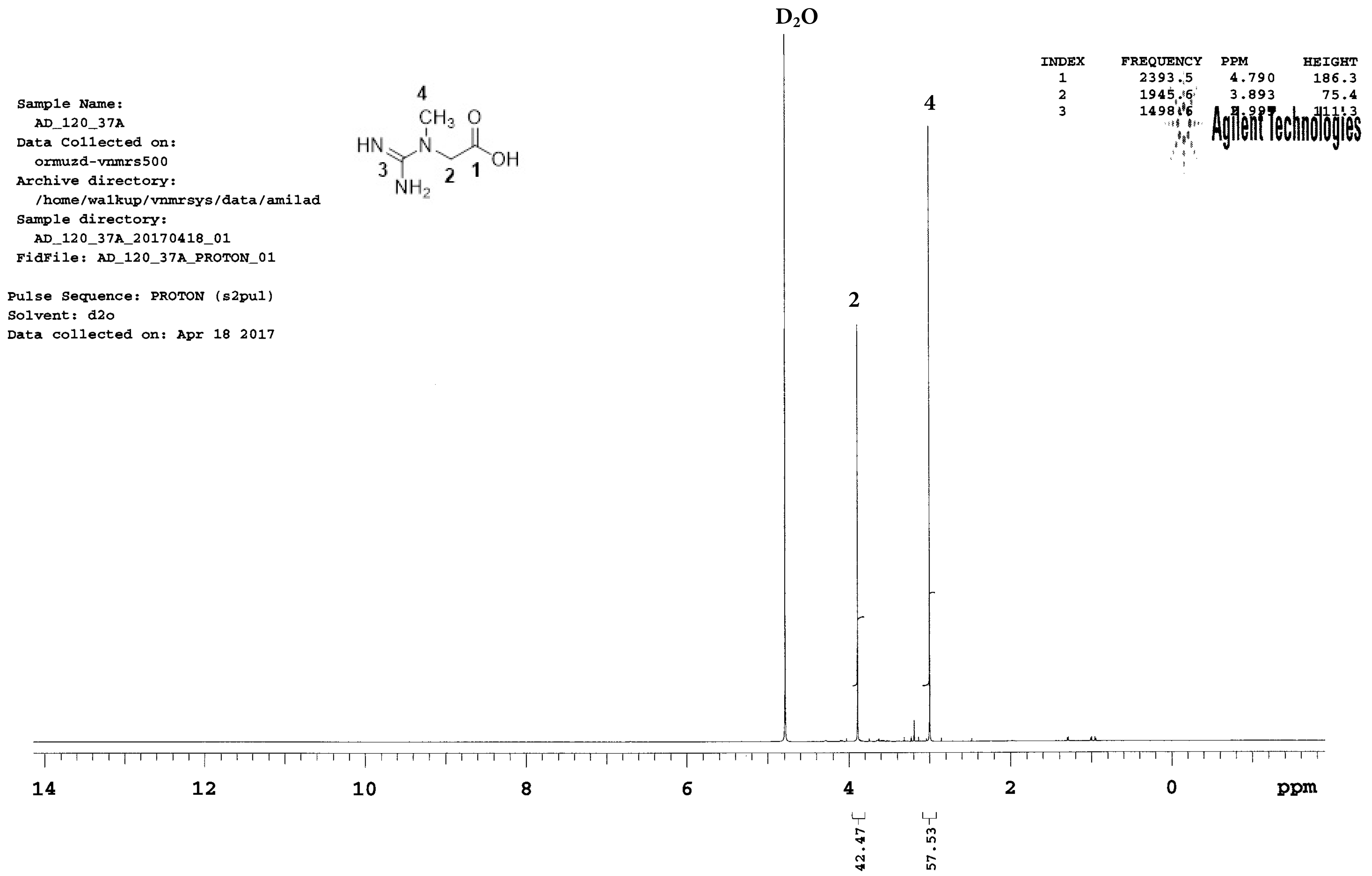
D2O
Sample Name:
AD_120_37A
Data Collected on:
ormuzd-vnmrs500
Archive directory:
/home/walkup/vnmrsys/data/amilad
Sample directory:
AD_120_37A_20170418_01
FidFile: AD_120_37A_PROTON_01
Pulse Sequence: PROTON (s2pul)
Solvent: d2o
Data collected on: Apr 18 2017
INDEX FREQUENCY PPM HEIGHT
1 2393.5 4.790 186.3
2 1945.6 3.893 75.4
Agilent Technologies
HN
NH2
CH3
N
O
OH
1
2
3
4
14
12
10
8
6
4
2
0
ppm
42.47
57.53

Figure C

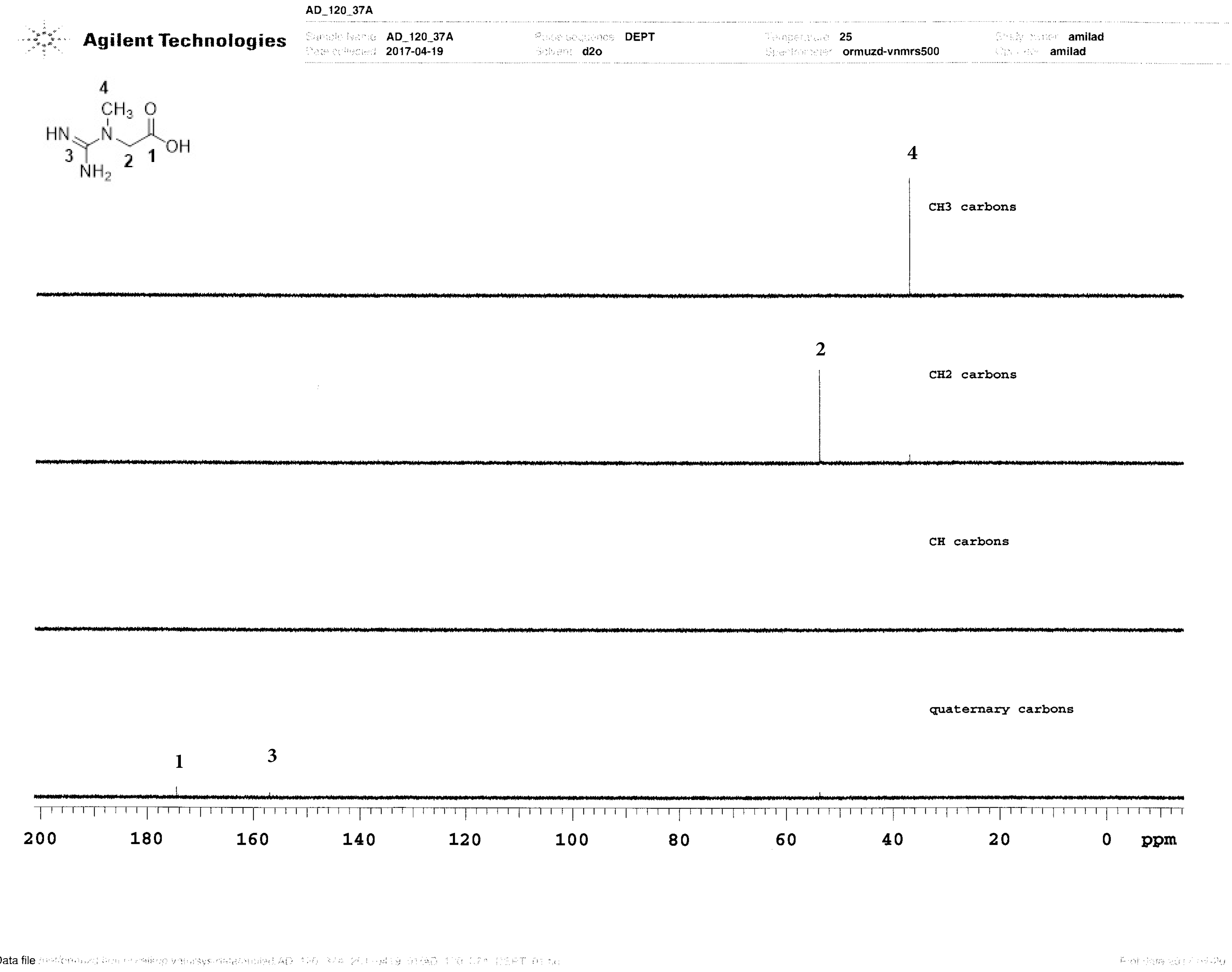
AD_120_37A
Agilent Technologies
AD_120_37A
DEPT
25
amilad
2017-04-19
d2o
ormuzd-vnmrs500
amilad
4
CH_3
O
HN
N
OH
3
2
1
NH_2
4
CH3 carbons
2
CH2 carbons
CH carbons
quaternary carbons
1
3
200
180
160
140
120
100
80
60
40
20
0
ppm
Data file

Figure D

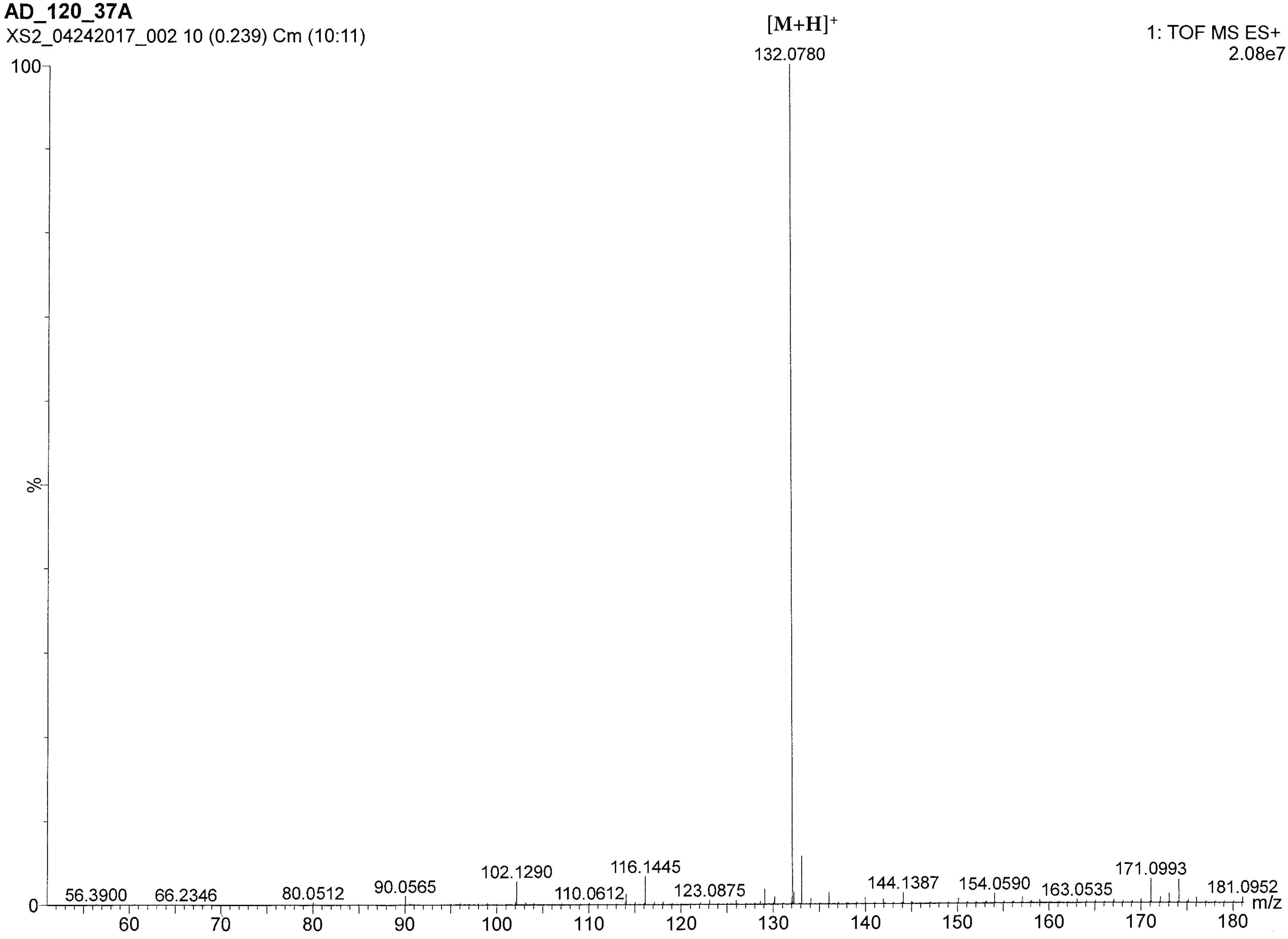
AD_120_37A
XS2_04242017_002 10 (0.239) Cm (10:11)
1: TOF MS ES+
2.08e7
[M+H]+
132.0780
100
%
0
56.3900
66.2346
80.0512
90.0565
102.1290
110.0612
116.1445
123.0875
144.1387
154.0590
163.0535
171.0993
181.0952
60
70
80
90
100
110
120
130
140
150
160
170
180
m/z

Figure E

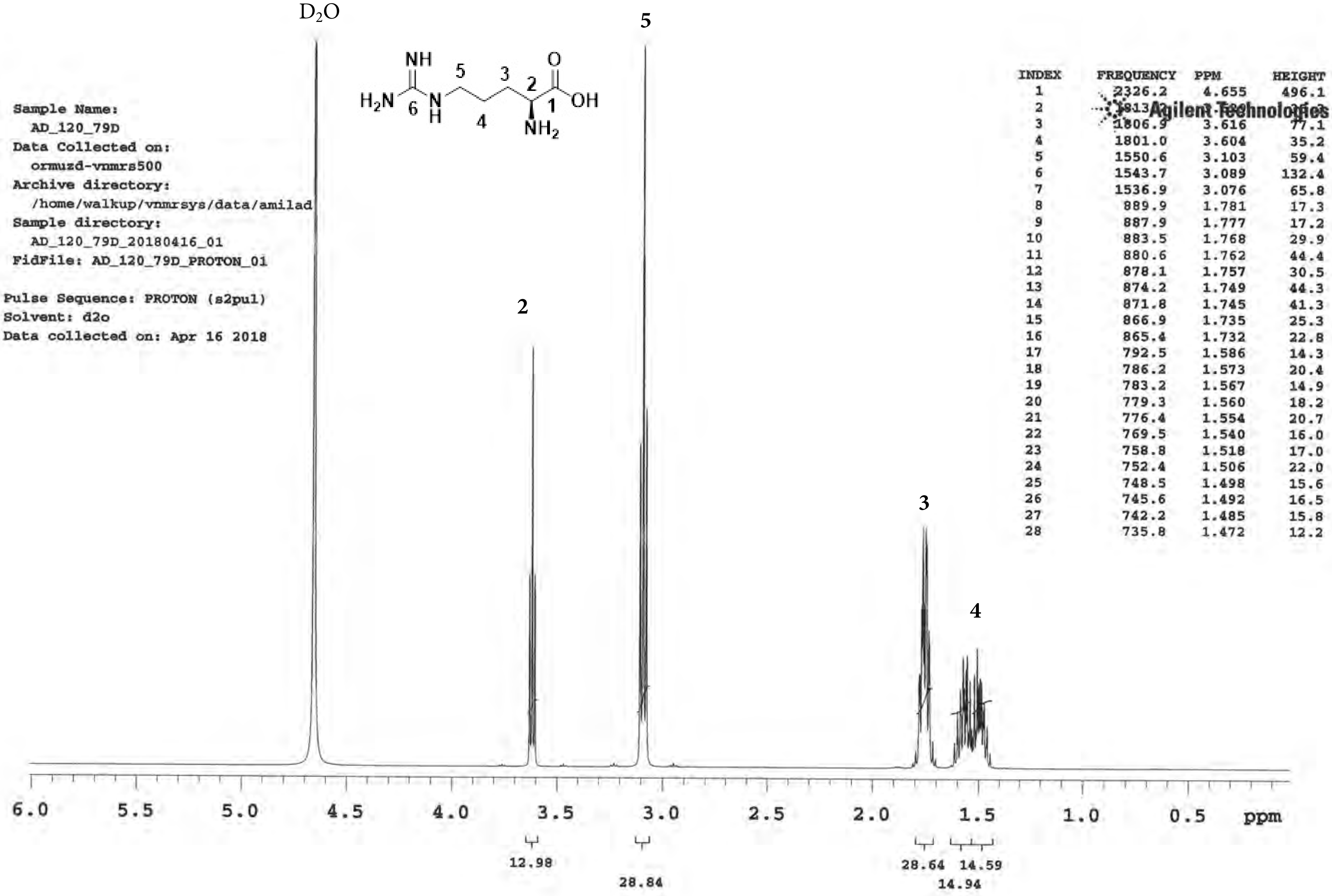

INDEX	FREQUENCY	PPM	HEIGHT
1	2326.2	4.655	496.1
2	[illegible]	[illegible]	[illegible]
3	1806.9	3.616	77.1
4	1801.0	3.604	35.2
5	1550.6	3.103	59.4
6	1543.7	3.089	132.4
7	1536.9	3.076	65.8
8	889.9	1.781	17.3
9	887.9	1.777	17.2
10	883.5	1.768	29.9
11	880.6	1.762	44.4
12	878.1	1.757	30.5
13	874.2	1.749	44.3
14	871.8	1.745	41.3
15	866.9	1.735	25.3
16	865.4	1.732	22.8
17	792.5	1.586	14.3
18	786.2	1.573	20.4
19	783.2	1.567	14.9
20	779.3	1.560	18.2
21	776.4	1.554	20.7
22	769.5	1.540	16.0
23	758.8	1.518	17.0
24	752.4	1.506	22.0
25	748.5	1.498	15.6
26	745.6	1.492	16.5
27	742.2	1.485	15.8
28	735.8	1.472	12.2

Figure F

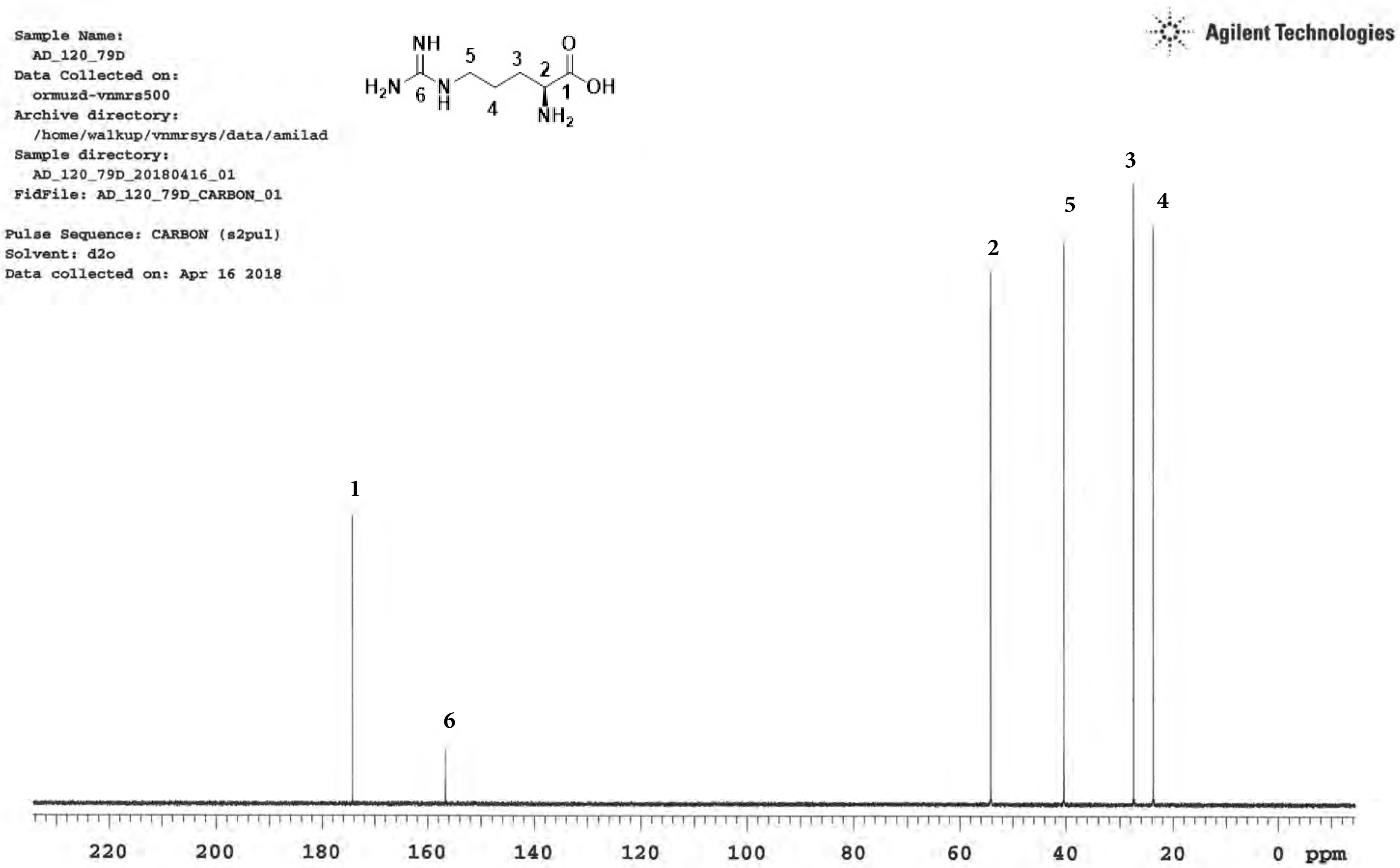
Sample Name:
AD_120_79D
Data Collected on:
ormuzd-vnmrs500
Archive directory:
/home/walkup/vnmrsys/data/amilad
Sample directory:
AD_120_79D_20180416_01
FidFile: AD_120_79D_CARBON_01
Pulse Sequence: CARBON (s2pul)
Solvent: d2o
Data collected on: Apr 16 2018
Agilent Technologies
1
6
2
5
3
4
220
200
180
160
140
120
100
80
60
40
20
0
ppm

Figure G

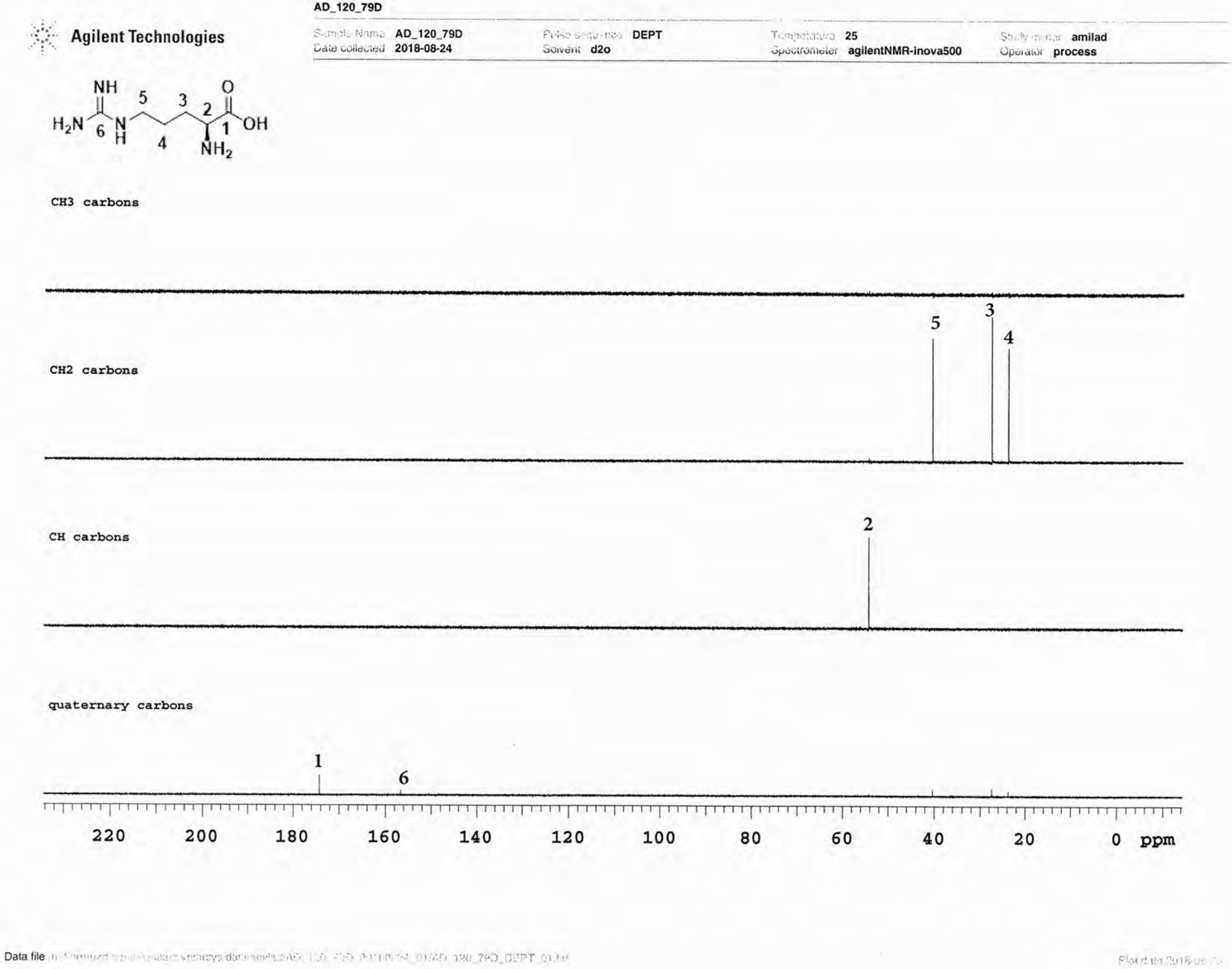
AD_120_79D
Agilent Technologies
AD_120_79D
2018-08-24
DEPT
d2o
25
agilentNMR-inova500
amilad
process
CH3 carbons
CH2 carbons
CH carbons
quaternary carbons
220
200
180
160
140
120
100
80
60
40
20
0
ppm
Data file

Figure H

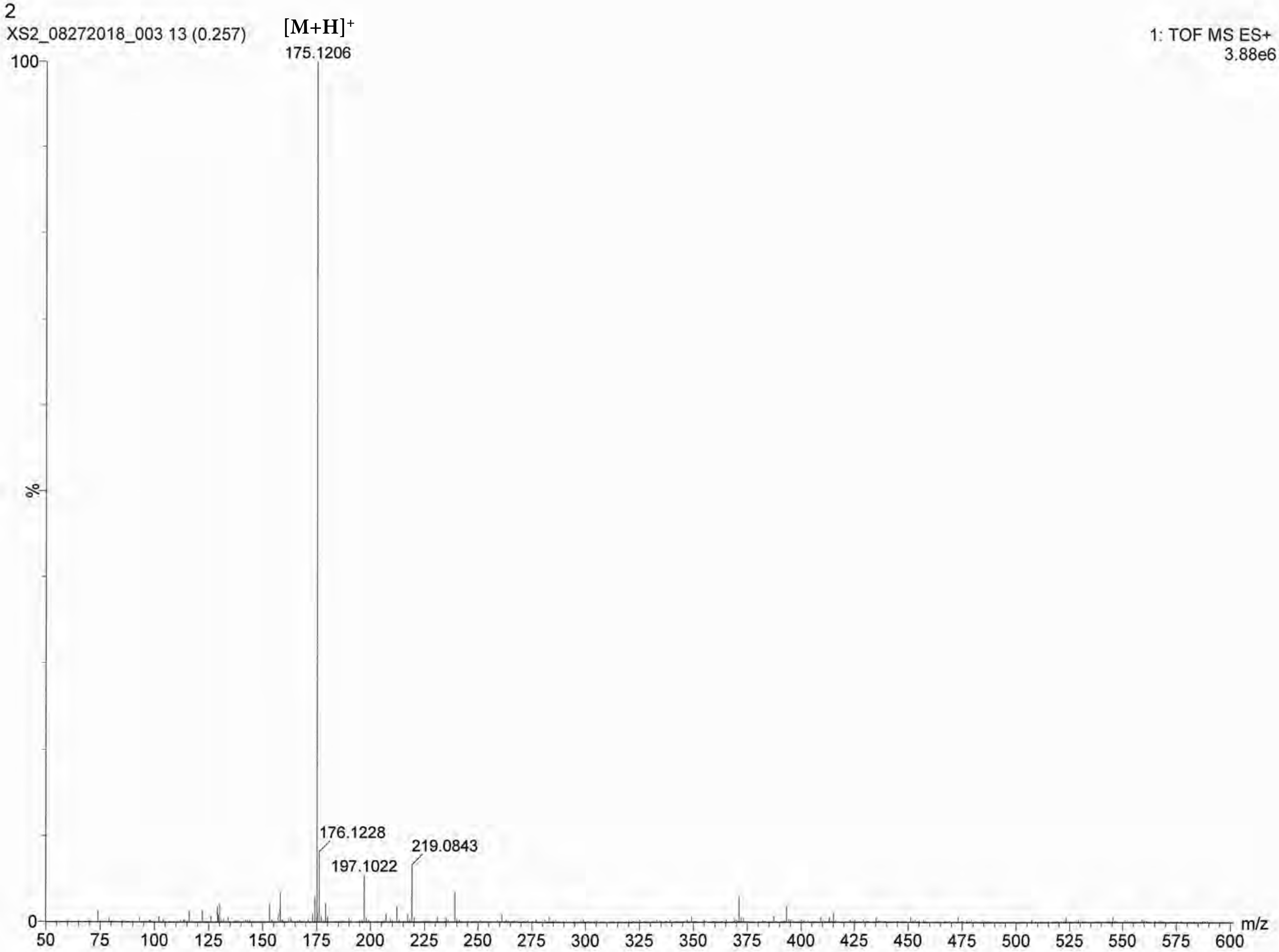

2
XS2_08272018_003 13 (0.257)
[M+H]+
175.1206
1: TOF MS ES+
3.88e6
100
%
0
176.1228
219.0843
197.1022
m/z
50
75
100
125
150
175
200
225
250
275
300
325
350
375
400
425
450
475
500
525
550
575
600

Figure I

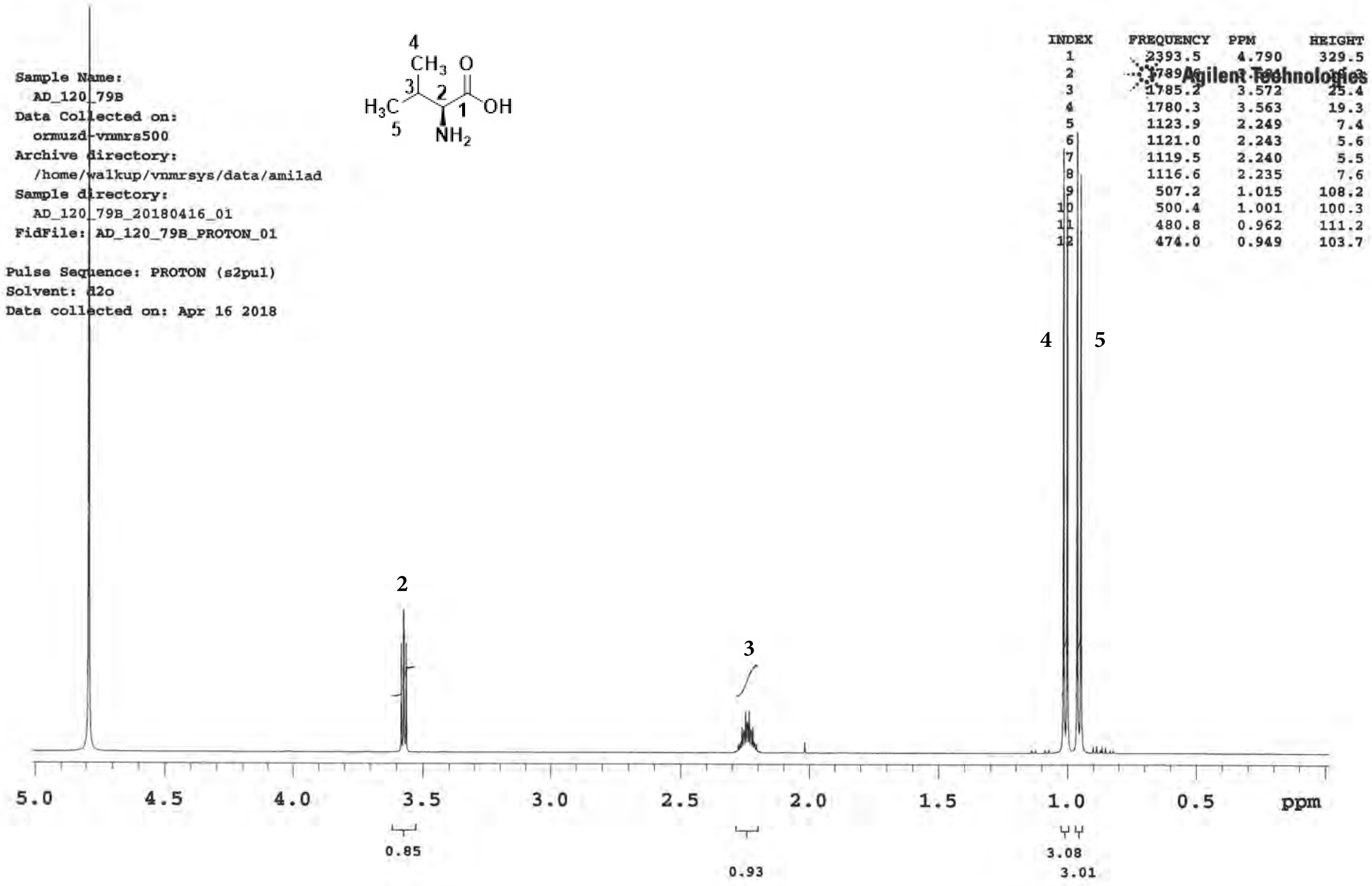

Sample Name:
AD_120_79B
Data Collected on:
ormuzd-vnmrs500
Archive directory:
/home/walkup/vnmrsys/data/amilad
Sample directory:
AD_120_79B_20180416_01
FidFile: AD_120_79B_PROTON_01
Pulse Sequence: PROTON (s2pul)
Solvent: d2o
Data collected on: Apr 16 2018
4
CH_3
O
3
2
H_3C
1
OH
5
NH_2
INDEX FREQUENCY PPM HEIGHT
1 2393.5 4.790 329.5
2
Agilent Technologies
3 1785.2 3.572 25.4
4 1780.3 3.563 19.3
5 1123.9 2.249 7.4
6 1121.0 2.243 5.6
7 1119.5 2.240 5.5
8 1116.6 2.235 7.6
9 507.2 1.015 108.2
10 500.4 1.001 100.3
11 480.8 0.962 111.2
12 474.0 0.949 103.7
4
5
2
3
5.0
4.5
4.0
3.5
3.0
2.5
2.0
1.5
1.0
0.5
ppm
0.85
0.93
3.08
3.01

Figure J

Sample Name:
AD_120_79B
Data Collected on:
ormuzd-vnmrs500
Archive directory:
/home/walkup/vnmrsys/data/amilad
Sample directory:
AD_120_79B_20180417_01
FidFile: AD_120_79B_CARBON_01

Pulse Sequence: CARBON (s2pul)
Solvent: d2o
Data collected on: Apr 18 2018

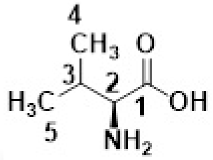

INDEX	FREQUENCY	PPM	HEIGHT
1	21883.7	174.166	36.2
2	[illegible]	[illegible]	[illegible]
3	3639.0	28.961	85.4
4	2239.9	17.827	106.3
5	2072.1	16.491	85.2

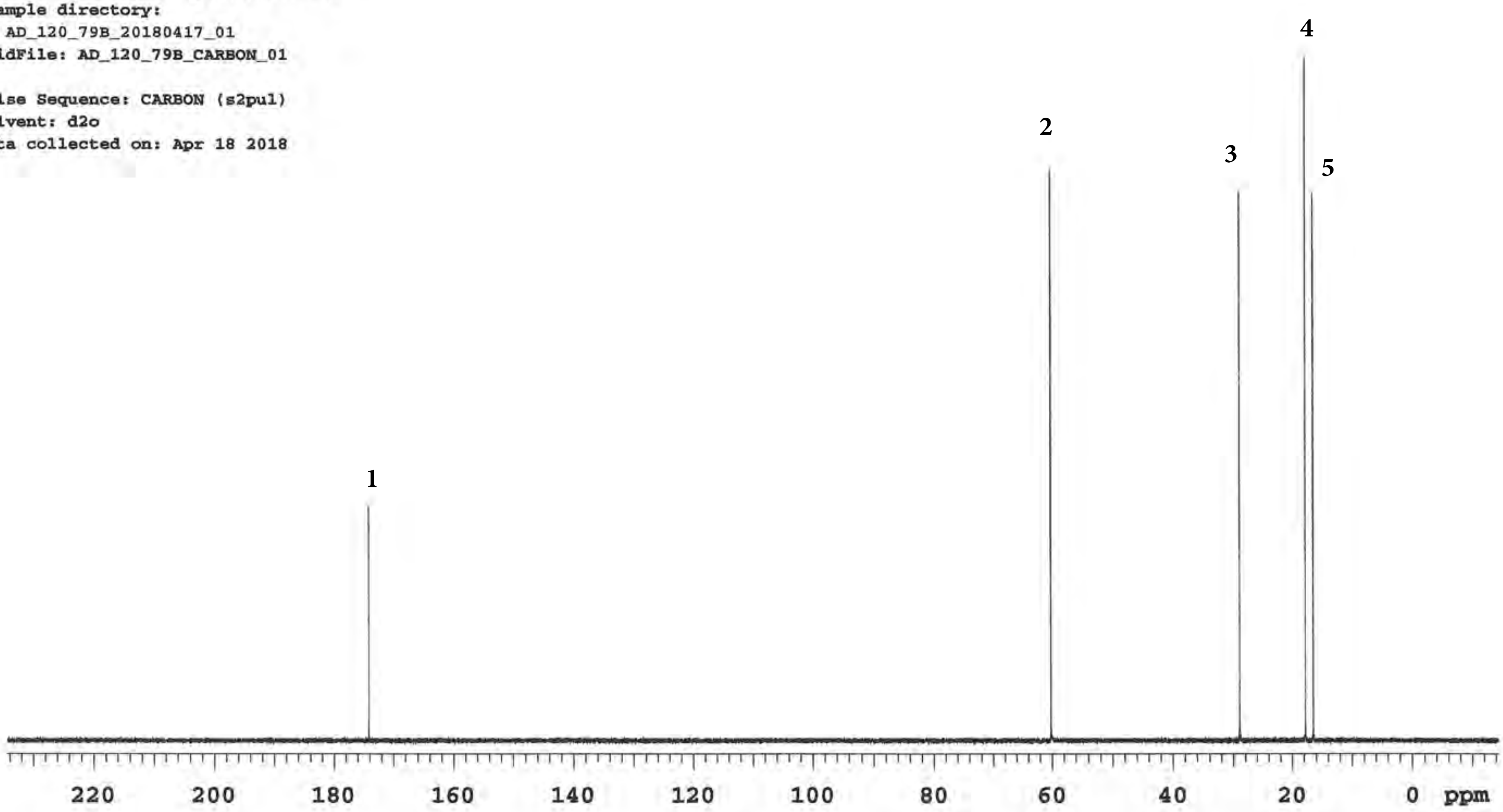

Figure K

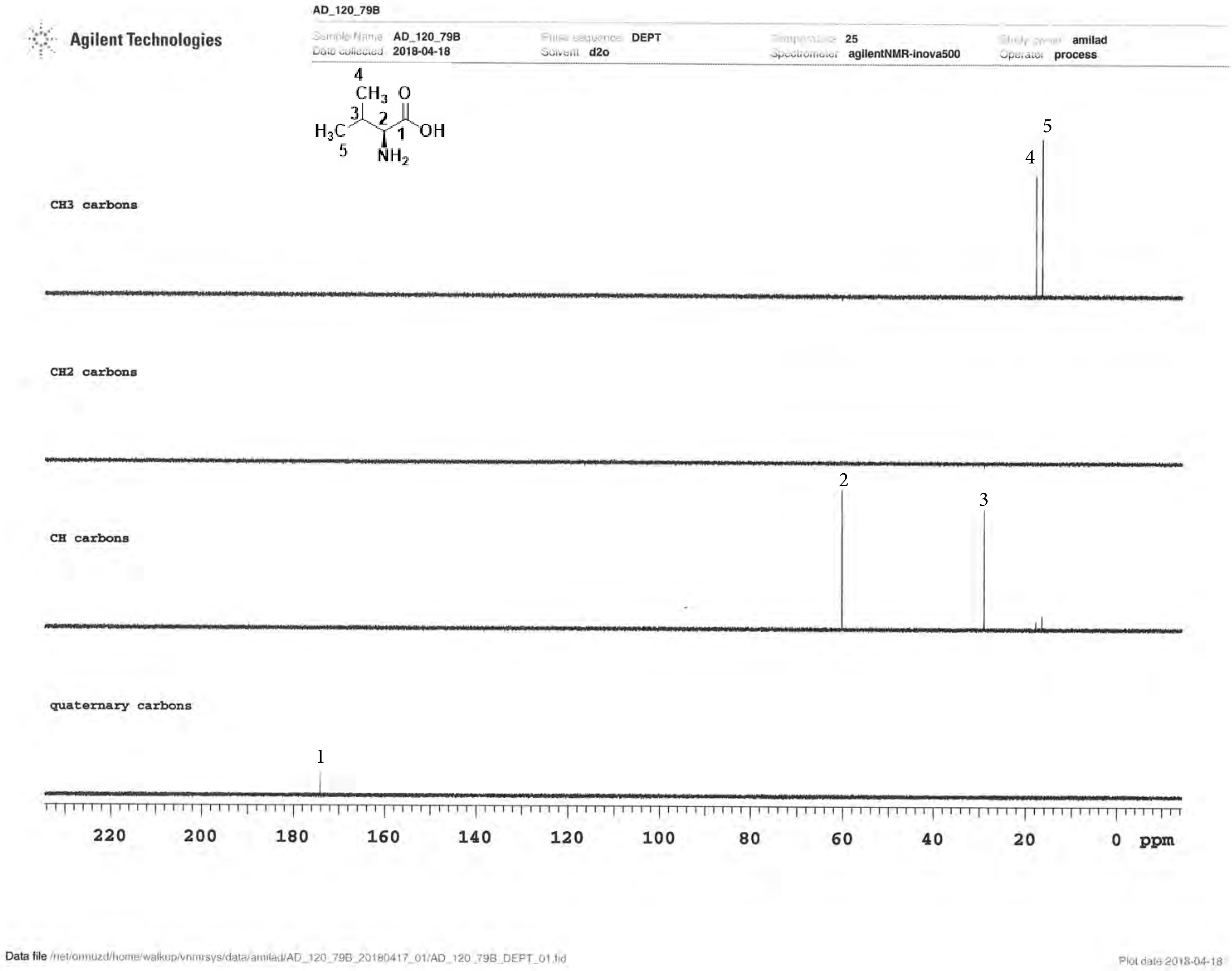
AD_120_79B
Agilent Technologies
AD_120_79B
2018-04-18
DEPT
Solvent d2o
25
agilentNMR-inova500
amilad
Operator process
CH3 carbons
CH2 carbons
CH carbons
quaternary carbons
1
2
3
4
5
220 200 180 160 140 120 100 80 60 40 20 0 ppm
Data file /net/ormuzd/home/walkup/vnmrsys/data/amilad/AD_120_79B_20180417_01/AD_120_79B_DEPT_01.fid
Plot date 2018-04-18

Figure L

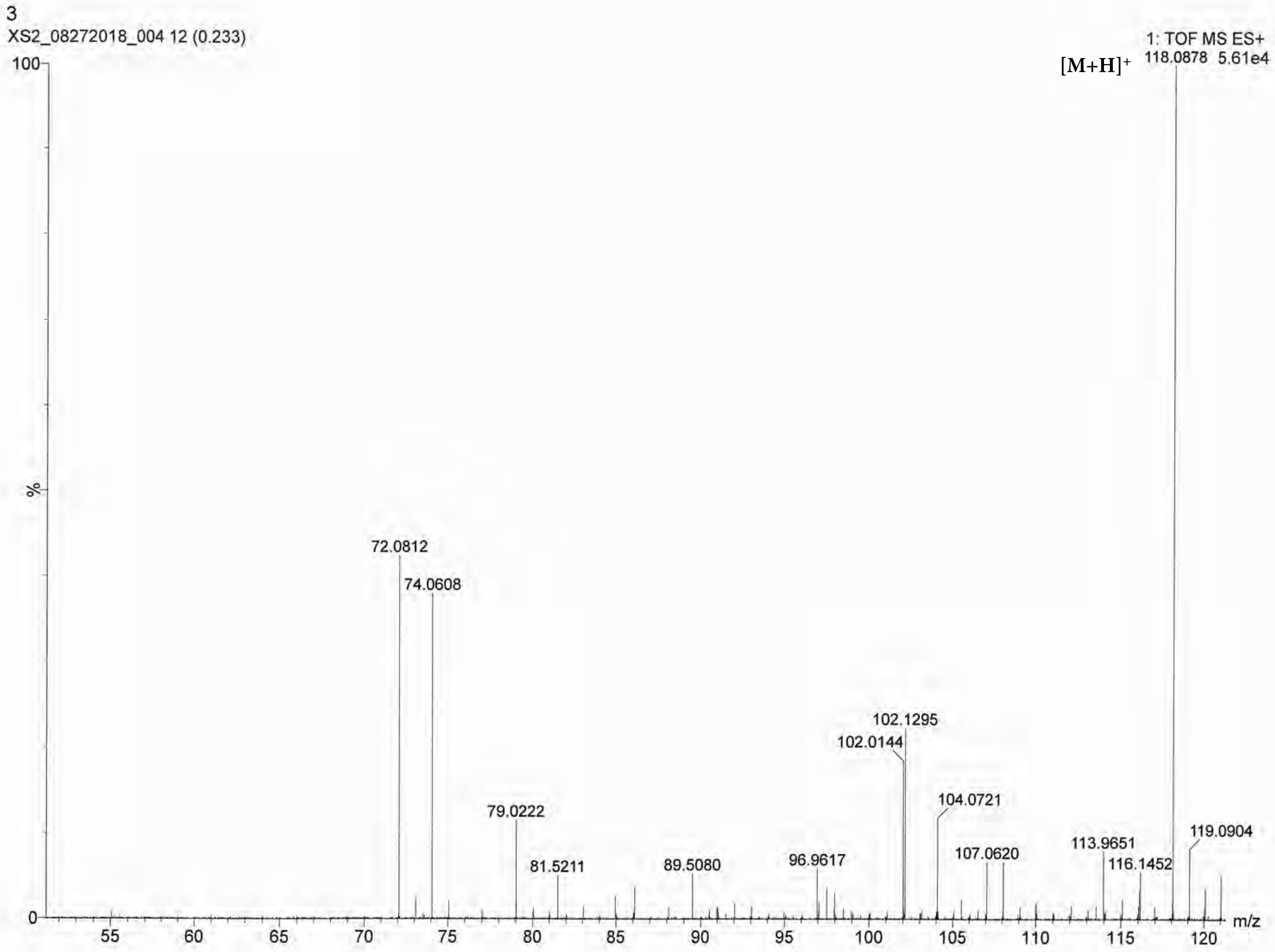
3
XS2_08272018_004 12 (0.233)
1: TOF MS ES+
118.0878 5.61e4
[M+H]+
100
%
0
72.0812
74.0608
79.0222
81.5211
89.5080
96.9617
102.1295
102.0144
104.0721
107.0620
113.9651
116.1452
119.0904
55
60
65
70
75
80
85
90
95
100
105
110
115
120
m/z

Figure M

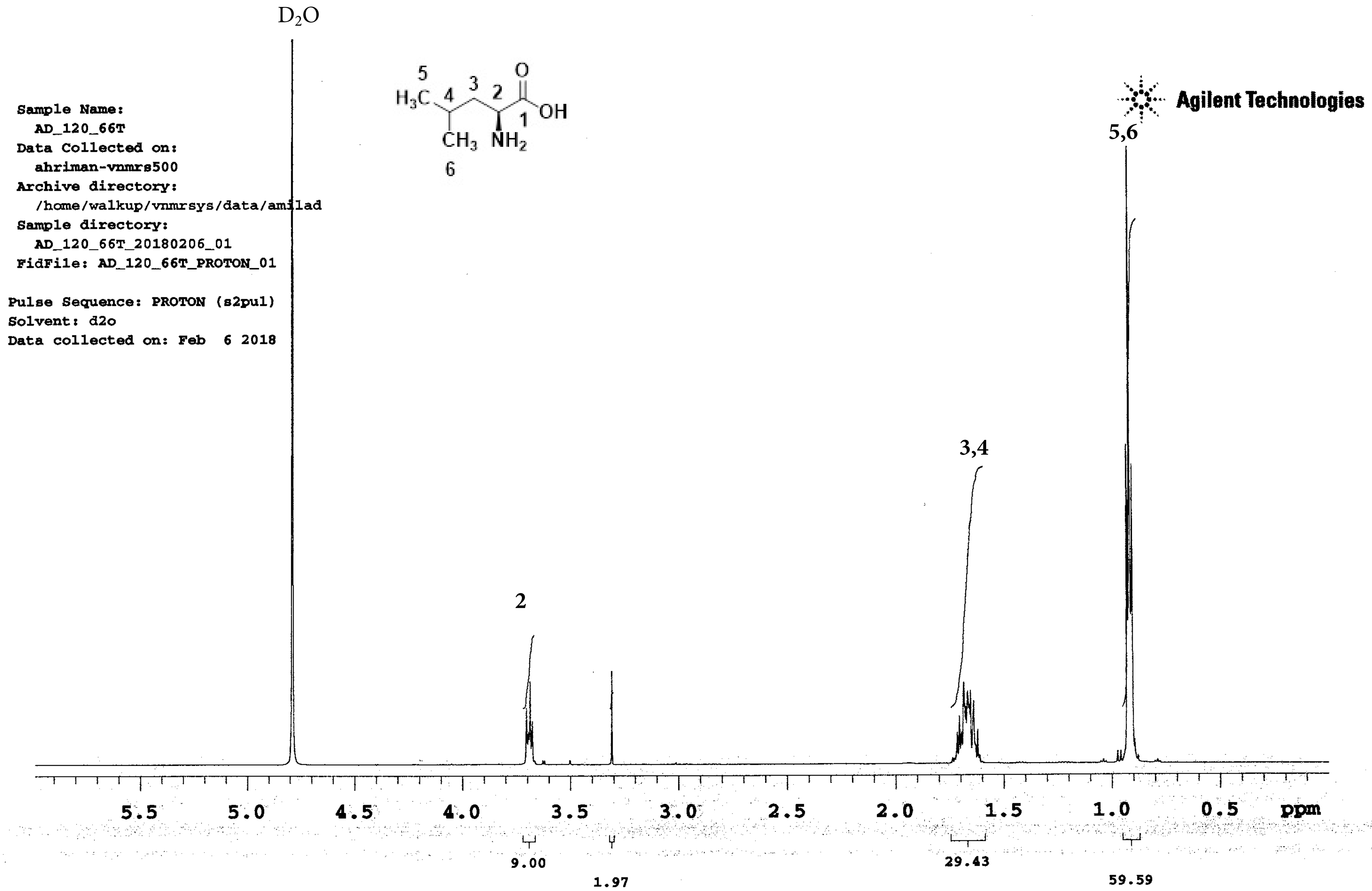
D$_2$O
5
3
2
O
H_3C
4
OH
1
CH_3
NH_2
6
Sample Name:
AD_120_66T
Data Collected on:
ahriman-vnmrs500
Archive directory:
/home/walkup/vnmrsys/data/amilad
Sample directory:
AD_120_66T_20180206_01
FidFile: AD_120_66T_PROTON_01
Pulse Sequence: PROTON (s2pul)
Solvent: d2o
Data collected on: Feb 6 2018
Agilent Technologies
5,6
3,4
2
5.5
5.0
4.5
4.0
3.5
3.0
2.5
2.0
1.5
1.0
0.5
ppm
9.00
1.97
29.43
59.59

Figure N

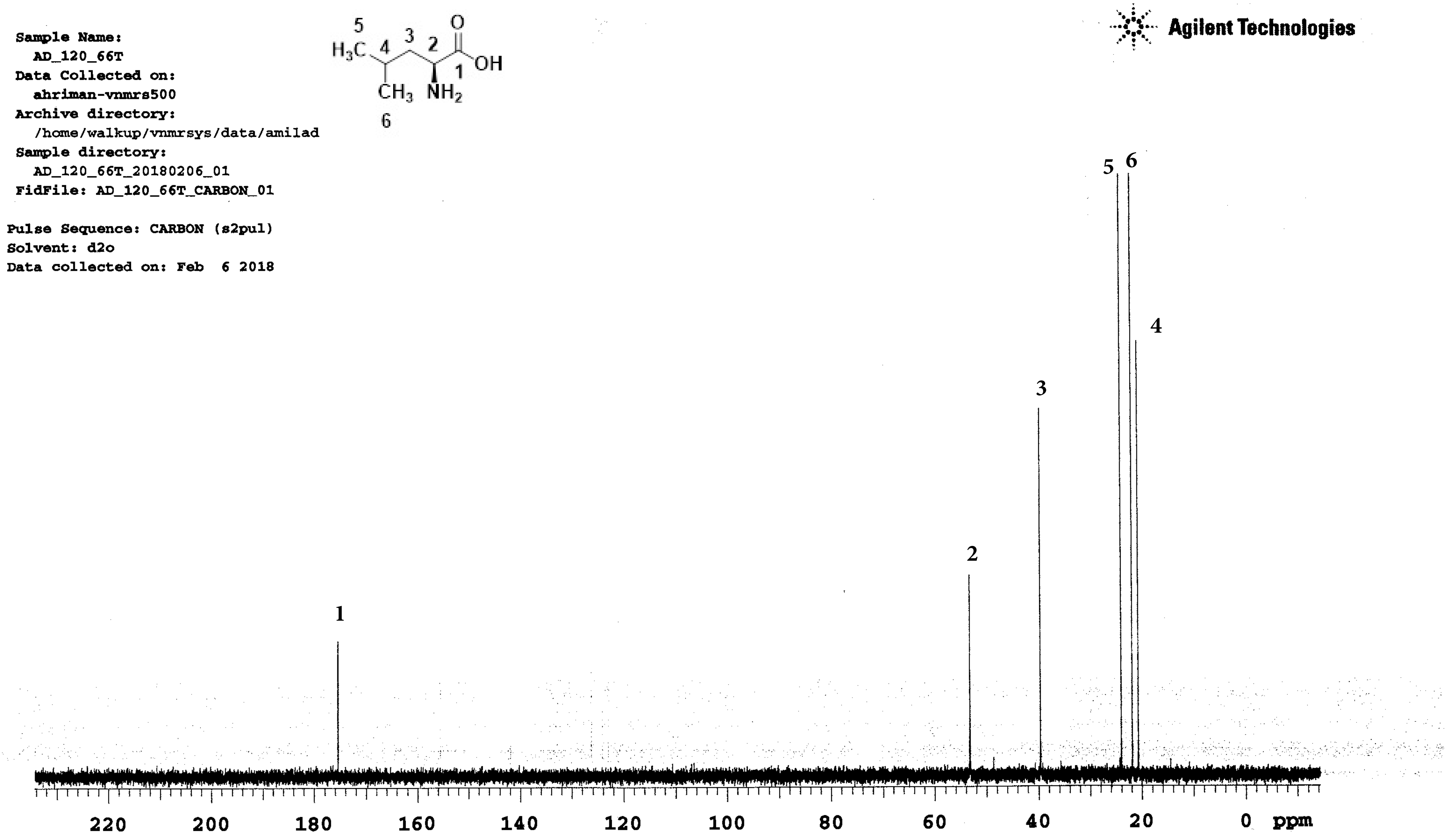

Sample Name:
AD_120_66T
Data Collected on:
ahriman-vnmrs500
Archive directory:
/home/walkup/vnmrsys/data/amilad
Sample directory:
AD_120_66T_20180206_01
FidFile: AD_120_66T_CARBON_01
Pulse Sequence: CARBON (s2pul)
Solvent: d2o
Data collected on: Feb 6 2018
Agilent Technologies
H3C
CH3
NH2
OH
O
1
2
3
4
5
6
220
200
180
160
140
120
100
80
60
40
20
0
ppm

Figure O

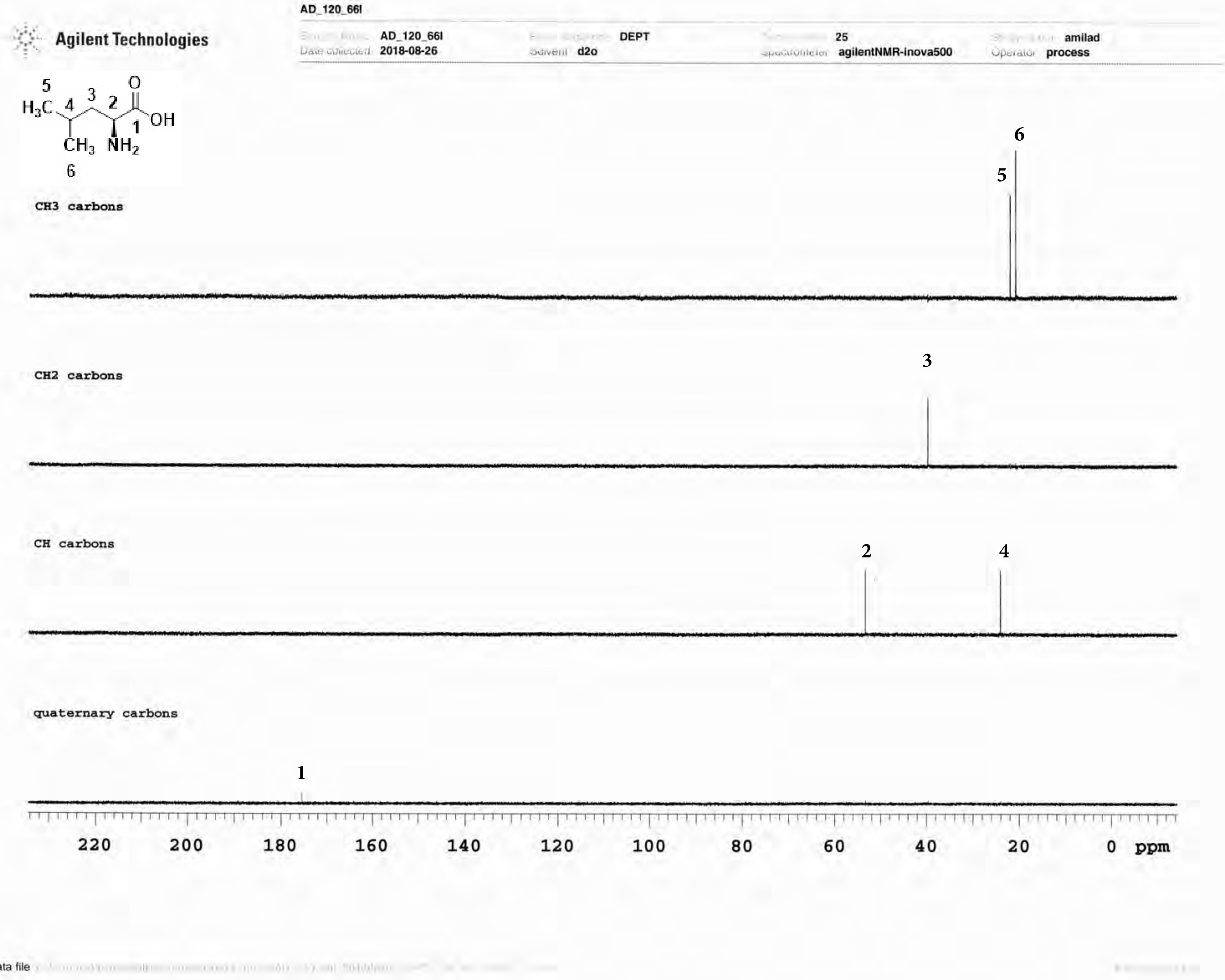
AD_120_66l
Agilent Technologies
AD_120_66l
Date collected 2018-08-26
DEPT
Solvent d2o
25
Spectrometer agilentNMR-inova500
amilad
Operator process
5
3
2
O
H3C
4
OH
1
CH3
NH2
6
CH3 carbons
6
5
CH2 carbons
3
CH carbons
2
4
quaternary carbons
1
220
200
180
160
140
120
100
80
60
40
20
0
ppm
Data file

Figure P

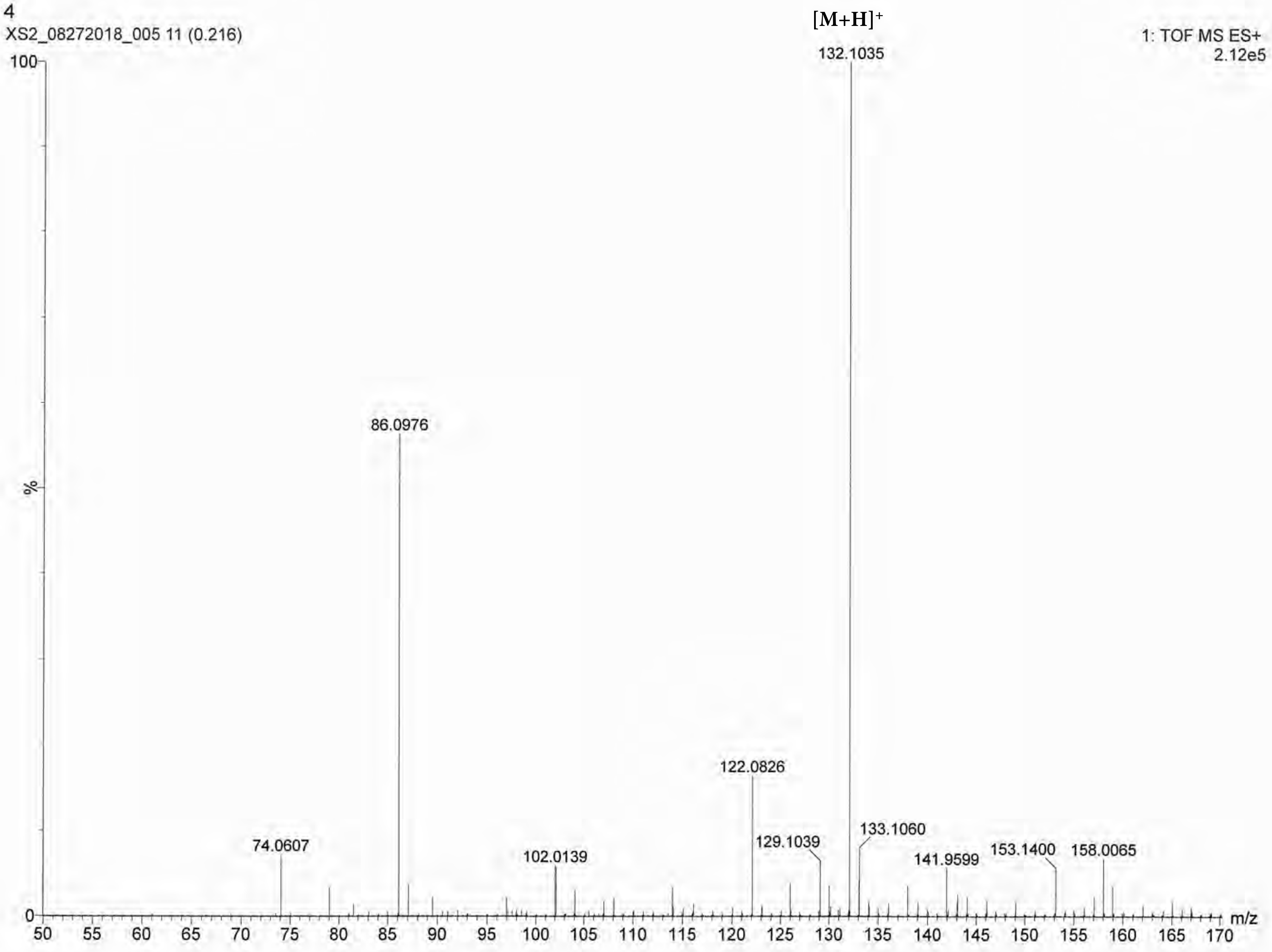
4
XS2_08272018_005 11 (0.216)
[M+H]+
1: TOF MS ES+
2.12e5
100
%
0
132.1035
86.0976
122.0826
74.0607
102.0139
129.1039
133.1060
141.9599
153.1400
158.0065
50
55
60
65
70
75
80
85
90
95
100
105
110
115
120
125
130
135
140
145
150
155
160
165
170
m/z

Figure Q

Sample Name:
AD_120_66E
Data Collected on:
ormuzd-vnmrs500
Archive directory:
/home/walkup/vnmrsys/data/amilad
Sample directory:
AD_120_66E_20180821_01
FidFile: AD_120_66E_PROTON_01

Pulse Sequence: PROTON (s2pul)
Solvent: d2o
Data collected on: Aug 21 2018

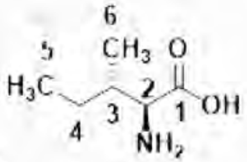

INDEX	FREQUENCY	PPM	HEIGHT
1	2393.5	4.790	47.0
2	[illegible]	[illegible]	[illegible]
3	1807.7	3.617	35.8
4	971.4	1.944	8.0
5	968.9	1.939	8.5
6	966.9	1.935	8.6
7	964.5	1.930	8.3
8	712.6	1.426	10.8
9	707.3	1.415	9.3
10	704.8	1.410	9.0
11	700.4	1.402	8.8
12	614.8	1.230	9.4
13	613.4	1.227	8.3
14	606.0	1.213	8.9
15	486.2	0.973	101.3
16	478.9	0.958	101.6
17	466.1	0.933	11.9
18	458.3	0.917	23.8
19	453.9	0.908	55.5
20	449.5	0.900	33.9
21	446.6	0.894	107.1
22	442.7	0.886	27.1
23	439.2	0.879	48.5

Agilent Technologies

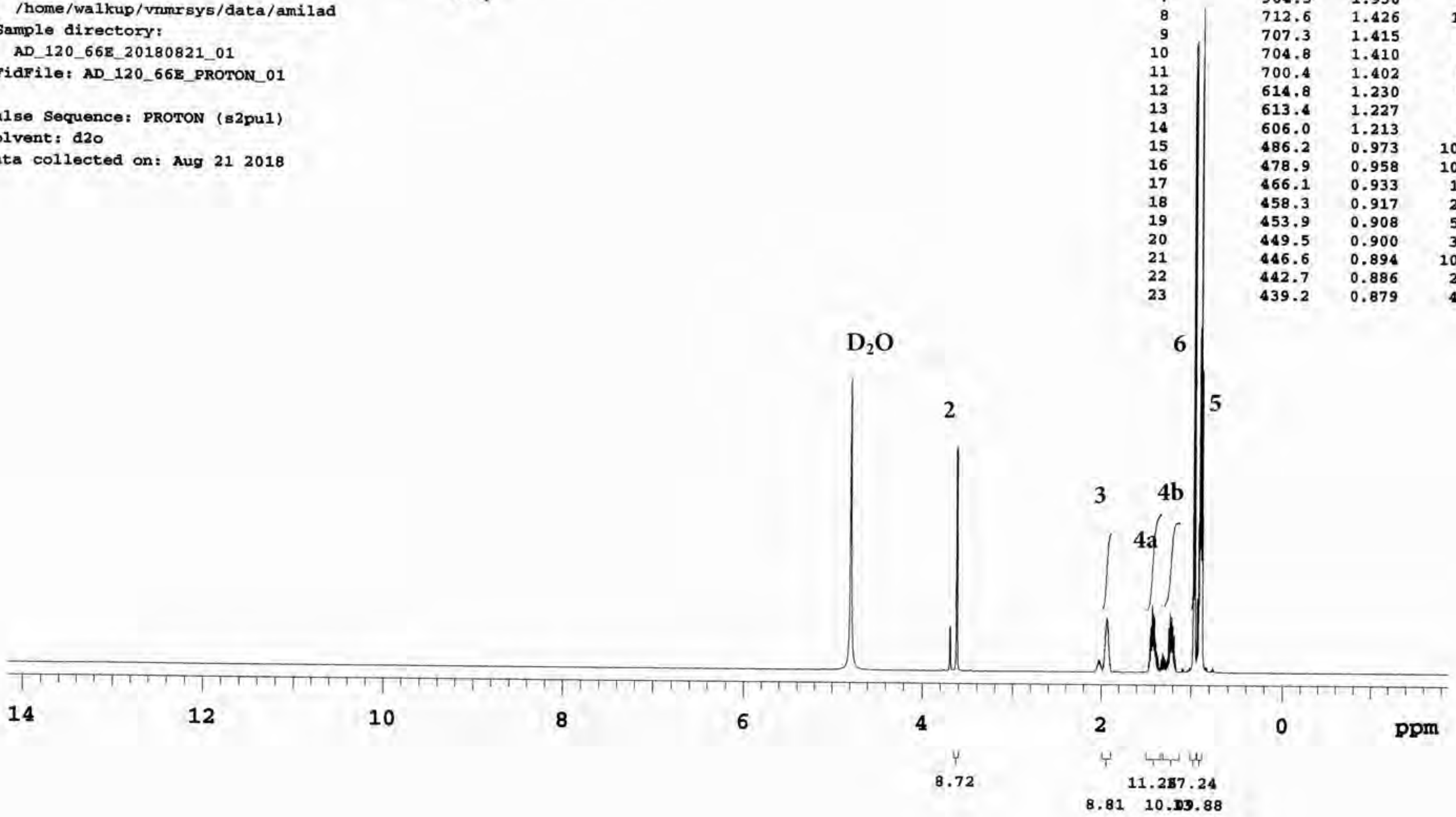

Figure R

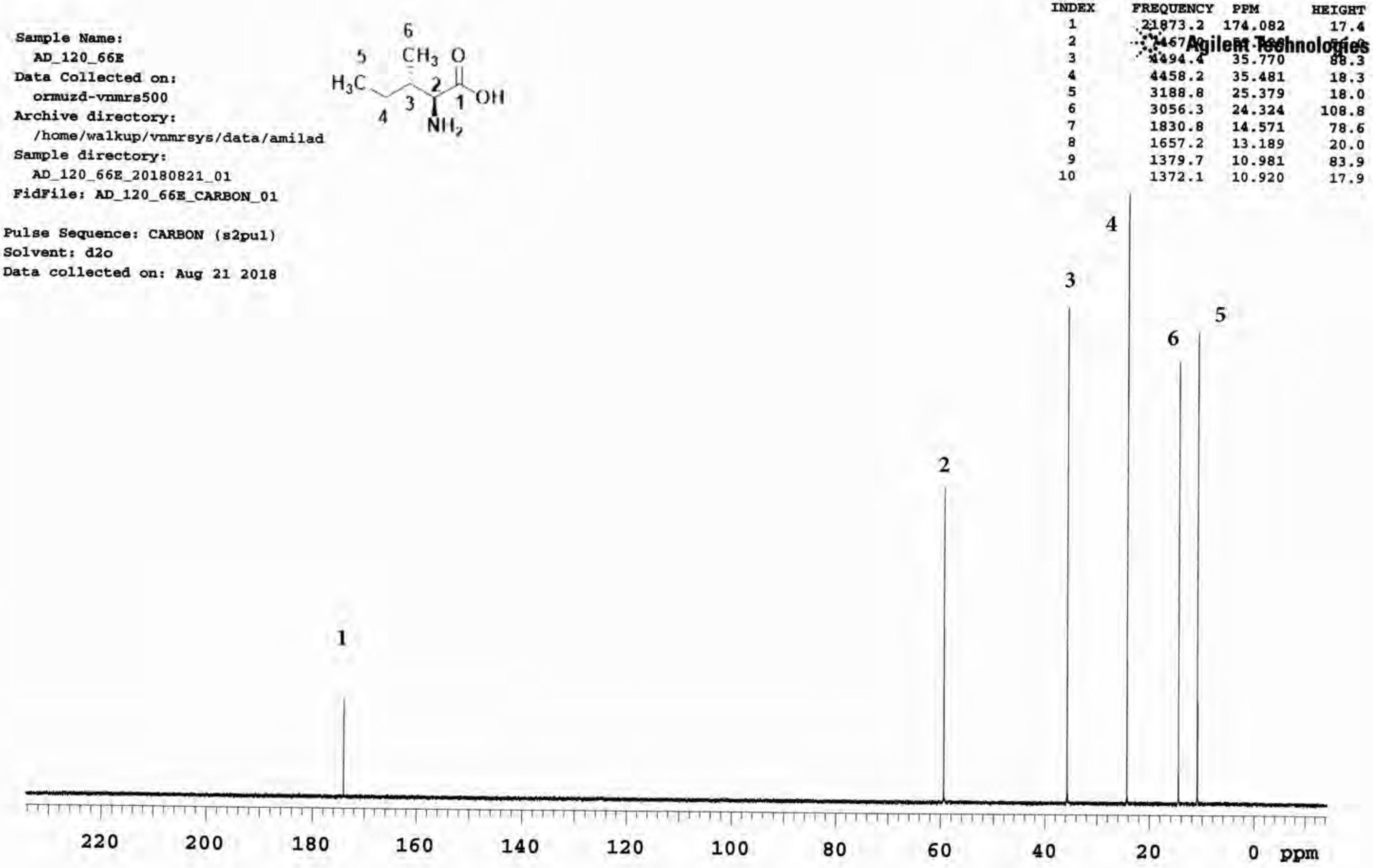

INDEX	FREQUENCY	PPM	HEIGHT
1	21873.2	174.082	17.4
2	[illegible]	[illegible]	[illegible]
3	4494.4	35.770	88.3
4	4458.2	35.481	18.3
5	3188.8	25.379	18.0
6	3056.3	24.324	108.8
7	1830.8	14.571	78.6
8	1657.2	13.189	20.0
9	1379.7	10.981	83.9
10	1372.1	10.920	17.9

Figure S

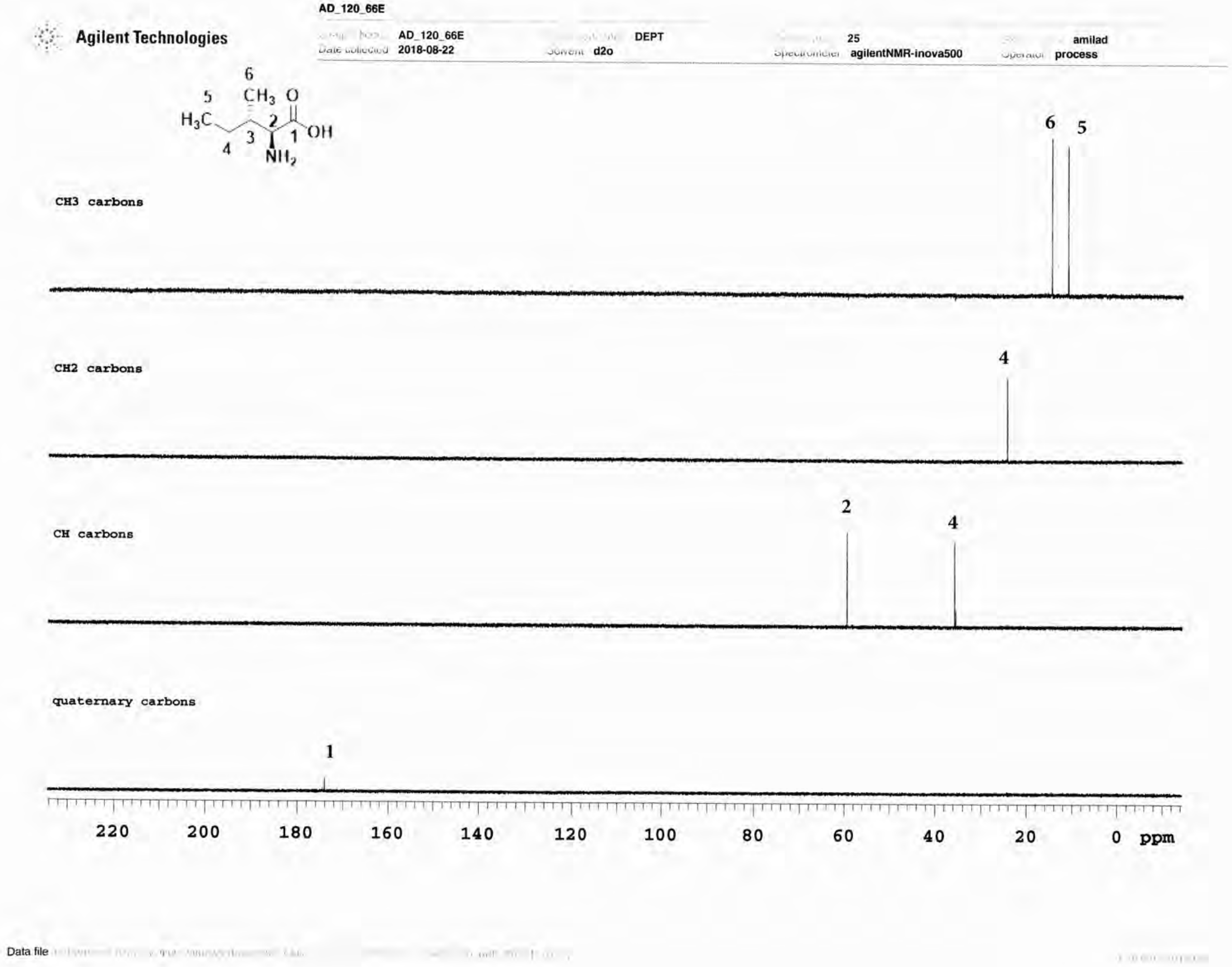
AD_120_66E
Agilent Technologies
AD_120_66E
2018-08-22
DEPT
d2o
25
agilentNMR-inova500
amilad
process
CH3 carbons
CH2 carbons
CH carbons
quaternary carbons
6
5
4
2
4
1
220
200
180
160
140
120
100
80
60
40
20
0
ppm
Data file

Figure T

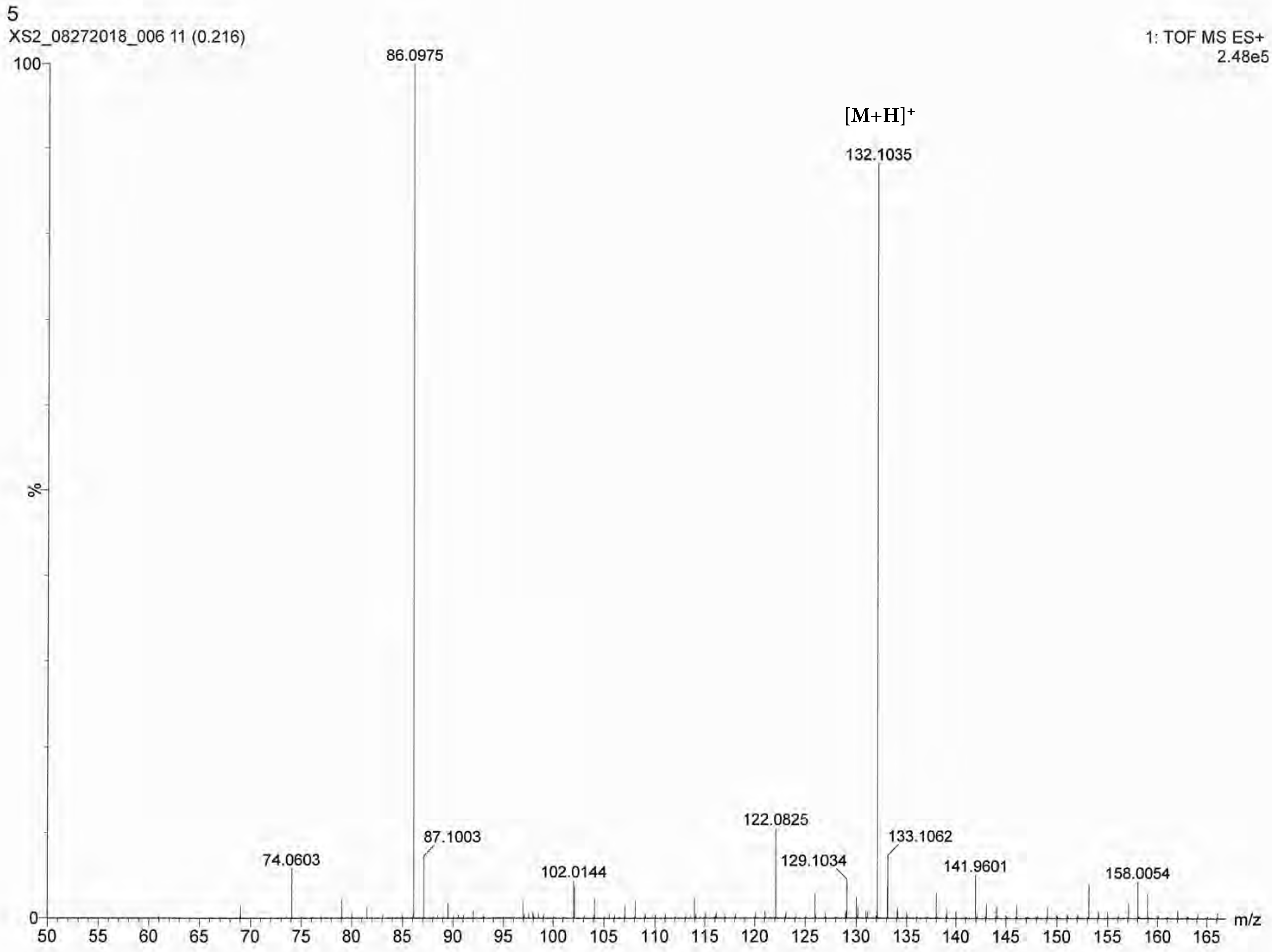
5
XS2_08272018_006 11 (0.216)
1: TOF MS ES+
2.48e5
100
%
0
86.0975
[M+H]+
132.1035
74.0603
87.1003
102.0144
122.0825
129.1034
133.1062
141.9601
158.0054
50
55
60
65
70
75
80
85
90
95
100
105
110
115
120
125
130
135
140
145
150
155
160
165
m/z

Figure U

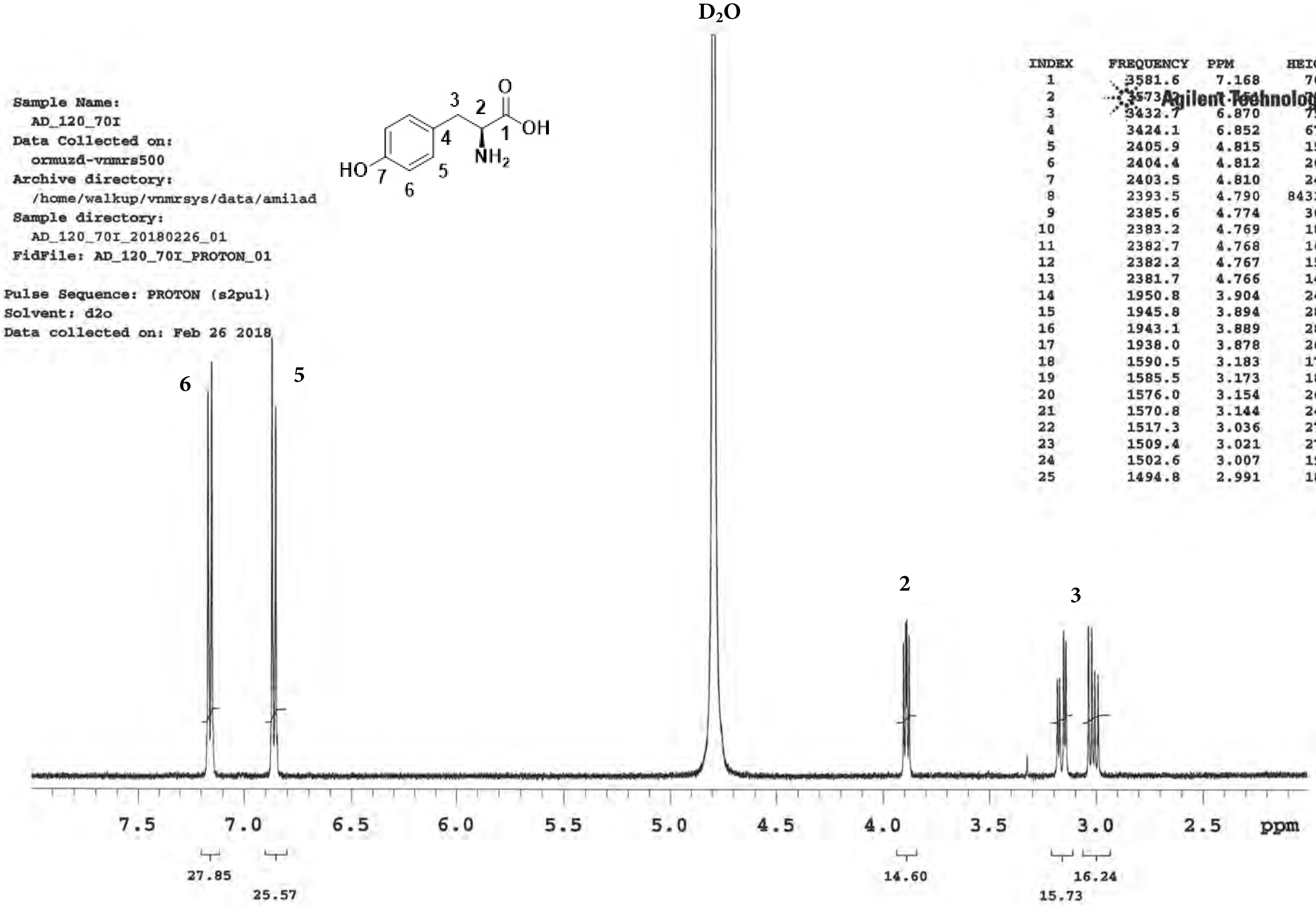

INDEX	FREQUENCY	PPM	HEIGHT
1	3581.6	7.168	70.1
2	[illegible]	[illegible]	[illegible]
3	3432.7	6.870	79.9
4	3424.1	6.852	67.3
5	2405.9	4.815	15.5
6	2404.4	4.812	20.5
7	2403.5	4.810	24.3
8	2393.5	4.790	8432.7
9	2385.6	4.774	30.1
10	2383.2	4.769	18.0
11	2382.7	4.768	16.7
12	2382.2	4.767	15.4
13	2381.7	4.766	14.4
14	1950.8	3.904	24.5
15	1945.8	3.894	28.3
16	1943.1	3.889	28.7
17	1938.0	3.878	26.0
18	1590.5	3.183	17.9
19	1585.5	3.173	18.2
20	1576.0	3.154	26.7
21	1570.8	3.144	24.9
22	1517.3	3.036	27.6
23	1509.4	3.021	27.3
24	1502.6	3.007	19.6
25	1494.8	2.991	18.7

Figure V

Sample Name:
AD_120_70I
Data Collected on:
ormuzd-vnmrs500
Archive directory:
/home/walkup/vnmrsys/data/amilad
Sample directory:
AD_120_70I_20180301_01
FidFile: AD_120_70I_CARBON_01

Pulse Sequence: CARBON (s2pul)
Solvent: d2o
Data collected on: Mar 1 2018

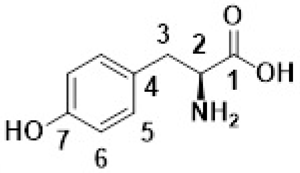

INDEX	FREQUENCY	PPM	HEIGHT
1	21887.1	174.192	10.6
2	[illegible]	[illegible]	[illegible]
3	16416.3	130.652	90.0
4	15919.9	126.702	19.5
5	14543.5	115.747	90.7
6	7038.6	56.018	40.1
7	4458.7	35.485	37.7

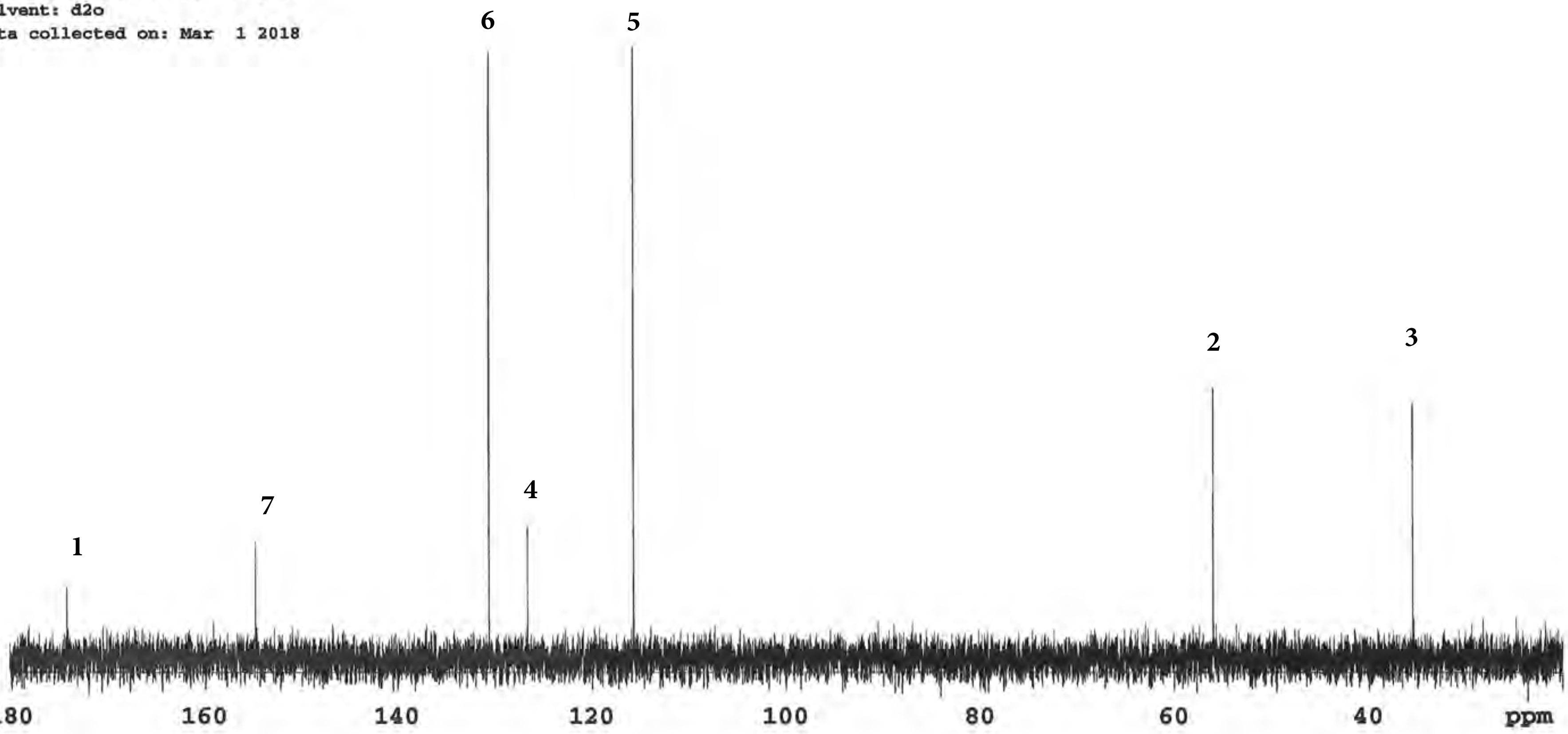

Figure W

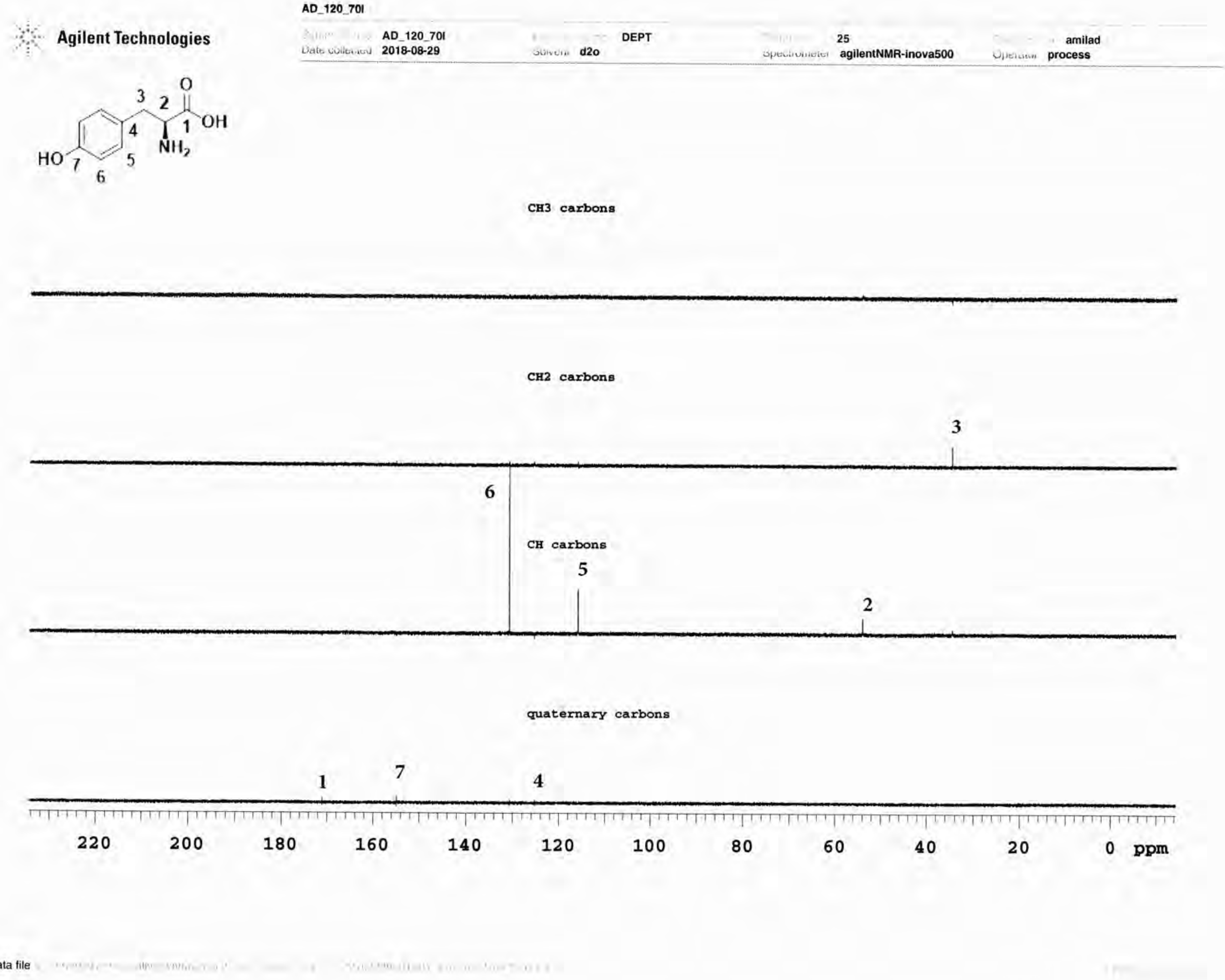
Agilent Technologies
AD_120_70l
AD_120_70l
Date collected 2018-08-29
DEPT
Solvent d2o
25
Spectrometer agilentNMR-inova500
amilad
Operator process
3 2 O
4 1 OH
NH_2
HO 7 6 5
CH3 carbons
CH2 carbons
3
6
CH carbons
5
2
quaternary carbons
1
7
4
220 200 180 160 140 120 100 80 60 40 20 0 ppm
Data file

Figure X

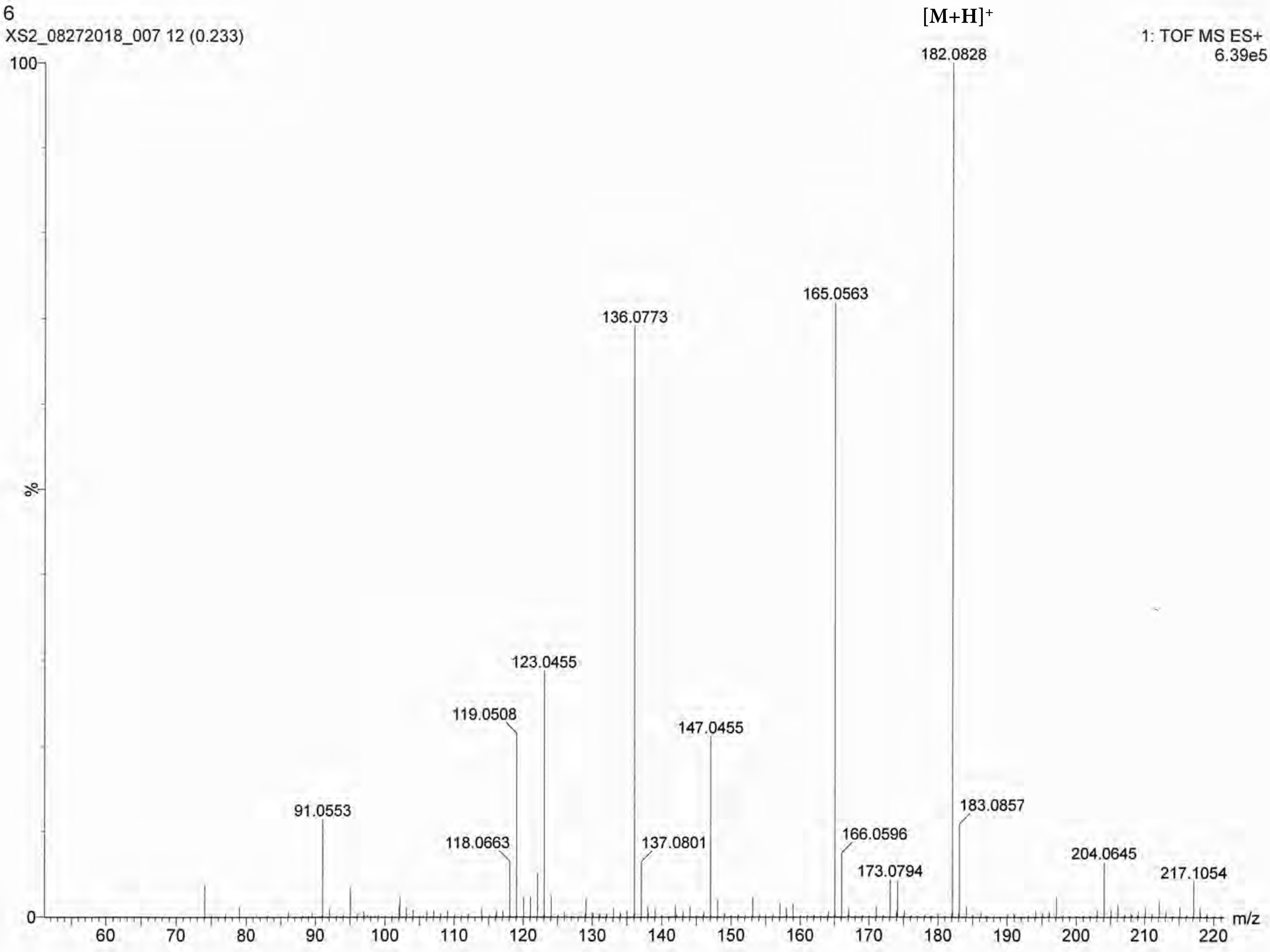
6
XS2_08272018_007 12 (0.233)
[M+H]+
1: TOF MS ES+
6.39e5
182.0828
100
%
0
165.0563
136.0773
123.0455
119.0508
147.0455
91.0553
118.0663
137.0801
183.0857
166.0596
173.0794
204.0645
217.1054
60
70
80
90
100
110
120
130
140
150
160
170
180
190
200
210
220
m/z
