# Supplementary material for: Nitrogenous compounds characterized in the deterrent skin extract of migratory adult sea lamprey from the Great Lakes region
Source: PLoS One. 2019 May 23;14(5):e0217417. doi: 10.1371/journal.pone.0217417 (PMC6532902; doi:10.1371/journal.pone.0217417)
Supplement: S3 File — (PDF) [file pone.0217417.s003.pdf]

Nitrogenous Compounds Characterized in the deterrent Skin extract of Migratory Adult Sea Lamprey from the Great Lakes Region

Amila A. Dissanayake,[1] C. Michael Wagner,[2] Muraleedharan G. Nair,[1*]

[1] Department of Horticulture, Michigan State University, East Lansing, Michigan, United States of America

[2] Department of Fisheries and Wildlife, Michigan State University, East Lansing, Michigan, United States of America

Supporting Information

Figure A. ^{1}H NMR spectrum of hypoxanthine in DMSO
Figure B. ^{13}C NMR spectrum of hypoxanthine in DMSO
Figure C. DEPT spectrum of hypoxanthine in DMSO
Figure D. HRMS spectrum of hypoxanthine (positive ion mode)
Figure E. ^{1}H NMR spectrum of inosine in DMSO
Figure F. ^{13}C NMR spectrum of inosine in DMSO
Figure G. DEPT spectrum of inosine in DMSO
Figure H. HRMS spectrum of inosine (positive ion mode)
Figure I. ^{1}H NMR spectrum of adenine in DMSO
Figure J. ^{13}C NMR spectrum of adenine in DMSO
Figure K. DEPT spectrum of adenine in DMSO
Figure L. HRMS spectrum of adenine (positive ion mode)
Figure M. ^{1}H NMR spectrum of xanthine in DMSO
Figure N. ^{13}C NMR spectrum of xanthine in DMSO
Figure O. DEPT spectrum of xanthine in DMSO
Figure P. HRMS spectrum of xanthine (positive ion mode)
Figure Q. ^{1}H NMR spectrum of histidine in D_2O
Figure R. ^{13}C NMR spectrum of histidine in D_2O
Figure S. DEPT spectrum of histidine in D_2O
Figure T. HRMS spectrum of histidine (positive ion mode)
Figure U. ^{1}H NMR spectrum of glutamic acid in D_2O
Figure V. ^{13}C NMR spectrum of glutamic acid in D_2O
Figure W. DEPT spectrum of glutamic acid in D_2O
Figure X. HRMS spectrum of glutamic acid (positive ion mode)

Figure A

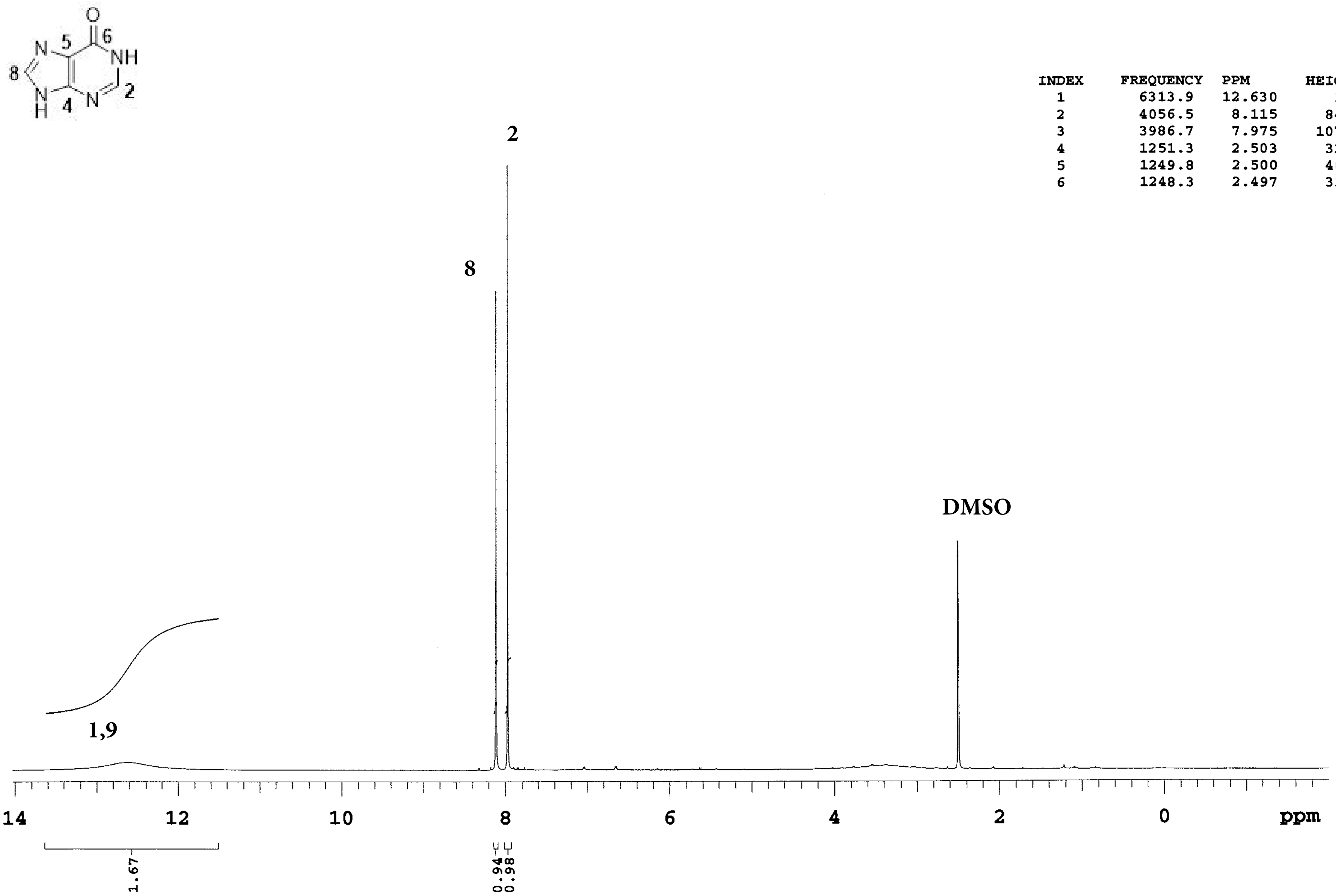

O
6
NH
5
N
8
2
N
H
4
N
INDEX FREQUENCY PPM HEIGHT
1 6313.9 12.630 1.4
2 4056.5 8.115 84.9
3 3986.7 7.975 107.1
4 1251.3 2.503 32.1
5 1249.8 2.500 40.5
6 1248.3 2.497 31.1
2
8
DMSO
1,9
14
12
10
8
6
4
2
0
ppm
1.67
0.94
0.98

Figure B

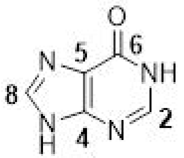

Sample Name:
AD_120_53E
Data Collected on:
ahriman-vnmrs500
Archive directory:
/home/walkup/vnmrsys/data/amilad
Sample directory:
AD_120_53E_20170726_02
FidFile: AD_120_53E_CARBON_01

Pulse Sequence: CARBON (s2pul)
Solvent: dmso
Data collected on: Jul 26 2017

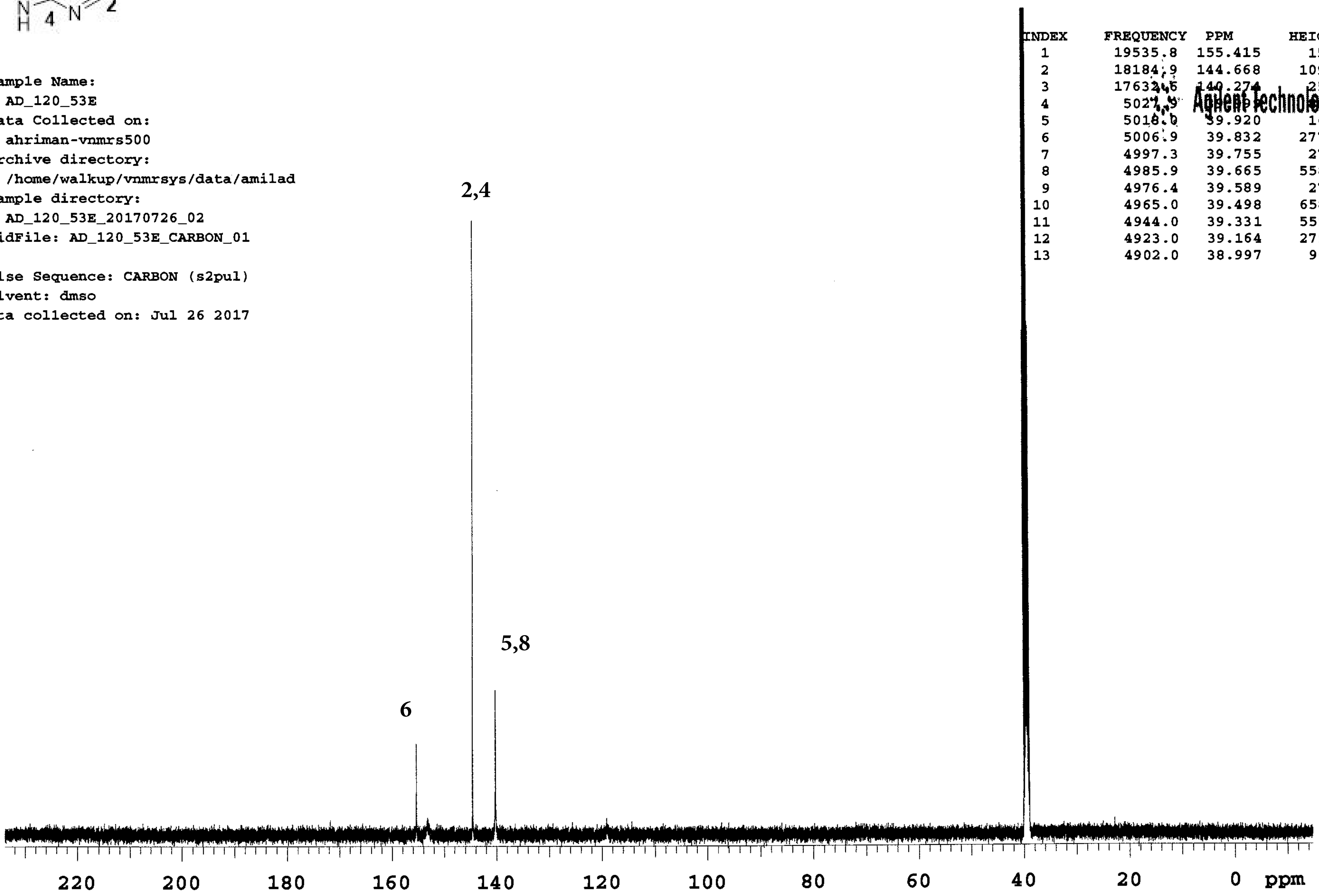

INDEX	FREQUENCY	PPM	HEIGHT
1	19535.8	155.415	15.8
2	18184.9	144.668	109.5
3	1763[illegible].6	140.274	25.7
4	502[illegible].9	[illegible]	[illegible]
5	5018.0	39.920	1[illegible].4
6	5006.9	39.832	277.1
7	4997.3	39.755	27.1
8	4985.9	39.665	558.0
9	4976.4	39.589	27.0
10	4965.0	39.498	658.2
11	4944.0	39.331	559.7
12	4923.0	39.164	279.5
13	4902.0	38.997	90.4

Figure C

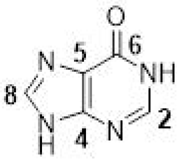

AD_120_53E

Sample Name	AD_120_53E	Pulse sequence	DEPT	Temperature	25	Study owner	amilad
Date collected	2017-07-27	Solvent	dmso	Spectrometer	ahriman-vnmrs500	Operator	amilad

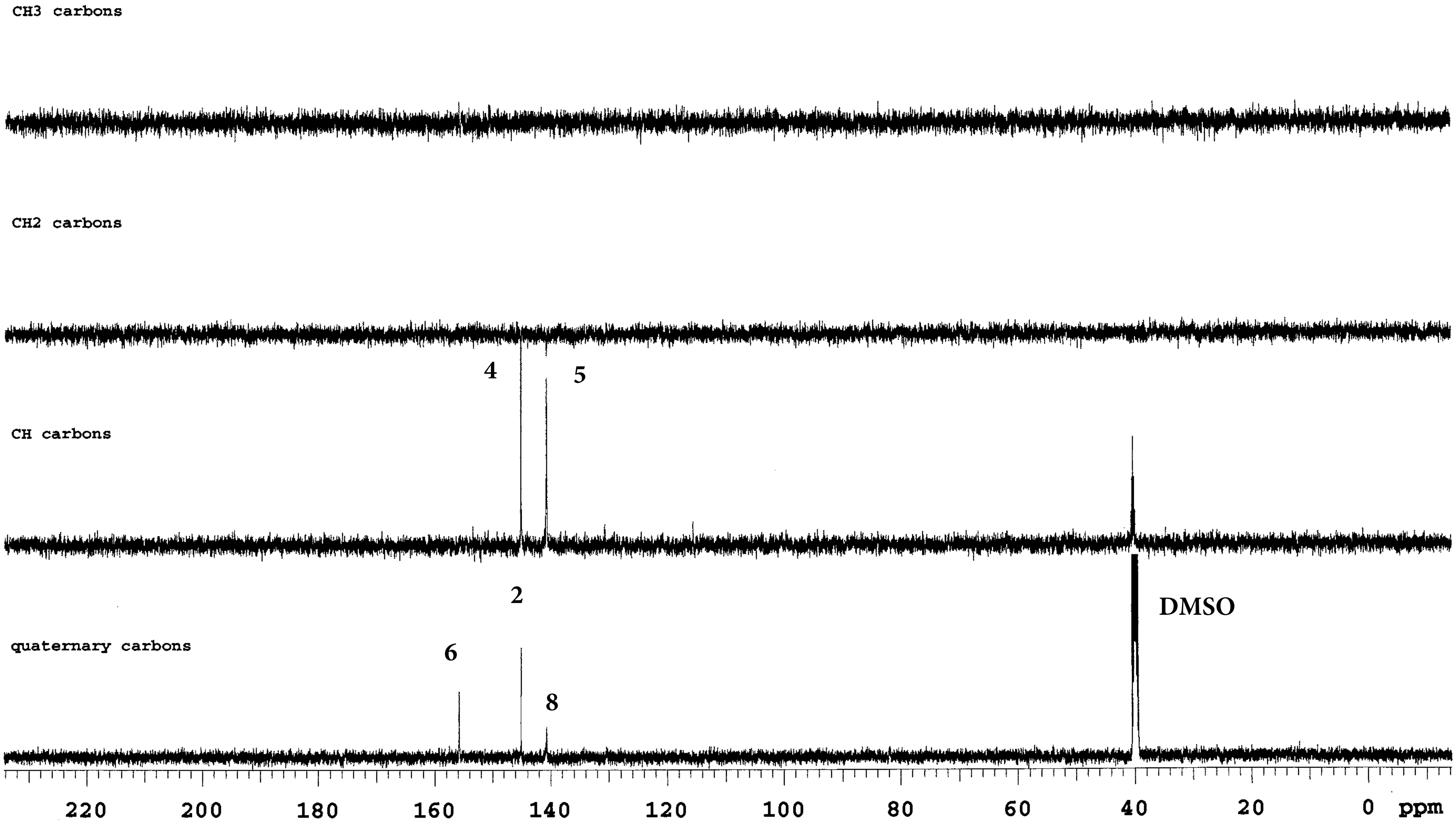

Data file [illegible]

Figure D

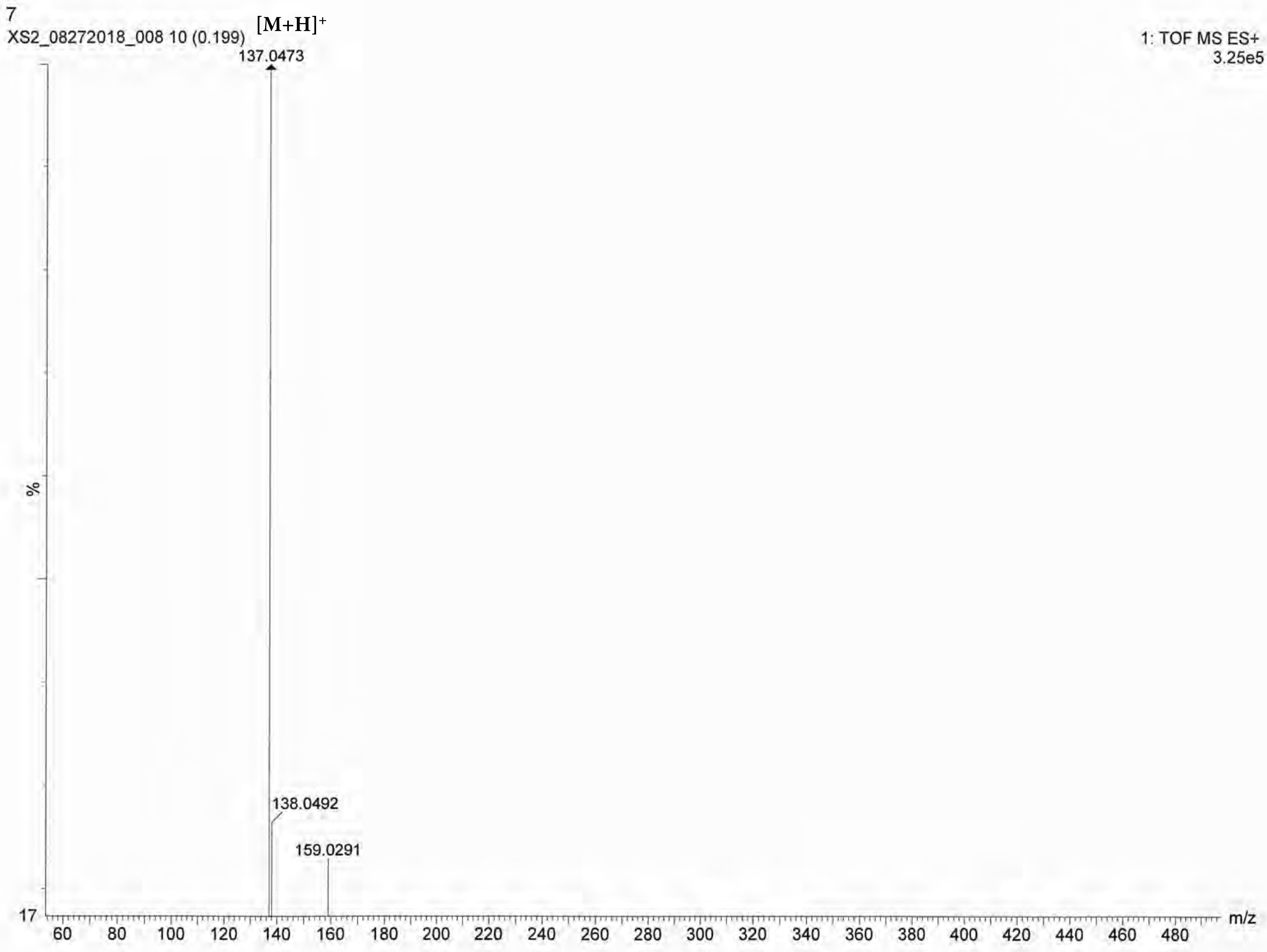
7
XS2_08272018_008 10 (0.199)
[M+H]+
137.0473
1: TOF MS ES+
3.25e5
%
138.0492
159.0291
17
60 80 100 120 140 160 180 200 220 240 260 280 300 320 340 360 380 400 420 440 460 480
m/z

Figure E

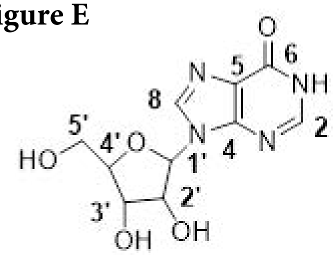

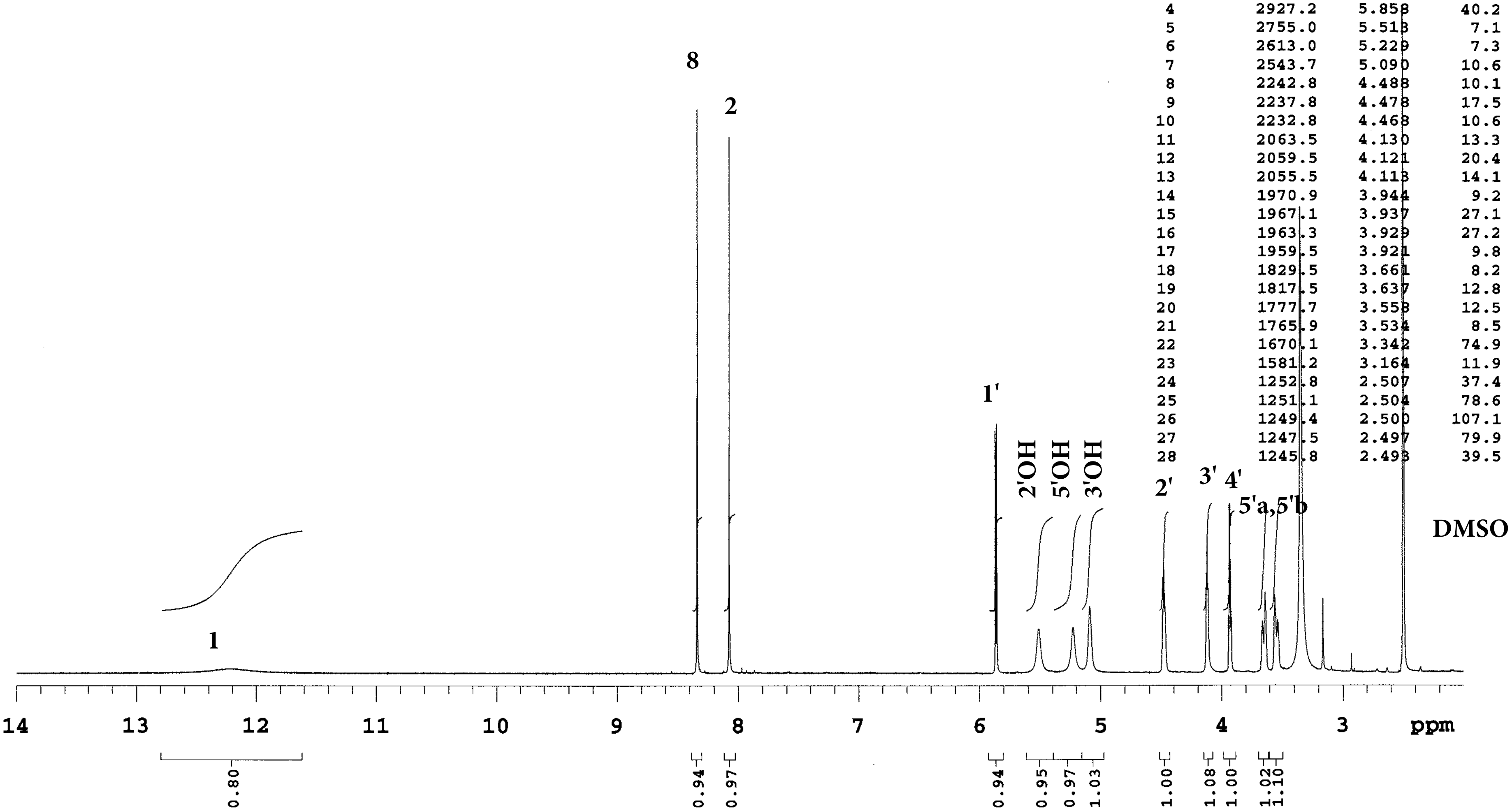

INDEX	FREQUENCY	PPM	HEIGHT
1	4167.4	8.340	90.8
2	4035.3	8.076	86.3
3	2933.0	5.869	39.0
4	2927.2	5.858	40.2
5	2755.0	5.513	7.1
6	2613.0	5.229	7.3
7	2543.7	5.090	10.6
8	2242.8	4.488	10.1
9	2237.8	4.478	17.5
10	2232.8	4.468	10.6
11	2063.5	4.130	13.3
12	2059.5	4.121	20.4
13	2055.5	4.113	14.1
14	1970.9	3.944	9.2
15	1967.1	3.937	27.1
16	1963.3	3.929	27.2
17	1959.5	3.921	9.8
18	1829.5	3.661	8.2
19	1817.5	3.637	12.8
20	1777.7	3.558	12.5
21	1765.9	3.534	8.5
22	1670.1	3.342	74.9
23	1581.2	3.164	11.9
24	1252.8	2.507	37.4
25	1251.1	2.504	78.6
26	1249.4	2.500	107.1
27	1247.5	2.497	79.9
28	1245.8	2.493	39.5

Figure F

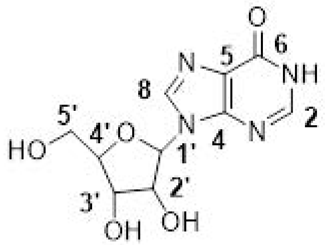

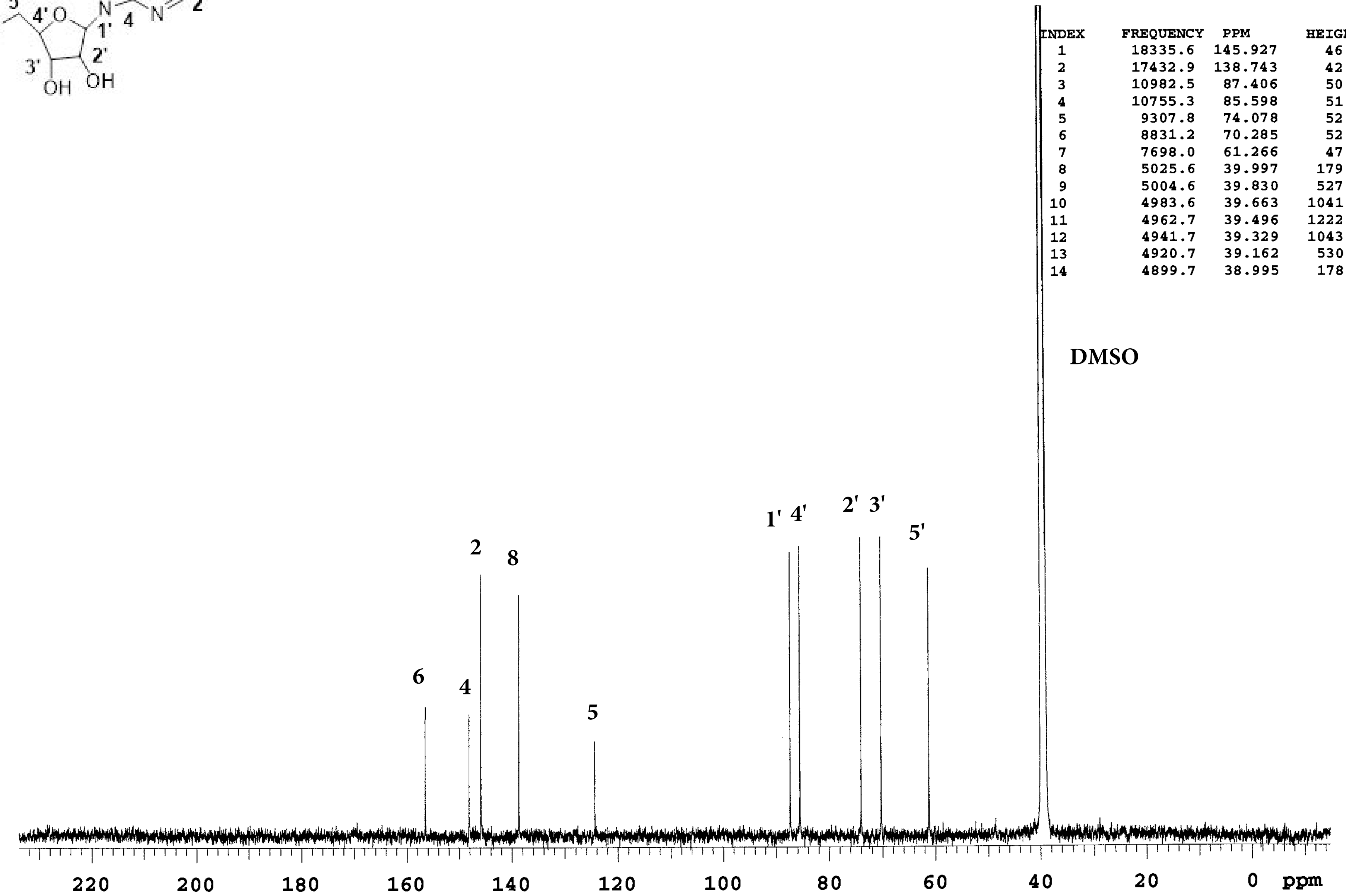

INDEX	FREQUENCY	PPM	HEIGHT
1	18335.6	145.927	46.4
2	17432.9	138.743	42.7
3	10982.5	87.406	50.2
4	10755.3	85.598	51.3
5	9307.8	74.078	52.7
6	8831.2	70.285	52.8
7	7698.0	61.266	47.2
8	5025.6	39.997	179.3
9	5004.6	39.830	527.0
10	4983.6	39.663	1041.0
11	4962.7	39.496	1222.1
12	4941.7	39.329	1043.6
13	4920.7	39.162	530.1
14	4899.7	38.995	178.8

Figure G

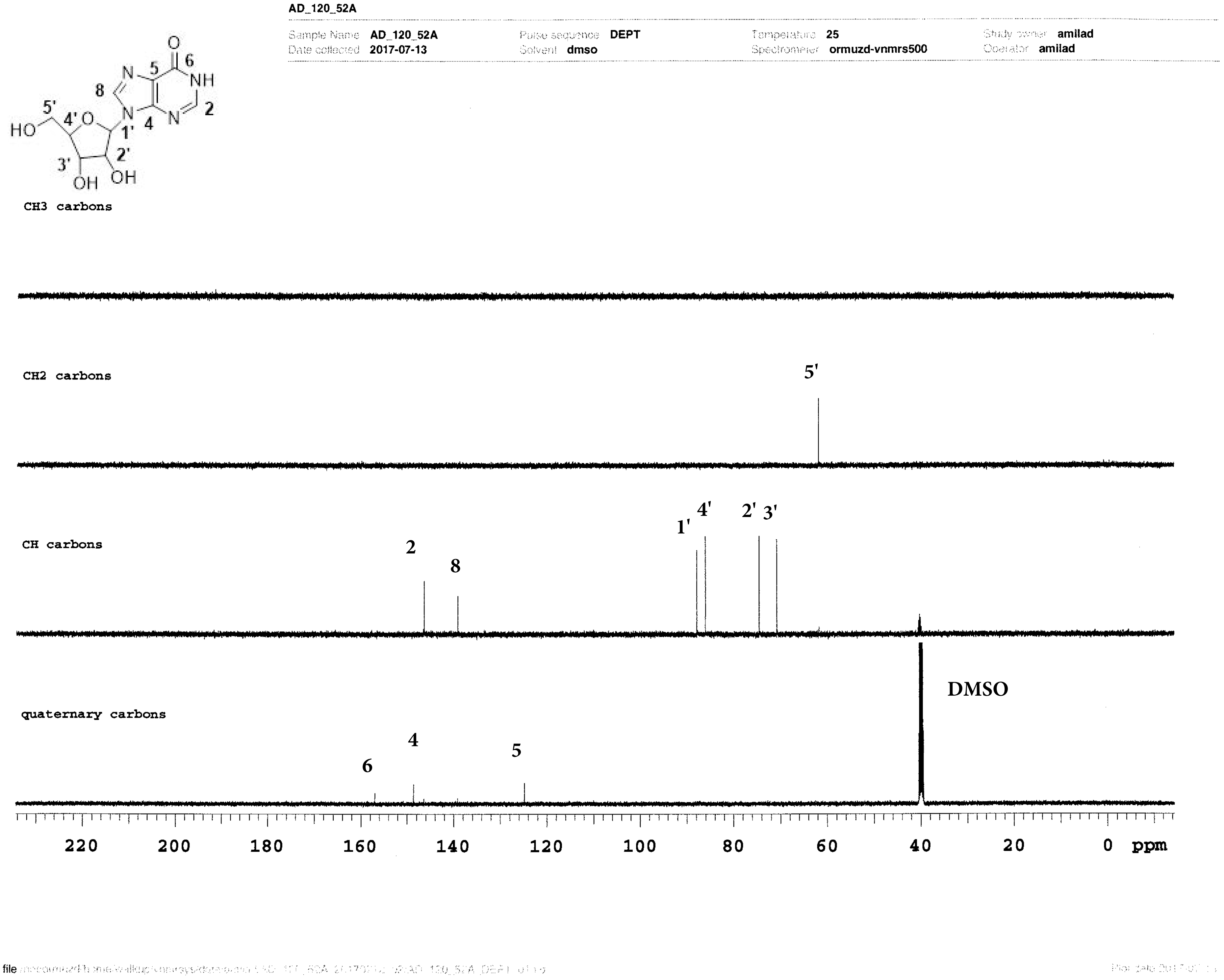

AD_120_52A
Sample Name AD_120_52A
Date collected 2017-07-13
Pulse sequence DEPT
Solvent dmso
Temperature 25
Spectrometer ormuzd-vnmrs500
Study owner amilad
Operator amilad
CH3 carbons
CH2 carbons
5'
CH carbons
2
8
1'
4'
2'
3'
quaternary carbons
6
4
5
DMSO
220
200
180
160
140
120
100
80
60
40
20
0
ppm
Data file

Figure H

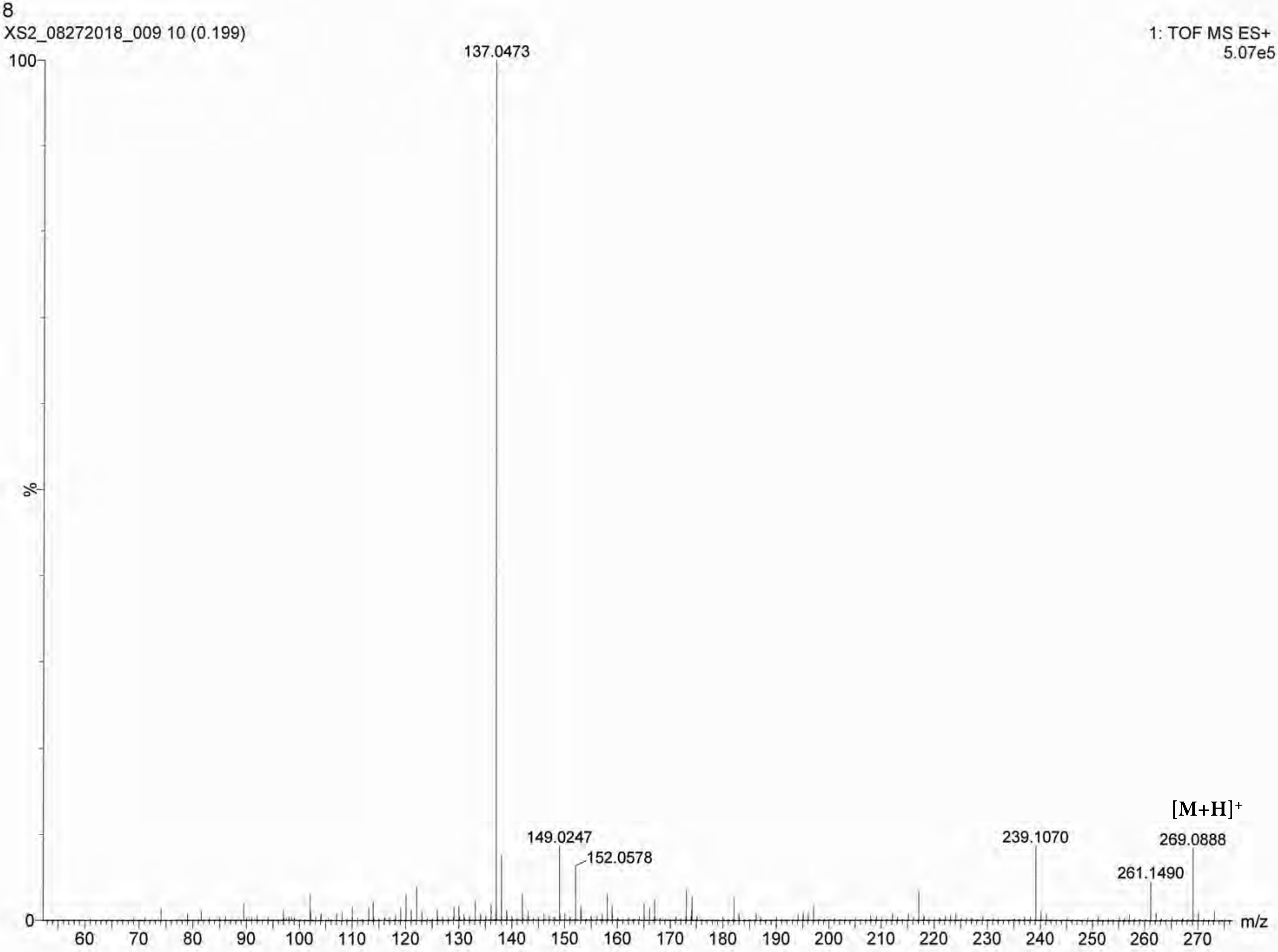

8
XS2_08272018_009 10 (0.199)
1: TOF MS ES+
5.07e5
100
%
0
137.0473
149.0247
152.0578
239.1070
[M+H]+
269.0888
261.1490
60
70
80
90
100
110
120
130
140
150
160
170
180
190
200
210
220
230
240
250
260
270
m/z

Figure I

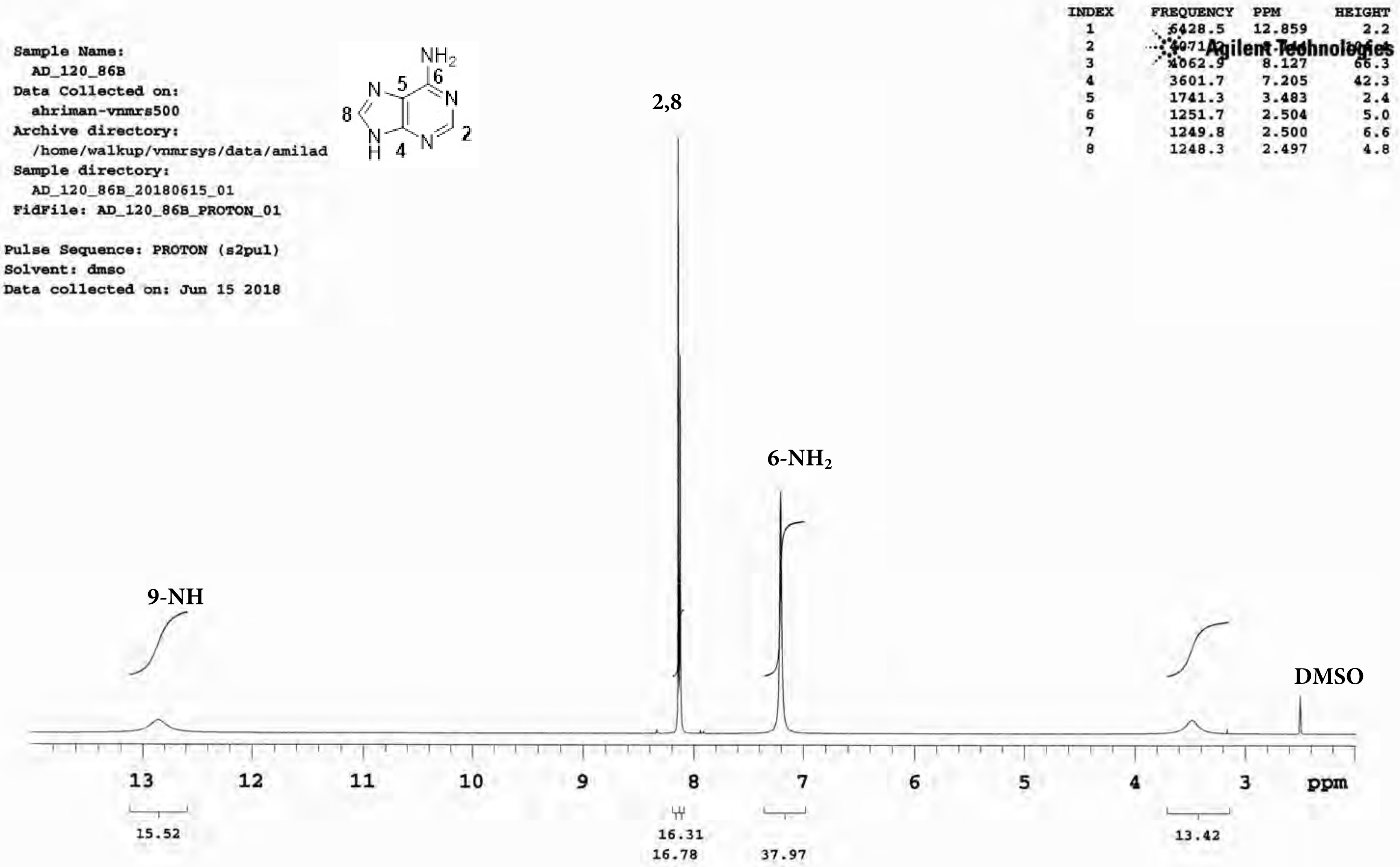
Sample Name:
AD_120_86B
Data Collected on:
ahriman-vnmrs500
Archive directory:
/home/walkup/vnmrsys/data/amilad
Sample directory:
AD_120_86B_20180615_01
FidFile: AD_120_86B_PROTON_01
Pulse Sequence: PROTON (s2pul)
Solvent: dmso
Data collected on: Jun 15 2018
NH2
6
5
N
N
8
2
N
N
H
4
INDEX FREQUENCY PPM HEIGHT
1 6428.5 12.859 2.2
2 [illegible] [illegible] [illegible]
3 4062.9 8.127 66.3
4 3601.7 7.205 42.3
5 1741.3 3.483 2.4
6 1251.7 2.504 5.0
7 1249.8 2.500 6.6
8 1248.3 2.497 4.8
Agilent Technologies
2,8
6-NH2
9-NH
DMSO
13
12
11
10
9
8
7
6
5
4
3
ppm
15.52
16.31
16.78
37.97
13.42

Figure J

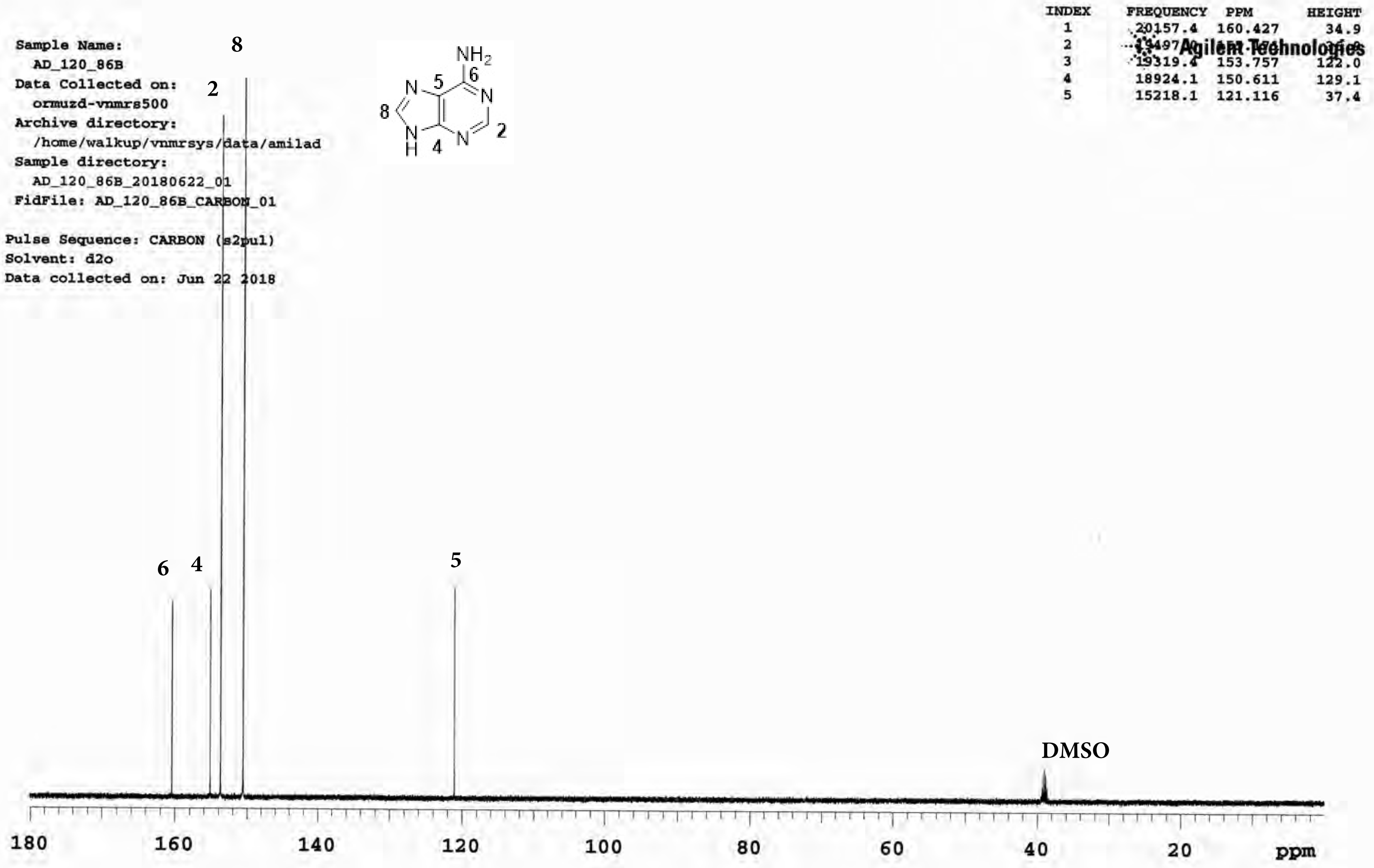
Sample Name:
AD_120_86B
Data Collected on:
ormuzd-vnmrs500
Archive directory:
/home/walkup/vnmrsys/data/amilad
Sample directory:
AD_120_86B_20180622_01
FidFile: AD_120_86B_CARBON_01
Pulse Sequence: CARBON (s2pul)
Solvent: d2o
Data collected on: Jun 22 2018
NH2
INDEX FREQUENCY PPM HEIGHT
1 20157.4 160.427 34.9
3 19319.4 153.757 122.0
4 18924.1 150.611 129.1
5 15218.1 121.116 37.4
Agilent Technologies
6
4
2
8
5
DMSO
180
160
140
120
100
80
60
40
20
ppm

Figure K

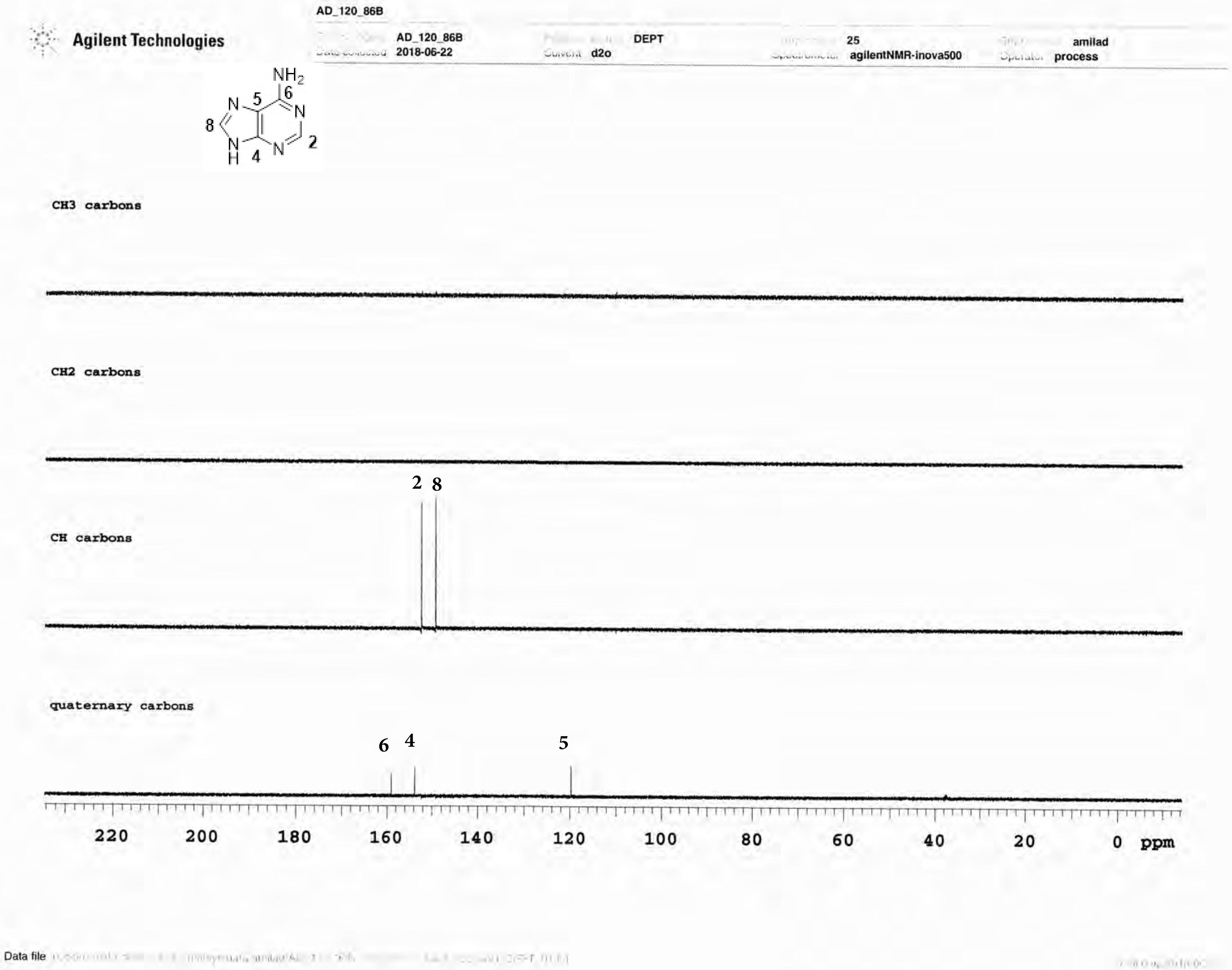
AD_120_86B
Agilent Technologies
AD_120_86B
2018-06-22
DEPT
d2o
25
agilentNMR-inova500
amilad
process
NH2
6
5
N
N
8
2
N
N
H
4
CH3 carbons
CH2 carbons
2 8
CH carbons
quaternary carbons
6 4
5
220
200
180
160
140
120
100
80
60
40
20
0 ppm
Data file

Figure L

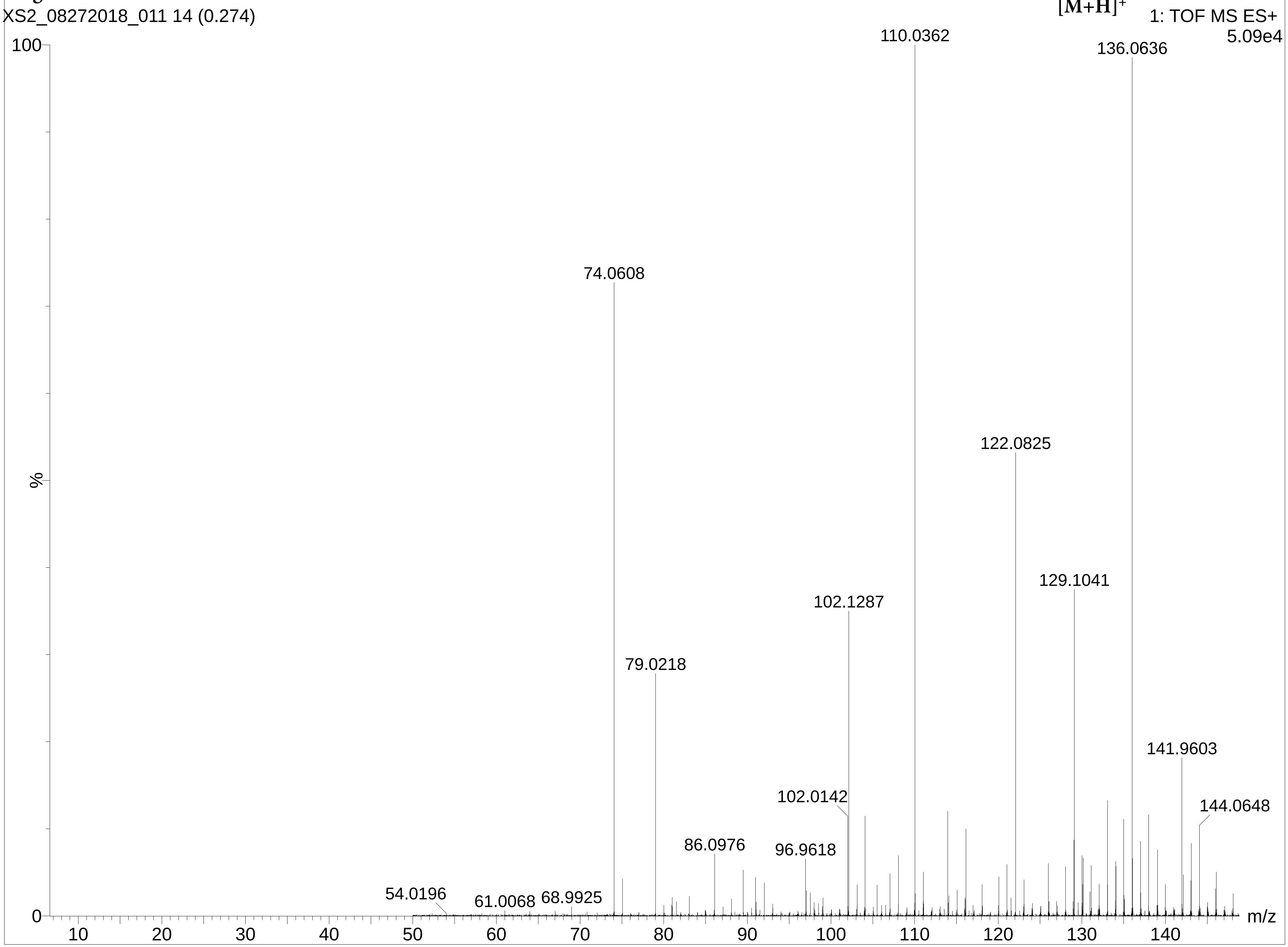
XS2_08272018_011 14 (0.274)
[M+H]+
1: TOF MS ES+
5.09e4
110.0362
136.0636
74.0608
122.0825
129.1041
102.1287
79.0218
141.9603
102.0142
144.0648
86.0976
96.9618
54.0196
61.0068
68.9925
100
%
0
m/z
10
20
30
40
50
60
70
80
90
100
110
120
130
140

Figure M

Sample Name:
AD_120_86C
Data Collected on:
ahriman-vnmrs500
Archive directory:
/home/walkup/vnmrsys/data/amilad
Sample directory:
AD_120_86C_20180615_01
FidFile: AD_120_86C_PROTON_01

Pulse Sequence: PROTON (s2pul)
Solvent: dmso
Data collected on: Jun 15 2018

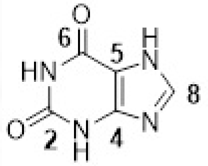

INDEX	FREQUENCY	PPM	HEIGHT
1	5434.7	10.871	1.2
2	[illegible]	[illegible]	[illegible]
3	1748.1	3.497	3.2
4	1683.1	3.367	15.1
5	1251.2	2.503	22.8
6	1249.8	2.500	28.4
7	1247.8	2.496	21.7
8	609.6	1.219	2.8

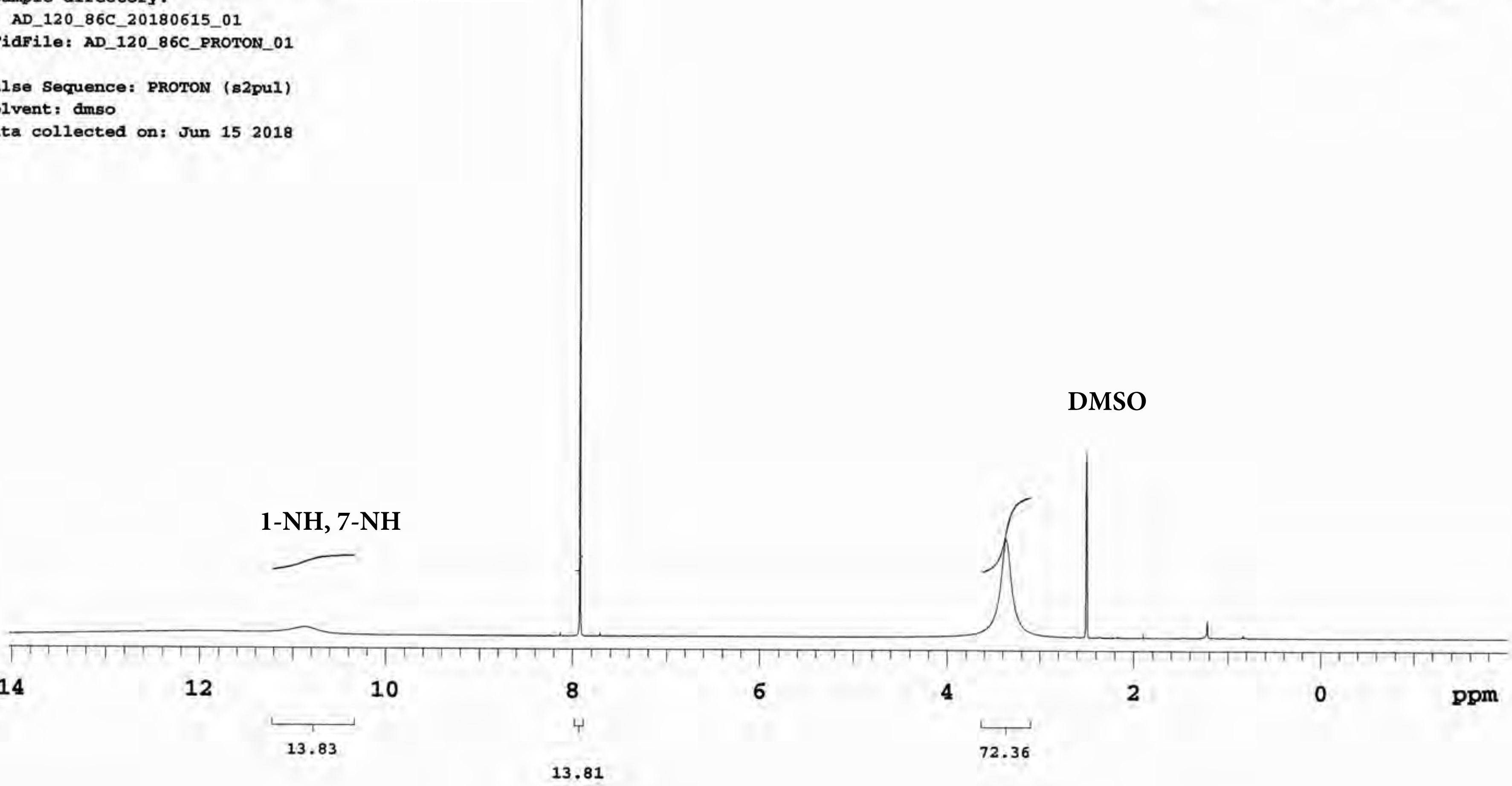

Figure N

Sample Name:
AD_120_86C
Data Collected on:
ormuzd-vnmrs500
Archive directory:
/home/walkup/vnmrsys/data/amilad
Sample directory:
AD_120_86C_20180623_01
FidFile: AD_120_86C_CARBON_01

Pulse Sequence: CARBON (s2pul)
Solvent: d2o
Data collected on: Jun 23 2018

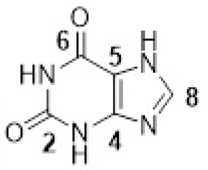

INDEX	FREQUENCY	PPM	HEIGHT
1	20636.9	164.243	91.1
2	[illegible]	[illegible]	[illegible]
3	20130.0	160.209	73.0
4	19033.8	151.484	111.5
5	19030.9	151.461	97.4
6	14675.2	116.796	79.4

Agilent Technologies

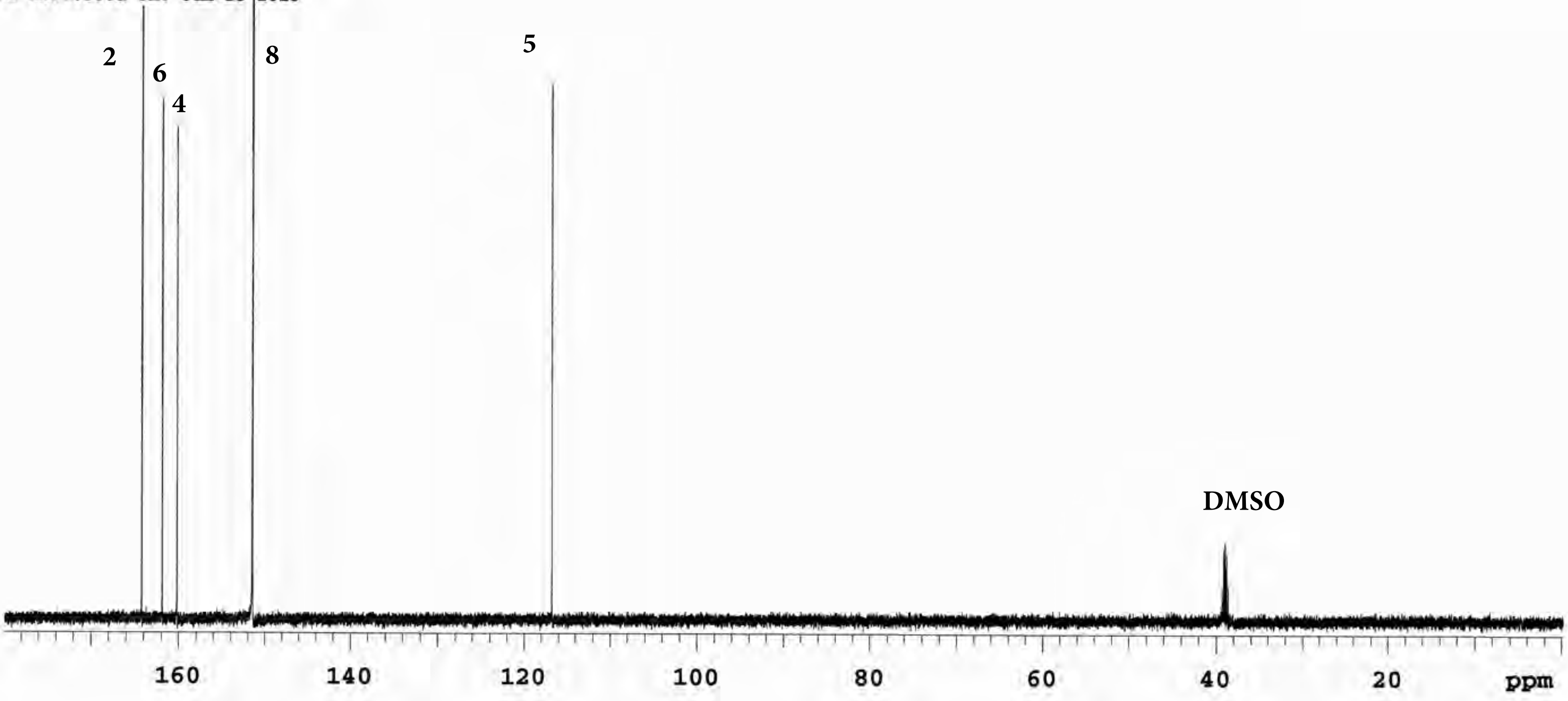

Figure O

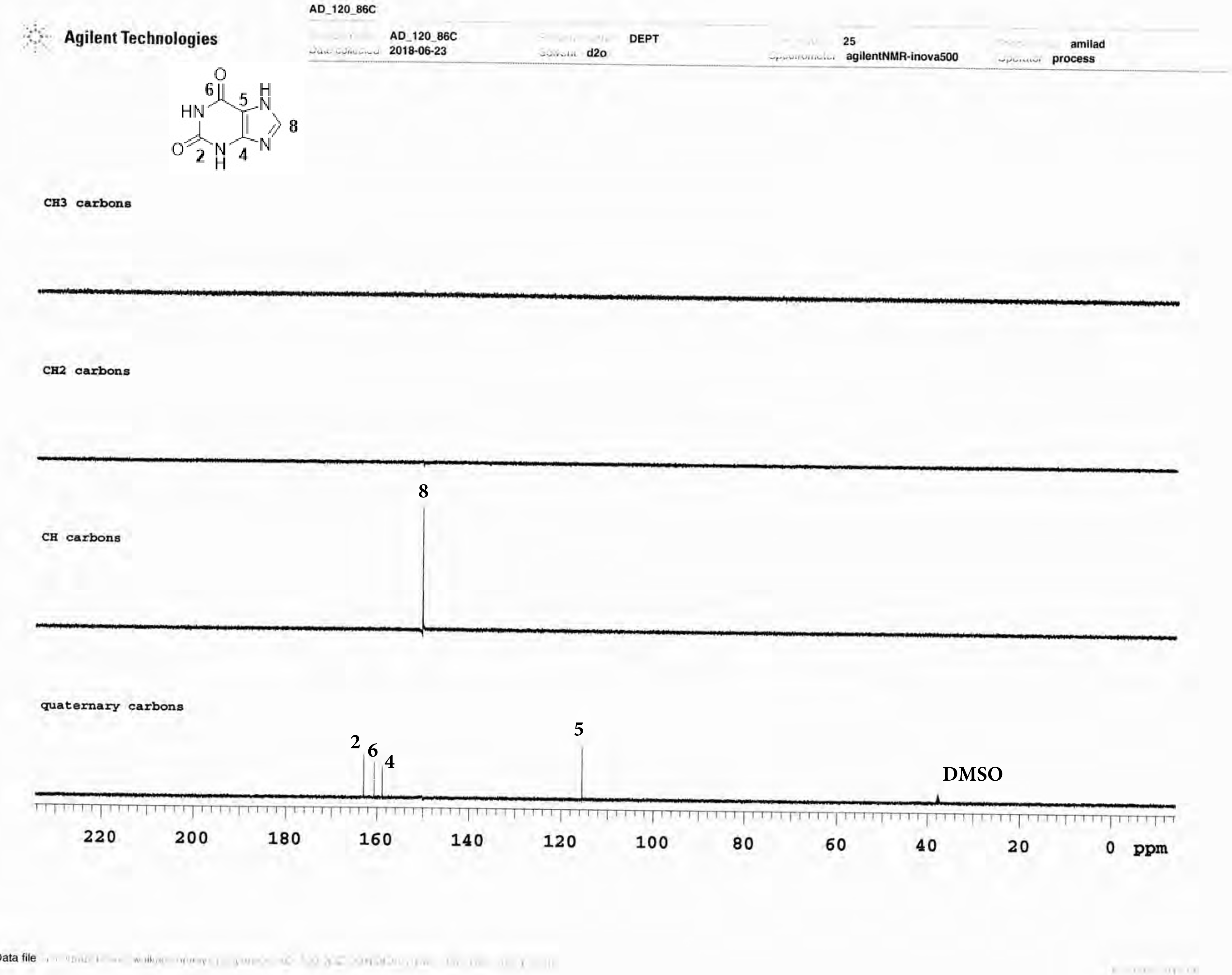

AD_120_86C
Agilent Technologies
AD_120_86C
2018-06-23
DEPT
d2o
25
agilentNMR-inova500
amilad
process
CH3 carbons
CH2 carbons
CH carbons
quaternary carbons
8
2
6
4
5
DMSO
220
200
180
160
140
120
100
80
60
40
20
0
ppm
Data file

Figure P

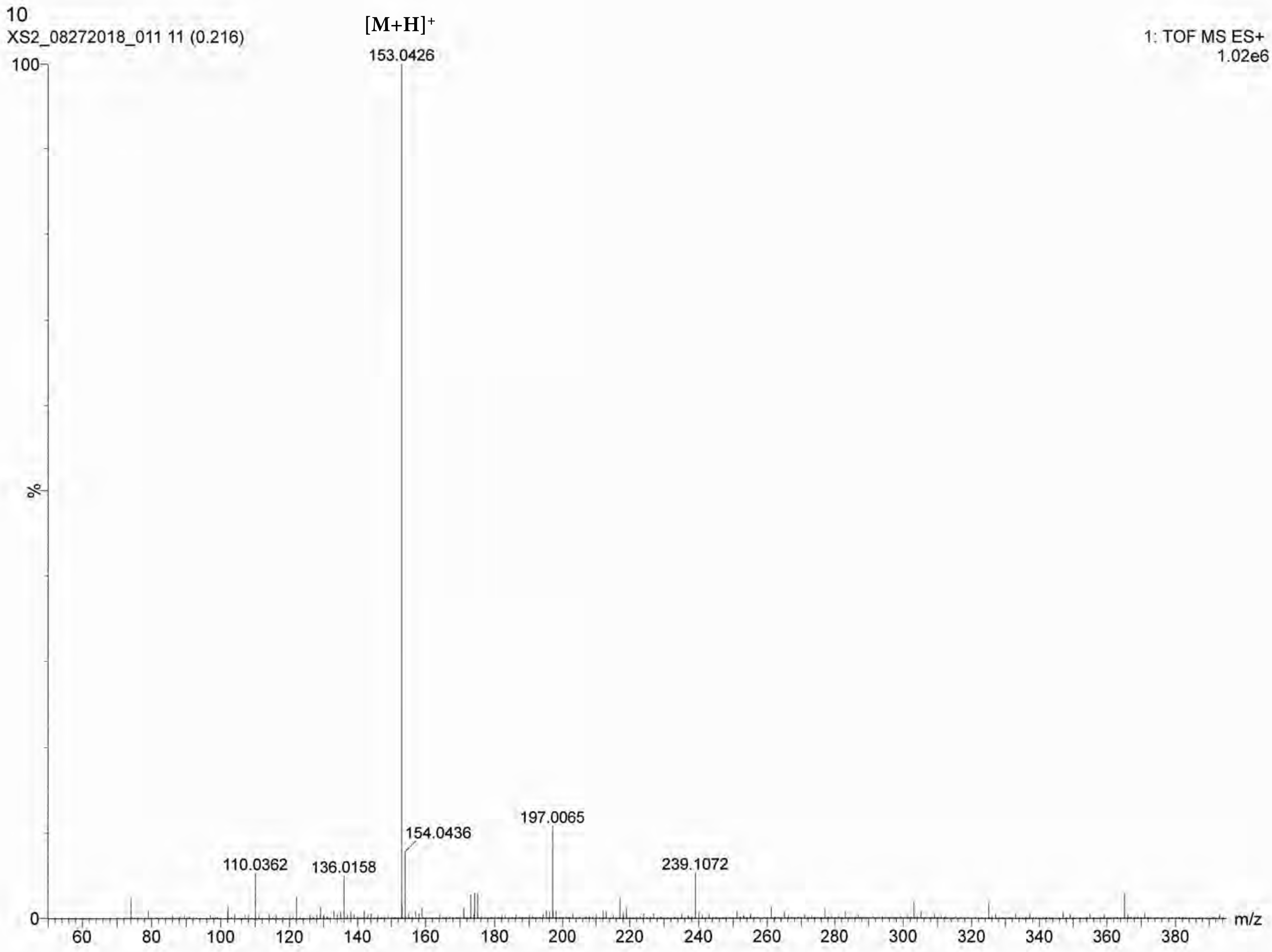

10
XS2_08272018_011 11 (0.216)
[M+H]+
153.0426
1: TOF MS ES+
1.02e6
100
%
0
154.0436
197.0065
110.0362
136.0158
239.1072
60
80
100
120
140
160
180
200
220
240
260
280
300
320
340
360
380
m/z

Figure Q

Sample Name:
AD_120_82G
Data Collected on:
ormuzd-vnmrs500
Archive directory:
/home/walkup/vnmrsys/data/amilad
Sample directory:
AD_120_82G_20180510_02
FidFile: AD_120_82G_PROTON_01

Pulse Sequence: PROTON (s2pul)
Solvent: d2o
Data collected on: May 10 2018

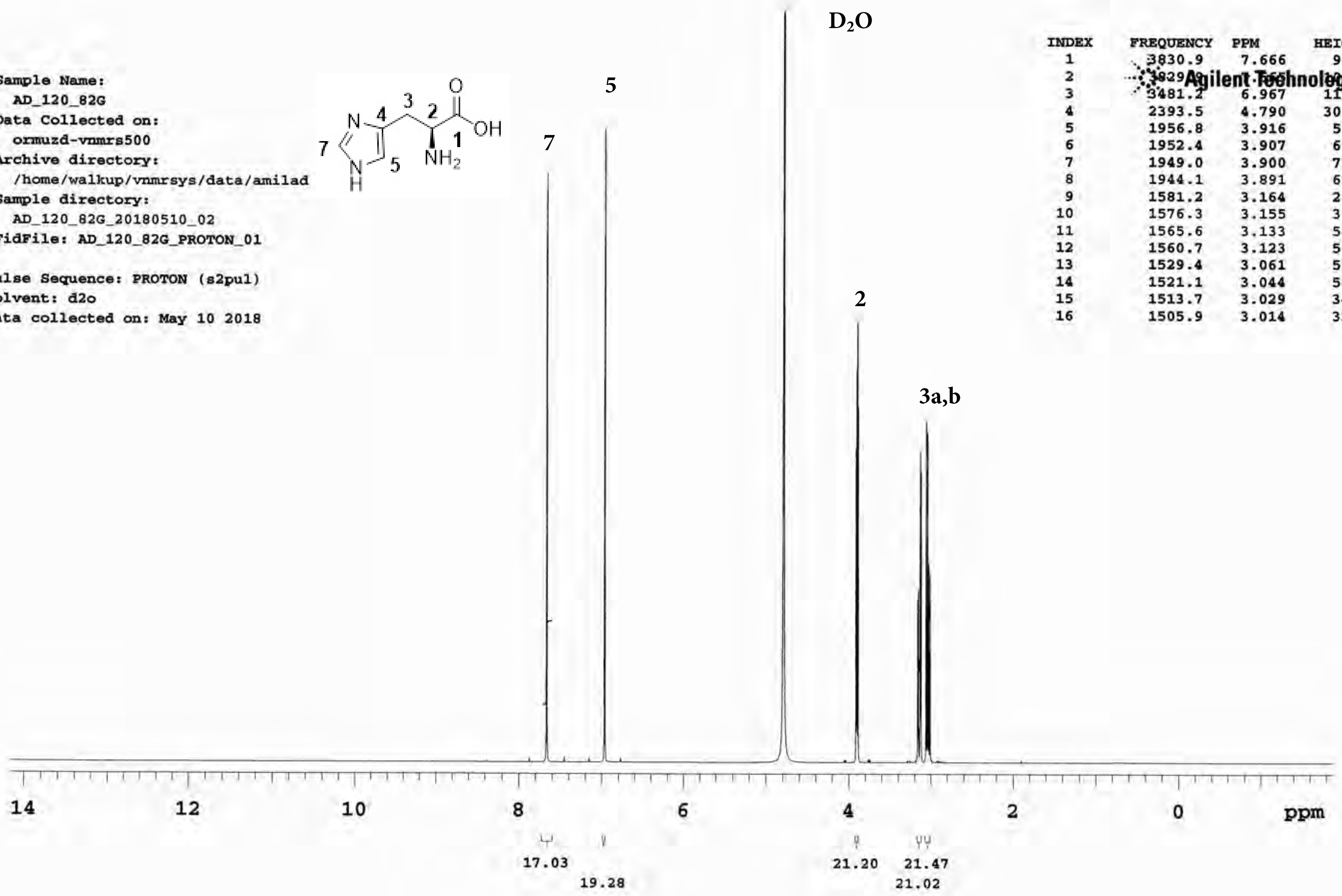

INDEX	FREQUENCY	PPM	HEIGHT
1	3830.9	7.666	98.0
2	[illegible]	[illegible]	[illegible]
3	3481.2	6.967	111.3
4	2393.5	4.790	300.0
5	1956.8	3.916	55.4
6	1952.4	3.907	69.0
7	1949.0	3.900	77.4
8	1944.1	3.891	62.4
9	1581.2	3.164	28.6
10	1576.3	3.155	30.3
11	1565.6	3.133	54.7
12	1560.7	3.123	50.9
13	1529.4	3.061	59.9
14	1521.1	3.044	58.0
15	1513.7	3.029	34.8
16	1505.9	3.014	32.9

Figure R

Sample Name:
AD_120_82G
Data Collected on:
ormuzd-vnmrs500
Archive directory:
/home/walkup/vnmrsys/data/amilad
Sample directory:
AD_120_82G_20180510_01
FidFile: AD_120_82G_CARBON_01

Pulse Sequence: CARBON (s2pul)
Solvent: d2o
Data collected on: May 10 2018

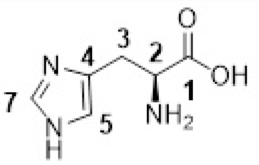

INDEX	FREQUENCY	PPM	HEIGHT
1	21855.1	173.938	30.7
2	[illegible]	[illegible]	[illegible]
3	16601.3	132.125	12.4
4	14652.0	116.611	30.1
5	6872.9	54.699	108.7
6	3532.2	28.111	92.1

Agilent Technologies

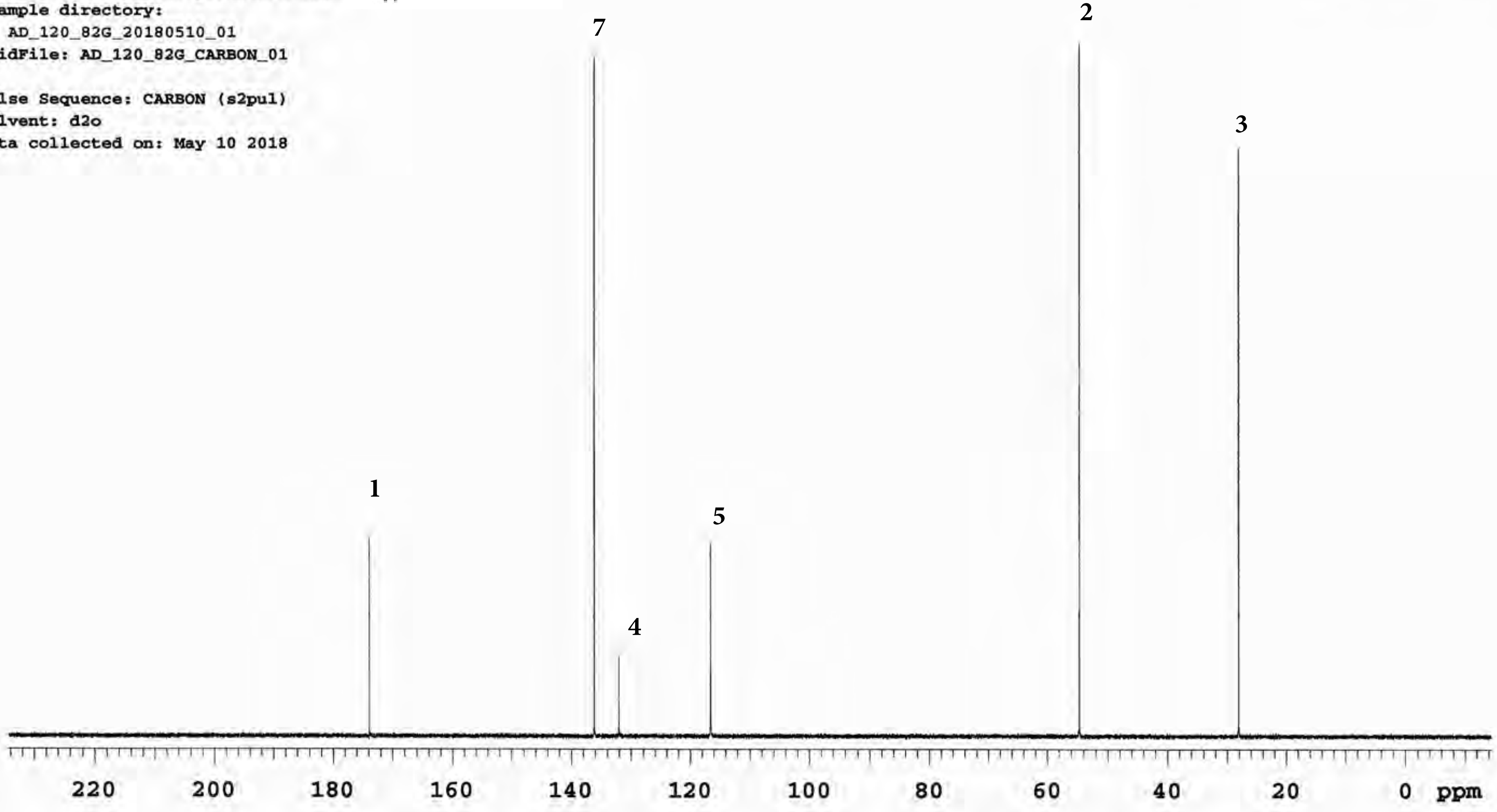

Figure S

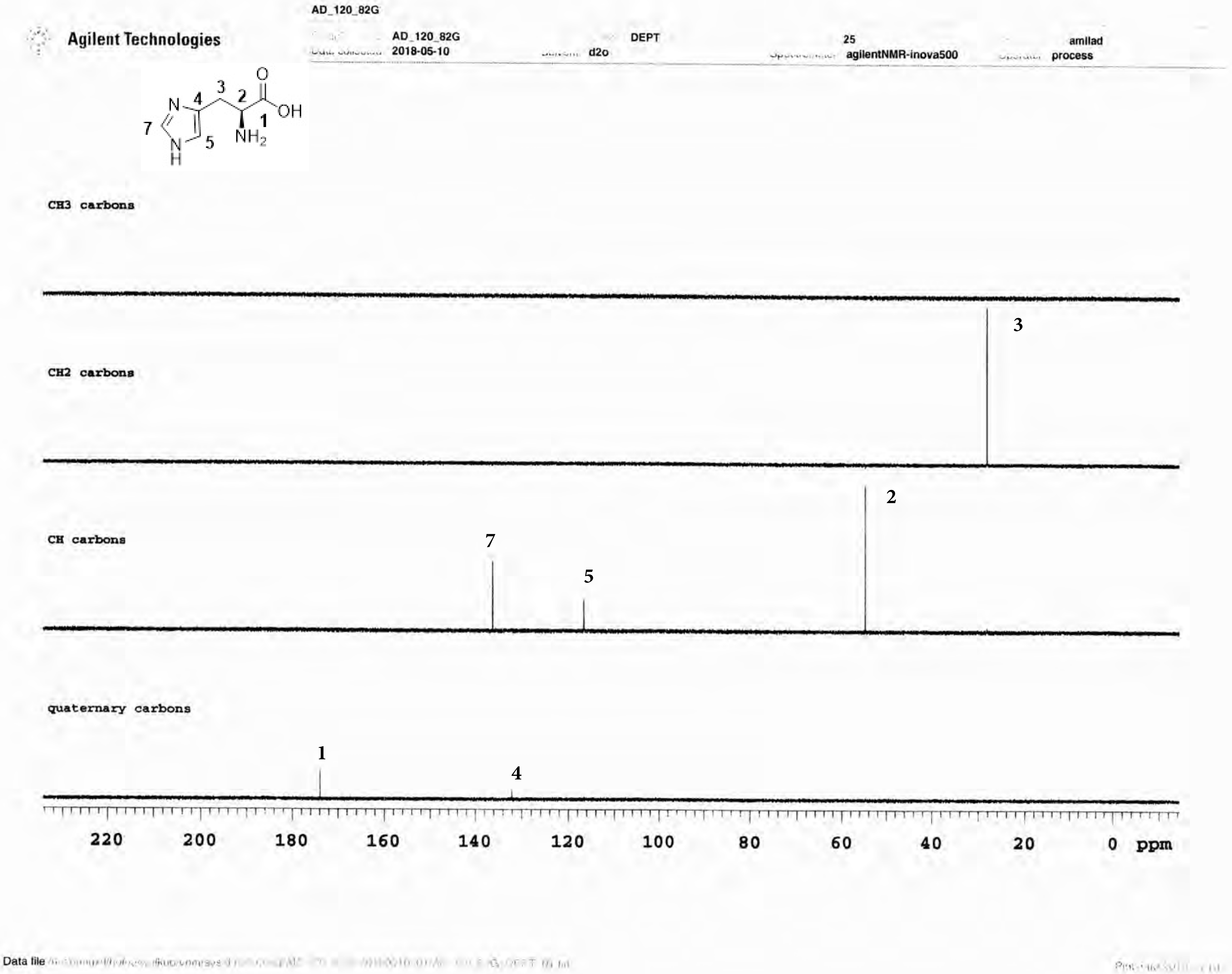
AD_120_82G
Agilent Technologies
AD_120_82G
2018-05-10
DEPT
d2o
25
agilentNMR-inova500
amilad
process
N
7
N
H
5
4
3
2
O
OH
1
NH2
CH3 carbons
CH2 carbons
3
CH carbons
7
5
2
quaternary carbons
1
4
220
200
180
160
140
120
100
80
60
40
20
0 ppm
Data file

Figure T

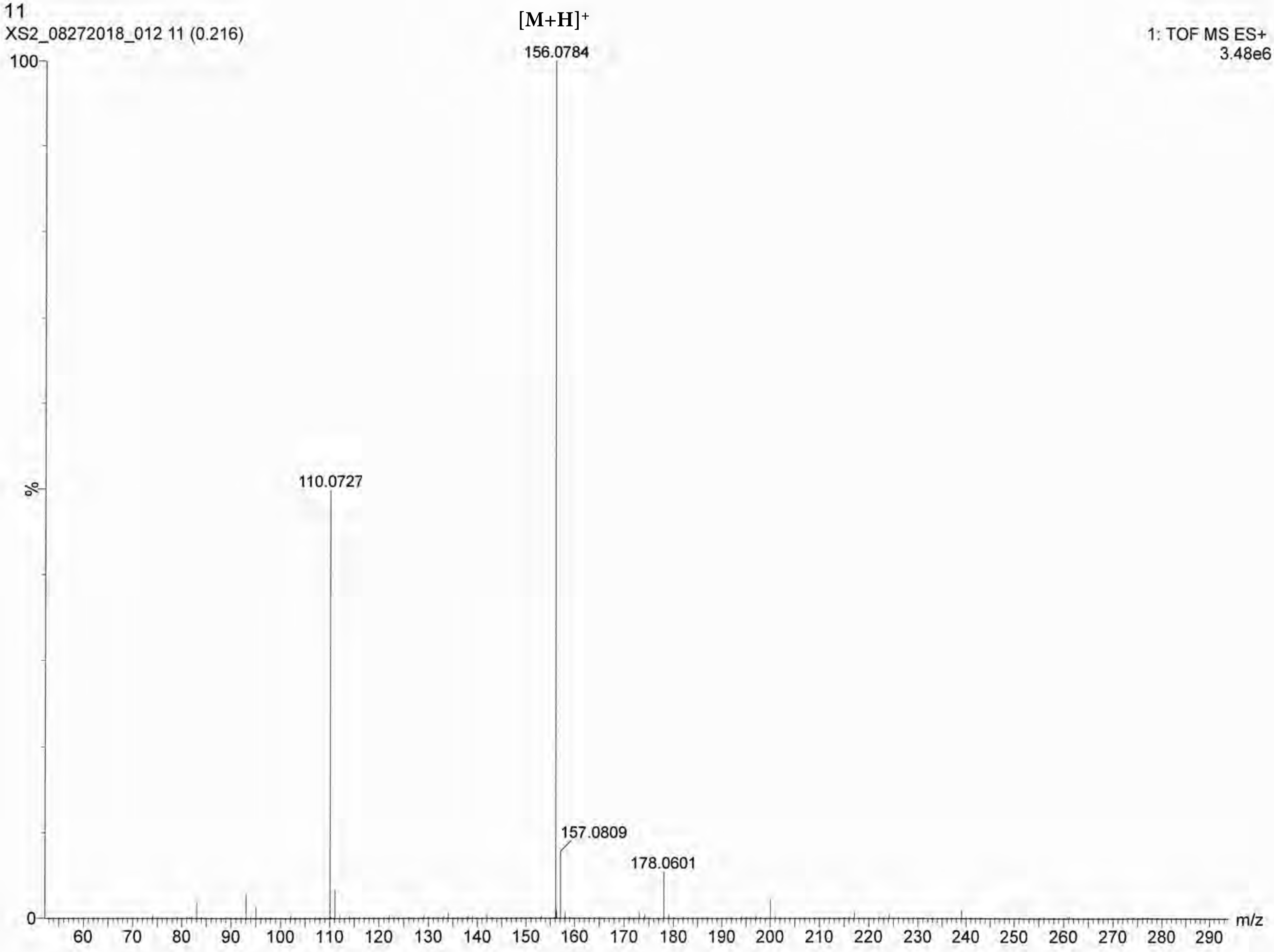

11
XS2_08272018_012 11 (0.216)
[M+H]+
156.0784
1: TOF MS ES+
3.48e6
100
%
110.0727
157.0809
178.0601
0
60
70
80
90
100
110
120
130
140
150
160
170
180
190
200
210
220
230
240
250
260
270
280
290
m/z

Figure U

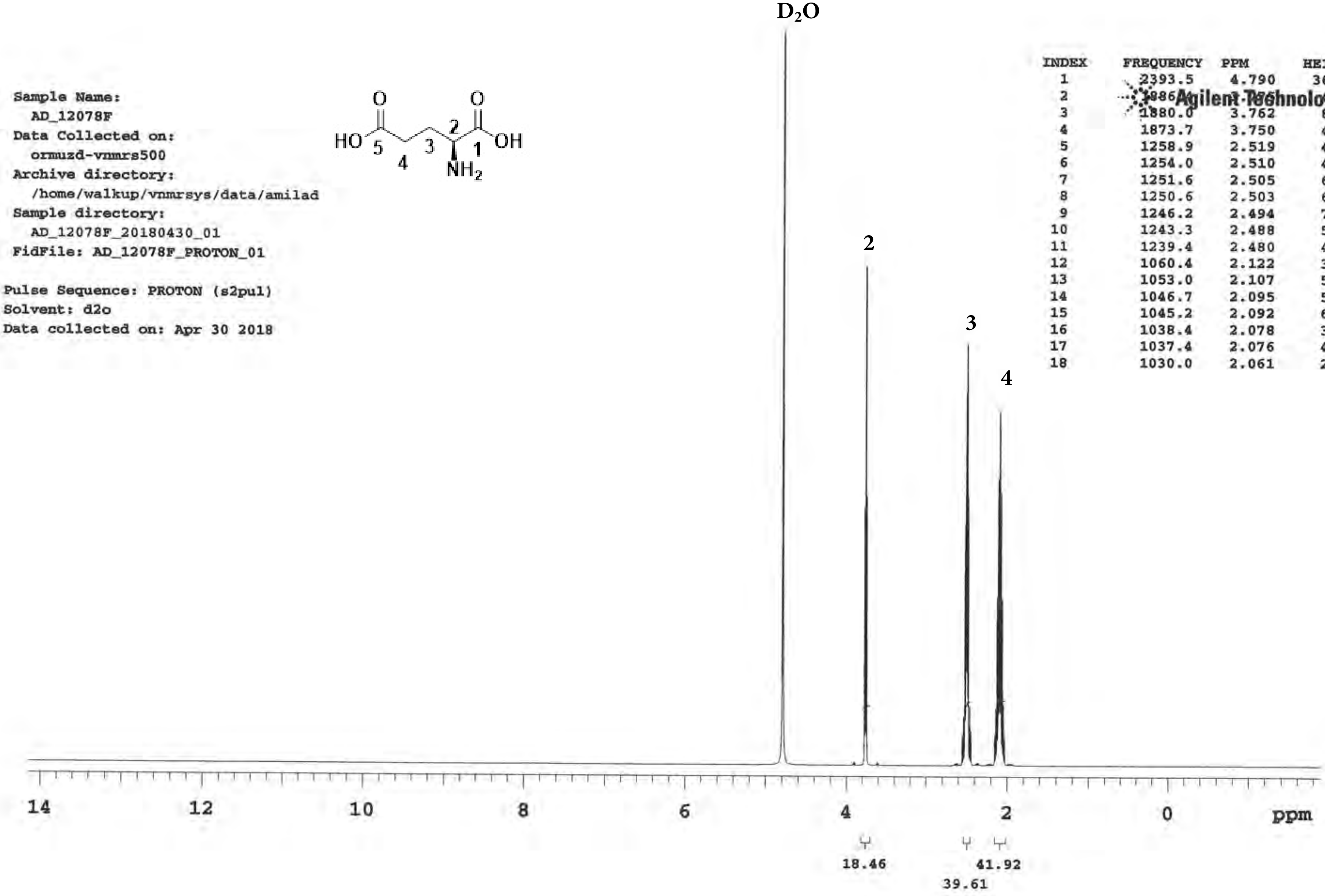

INDEX	FREQUENCY	PPM	HEIGHT
1	2393.5	4.790	360.2
2	[illegible]	[illegible]	[illegible]
3	1880.0	3.762	[illegible]
4	1873.7	3.750	48.3
5	1258.9	2.519	43.3
6	1254.0	2.510	48.6
7	1251.6	2.505	61.6
8	1250.6	2.503	67.1
9	1246.2	2.494	75.9
10	1243.3	2.488	52.0
11	1239.4	2.480	48.3
12	1060.4	2.122	30.2
13	1053.0	2.107	51.5
14	1046.7	2.095	51.5
15	1045.2	2.092	63.8
16	1038.4	2.078	38.9
17	1037.4	2.076	49.0
18	1030.0	2.061	29.9

Figure V

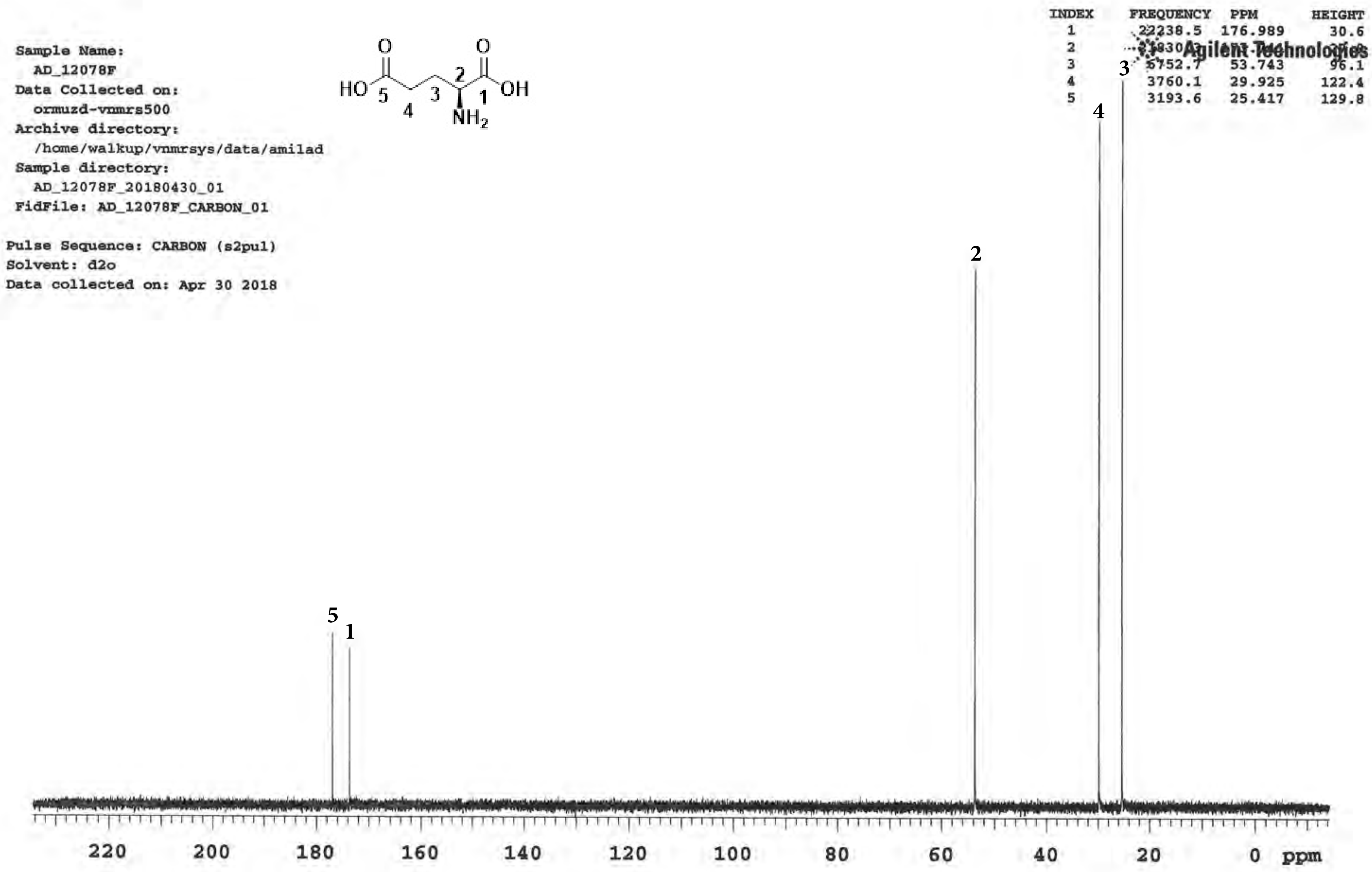
Sample Name:
AD_12078F
Data Collected on:
ormuzd-vnmrs500
Archive directory:
/home/walkup/vnmrsys/data/amilad
Sample directory:
AD_12078F_20180430_01
FidFile: AD_12078F_CARBON_01
Pulse Sequence: CARBON (s2pul)
Solvent: d2o
Data collected on: Apr 30 2018
HO 5 4 3 2 1 OH
O O
NH2
INDEX FREQUENCY PPM HEIGHT
1 22238.5 176.989 30.6
2 [illegible] [illegible] [illegible]
3 6752.7 53.743 96.1
4 3760.1 29.925 122.4
5 3193.6 25.417 129.8
Agilent Technologies
5 1 2 4 3
220 200 180 160 140 120 100 80 60 40 20 0 ppm

Figure W

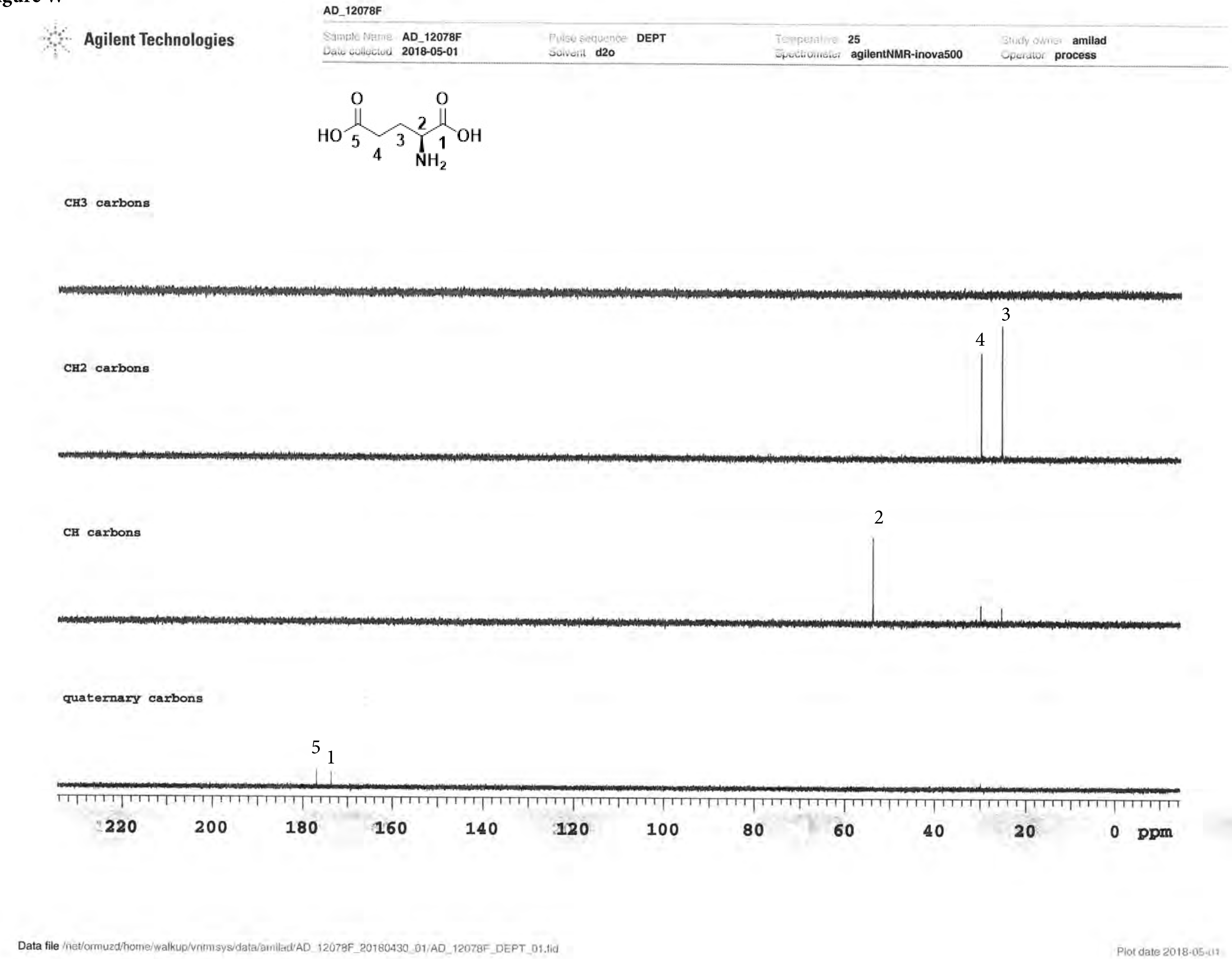
Agilent Technologies
AD_12078F
Sample Name AD_12078F
Date collected 2018-05-01
Pulse sequence DEPT
Solvent d2o
Temperature 25
Spectrometer agilentNMR-inova500
Study owner amilad
Operator process
HO
OH
O
O
NH2
1
2
3
4
5
CH3 carbons
CH2 carbons
CH carbons
quaternary carbons
220
200
180
160
140
120
100
80
60
40
20
0
ppm
Data file /net/ormuzd/home/walkup/vnmrsys/data/amilad/AD_12078F_20180430_01/AD_12078F_DEPT_01.fid
Plot date 2018-05-01

Figure X

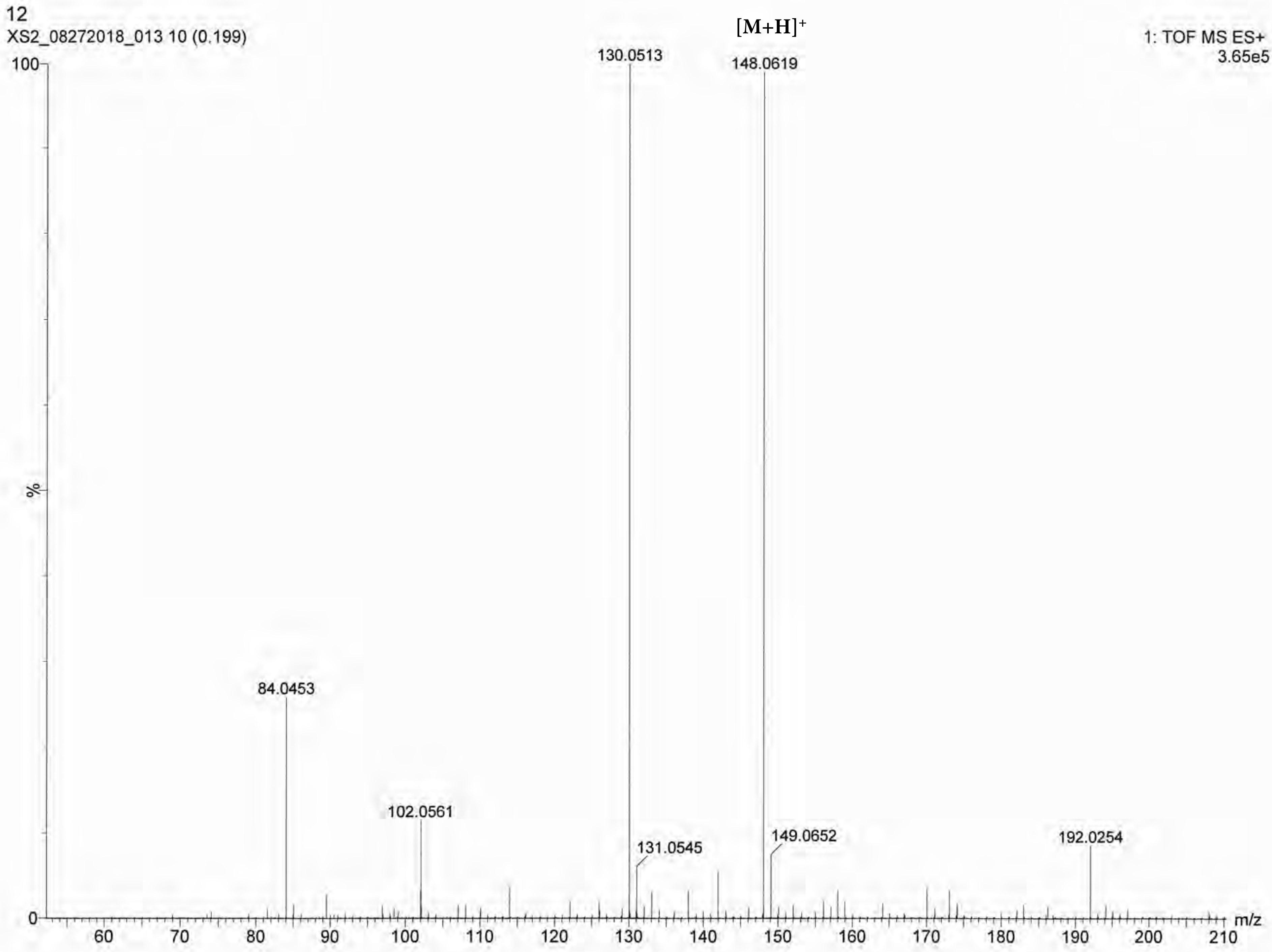

12
XS2_08272018_013 10 (0.199)
1: TOF MS ES+
3.65e5
[M+H]+
130.0513
148.0619
84.0453
102.0561
131.0545
149.0652
192.0254
100
%
0
60
70
80
90
100
110
120
130
140
150
160
170
180
190
200
210
m/z
