# Supplementary material for: Nitrogenous compounds characterized in the deterrent skin extract of migratory adult sea lamprey from the Great Lakes region
Source: PLoS One. 2019 May 23;14(5):e0217417. doi: 10.1371/journal.pone.0217417 (PMC6532902; doi:10.1371/journal.pone.0217417)
Supplement: S4 File — (PDF) [file pone.0217417.s004.pdf]

Nitrogenous Compounds Characterized in the deterrent Skin extract of Migratory Adult Sea Lamprey from the Great Lakes Region

Amila A. Dissanayake,[1] C. Michael Wagner,[2] Muraleedharan G. Nair,[1*]

[1] Department of Horticulture, Michigan State University, East Lansing, Michigan, United States of America

[2] Department of Fisheries and Wildlife, Michigan State University, East Lansing, Michigan, United States of America

Supporting Information

Figure A. ^{1}H NMR spectrum of phenylalanine in D_2O
Figure B. ^{13}C NMR spectrum of phenylalanine in D_2O
Figure C. DEPT spectrum of phenylalanine in D_2O
Figure D. HRMS spectrum of phenylalanine (positive ion mode)
Figure E. ^{1}H NMR spectrum of tryptophan in D_2O
Figure F. ^{13}C NMR spectrum of tryptophan in D_2O
Figure G. DEPT spectrum of tryptophan in D_2O
Figure H. HRMS spectrum of tryptophan (positive ion mode)
Figure I. ^{1}H NMR spectrum of threonine in D_2O
Figure J. ^{13}C NMR spectrum of threonine in D_2O
Figure K. DEPT spectrum of threonine in D_2O
Figure L. HRMS spectrum of threonine (positive ion mode)
Figure M. ^{1}H NMR spectrum of asparagine in D_2O
Figure N. ^{13}C NMR spectrum of asparagine in D_2O
Figure O. DEPT spectrum of asparagine in D_2O
Figure P. HRMS spectrum of asparagine (positive ion mode)
Figure Q. ^{1}H NMR spectrum of methionine in D_2O
Figure R. ^{13}C NMR spectrum of methionine in D_2O
Figure S. DEPT spectrum of methionine in D_2O
Figure T. HRMS spectrum of methionine (positive ion mode)
Figure U. ^{1}H NMR spectrum of cysteine in D_2O
Figure V. ^{13}C NMR spectrum of cysteine in D_2O
Figure W. DEPT spectrum of cysteine in D_2O
Figure X. HRMS spectrum of cysteine (positive ion mode)

Figure A

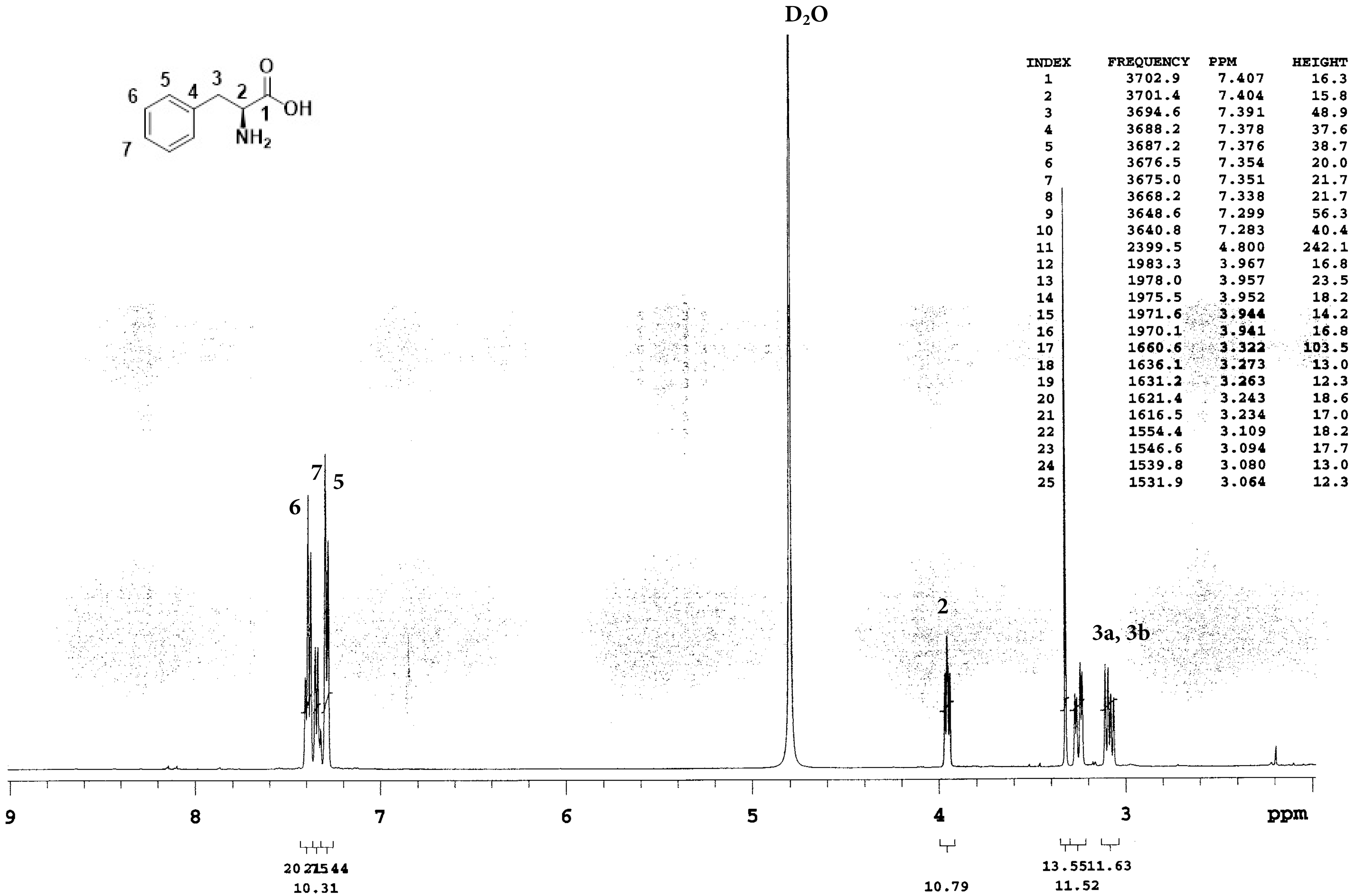

INDEX	FREQUENCY	PPM	HEIGHT
1	3702.9	7.407	16.3
2	3701.4	7.404	15.8
3	3694.6	7.391	48.9
4	3688.2	7.378	37.6
5	3687.2	7.376	38.7
6	3676.5	7.354	20.0
7	3675.0	7.351	21.7
8	3668.2	7.338	21.7
9	3648.6	7.299	56.3
10	3640.8	7.283	40.4
11	2399.5	4.800	242.1
12	1983.3	3.967	16.8
13	1978.0	3.957	23.5
14	1975.5	3.952	18.2
15	1971.6	3.944	14.2
16	1970.1	3.941	16.8
17	1660.6	3.322	103.5
18	1636.1	3.273	13.0
19	1631.2	3.263	12.3
20	1621.4	3.243	18.6
21	1616.5	3.234	17.0
22	1554.4	3.109	18.2
23	1546.6	3.094	17.7
24	1539.8	3.080	13.0
25	1531.9	3.064	12.3

Figure B

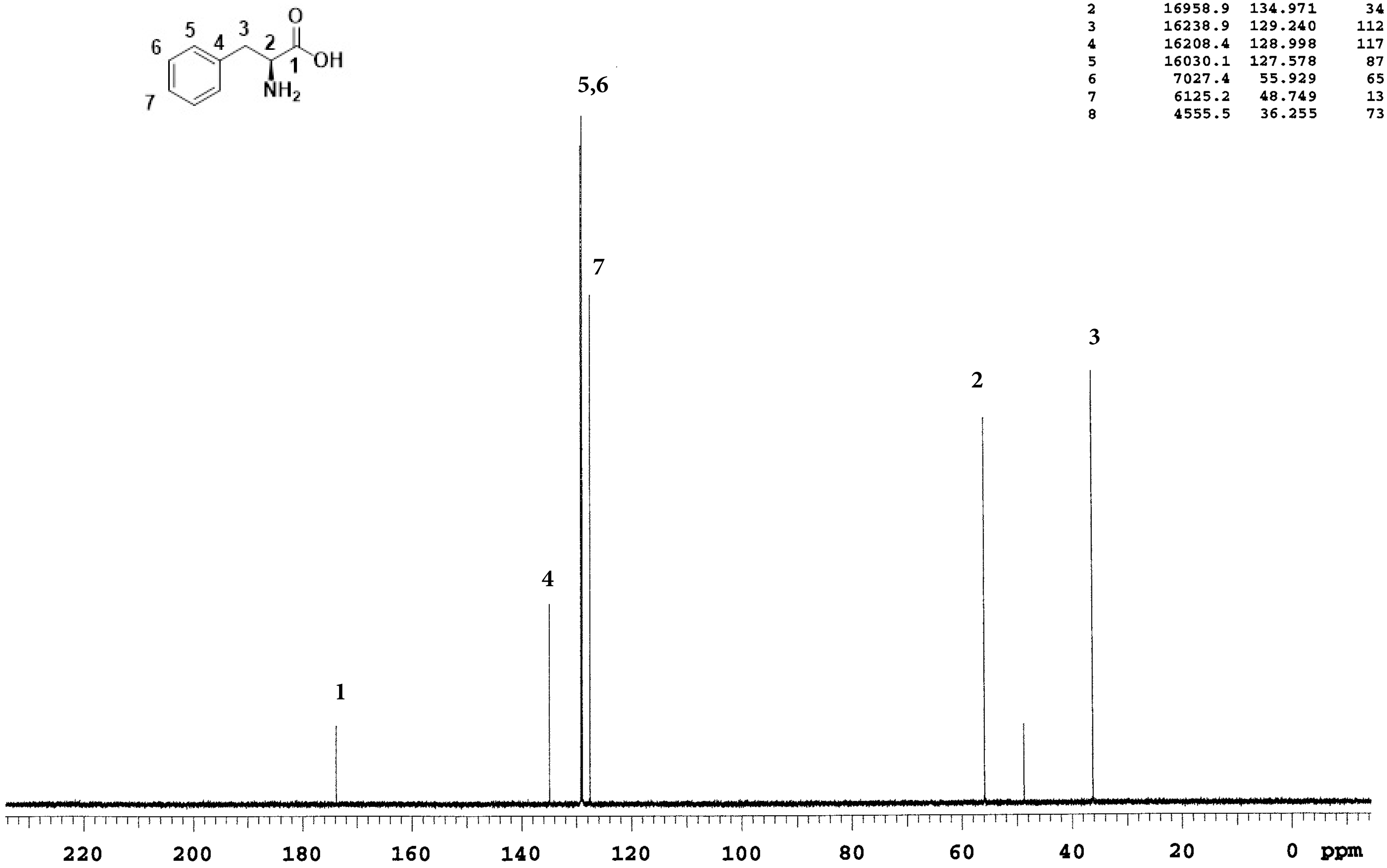

INDEX	FREQUENCY	PPM	HEIGHT
1	21846.5	173.870	13.4
2	16958.9	134.971	34.2
3	16238.9	129.240	112.8
4	16208.4	128.998	117.9
5	16030.1	127.578	87.3
6	7027.4	55.929	65.9
7	6125.2	48.749	13.5
8	4555.5	36.255	73.9

Figure C

Agilent Technologies

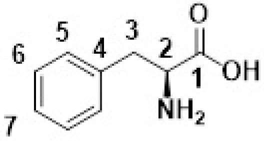

AD-120_65A

Sample Name	AD-120_65A	Pulse sequence	DEPT	Temperature	25	Study owner	amilad
Date collected	2018-01-26	Solvent	d2o	Spectrometer	agilentNMR-inova500	Operator	process

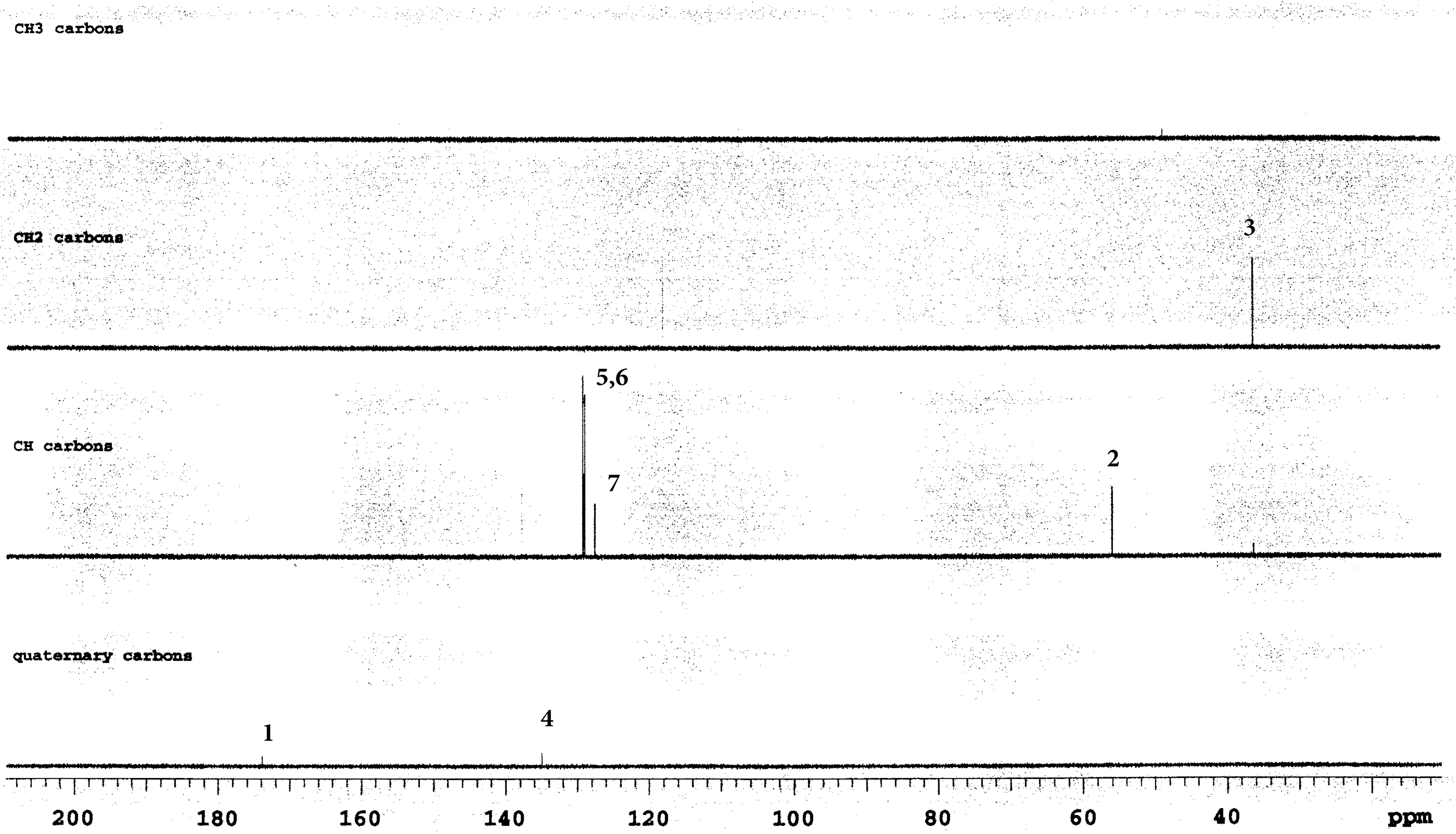

Data file /net/ormuzd/home/walkup/vnmrsys/data/amilad/AD-120_65A_20180125_01/AD-120_65A_DEPT_01.fid

Plot date 2018-01-26

Figure D

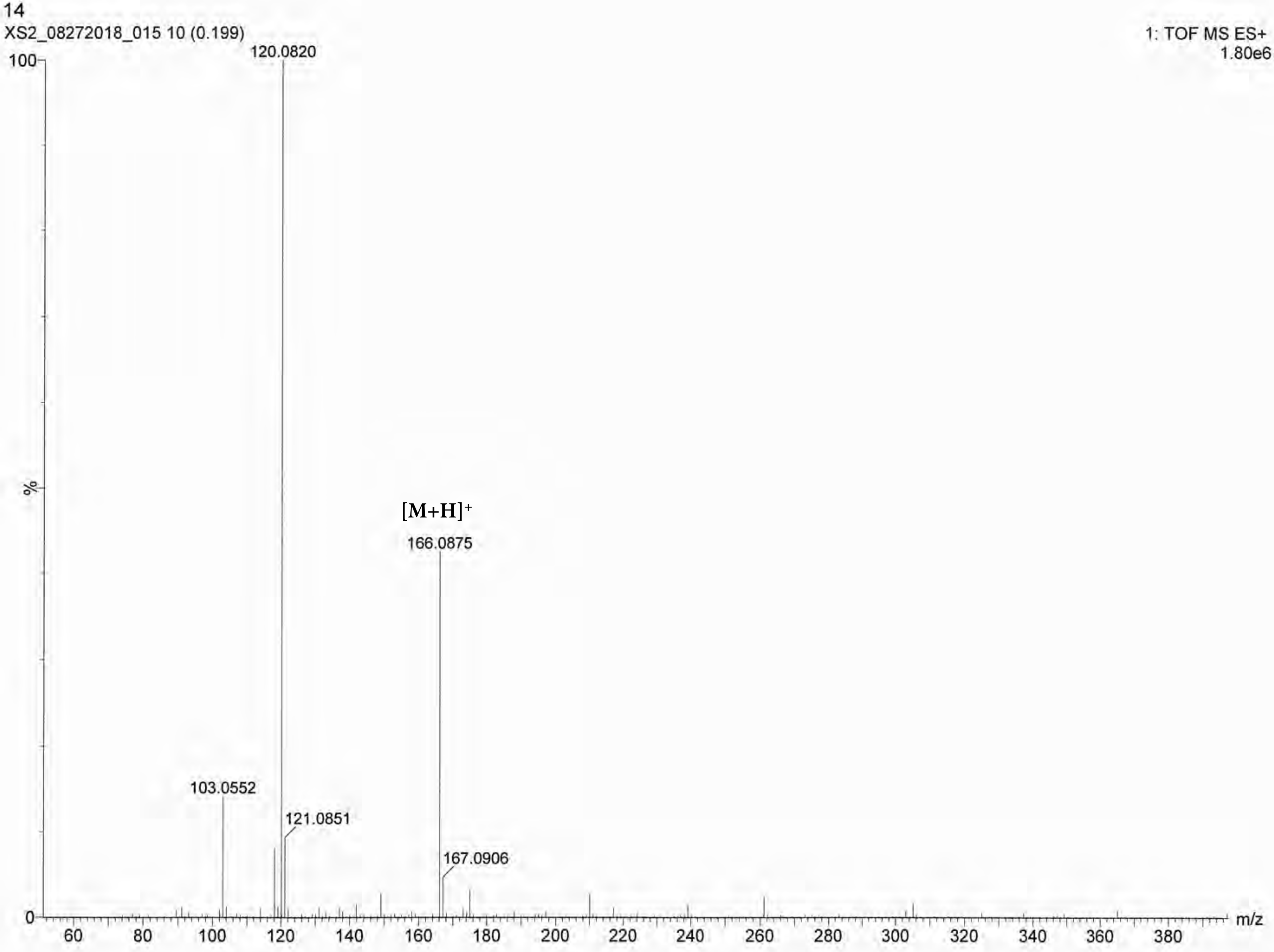

14
XS2_08272018_015 10 (0.199)
1: TOF MS ES+
1.80e6
100
%
0
120.0820
[M+H]+
166.0875
103.0552
121.0851
167.0906
60
80
100
120
140
160
180
200
220
240
260
280
300
320
340
360
380
m/z

Figure E

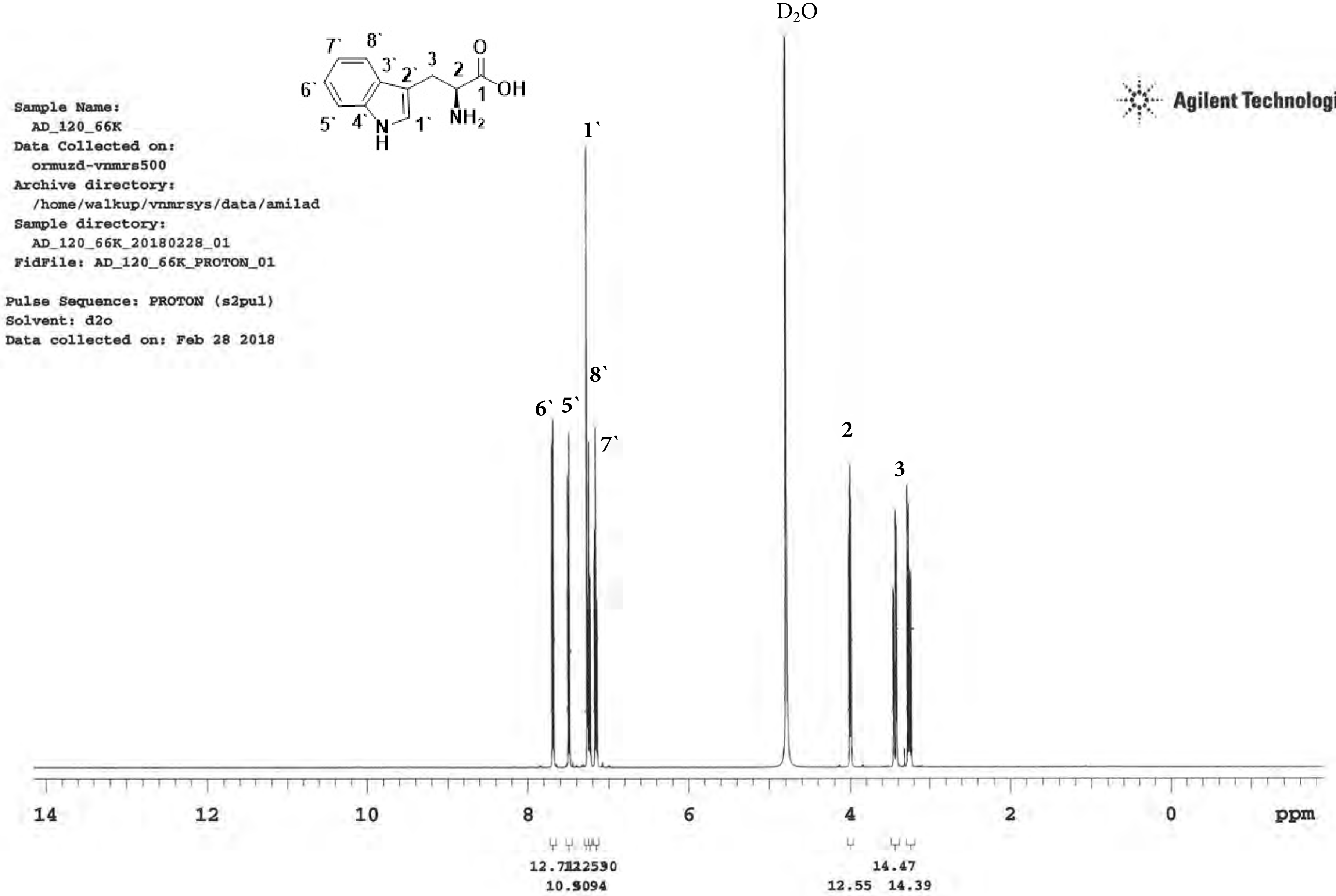
D$_2$O
Sample Name:
AD_120_66K
Data Collected on:
ormuzd-vnmrs500
Archive directory:
/home/walkup/vnmrsys/data/amilad
Sample directory:
AD_120_66K_20180228_01
FidFile: AD_120_66K_PROTON_01
Pulse Sequence: PROTON (s2pul)
Solvent: d2o
Data collected on: Feb 28 2018
Agilent Technologies
1`
8`
6`
5`
7`
2
3
14
12
10
8
6
4
2
0
ppm
14.47
12.55
14.39

Figure F

Sample Name:
AD_120_66K
Data Collected on:
ormuzd-vnmrs500
Archive directory:
/home/walkup/vnmrsys/data/amilad
Sample directory:
AD_120_66K_20180228_01
FidFile: AD_120_66K_CARBON_01

Pulse Sequence: CARBON (s2pul)
Solvent: d2o
Data collected on: Feb 28 2018

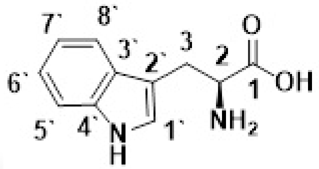

INDEX	FREQUENCY	PPM	HEIGHT
1	21918.1	174.439	47.2
2	[illegible]	[illegible]	[illegible]
3	15896.6	126.516	37.5
4	15693.4	124.899	98.6
5	15330.1	122.007	109.0
6	14994.4	119.336	103.1
7	14867.5	118.326	76.8
8	14050.2	111.821	67.7
9	13488.5	107.351	45.8
10	6901.5	54.927	71.2
11	3302.3	26.282	102.8

Agilent Technologies

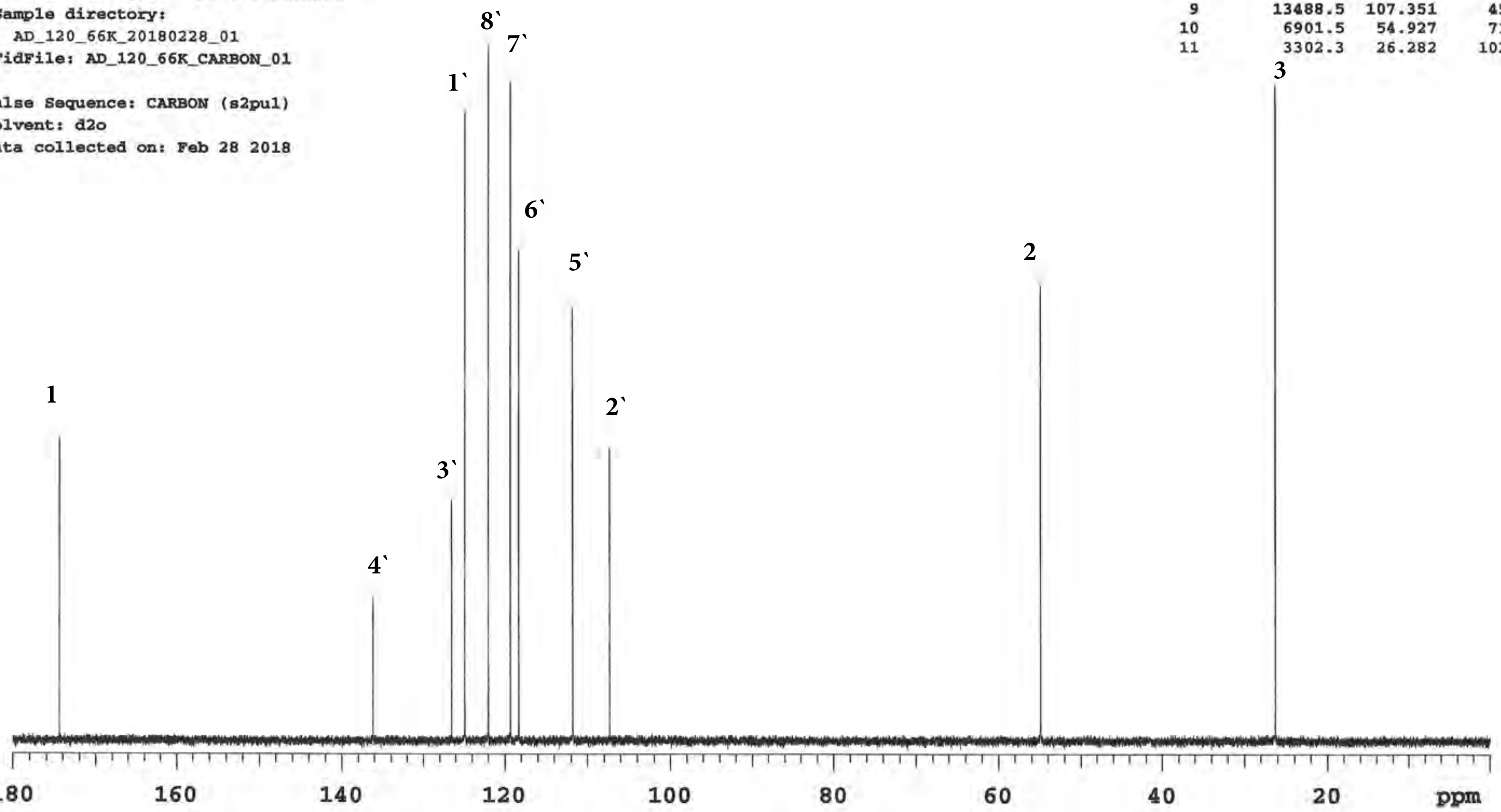

Figure G

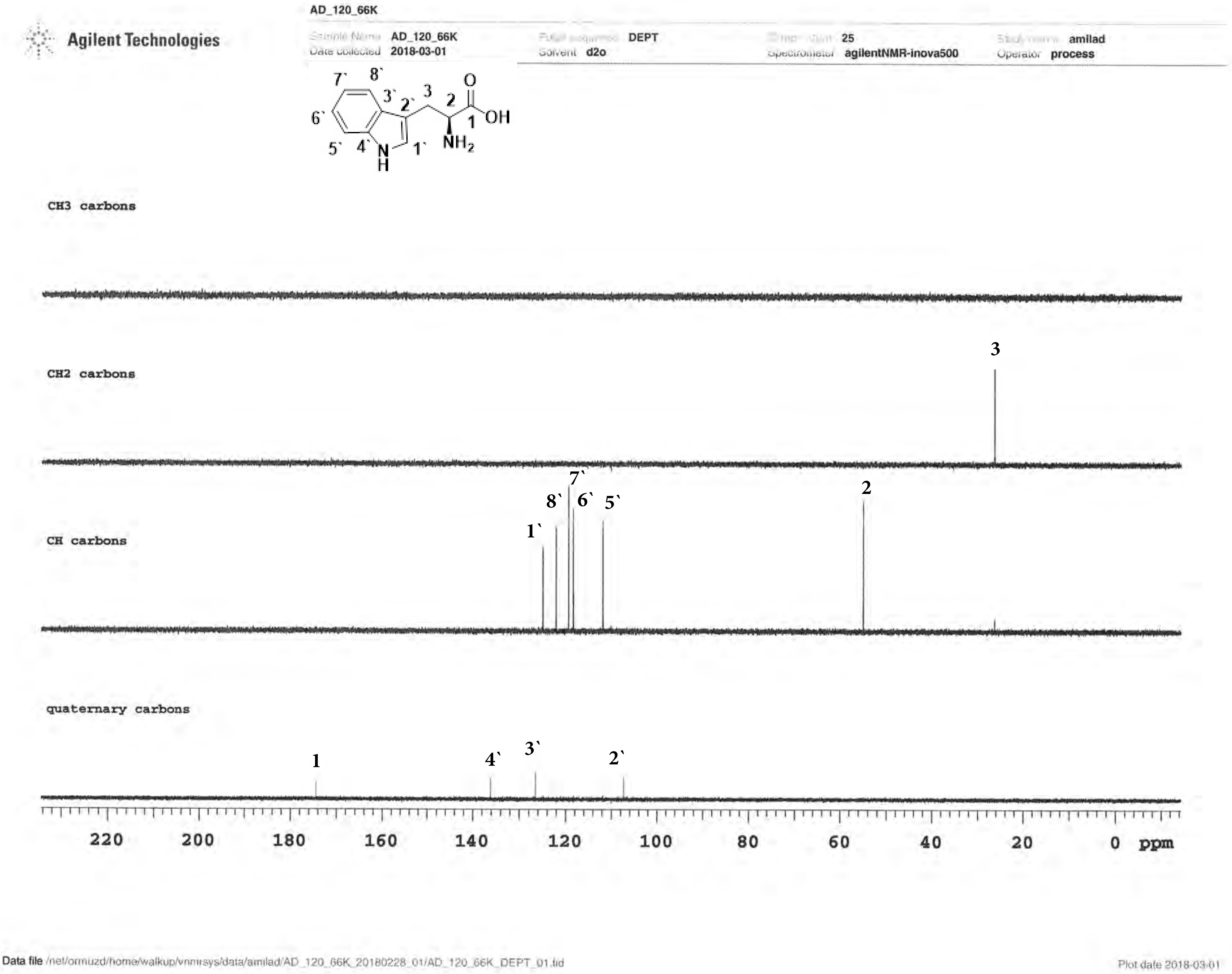
AD_120_66K
Agilent Technologies
AD_120_66K
2018-03-01
DEPT
d2o
25
agilentNMR-inova500
amilad
process
CH3 carbons
CH2 carbons
CH carbons
quaternary carbons
220
200
180
160
140
120
100
80
60
40
20
0 ppm
Data file /net/ormuzd/home/walkup/vnmrsys/data/amilad/AD_120_66K_20180228_01/AD_120_66K_DEPT_01.fid
Plot date 2018-03-01

Figure H

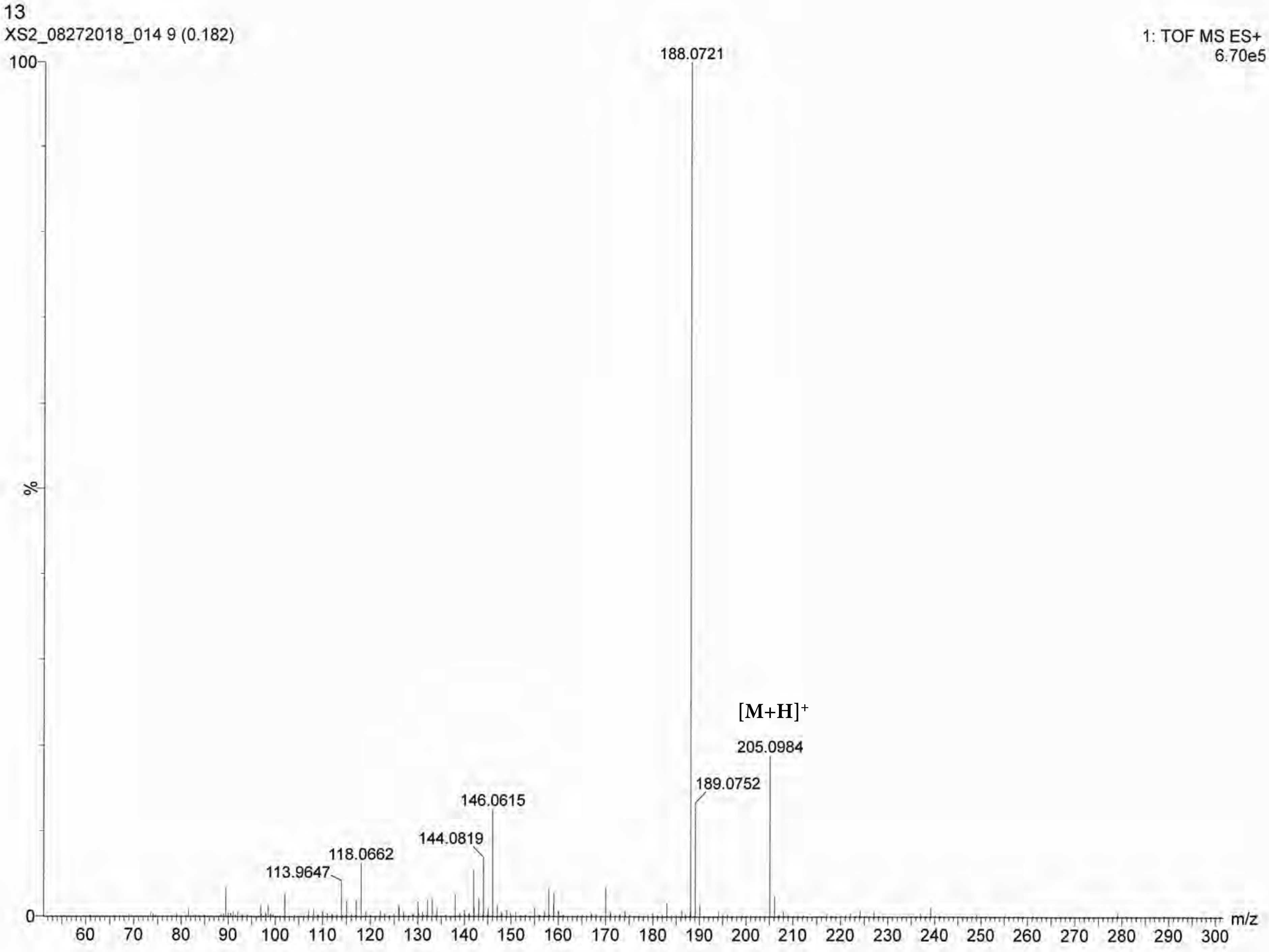
13
XS2_08272018_014 9 (0.182)
1: TOF MS ES+
6.70e5
100
%
0
188.0721
[M+H]+
205.0984
189.0752
146.0615
144.0819
118.0662
113.9647
60 70 80 90 100 110 120 130 140 150 160 170 180 190 200 210 220 230 240 250 260 270 280 290 300
m/z

Figure I

Sample Name:
AD_120_93D
Data Collected on:
ormuzd-vnmrs500
Archive directory:
/home/walkup/vnmrsys/data/amilad
Sample directory:
AD_120_93D_20180711_01
FidFile: AD_120_93D_PROTON_01

Pulse Sequence: PROTON (s2pul)
Solvent: d2o
Data collected on: Jul 11 2018

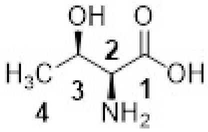

INDEX	FREQUENCY	PPM	HEIGHT
1	2393.5	4.790	16.5
2	2110.[illegible]	[illegible]	[illegible]
3	2108.9	4.220	11.8
4	2104.0	4.211	12.9
5	2102.6	4.208	14.0
6	2097.7	4.198	13.5
7	1771.5	3.545	34.3
8	1766.6	3.535	34.2
9	643.7	1.288	103.9
10	637.3	1.275	107.1

Agilent Technologies

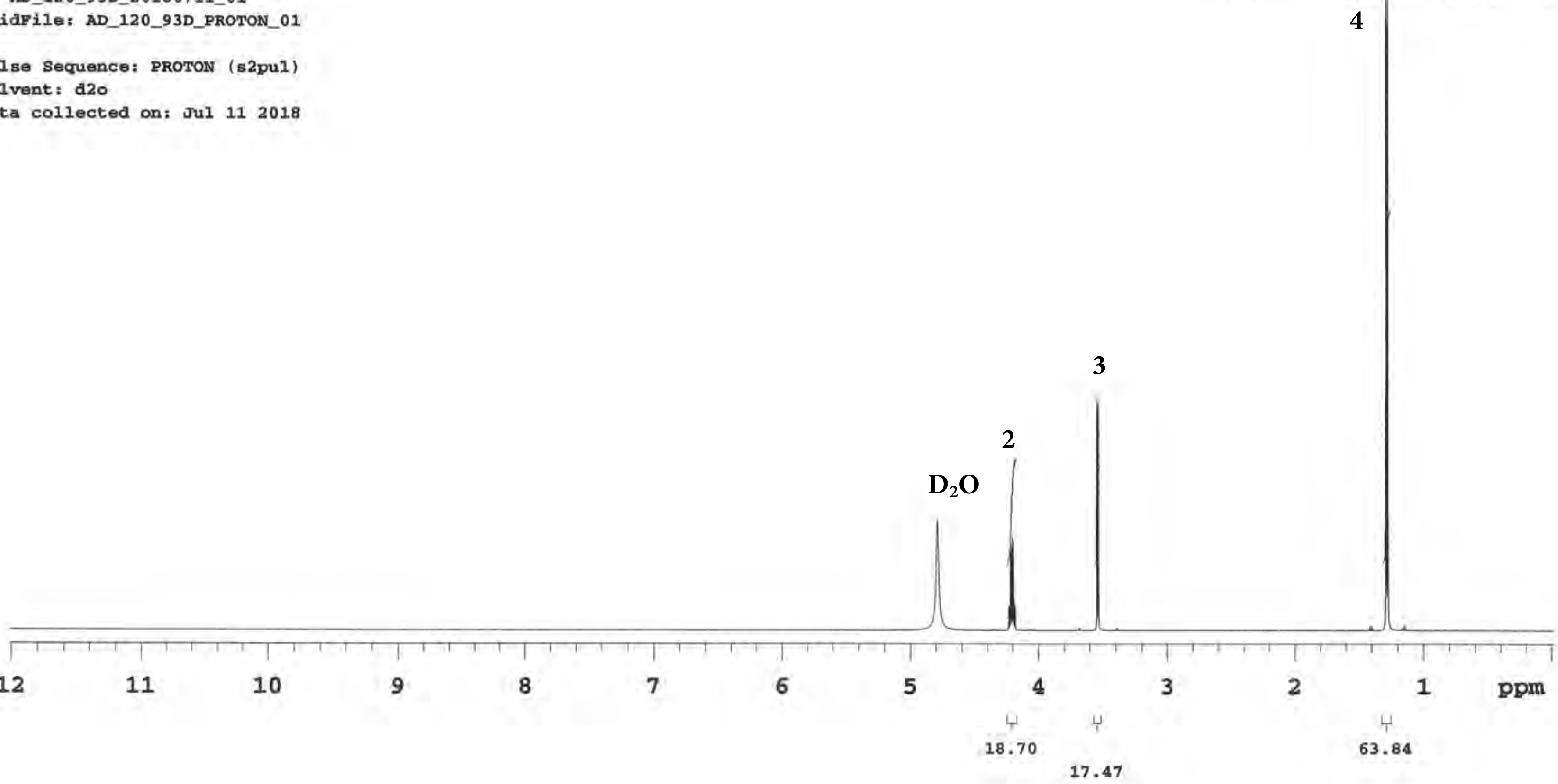

Figure J

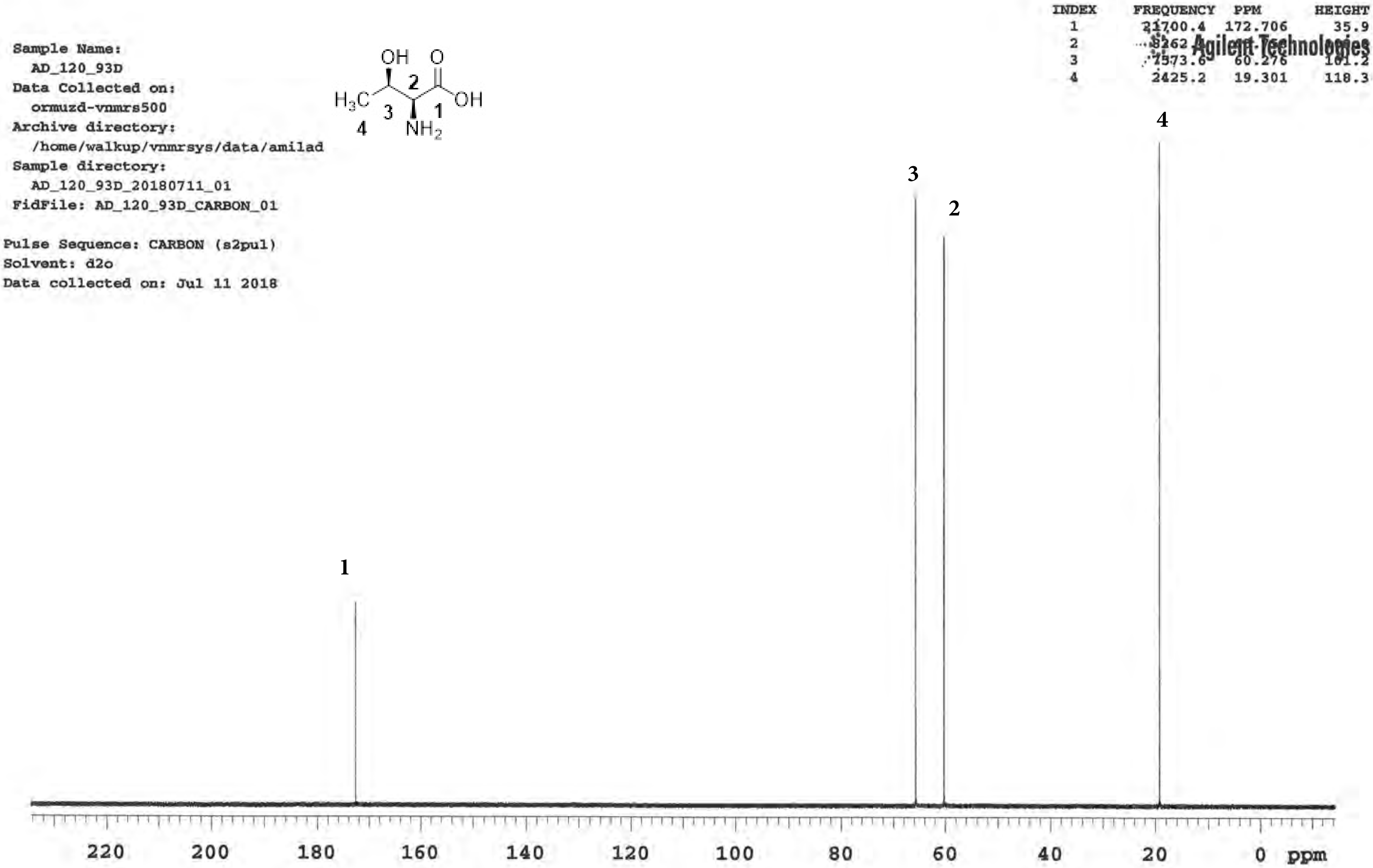
Sample Name:
AD_120_93D
Data Collected on:
ormuzd-vnmrs500
Archive directory:
/home/walkup/vnmrsys/data/amilad
Sample directory:
AD_120_93D_20180711_01
FidFile: AD_120_93D_CARBON_01
Pulse Sequence: CARBON (s2pul)
Solvent: d2o
Data collected on: Jul 11 2018
OH O
H3C 3 2 1 OH
4 NH2
INDEX FREQUENCY PPM HEIGHT
1 21700.4 172.706 35.9
2 8262 [illegible] [illegible]
3 7573.6 60.276 161.2
4 2425.2 19.301 118.3
Agilent Technologies
1
2
3
4
220 200 180 160 140 120 100 80 60 40 20 0 ppm

Figure K

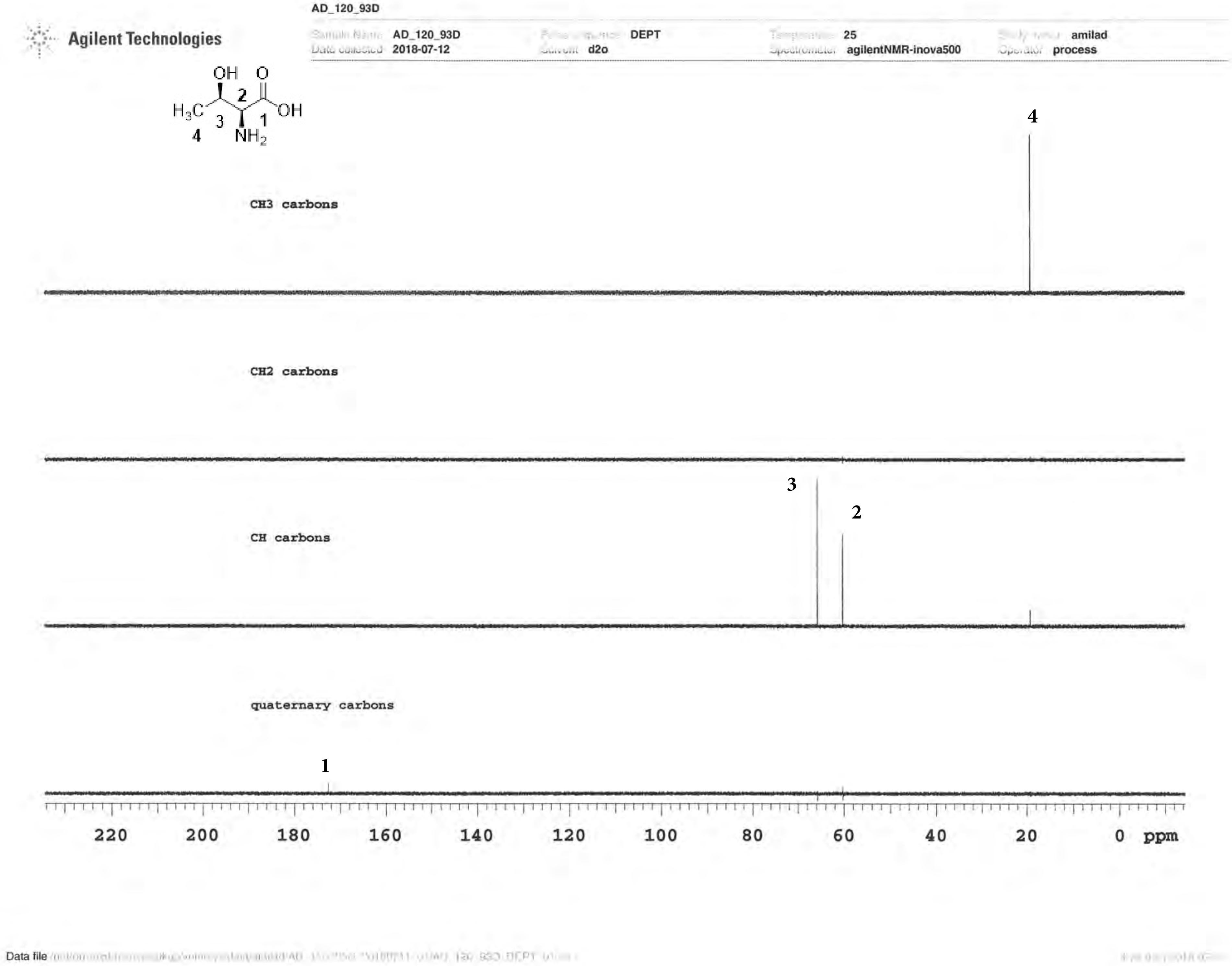
AD_120_93D
Agilent Technologies
AD_120_93D
2018-07-12
DEPT
d2o
25
agilentNMR-inova500
amilad
process
OH
O
OH
H_3C
NH_2
1
2
3
4
CH3 carbons
CH2 carbons
CH carbons
quaternary carbons
220
200
180
160
140
120
100
80
60
40
20
0
ppm
Data file

Figure L

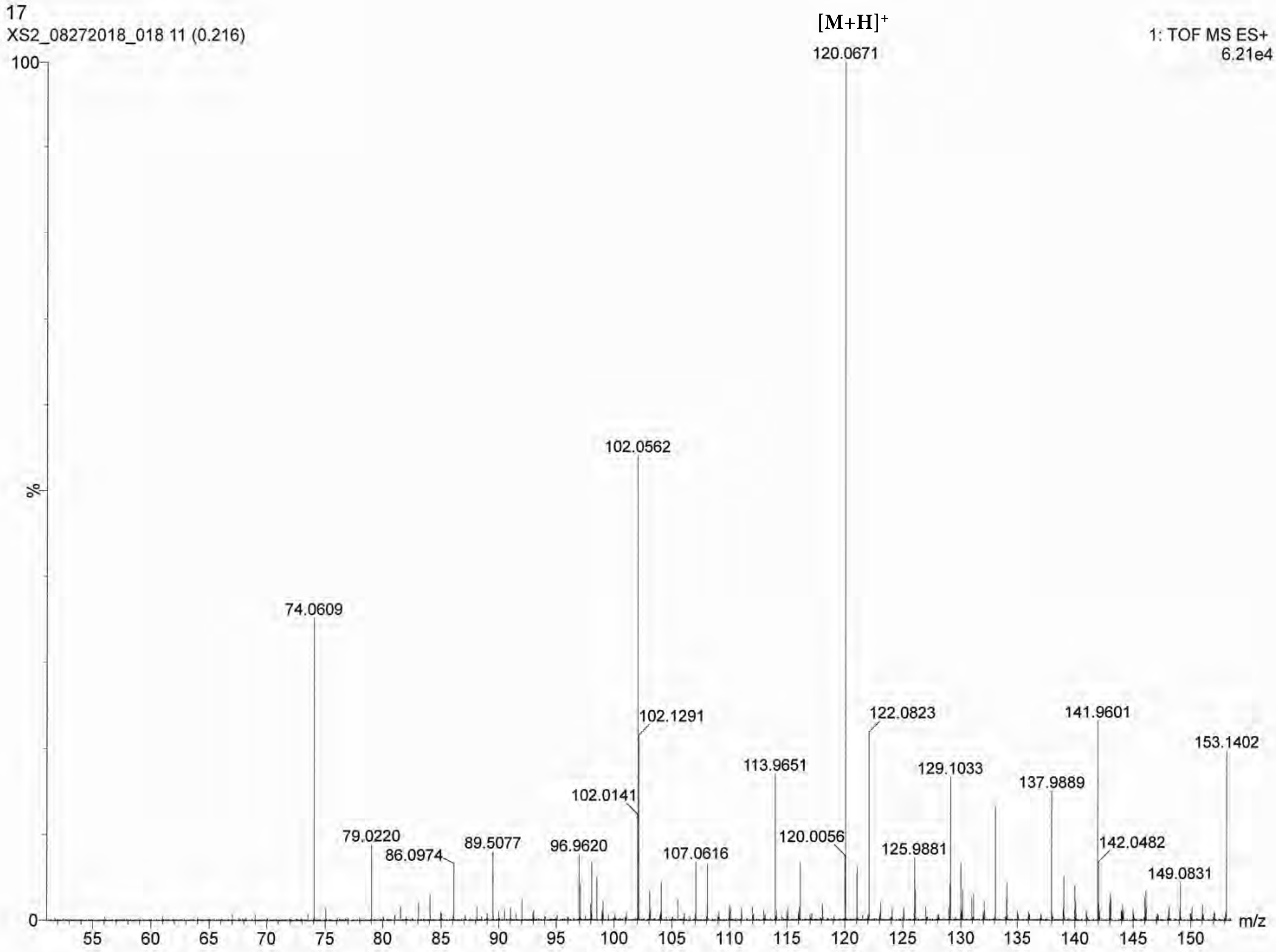
17
XS2_08272018_018 11 (0.216)
[M+H]+
120.0671
1: TOF MS ES+
6.21e4
100
%
0
102.0562
74.0609
102.1291
122.0823
141.9601
153.1402
113.9651
129.1033
137.9889
102.0141
79.0220
120.0056
142.0482
86.0974
89.5077
96.9620
107.0616
125.9881
149.0831
55
60
65
70
75
80
85
90
95
100
105
110
115
120
125
130
135
140
145
150
m/z

Figure M

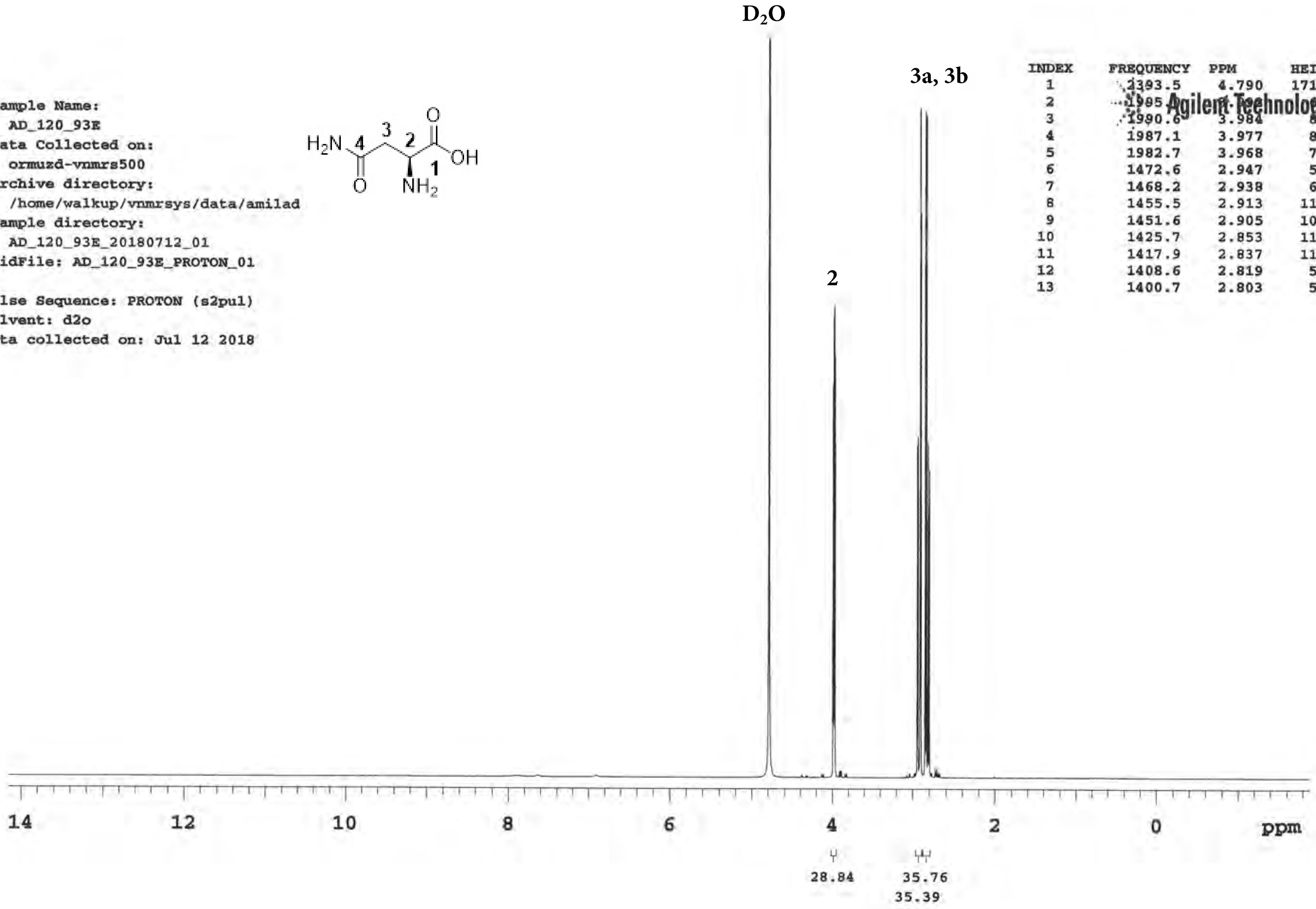

Sample Name:
AD_120_93E
Data Collected on:
ormuzd-vnmrs500
Archive directory:
/home/walkup/vnmrsys/data/amilad
Sample directory:
AD_120_93E_20180712_01
FidFile: AD_120_93E_PROTON_01

Pulse Sequence: PROTON (s2pul)
Solvent: d2o
Data collected on: Jul 12 2018

INDEX	FREQUENCY	PPM	HEIGHT
1	2393.5	4.790	1713.6
2	[illegible]	[illegible]	[illegible]
3	1990.6	3.984	82.1
4	1987.1	3.977	84.2
5	1982.7	3.968	75.5
6	1472.6	2.947	59.2
7	1468.2	2.938	60.8
8	1455.5	2.913	119.4
9	1451.6	2.905	101.0
10	1425.7	2.853	118.7
11	1417.9	2.837	118.2
12	1408.6	2.819	59.8
13	1400.7	2.803	54.8

Figure N

INDEX	FREQUENCY	PPM	HEIGHT
1	21900.2	174.297	29.0
2	21761.4	173.192	47.7
3	6424.7	51.132	108.7
4	4315.1	34.343	105.6

Sample Name:
AD_120_93E
Data Collected on:
ormuzd-vnmrs500
Archive directory:
/home/walkup/vnmrsys/data/amilad
Sample directory:
AD_120_93E_20180712_01
FidFile: AD_120_93E_CARBON_01

Pulse Sequence: CARBON (s2pul)
Solvent: d2o
Data collected on: Jul 12 2018

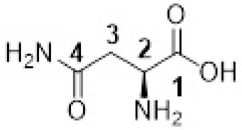

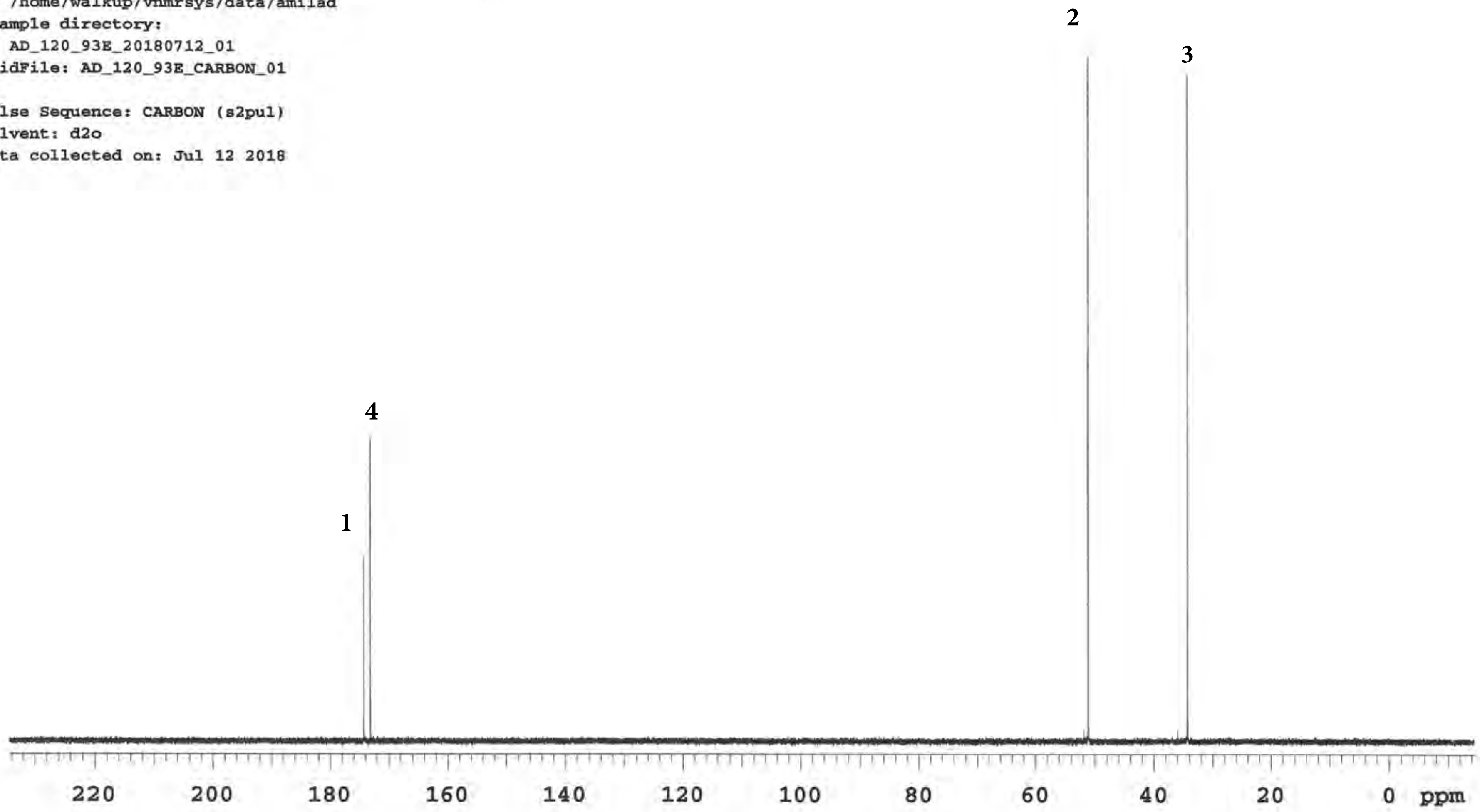

Figure O

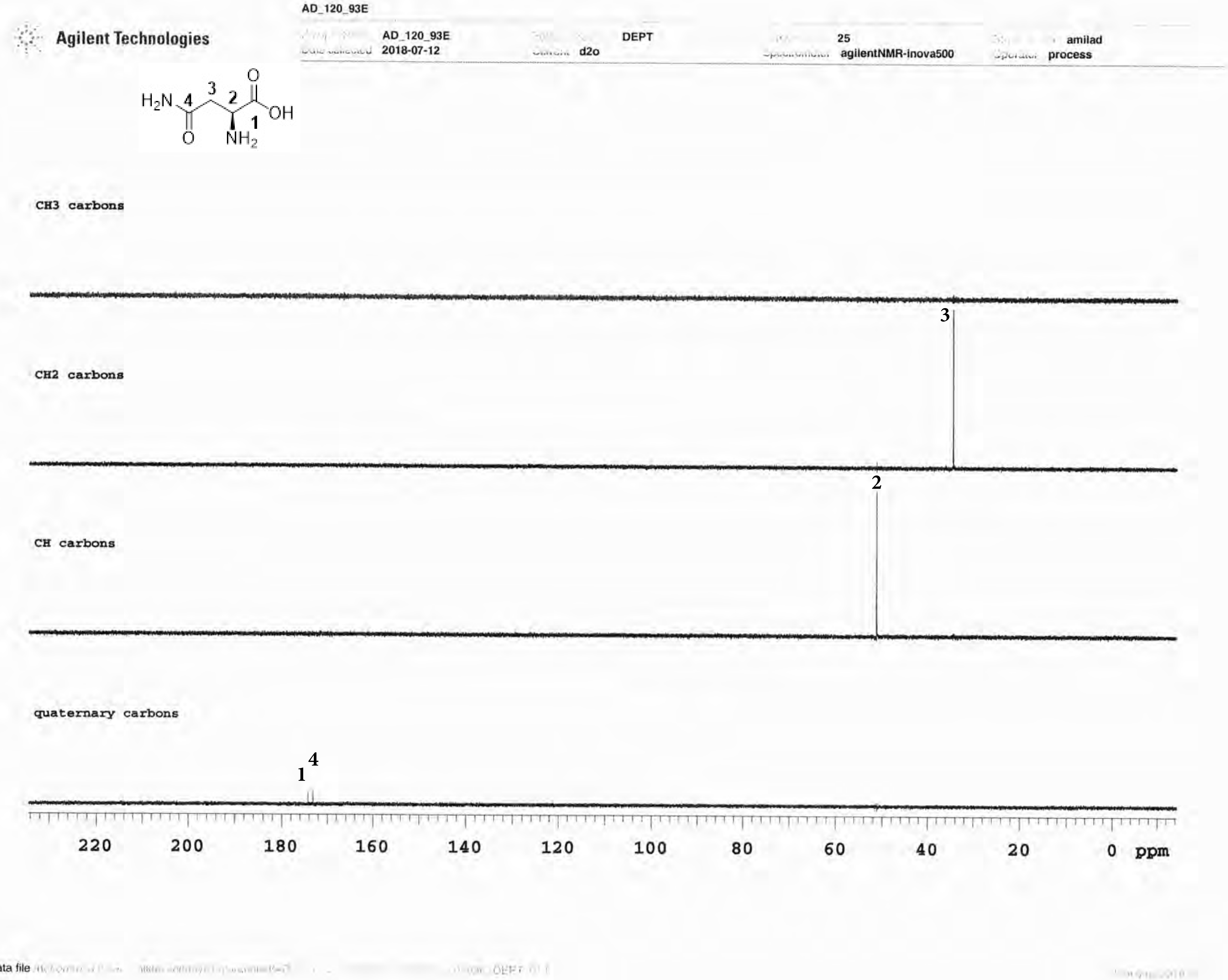

AD_120_93E
Agilent Technologies
AD_120_93E
2018-07-12
DEPT
d2o
25
agilentNMR-inova500
amilad
process
CH3 carbons
CH2 carbons
CH carbons
quaternary carbons
1
2
3
4
220 200 180 160 140 120 100 80 60 40 20 0 ppm
Data file

Figure P

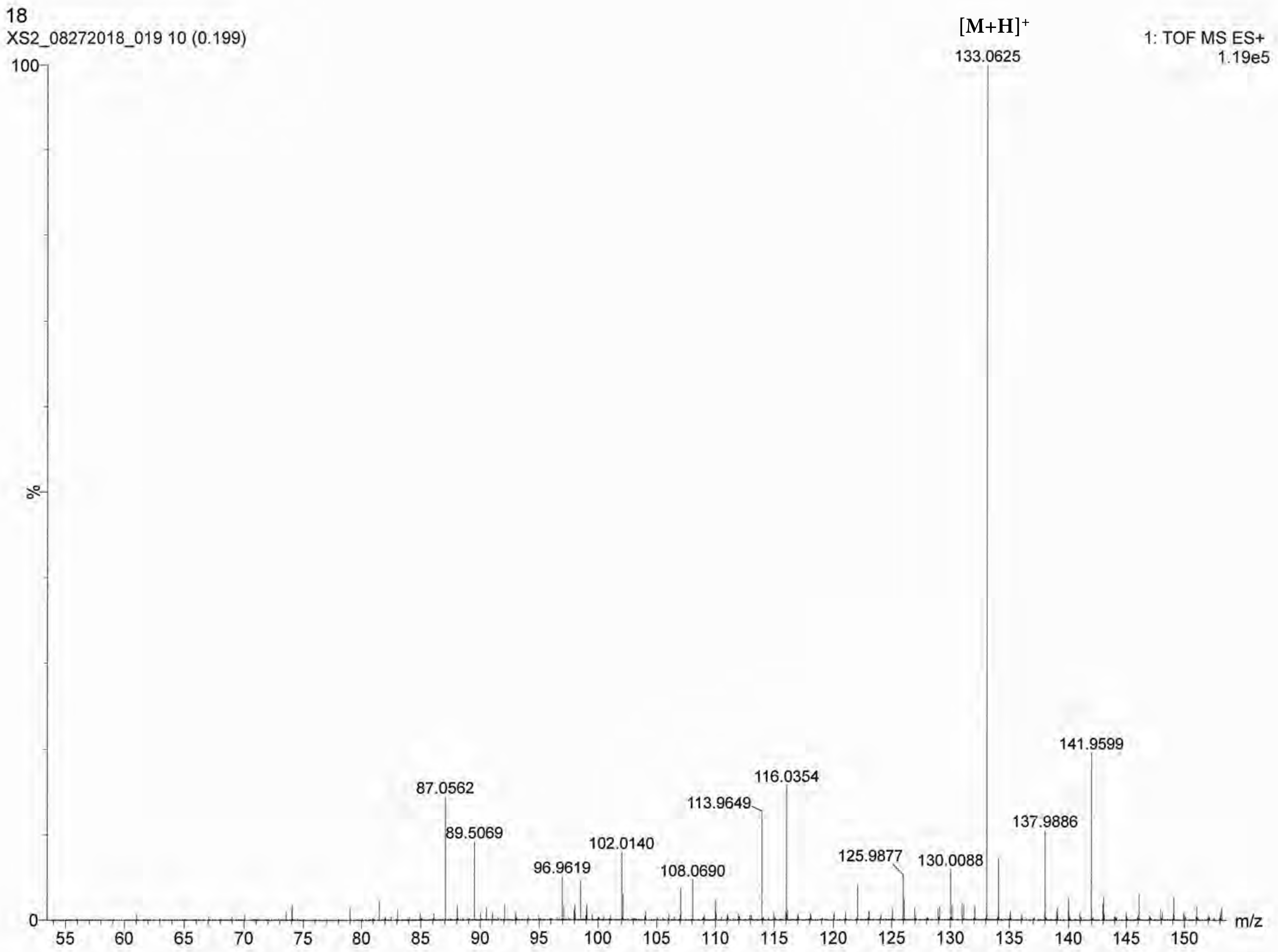
18
XS2_08272018_019 10 (0.199)
[M+H]+
133.0625
1: TOF MS ES+
1.19e5
100
%
0
141.9599
116.0354
87.0562
113.9649
137.9886
89.5069
102.0140
96.9619
108.0690
125.9877
130.0088
55 60 65 70 75 80 85 90 95 100 105 110 115 120 125 130 135 140 145 150
m/z

Figure Q

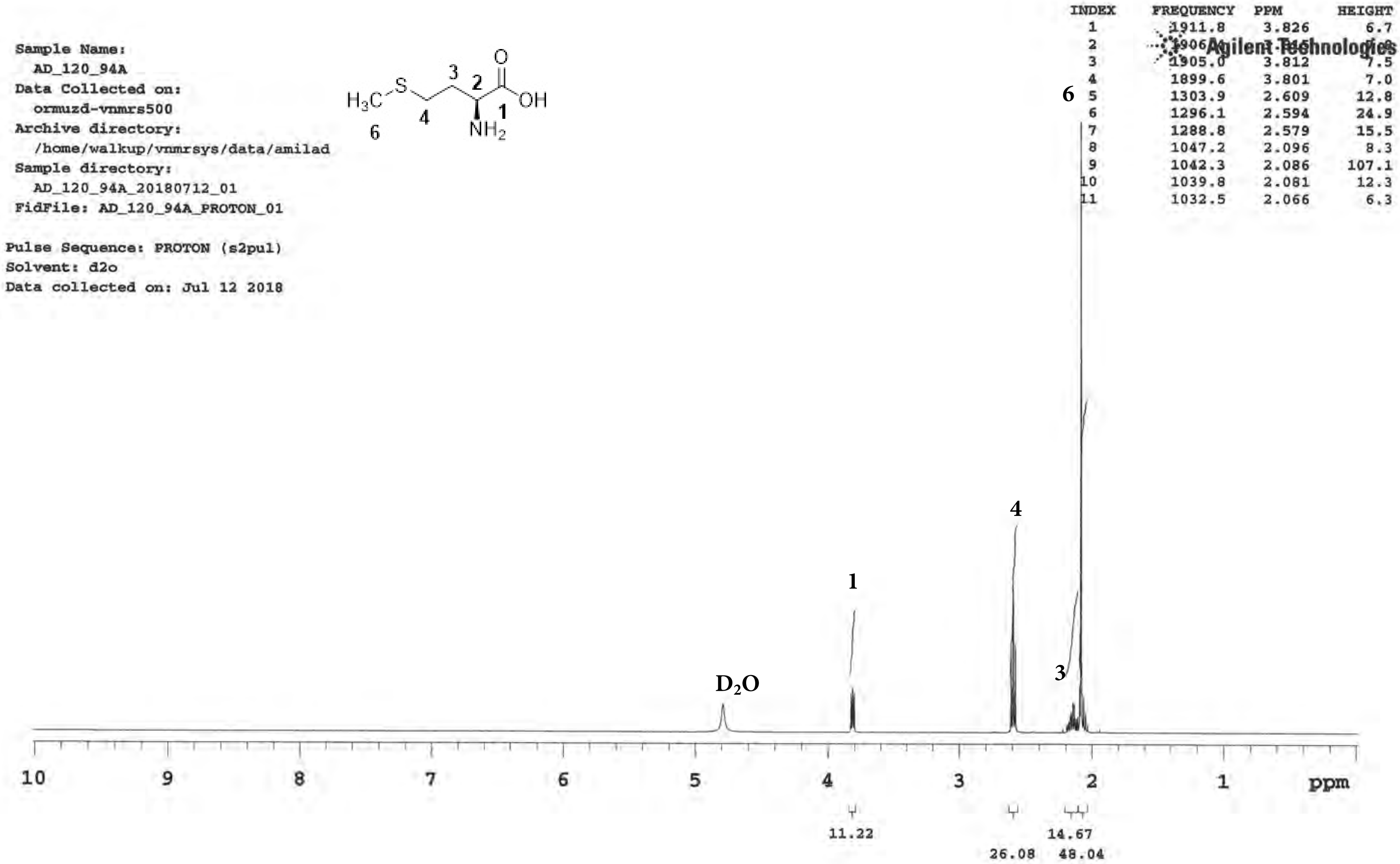

Sample Name:
AD_120_94A
Data Collected on:
ormuzd-vnmrs500
Archive directory:
/home/walkup/vnmrsys/data/amilad
Sample directory:
AD_120_94A_20180712_01
FidFile: AD_120_94A_PROTON_01
Pulse Sequence: PROTON (s2pul)
Solvent: d2o
Data collected on: Jul 12 2018
H_3C S OH O NH_2
1 2 3 4 6
INDEX FREQUENCY PPM HEIGHT
1 1911.8 3.826 6.7
2 1906.[illegible] [illegible] [illegible]
3 1905.0 3.812 7.5
4 1899.6 3.801 7.0
5 1303.9 2.609 12.8
6 1296.1 2.594 24.9
7 1288.8 2.579 15.5
8 1047.2 2.096 8.3
9 1042.3 2.086 107.1
10 1039.8 2.081 12.3
11 1032.5 2.066 6.3
Agilent Technologies
D_2O
1
4
3
6
10 9 8 7 6 5 4 3 2 1 ppm
11.22
26.08
14.67
48.04

Figure R

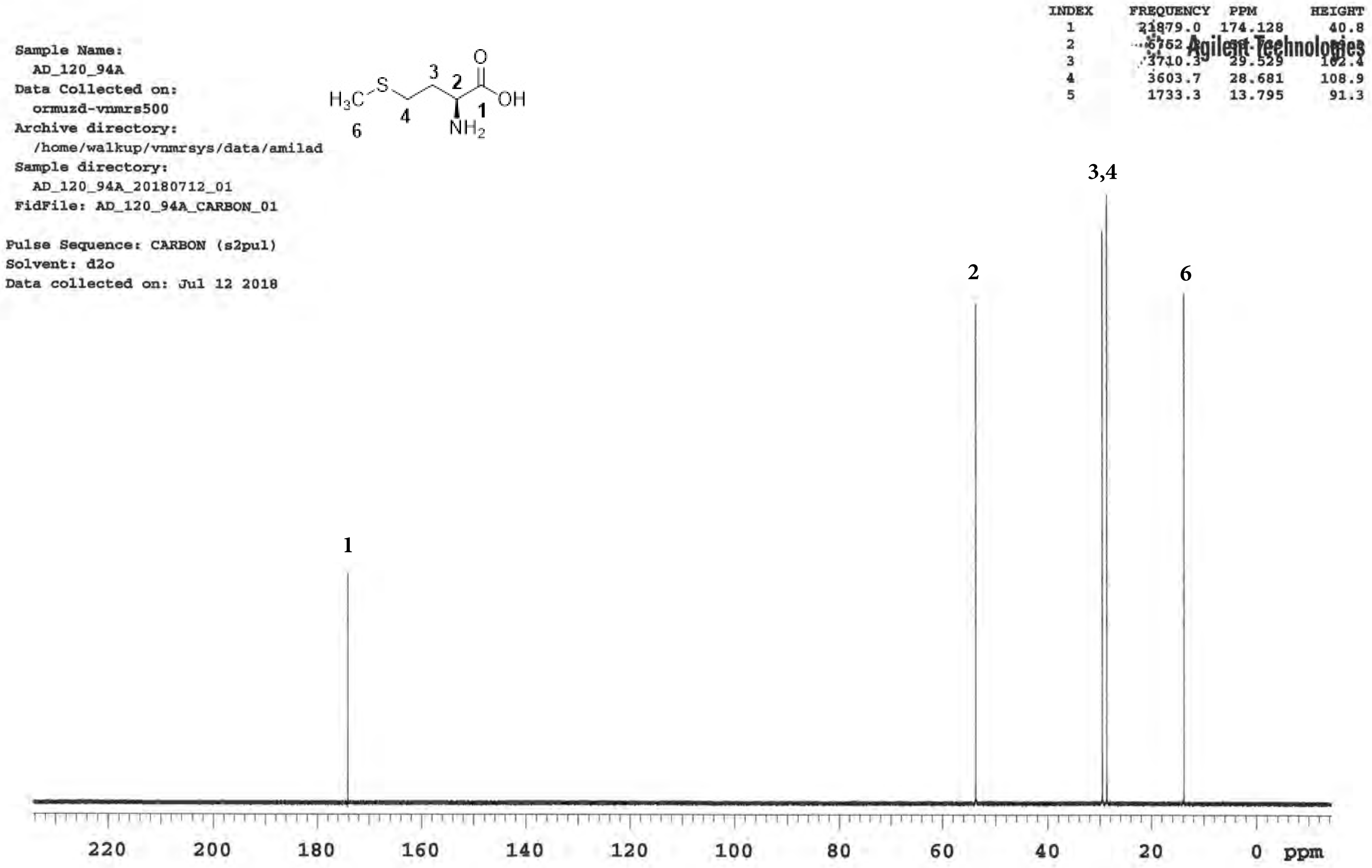
Sample Name:
AD_120_94A
Data Collected on:
ormuzd-vnmrs500
Archive directory:
/home/walkup/vnmrsys/data/amilad
Sample directory:
AD_120_94A_20180712_01
FidFile: AD_120_94A_CARBON_01
Pulse Sequence: CARBON (s2pul)
Solvent: d2o
Data collected on: Jul 12 2018
INDEX FREQUENCY PPM HEIGHT
1 21879.0 174.128 40.8
2 6752[illegible] [illegible] [illegible]
3 3710.3 29.529 1[illegible]2.4
4 3603.7 28.681 108.9
5 1733.3 13.795 91.3
Agilent Technologies
1
2
3,4
6
220 200 180 160 140 120 100 80 60 40 20 0 ppm

Figure S

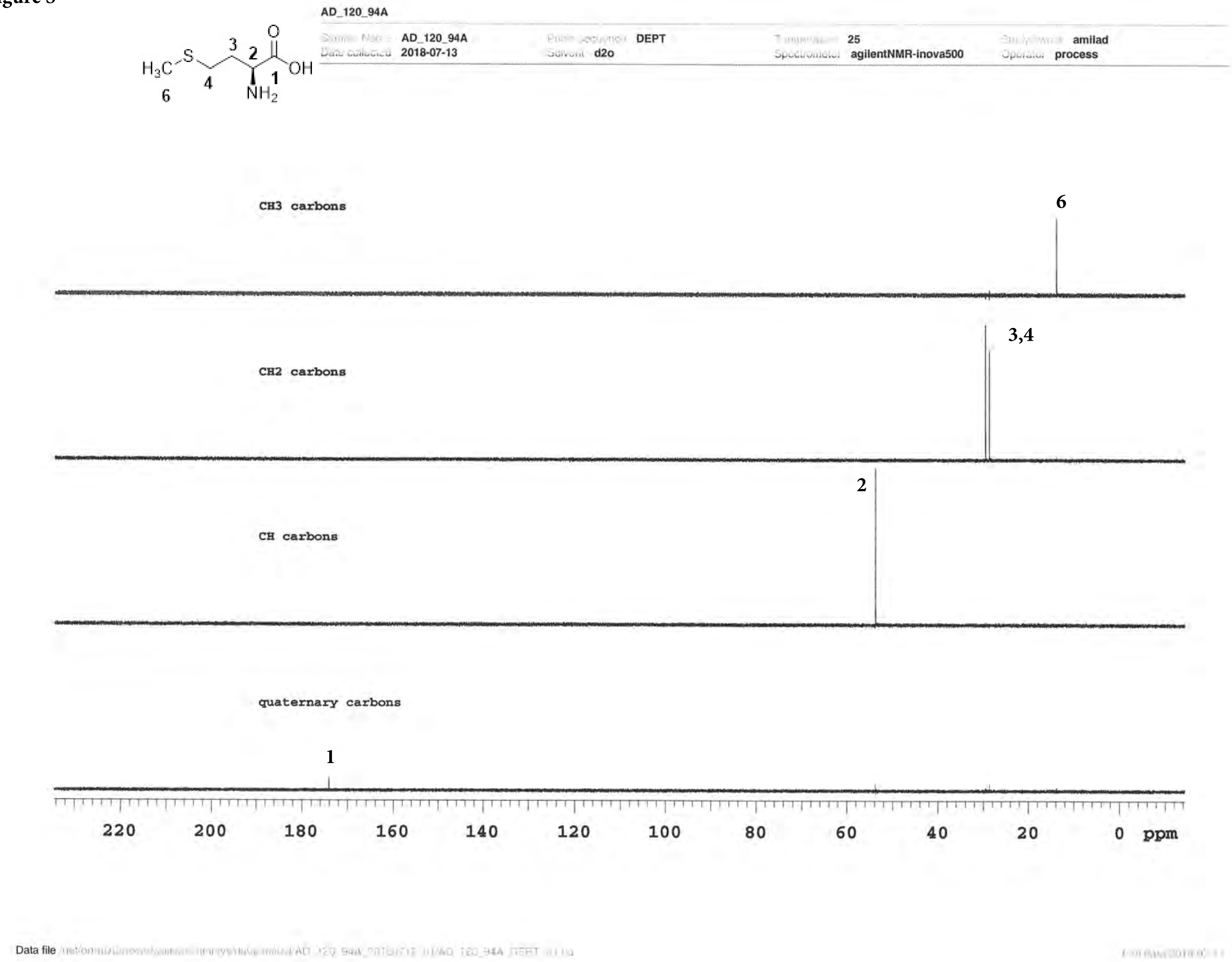

AD_120_94A
AD_120_94A
2018-07-13
DEPT
d2o
25
agilentNMR-inova500
amilad
process
CH3 carbons
CH2 carbons
CH carbons
quaternary carbons
6
3,4
2
1
220 200 180 160 140 120 100 80 60 40 20 0 ppm
Data file

Figure T

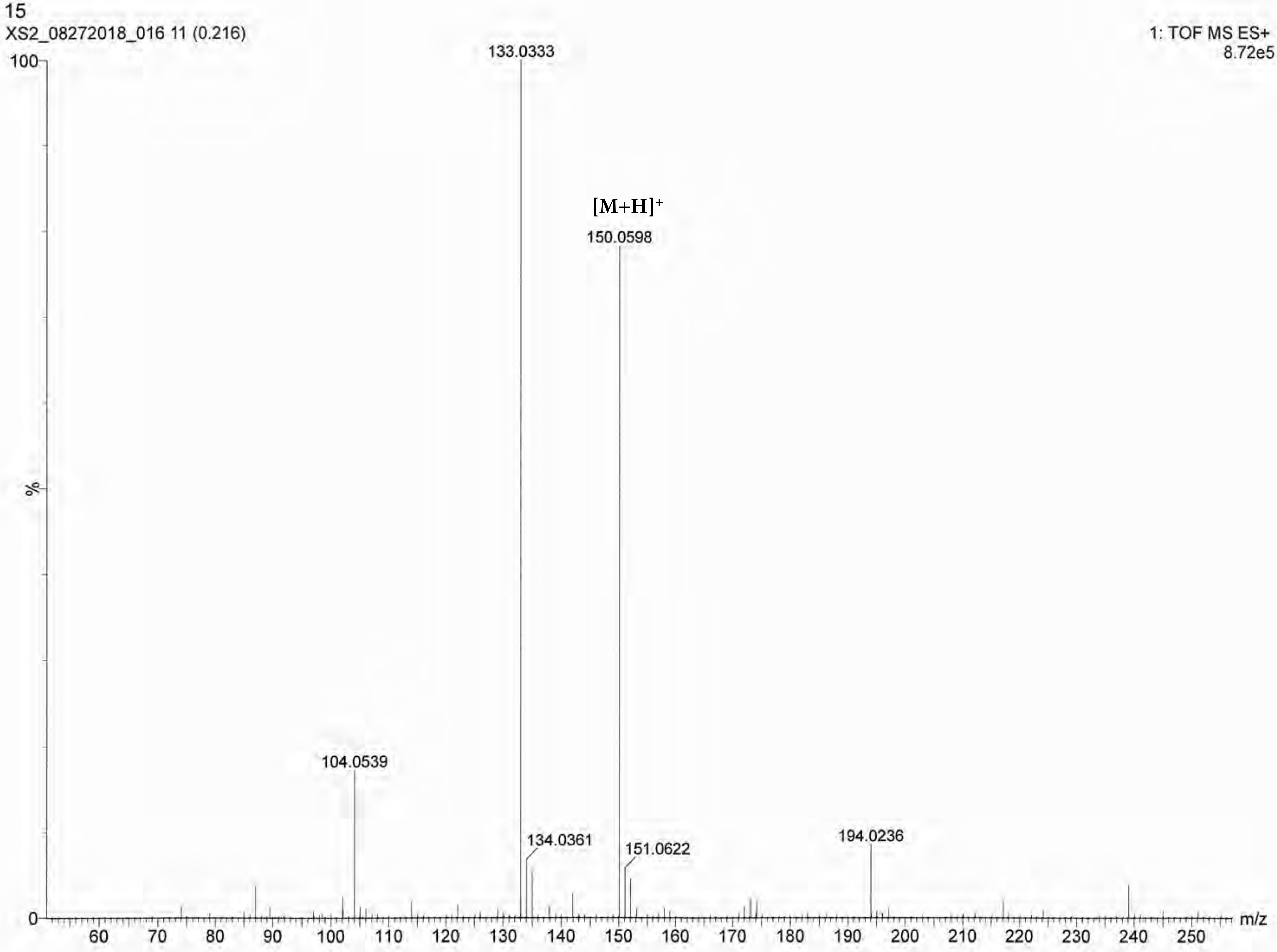
15
XS2_08272018_016 11 (0.216)
1: TOF MS ES+
8.72e5
100
%
0
133.0333
[M+H]+
150.0598
104.0539
134.0361
151.0622
194.0236
60
70
80
90
100
110
120
130
140
150
160
170
180
190
200
210
220
230
240
250
m/z

Figure U

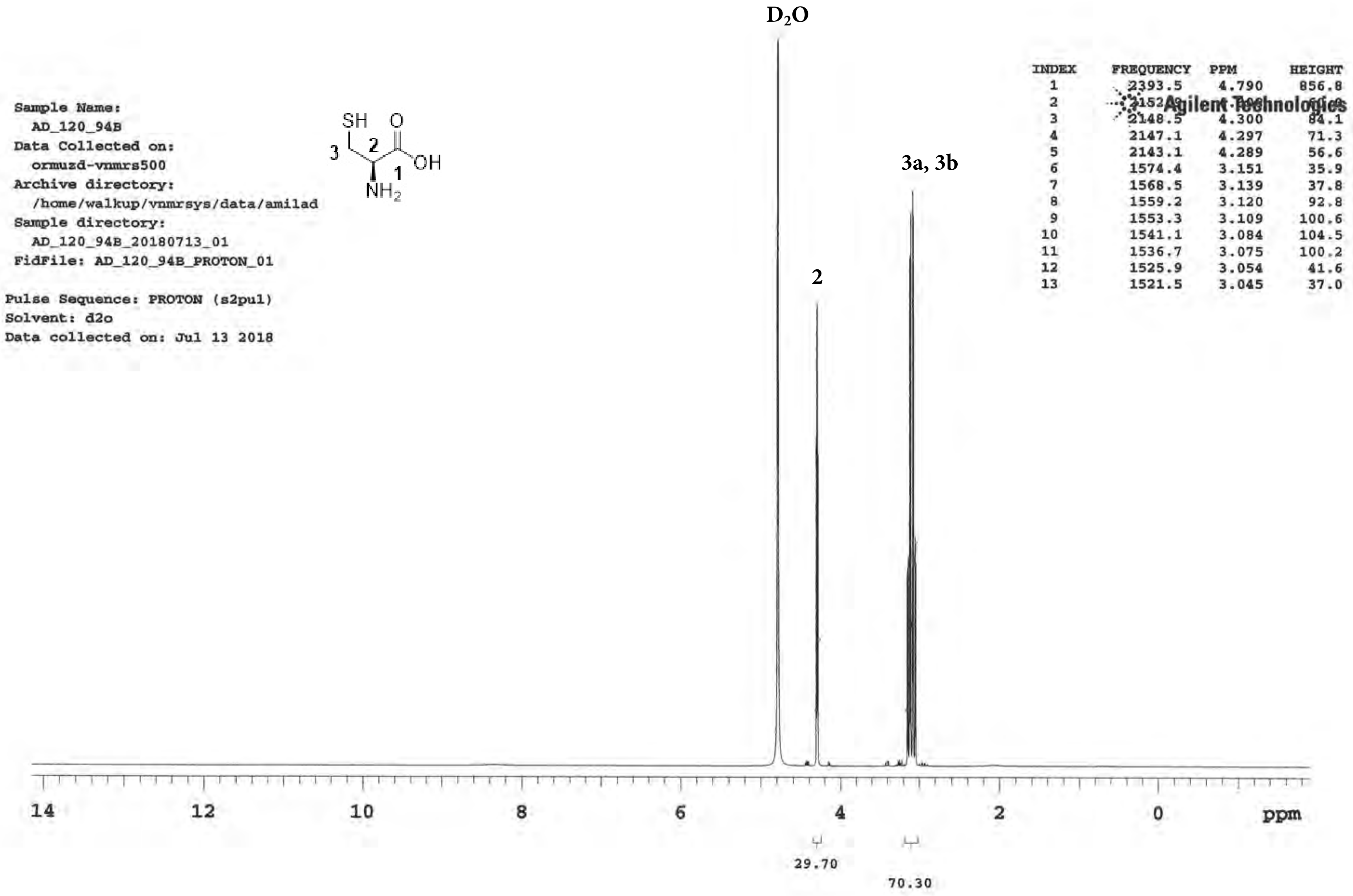

D2O
Sample Name:
AD_120_94B
Data Collected on:
ormuzd-vnmrs500
Archive directory:
/home/walkup/vnmrsys/data/amilad
Sample directory:
AD_120_94B_20180713_01
FidFile: AD_120_94B_PROTON_01
Pulse Sequence: PROTON (s2pul)
Solvent: d2o
Data collected on: Jul 13 2018
SH
O
OH
NH2
3
2
1
3a, 3b
INDEX FREQUENCY PPM HEIGHT
1 2393.5 4.790 856.8
2 2152.[illegible] [illegible] [illegible]
3 2148.5 4.300 84.1
4 2147.1 4.297 71.3
5 2143.1 4.289 56.6
6 1574.4 3.151 35.9
7 1568.5 3.139 37.8
8 1559.2 3.120 92.8
9 1553.3 3.109 100.6
10 1541.1 3.084 104.5
11 1536.7 3.075 100.2
12 1525.9 3.054 41.6
13 1521.5 3.045 37.0
14
12
10
8
6
4
2
0
ppm
29.70
70.30

Figure V

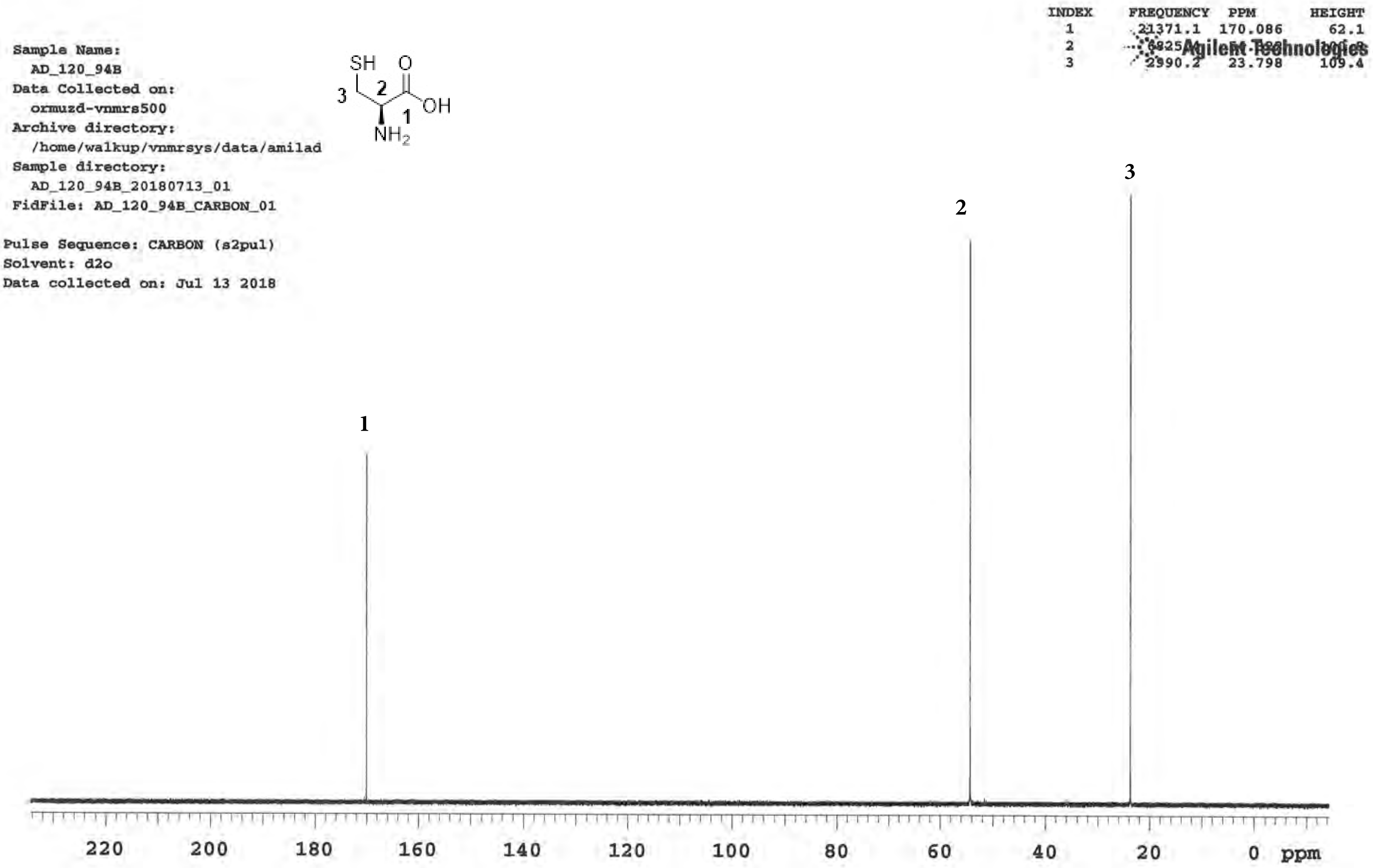
Sample Name:
AD_120_94B
Data Collected on:
ormuzd-vnmrs500
Archive directory:
/home/walkup/vnmrsys/data/amilad
Sample directory:
AD_120_94B_20180713_01
FidFile: AD_120_94B_CARBON_01
Pulse Sequence: CARBON (s2pul)
Solvent: d2o
Data collected on: Jul 13 2018
SH
O
OH
NH2
1
2
3
INDEX FREQUENCY PPM HEIGHT
1 21371.1 170.086 62.1
2 6825.[illegible] [illegible] 10[illegible]
3 2990.2 23.798 109.4
Agilent Technologies
220
200
180
160
140
120
100
80
60
40
20
0
ppm

Figure W

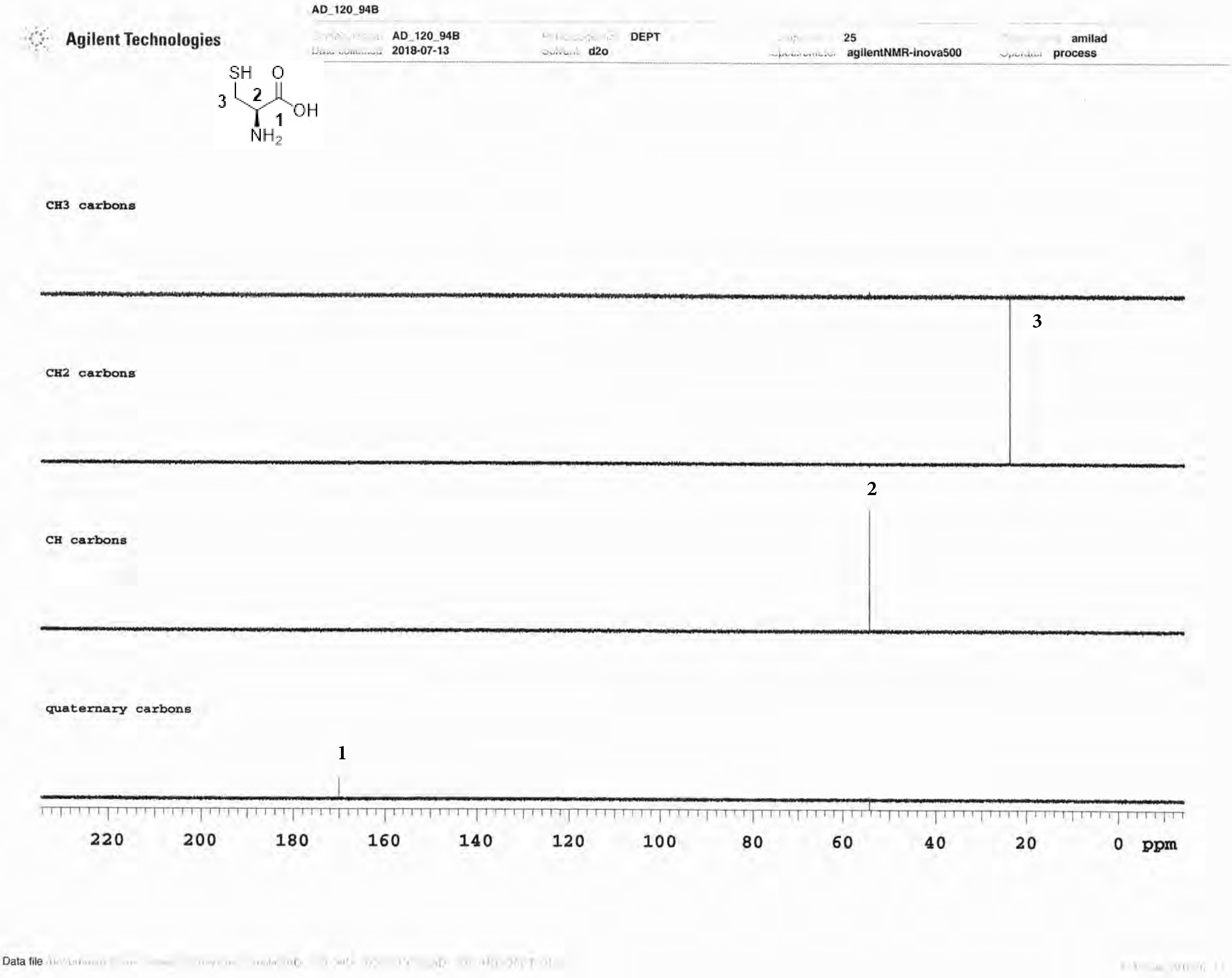
AD_120_94B
Agilent Technologies
AD_120_94B
2018-07-13
DEPT
d2o
25
agilentNMR-inova500
amilad
process
SH
O
3
2
OH
1
NH2
CH3 carbons
3
CH2 carbons
2
CH carbons
quaternary carbons
1
220
200
180
160
140
120
100
80
60
40
20
0
ppm
Data file

Figure X

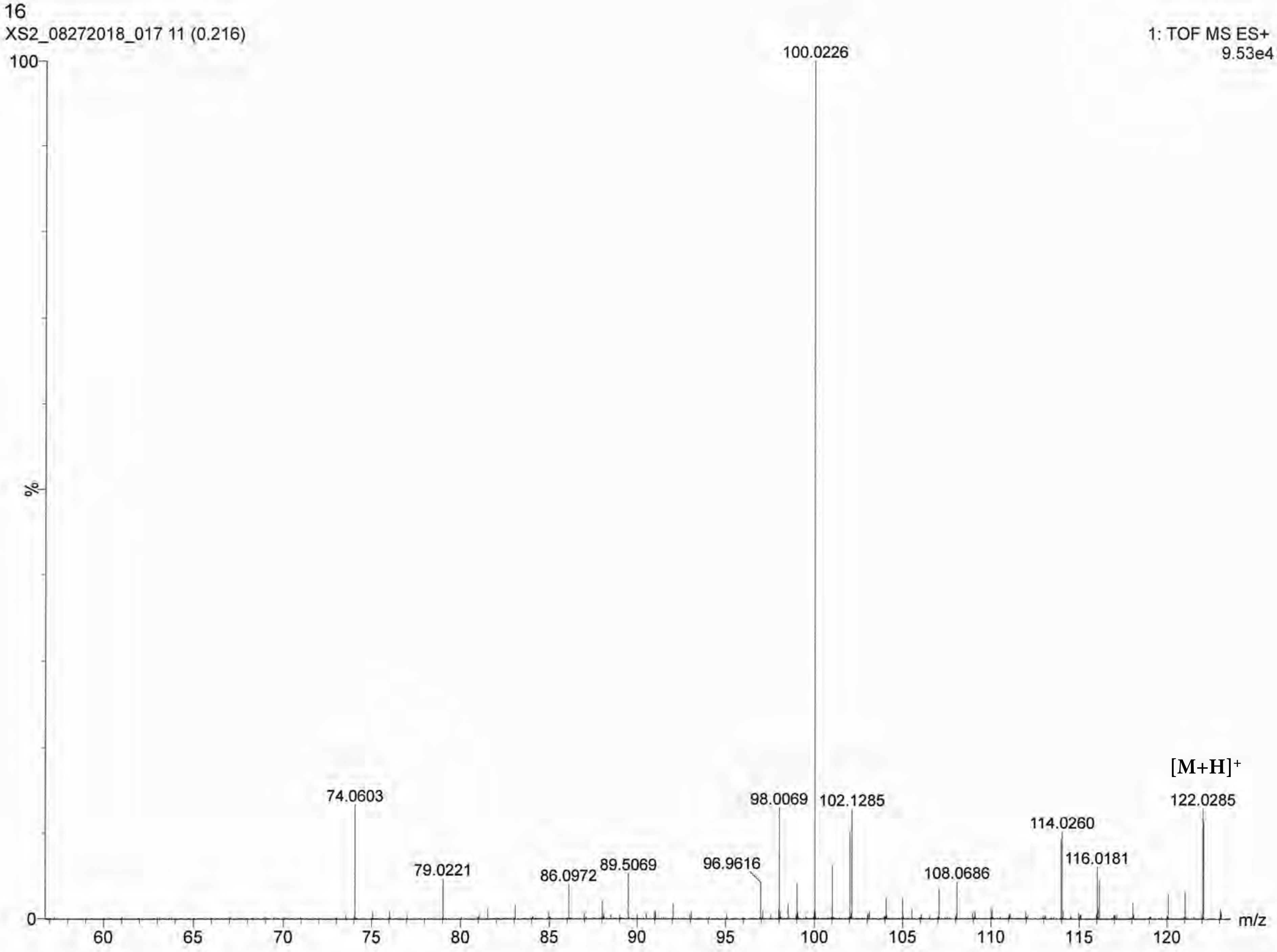
16
XS2_08272018_017 11 (0.216)
1: TOF MS ES+
9.53e4
100.0226
%
[M+H]+
122.0285
74.0603
98.0069
102.1285
114.0260
116.0181
79.0221
86.0972
89.5069
96.9616
108.0686
m/z
100
0
60
65
70
75
80
85
90
95
100
105
110
115
120
