# Supplementary material for: Nitrogenous compounds characterized in the deterrent skin extract of migratory adult sea lamprey from the Great Lakes region
Source: PLoS One. 2019 May 23;14(5):e0217417. doi: 10.1371/journal.pone.0217417 (PMC6532902; doi:10.1371/journal.pone.0217417)
Supplement: S5 File — (PDF) [file pone.0217417.s005.pdf]

Nitrogenous Compounds Characterized in the deterrent Skin extract of Migratory Adult Sea Lamprey from the Great Lakes Region

Amila A. Dissanayake,[1] C. Michael Wagner,[2] Muraleedharan G. Nair,[1*]

[1] Department of Horticulture, Michigan State University, East Lansing, Michigan, United States of America

[2] Department of Fisheries and Wildlife, Michigan State University, East Lansing, Michigan, United States of America

Supporting Information

Figure A. ^{1}H NMR spectrum of adenosine in DMSO
Figure B. ^{13}C NMR spectrum of adenosine in DMSO
Figure C. DEPT spectrum of adenosine in DMSO
Figure D. HRMS spectrum of adenosine (positive ion mode)
Figure E. ^{1}H NMR spectrum of glycine in D_2O
Figure F. ^{13}C NMR spectrum of glycine in D_2O
Figure G. DEPT spectrum of glycine in D_2O
Figure H. HRMS spectrum of glycine (positive ion mode)

Figure A

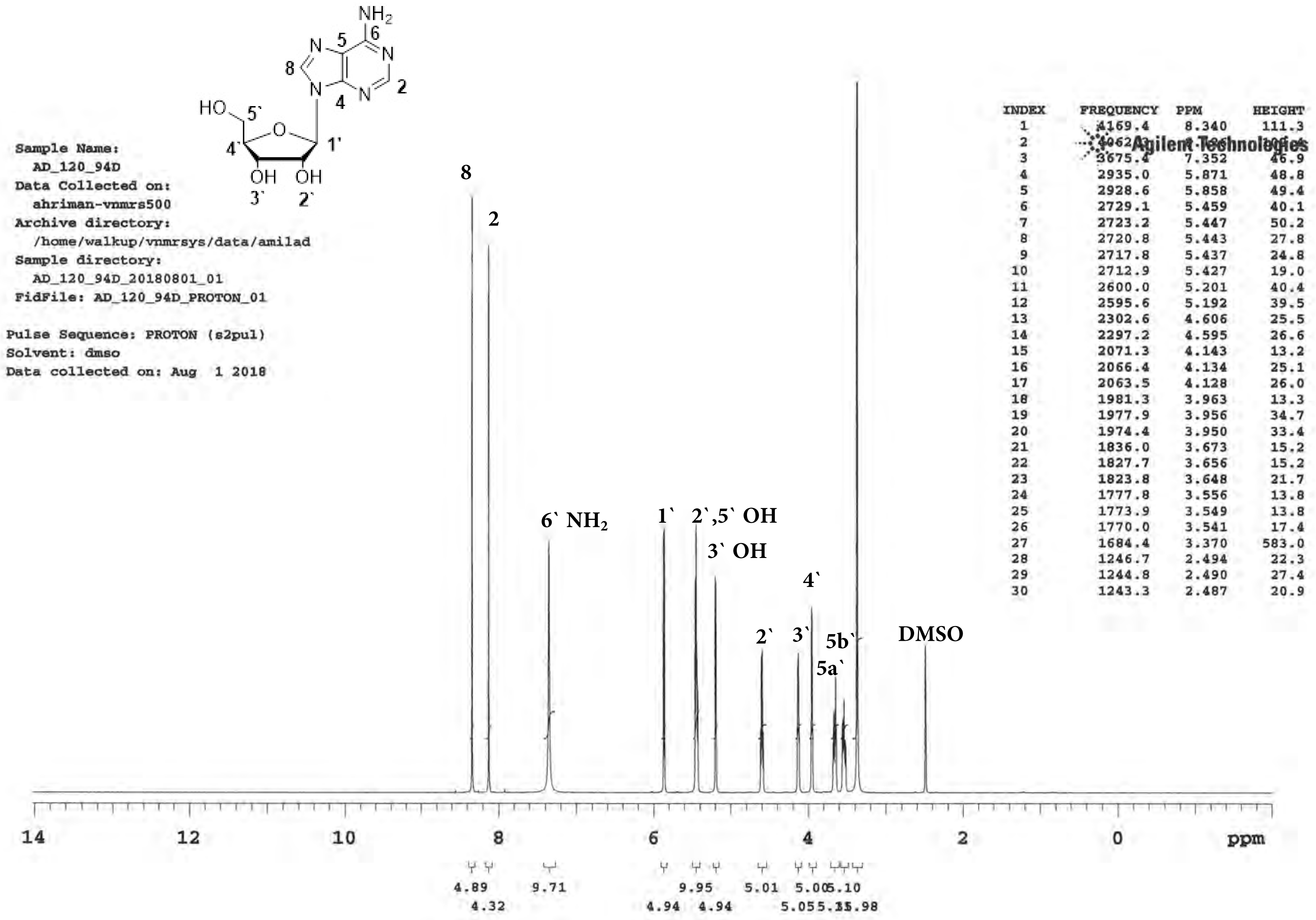

INDEX	FREQUENCY	PPM	HEIGHT
1	4169.4	8.340	111.3
2	4062.[illegible]	[illegible]	[illegible]
3	3675.4	7.352	46.9
4	2935.0	5.871	48.8
5	2928.6	5.858	49.4
6	2729.1	5.459	40.1
7	2723.2	5.447	50.2
8	2720.8	5.443	27.8
9	2717.8	5.437	24.8
10	2712.9	5.427	19.0
11	2600.0	5.201	40.4
12	2595.6	5.192	39.5
13	2302.6	4.606	25.5
14	2297.2	4.595	26.6
15	2071.3	4.143	13.2
16	2066.4	4.134	25.1
17	2063.5	4.128	26.0
18	1981.3	3.963	13.3
19	1977.9	3.956	34.7
20	1974.4	3.950	33.4
21	1836.0	3.673	15.2
22	1827.7	3.656	15.2
23	1823.8	3.648	21.7
24	1777.8	3.556	13.8
25	1773.9	3.549	13.8
26	1770.0	3.541	17.4
27	1684.4	3.370	583.0
28	1246.7	2.494	22.3
29	1244.8	2.490	27.4
30	1243.3	2.487	20.9

Figure B

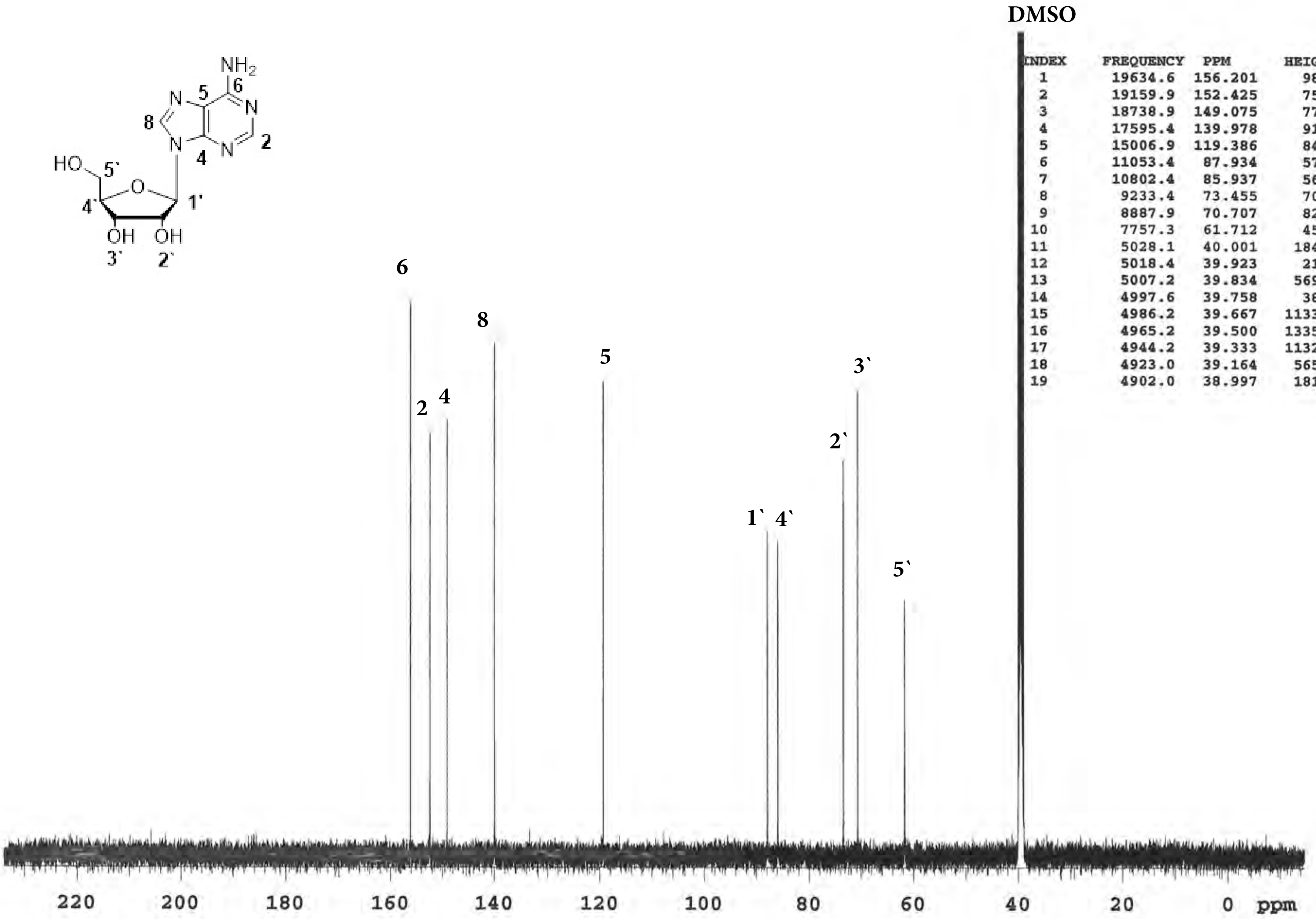

INDEX	FREQUENCY	PPM	HEIGHT
1	19634.6	156.201	98.4
2	19159.9	152.425	75.2
3	18738.9	149.075	77.6
4	17595.4	139.978	91.3
5	15006.9	119.386	84.4
6	11053.4	87.934	57.8
7	10802.4	85.937	56.0
8	9233.4	73.455	70.4
9	8887.9	70.707	82.9
10	7757.3	61.712	45.6
11	5028.1	40.001	184.7
12	5018.4	39.923	21.6
13	5007.2	39.834	569.3
14	4997.6	39.758	38.5
15	4986.2	39.667	1133.3
16	4965.2	39.500	1335.2
17	4944.2	39.333	1132.2
18	4923.0	39.164	565.1
19	4902.0	38.997	181.3

Figure C

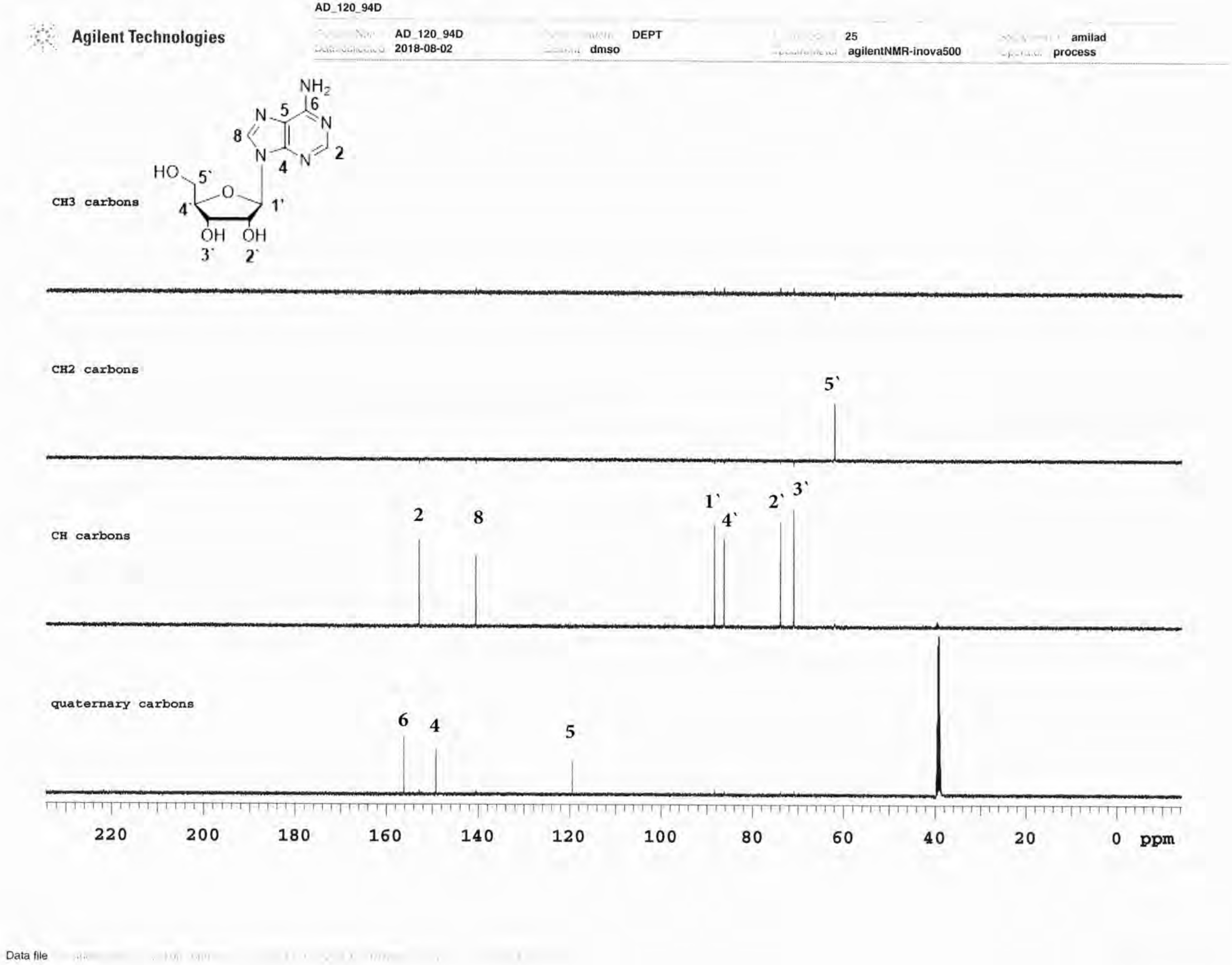
AD_120_94D
Agilent Technologies
AD_120_94D
2018-08-02
DEPT
dmso
25
agilentNMR-inova500
amilad
process
CH3 carbons
CH2 carbons
CH carbons
quaternary carbons
5`
2
8
1`
4`
2`
3`
6
4
5
220
200
180
160
140
120
100
80
60
40
20
0
ppm
Data file

Figure D

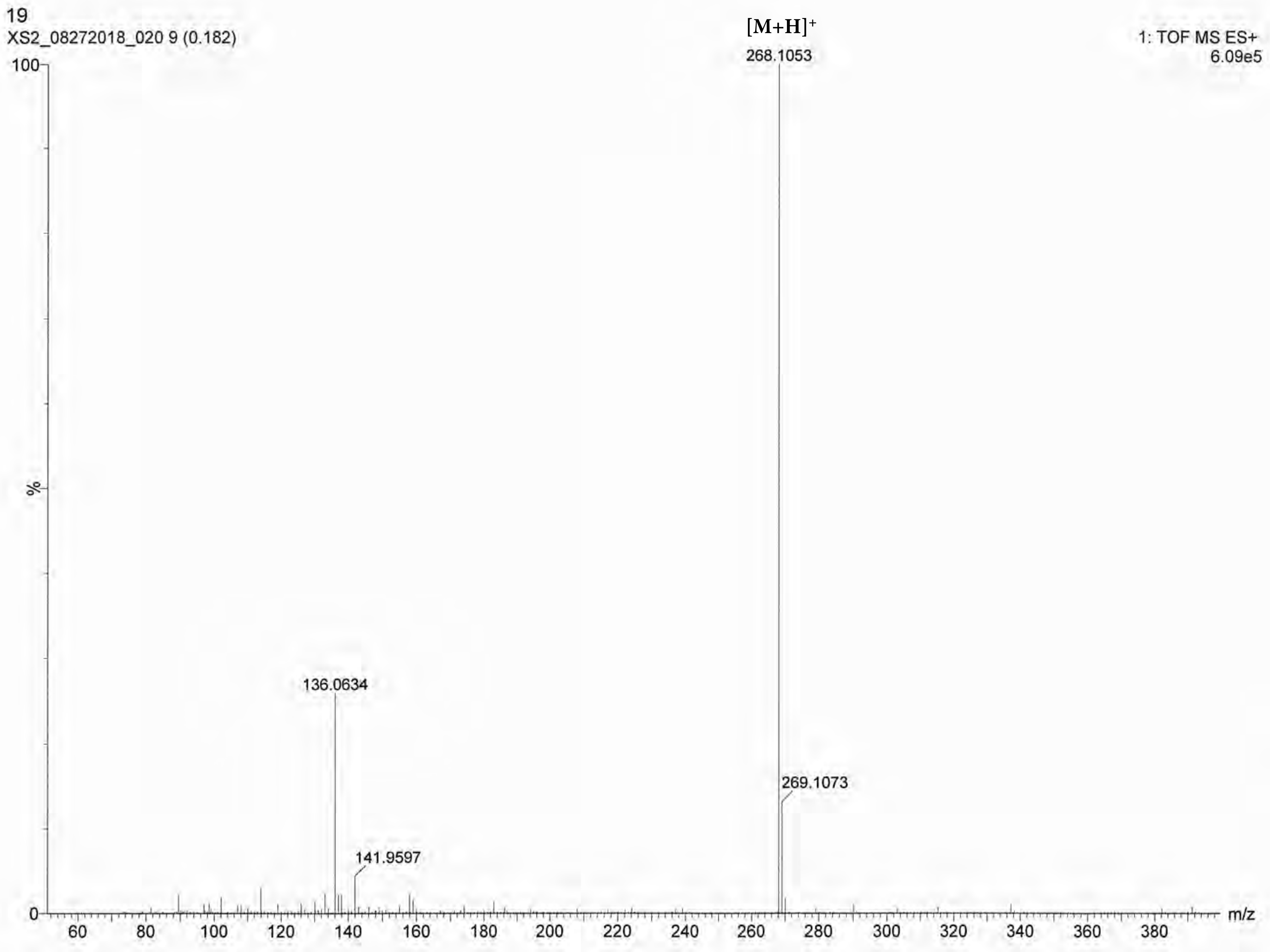

19
XS2_08272018_020 9 (0.182)
[M+H]+
268.1053
1: TOF MS ES+
6.09e5
100
%
0
136.0634
141.9597
269.1073
60
80
100
120
140
160
180
200
220
240
260
280
300
320
340
360
380
m/z

Figure E

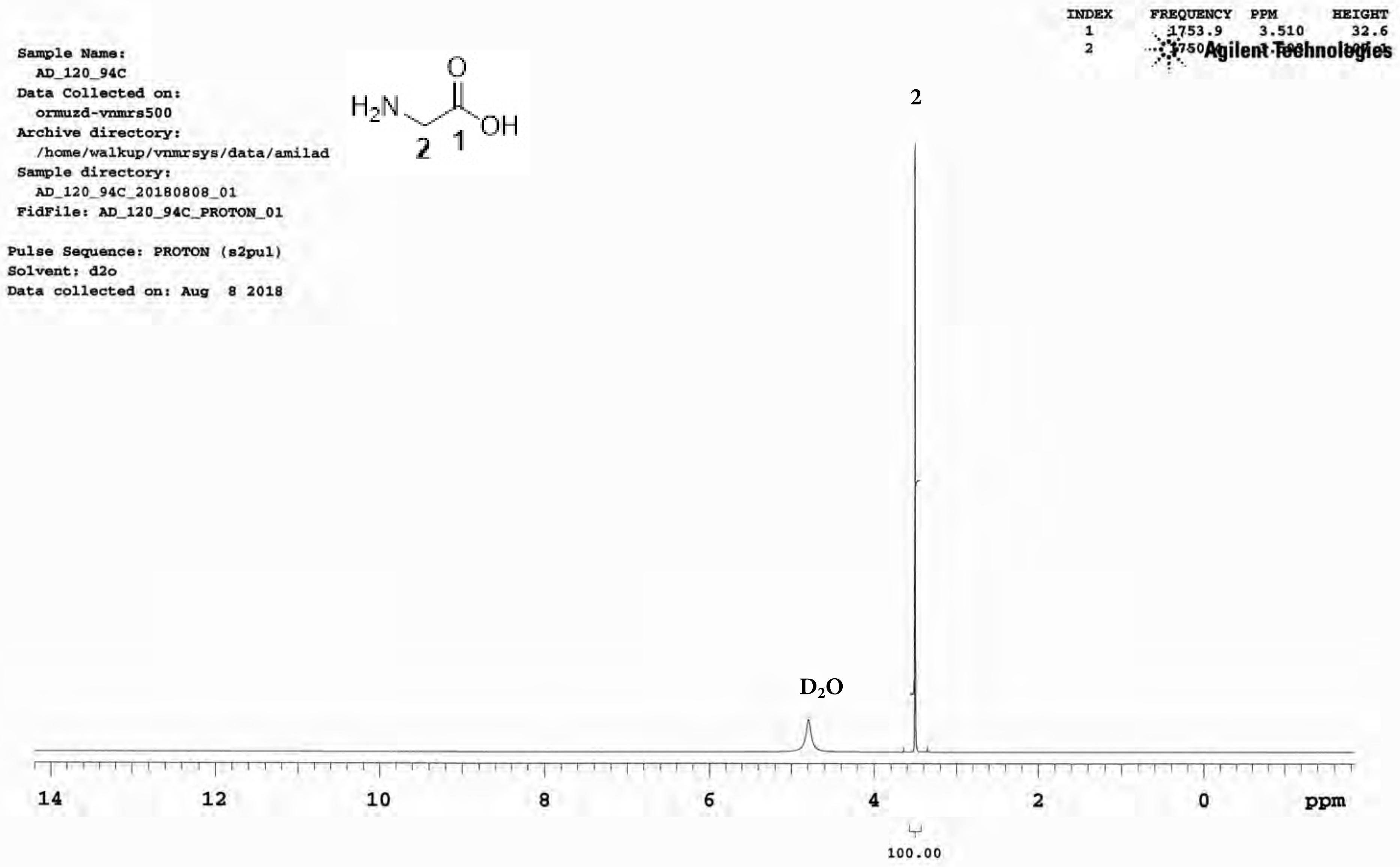

INDEX FREQUENCY PPM HEIGHT
1 1753.9 3.510 32.6
2
Agilent Technologies
Sample Name:
AD_120_94C
Data Collected on:
ormuzd-vnmrs500
Archive directory:
/home/walkup/vnmrsys/data/amilad
Sample directory:
AD_120_94C_20180808_01
FidFile: AD_120_94C_PROTON_01
Pulse Sequence: PROTON (s2pul)
Solvent: d2o
Data collected on: Aug 8 2018
O
H2N
OH
2
1
2
D2O
14
12
10
8
6
4
2
0
ppm
100.00

Figure F

INDEX	FREQUENCY	PPM	HEIGHT
1	21654.4	172.340	7.6
2	[illegible]	[illegible]	[illegible]
3	5186.1	41.274	97.9

Sample Name:
AD_120_94C
Data Collected on:
ormuzd-vnmrs500
Archive directory:
/home/walkup/vnmrsys/data/amilad
Sample directory:
AD_120_94C_20180809_01
FidFile: AD_120_94C_CARBON_01

Pulse Sequence: CARBON (s2pul)
Solvent: d2o
Data collected on: Aug 9 2018

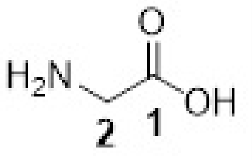

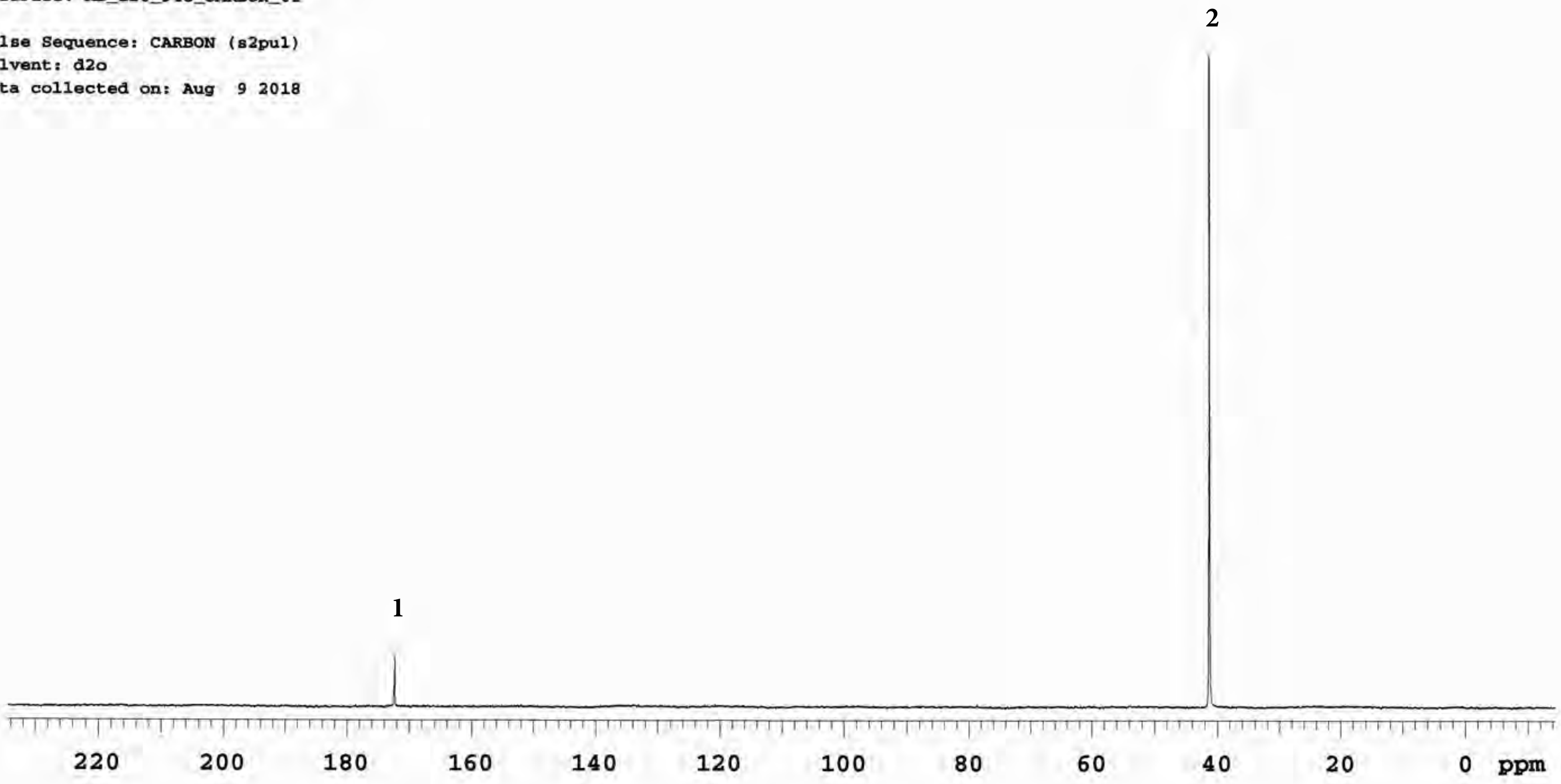

Figure G

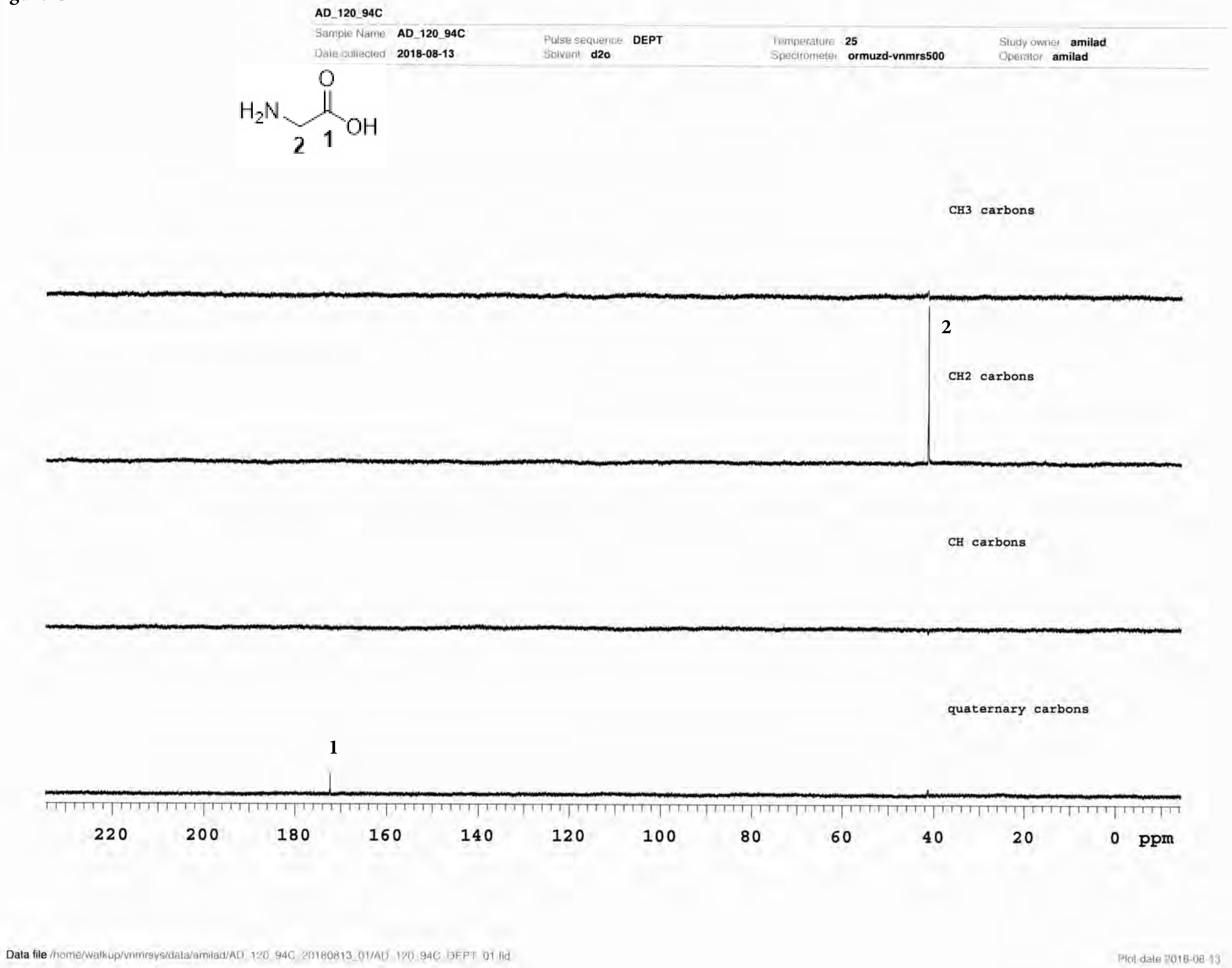

AD_120_94C
Sample Name AD_120_94C
Date collected 2018-08-13
Pulse sequence DEPT
Solvent d2o
Temperature 25
Spectrometer ormuzd-vnmrs500
Study owner amilad
Operator amilad
H_2N
O
OH
2
1
CH3 carbons
2
CH2 carbons
CH carbons
quaternary carbons
1
220
200
180
160
140
120
100
80
60
40
20
0 ppm
Data file /home/walkup/vnmrsys/data/amilad/AD_120_94C_20180813_01/AD_120_94C_DEPT_01.fid
Plot date 2018-08-13

Figure H

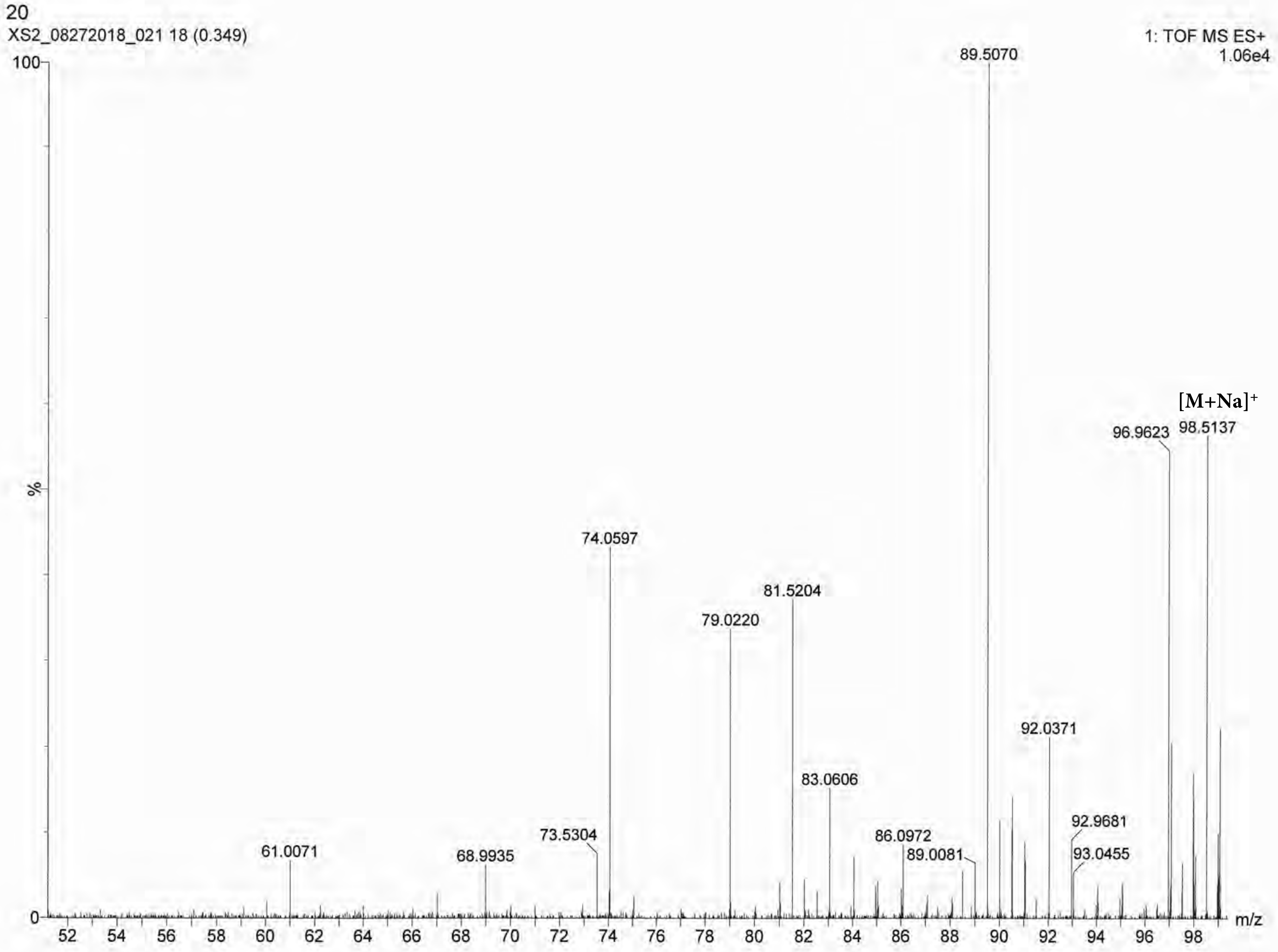
20
XS2_08272018_021 18 (0.349)
1: TOF MS ES+
1.06e4
100
%
0
89.5070
[M+Na]+
96.9623
98.5137
74.0597
81.5204
79.0220
92.0371
83.0606
92.9681
73.5304
86.0972
61.0071
68.9935
89.0081
93.0455
52
54
56
58
60
62
64
66
68
70
72
74
76
78
80
82
84
86
88
90
92
94
96
98
m/z
